# Quality of mobility measures among individuals with acquired brain injury: an umbrella review

**DOI:** 10.1007/s11136-022-03103-4

**Published:** 2022-03-11

**Authors:** Rehab Alhasani, Claudine Auger, Matheus Paiva Azevedo, Sara Ahmed

**Affiliations:** 1 School of Physical and Occupation Therapy, Faculty of Medicine, McGill University, Montreal, Canada; 2 Centre for Interdisciplinary Research in Rehabilitation of Greater Montreal (CRIR), Montreal, Canada; 3 Constance Lethbridge Rehabilitation Center, CIUSSS Centre-Ouest de l’Île de Montreal, Montreal, Canada; 4 School of Rehabilitation, Faculty of Medicine, University of Montreal, Montreal, Canada; 5 Site Institut Universitaire sur la Réadaptation en Déficience Physique de Montréal (IURDPM), CIUSSS Centre-Sud-de-l’Île-de-Montréal, Montreal, Canada; 6 Department of Rehabilitation Sciences, College of Health and Rehabilitation Sciences, Princess Nourah Bint Abdulrahman University, Riyadh, Saudi Arabia

**Keywords:** Umbrella review, Acquired brain injury, Mobility, Measures, Psychometrics

## Abstract

**Background and objective:**

While several mobility measures exist, there is large variability across measures in how mobility is conceptualized, the source of information and the measurement properties making it challenging to select relevant mobility measures for individuals with acquired brain injury (ABI). Therefore, the objective was to conduct a comprehensive synthesis of existing evidence on the measurement properties, the interpretability and the feasibility of mobility measures from various sources of information (patients, clinicians, technology) using an umbrella review of published systematic reviews among individuals with ABI.

**Methods:**

Ovid MEDLINE, CINHAL, Cochrane Library and EMBASE electronic databases were searched from 2000 to March 2020. Two independent reviewers appraised the methodological quality of the systematic reviews using the Joanna Briggs Institute critical appraisal checklist. Measurement properties and quality of evidence were applied according to COnsensus-based Standards for the Selection of Health Measurement Instrument (COSMIN) guidelines. Mobility measures were categorized using international standards with the international classification of functioning, disability and health (ICF).

**Results:**

Thirty-five systematic reviews were included covering 147 mobility measures, of which 85% were mapped to the ICF Activity and Participation component. Results showed an acceptable overall "sufficient" rating for reliability, construct validity and responsiveness for 132 (90%), 127 (86%) and 76 (52%) of the measures, respectively; however, among these measures, ≤ 25% of the methods for evaluating these properties were rated as ‘high’ quality of evidence. Also, there was limited information that supports measure feasibility and scoring interpretability.

**Conclusions:**

Future systematic reviews should report measures’ content validity to support the use of the measure in clinical care and research. More evaluations of the minimal important difference and floor and ceiling effects are needed to help guide clinical interpretation.

**Registration information:**

International Prospective Register of Systematic Reviews (PROSPERO); ID: CRD42018100068.

**Supplementary Information:**

The online version contains supplementary material available at 10.1007/s11136-022-03103-4.

## Summary

This umbrella review presents a comprehensive synthesis of measurement properties, interpretability and feasibility of mobility measures, from various sources of information (patients, clinicians, technology) among individuals with acquired brain injury including stroke and traumatic brain injury. Both researchers and clinicians search for measures that can be used to evaluate the impact of interventions and monitor changes in patients’ health. Mobility is a key focus of rehabilitation and essential to prepare individuals to return to the community. Evaluating the interplay between the determinants that influence mobility is essential to better understand what influences each patient’s mobility and tailor interventions to meet their needs. This review comprised 147 mobility measures. Results showed an acceptable overall "sufficient" rating for reliability, construct validity and responsiveness for 132 (90%), 127 (86%) and 76 (52%) of the measures, respectively; however, among these measures ≤ 25% of the methods for evaluating these properties were rated as ‘high' quality of evidence. Using different sources of information to measure mobility among individuals with acquired brain injury provides complementary information allowing us to incorporate the self-administered questionnaires, clinical data and data from technology measures to evaluate factors that cannot be readily reported, to support decision making in rehabilitation care. Thus, this review presents the characteristics, application, measurement properties, interpretability and feasibility of mobility measures.

## Introduction

Acquired brain injury (ABI), including traumatic brain injury (TBI) and stroke, is the leading cause of disability worldwide [1–3]. According to the World Health Organization, the global incidence of all-severity TBI is estimated at 69 million people, while 15 million people suffer a stroke worldwide each year [4–6]. Statistics Canada indicates that 100,000 Canadians will experience a stroke (59%) or a TBI (71%) each year [5]. Among the 1.5 million Canadians with ABI that go through the care continuum annually; over 60% report ongoing restrictions in mobility and participation in societal roles [5]. Webber et al. [7] defined mobility broadly as the ability to move oneself within environments that expand from one's home to the neighbourhood and regions beyond. It identifies five "key" interrelated determinants of mobility: cognitive, psychosocial, physical, environmental and financial influences. The multidimensionality and complexity of all domains that encompass mobility are also reflected in the international classification, functioning, disability and health Framework (ICF) mobility core set [8]. The ICF classifies mobility under body function including motion of all body bones and joints. In the activities and participation section, mobility is given an entire chapter and it is about moving by changing body position or location; or by transferring from one place to another, by using the upper extremity in carrying, moving or manipulating objects, by walking, running, or climbing, and by using various forms of transportation. In the environmental factors section, mobility is classified as products, devices, domesticated animals and services used for transportation [8].

Appropriate outcome measures are critical to accurately characterize and monitor changes in mobility during rehabilitation interventions for individuals with ABI [9]. However, selection of the best measure is difficult given the vast number of measures available, and the often unclear distinctions between them. While published guidelines recommend the use of valid, reliable and responsive assessment tools [10–13], guidance does not extend to which outcome measures are optimal for particular evaluative needs [14–18]. Researchers and clinicians also need to consider the content of measures and whether the domains evaluated match research and clinical objectives. A comparative examination of mobility measures will provide researchers and clinicians with the information needed to select the best outcome measure(s) to address the impairments, activity limitations and participation restrictions experienced by individuals with ABI. The ICF framework can be used to systematically classify the different domains of available outcome measures and, therefore, provide an additional basis for selection of a measure, based on comparison of the content [8].

There are also different sources of information of mobility measures. Outcomes that can only be assessed by asking the person directly are termed patient-reported outcomes (PROs) while clinician-reported outcomes (ClinROs) involve clinical judgement. Performance-reported outcomes (PerfOs) require patient cooperation and motivation [19]. Technology-based outcomes (TechOs) include sensors or assistive technologies to capture community mobility [20]. Self-reported outcomes (SROs) are not the same as PROs because SROs are outcomes that can be reported by the person with ABI but also observed and scored by someone else [22, 24, 25]. Most existing reviews on measuring mobility among individuals with ABI are limited to physical aspects and do not account for an expanded definition of mobility that encompasses mobility determinants [21–27]. Many walking measures are available and provide an index of what an individual can do or believes they can do, but the extent to which they indicate actual performance in the home environment is limited [28]. Life-space measures attempt to capture broader mobility, including mobility inside and outside the home, within the neighbourhood and beyond [29]. However, life-space measures do not capture transportation patterns or community engagement directly. To date, reviews have indicated that no measure evaluates mobility holistically among individuals with ABI.

Without considering the multidimensional nature of mobility, evaluations will inadequately prepare individuals to return to the community post-rehabilitation and limit our ability to correctly identify interventions which target factors that influence mobility in a given context. Clinicians require information on the content of measures to select comprehensive measures of mobility, as well as on measurement properties to ensure the minimum decision criteria to personalize care and deliver high-quality rehabilitation.

Moreover, Clinicians and clinical researchers may be unfamiliar with how to interpret the score of the mobility measure. They may not understand or have reference to the usual distribution of scores of a particular measure in a clinical or general population. Distribution of scores constitutes the absence of a problem, or meaningful changes in scores are needed for clinicians to know what cut-points of scoring indicate an action is warranted. Without reference values from a comparable population, researchers will not know whether an observed difference between two groups is meaningful, and whether a given change within or between groups is important [30]. In addition, the feasibility of using a measure (i.e. the time, cost required, length of the instrument, type and ease of administration) is another important aspect for a well-considered selection of the most appropriate measure [30, 31]. Thus, this study aimed to address these gaps by conducting a comprehensive synthesis of existing evidence on the measurement properties, the interpretability and the feasibility of mobility measures using an umbrella review [32] of published systematic reviews among individuals with ABI.

### Objective

An umbrella review of published systematic reviews among individuals with ABI was used to conduct a comprehensive synthesis of existing evidence on the measurement properties, the interpretability and the feasibility of mobility measures from various sources of information (patients, clinicians, technology).

### Methods

**Fig. 1:**
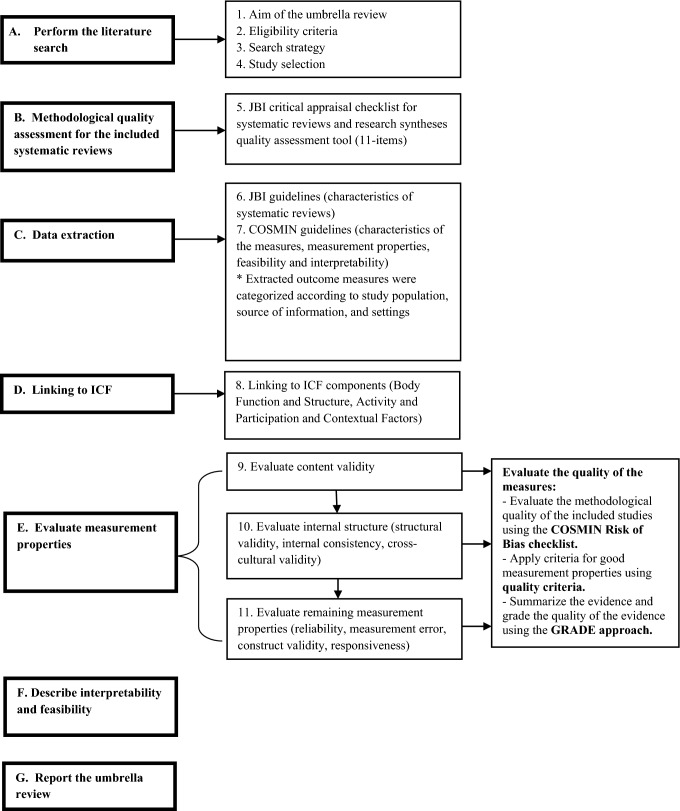
Steps of conducting the umbrella review

This umbrella review was reported according to both the Joanna Briggs Institute (JBI) guidelines for conducting an umbrella review [33] and the COnsensus-based Standards for the Selection of Health Measurement Instrument (COSMIN) guidelines for systematic reviews of outcome measures [31] (Fig. 1). The reason for conducting a JBI umbrella review was to summarize evidence from existing research syntheses on the properties of mobility measures, making use of the work already completed in this area [33]. Given that the JBI umbrella review guidelines did not focus on providing a rigorous methodology to assess the measurement properties and describe the interpretability and the feasibility of an instrument, the COSMIN guidelines were used [31].

### Eligibility criteria

The inclusion criteria were systematic reviews published in peer-reviewed journals. Systematic reviews were included if they met all of the following criteria: (1) individuals with ABI (Stroke, TBI) ≥ 18 years; (2) report a clear objective to identify measures of mobility; and (3) evaluate at least one measurement property of the measures. The exclusion criteria were reviews investigating the effectiveness of interventions, monitoring recovery, focusing on diagnostic screening, clinical commentaries, case reports, non-structured reviews, qualitative reviews, non-human studies and grey literature.

### Search strategy

A search of the literature was performed using electronic databases of Ovid MEDLINE, CINHAL, Cochrane Library and EMBASE. The search was conducted in collaboration with a health sciences librarian to ensure that the review included the appropriate and necessary keywords. The initial search strategy was constructed for Ovid MEDLINE (SI. 1) and adapted to other databases. A combination of Medical Subject Headings (MeSH) terms, subject headings and/or key words was used. Three groups of terms were generated describing the following: (1) the population ‘acquired brain injury’ AND, (2) the outcome measure ‘mobility’ AND and (3) the psychometric properties using a sensitive validated search filter [34]. Terms within each group were combined with the Boolean operator ‘OR’. Because the search included different types of studies, the search was narrowed by filtering the search specifying the type of studies including systematic review, review and meta-analyses. This filter has been used to avoid missing important information related to mobility measures. Searches were run in July 2019 (*n* = 32) with an updated search in March 2020 (*n* = 35).

### Study selection

All identified systematic reviews were uploaded into ENDNote X9.1 (Clarivate Analytics, PA, USA) and duplicates were removed. Two independent reviewers screened titles and abstracts of each systematic review against the eligibility criteria. Then, full text of the included systematic reviews was retrieved and evaluated for eligibility. Disagreements were resolved by discussion and consensus. The reference list of the articles included for the full-text screening was also hand-searched for additional identification of relevant systematic reviews. The Preferred Reporting Items for Systematic reviews and Meta-Analyses (PRISMA) flow diagram [35] were used to guide the selection process.

### Data extraction

Two reviewers independently extracted descriptive data from the included systematic reviews based on both JBI data extraction tool for Systematic Reviews and Research Syntheses [33] and COSMIN guidelines [31]. We extracted the characteristics of each systematic review, characteristics of mobility measures, healthcare settings or recovery phase where the mobility measure was used (if possible), results on the measurement properties, the interpretability of the scores of the measure and the feasibility of the measure. Extracted outcome measures were categorized according to the study population, sources of information and settings.

### Linking to the ICF

**Fig. 2:**
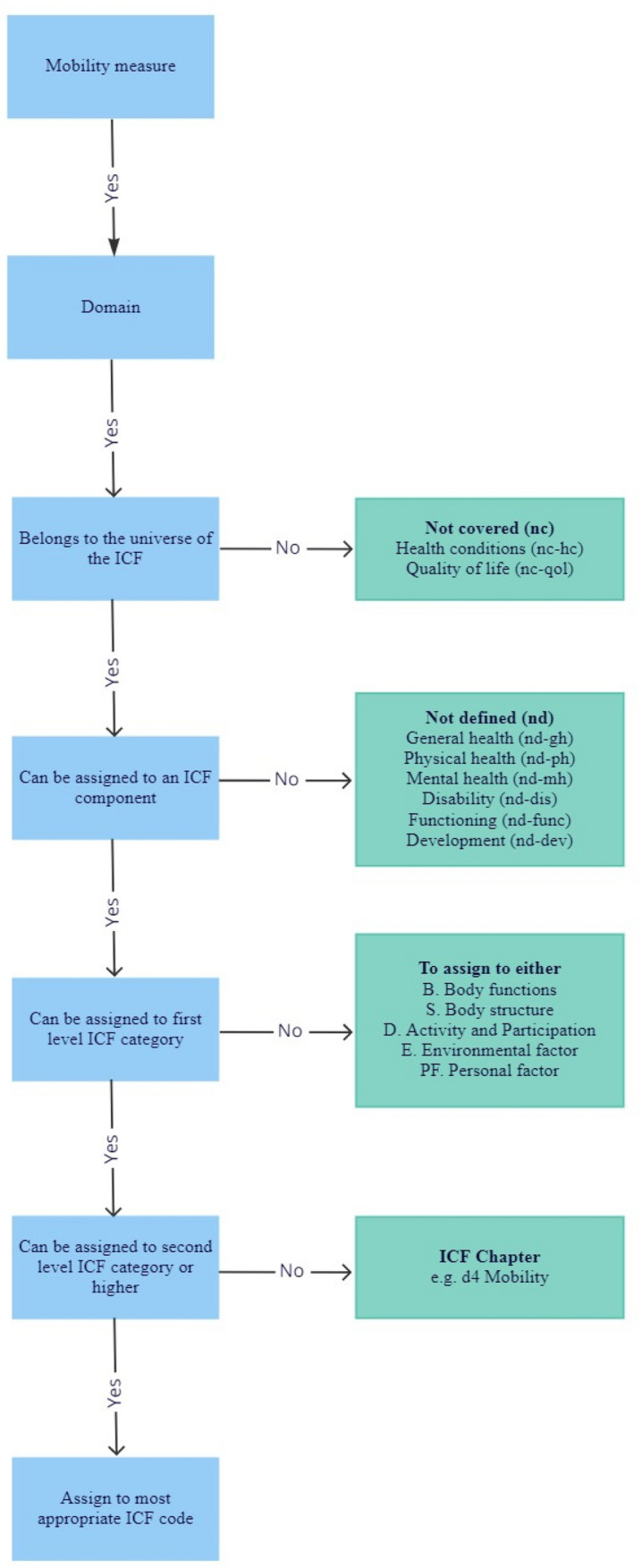
The international classification of functioning, disability and health linking decision tree

Each extracted mobility measure was linked to the ICF according to a set of linking rules [36] (Fig. 2). A measure can be linked to one or more ICF components (body functions and structures, activity and participation and contextual factors), depending on the number of constructs contained in each measure.

### Appraising methodological quality

The JBI critical appraisal checklist for systematic reviews and research syntheses quality assessment tool that includes 11 items was used to evaluate the quality of the systematic reviews [33]. In addition, the 4-point COSMIN rating scale was used to evaluate the methodological quality for evaluating the measurement properties of each study included in a given systematic review. The checklist consists of 10 measurement properties, each with their own quality criteria, which form three domains (content validity, internal structure and remaining measurement properties) [31, 37]. Each study was rated as very good, adequate, doubtful, or inadequate quality. Two independent reviewers evaluated the methodological quality followed by discussions and consensus [31, 37].

## Levels of evidence appraisal

Based on COSMIN guidelines [31], for each study in a systematic review, the estimate of the measurement property was rated against the updated criteria for good measurement properties [38]. Each estimate was rated as sufficient (+), insufficient (−), or indeterminate (?). A level-of-evidence appraisal was undertaken to determine the overall quality of each measurement property for a given measure across all studies reported in the systematic reviews. The appraisal produced a final rating for each measure for each of the measurement properties. All available information was synthesized, combining the results quantitatively into one overall category of the different studies for each measure. The overall rating for the summarized results was then rated as sufficient (+), insufficient (−), inconsistent (±), or indeterminate (?) [31, 39]. The quality of the evidence was graded by using the modified-Grading of Recommendations Assessment, Development and Evaluation (modified-GRADE) approach and the quality of the evidence was graded as high, moderate, low, or very low [31]. Two independent reviewers completed the evaluation before consensus discussions.

## Measurement properties

The psychometric results reported in the systematic reviews were described and categorized into the following COSMIN measurement properties including content/structural validity, internal structure, reliability, measurement error, construct validity and responsiveness. Table 1 presents the updated criteria for good measurement properties based on COSMIN guidelines [31].

**Table 1:** Updated criteria for good measurement properties

Measurement property	Rating^a^	Criteria
Structural/content validity	+	CTT CFA: CFI or TLI or comparable measure > 0.95 OR RMSEA < 0.06 OR SRMR < 0.08^b^ IRT/Rasch No violation of unidimensionality^c^: CFI or TLI or comparable measure > 0.95 OR RMSEA < 0.06 OR SRMR < 0.08 AND no violation of local independence: residual correlations among the items after controlling for the dominant factor < 0.20 OR Q3’s < 0.37 AND no violation of monotonicity: adequate looking graphs OR item scalability > 0.30 AND adequate model fit IRT: χ2 > 0.001 Rasch: infit and outfit mean squares ≥ 0.5 and ≤ 1.5 OR Z-standardized values > − 2 and < 2
	?	CTT: not all information for ‘ + ’ reported IRT/Rasch: model fit not reported
	−	Criteria for ‘ + ’ not met
Internal consistency	+	At least low evidence^d^ for sufficient structural validity^e^ AND Cronbach’s alpha(s) ≥ 0.70 for each unidimensional scale or sub-scale^f^
	?	Criteria for At least low evidence for sufficient structural validity^e^ not met
	−	At least low evidence for sufficient structural validity AND Cronbach’s alpha(s) < 0.70 for each unidimensional scale or sub-scale^f^
Reliability	+	ICC or weighted Kappa ≥ 0.70
	?	ICC or weighted Kappa not reported
	−	ICC or weighted Kappa < 0.70
Measurement error	+	SDC or LoA < MIC^e^
	?	MIC not defined
	−	SDC or LoA > MIC^e^
Hypotheses testing for construct validity	+	The result is in accordance with the hypothesis^g^
	?	No hypothesis defined (by the review team)
	−	The result is not in accordance with the hypothesis^g^
Responsiveness	+	The result is in accordance with the hypothesis^g^ OR AUC ≥ 0.70
	?	No hypothesis defined (by the review team)
	−	The result is not in accordance with the hypothesis^g^ OR AUC < 0.70

The criteria are based on COSMIN guidelines

*AUC* area under the curve, *CFA* confirmatory factor analysis, *CFI* comparative fit index, *CTT* classical test theory, *DIF* differential item functioning, *ICC* intraclass correlation coefficient, *IRT* item response theory, *LoA* limits of agreement, *MIC* minimal important change, *RMSEA* root mean square error of approximation, *SEM* standard error of measurement, *SDC* smallest detectable change, *SRMR* standardized root mean residuals, *TLI* Tucker–Lewis index

^a^ ‘+’ = sufficient,‘− ’ = insufficient, ‘?’ = indeterminate

^b^To rate the quality of the summary score, the factor structures should be equal across studies

^C^unidimensionality refers to a factor analysis per subscale, while structural validity refers to a factor analysis of a (multidimensional) patient‐reported outcome measure

^d^As defined by grading the evidence according to the GRADE approach

^e^This evidence may come from different studies

^f^The criterion ‘Cronbach alpha < 0.95’ was deleted, as this is relevant in the development phase of a PROM and not when evaluating an existing PROM

^g^The results of all studies should be taken together and it should then be decided if 75% of the results are in accordance with the hypotheses

### Evaluate content validity

Content validity is defined as ‘the degree to which the content of the outcome measure is an adequate reflection of the construct to be measured’ and is considered the most important measurement property [40]. In the COSMIN guidelines, Terwee et al. [41] describe three aspects of content validity, including relevance, comprehensiveness and comprehensibility.

### Evaluate internal structure

Internal structure refers to the relation among different items in the outcome measure. The evaluation of the internal structure includes an evaluation of the following:

*Structural validity* is defined as ‘the degree to which the scores of the outcome measures are an adequate reflection of the dimensionality of the construct to be measured’ [31].

*Internal consistency* is defined as ‘the degree of interrelatedness among the items’ [31].

*Cross-cultural validity* is defined as ‘the degree to which the performance of the items on a translated or culturally adapted outcome measure is comparable with the performance of the original version of the outcome measure’ [31].

### Evaluate the remaining measurement properties

*Reliability* is defined as ‘the degree to which the measurement is free from measurement error’ [31].

*Measurement error* is defined as ‘the systematic and random error of a patient’s score that is not attributed to true changes in the construct to be measured’ [31].

*Construct validity* is defined as ‘the degree to which the scores of an instrument are consistent with hypotheses (for instance, with regard to internal relationships, relationships to scores of other instruments, or differences between relevant groups) based on the assumption that the instrument validly measures the construct to be measured’ [31].

*Responsiveness* is defined as ‘the ability of an instrument to detect change over time in the construct to be measured’ [31].

## Describe interpretability and feasibility

Interpretability and feasibility are not measurement properties, because they do not refer to the quality of the outcome measure. However, they are considered important aspects for a well-considered selection of the outcome measure.

*Interpretability* is defined as ‘the degree to which one can assign qualitative meaning, that is, clinical or commonly understood connotations to an instrument’s quantitative scores or change in scores‘. It includes distribution of the scores in the study population, floor and ceiling effect, minimal important change and minimal important difference [31].

*Feasibility* is defined as ‘the ease of application of the measure in its intended setting, given constraints such as time or money‘. It includes type and ease of administration, length of the instrument, completion time, ease of score calculation, cost of the instruments and required equipment available in different settings [31].

## Results

### Search results

The search strategy yielded a total of 35 systematic reviews. Figure 3 in supplementary presents the PRISMA flow diagram including the selection process and reasons for exclusion.

### Characteristics of the systematic reviews

The 35 systematic reviews were published between 2004 and 2019 in peer-reviewed journals. Nine reviews focused mainly on ClinRO/PerfO, 7 on PRO/SRO; 2 on TechO and 17 reports mixed sources of information. Twenty-six reviews targeted individuals with stroke [16–18, 22, 24, 26, 42–61], three targeted both stroke and TBI [21, 62, 63], one targeted TBI [64] and five incorporated stroke and TBI as part of a wider population search [23, 25, 27, 28, 65]. 320 mobility measures were extracted from the systematic reviews. After removing the duplicates, 147 measures were identified; some measures were used in multiple healthcare settings. The included systematic reviews did not specify the recovery phase for individuals with TBI (SI. 2).

### Linking to the ICF

The 147 mobility measures covered the component of activities and participation (85%), followed by body functions (30%) (Table 2).

**Table 2:** Linking to the International Classification of Functioning, Health and Disability Framework (ICF)

Name of the measure	Area of assessment	Number of domain/items	Activity and participation	Body function	Environmental factors	Personal factors
Clinician-Reported Outcome (ClinROs)						
Action Research Arm test (ARAT)	ADL, Coordination, Dexterity, Upper extremity function	4 domains and 19 items	x			
Actual Amount of Use Test (AAUT)	ADL, Dexterity, Upper extremity function	14 items	x			
Balance Assessment in Sitting and Standing Position (BASSP)	Functional mobility	2 items	x	x		
Box and Block test	ADL, Coordination, Dexterity, Upper extremity function	1 items	x			
Brunel Balance Assessment	Balance	12 items	x			
Chedoke McMaster Stroke Assessment (CMSA)	Functional mobility	Impairment: 6 domains; Activity: Gross motor function: 10 items; Walking index: 5 items	x	x		
Four Square Step	ADL and Balance	1 item	x			
Frenchay Arm Test (FAT)	ADL, Upper extremity function, Dexterity	5 items	x			
Fugl-Meyer Assessment (FMA)	ADL, Functional mobility, Pain	5 domains and 226 items		x		
Fugl-Meyer Assessment-Upper extremity (FMA-UE)	Upper extremity function	33 items		x		
Fugl-Meyer test-Balance subscale (FM-B)	Balance	7 items		x		
Functional Ambulation Category (FAC)	Functional mobility and Gait	1 item	x			
Functional Ambulation Classification Hospital (FACHS)	Functional ambulation	1 item	x			
Functional Independence measure (FIM)	ADL	18 items (Motor tasks: 13; Cognitive tasks: 5)	x	x	x	
Functional Test for the Hemiplegic Upper Extremity (FTHUE)	Upper extremity functioning	7 domains	x	x		
Grip strength	Strength, Upper extremity	1 item		x		
Grooved Pegboard Test (GPT)	Coordination, Dexterity	5 items		x		
Manual Function Test (MFT)	Strength	Determined by the number of muscles being tested				
Mini Mental State Examination (MMSE)	ADL and Cognition	7 domains and 11 items		x		
Modified Ashworth Scale	Spasticity	Depends on number of muscles/joints tested				
Modified Emory Functional Ambulation Profile (M-EFAM)	Functional ambulation	5 items	x			
Motor Assessment Scale (MAS)	ADL and Functional mobility	8 items	x			
Motor Assessment Scale-Upper limb (MAS-UL)	Upper extremity function	6 items	x			
Motor Evaluation Scale for Upper Extremity in Stroke Patients (MESUPES)	Dexterity, ROM, Upper extremity function	17 items (Arm function: 8 items, Hand function: 9 items)	x			
Motor Free Visual Perception Test	Vision and Perception	36 items		x		
Motor status scale	Upper extremity function, ROM	4 domains		x		
Motricity index (MI)	Upper extremity function and Functional mobility	6 items				
National Institute of Health Stroke Scale (NIHSS)	Aphasia, Behaviour, Cognition, Dysarthria, Vision and Perception	15 items		x		
Neurobehavioural Cognition Status Exam (NCSE)	Cognition	62 items		x		
Nine-Hole Peg test (NHPT)	Dexterity, Upper extremity Function	1 item	x	x		
Ottawa Sitting Scale (OSS)	Functional mobility	1 item	x	x		
Pens taped to feet	Motor control	1 item		x		
Quadriplegia Index of Function	ADL	37 items	x			
Sitting Rising Test (SRT)	Functional mobility and Balance	1 item	x			
Sodring motor evaluation for stroke patients	Motor Function	2 domains and 32 items	x			
Step test	Balance	1 item	x			
Stroke Arm Ladder (SAL)	Upper extremity Function	34 items	x			
Stroke Rehabilitation assessment of movement (STREAM)	Coordination, Functional mobility, ROM	3 domains and 30 items	x	x		
Trunk Control Test (TCT)	Balance, Functional mobility	4 items				
Trunk Impairment Scale	Balance, Coordination, Functional mobility	17 items	x			
Trunk Recovery Scale (TRS)	Recovery	12 item		x		
Upper Body Dressing Scale (UBDS)	Upper body dressing	7 items	x	x		
Upper Extremity Functional Index (UEFI)	Upper extremity function	20 items	x	x		
Upper Extremity Performance Test for Elderly (Test d’Evaluation des Members supérieurs de Personnes Agées (TEMPA)	Upper extremity function	9 items	x	x		
Van Lieshout Test	Dexterity, Functional mobility, ROM	10 items	x			
Observer-reported Outcome (Obser))						
Activities of Daily Living scale	Functional mobility	25 items	x			
Functional Arm Activity Behavioural Observation System (FAABOS)	Behaviour, Activity		x	x		
Performance-Reported Outcomes (PerfOs)						
10-m Walking test	Functional mobility, Gait	1 item	x			
12-m Walking test	Functional mobility, Gait	2 item	x			
2-m Walking test	Functional mobility, Gait	3 item	x	x		
300mWT (Three hundred metre Walk Test in community)	Functional mobility, Gait	1 item	x			
30mCWT (Thirty metre Comfortable Walk Test)	Functional mobility, Gait	1 item	x			
3-m Walking test	Functional mobility, Gait	1 item	x			
4mCWT (Four metre Comfortable Walk Test)	Functional mobility, Gait	1 item	x			
5-m Walking test	Functional mobility, Gait	1 item	x			
6-min Walking test	Functional mobility, Gait	1 item	x			
Arm motor ability test (AMAT)	ADL and Upper extremity function	13 items	x			
Assessment of life habit (LIFE-H)	ADL, Communication, ADL, Executive functioning, Life participation, Quality of life	2 domains and 77 items	x			
Assessment of motor and process skills (AMPS)	ADL, Attention and Working memory, Executive functioning, Insight, Processing speed, Reasoning, Balance, Coordination, Functional mobility, Gait	36 items (ADL motor skill: 16; ADL process skill: 20)	x			
Balance evaluation system test (BESTest)	Balance, Gait and Strength	6 domains and 36 items	x			
Barthel index (BI)	ADL, Functional mobility, Gait	10 items	x			
Berg balance scale (BBS)	Balance and Functional mobility	14 items	x			
Berg balance scale three point (BBS-3P)	Balance and Functional mobility	7 items	x			
Chedoke Arm and Hand Inventory (CAHAI)	ADL and Upper extremity function	13 items	x			
Community balance and mobility scale (CB&M)	Balance and Functional mobility	13 items	x			
Dynamic Gait index (DGI)	Balance, Functional mobility, Gait	8 items	x			
Fitts reaching test	Balance	4 items		x		
Five times sit to stand test (5xSTST)	Functional mobility and Strength	1 item	x			
Function in sitting Test (FIST)	Balance	14 items	x			
Functional Gait assessment (FGA)	Balance and Gait	10 items	x			
Grasp-release test	Upper extremity function	6 items	x	x		
High-level Mobility Assessment (HiMAT)	Functional mobility, Vestibular	5 domains and 13 items	x	x		
Jebsen hand function test (JHFT)	ADL, Upper extremity function	7 items	x			
Modified functional reach test (MFRT)	Balance, Functional mobility and Vestibular	1 item	x			
Postural assessment scale for stroke patients (PASS)	Balance	12 items	x	x		
Postural assessment scale for stroke patients trunk control (PASS-TC)	Balance	12 items		x		
Postural control and balance for stroke (PCBS)	Balance	12 items	x	x		
Rivermead motor assessment (RMA)	Functional mobility	38 items	x			
Short form berg balance scale (SFBBS)	Balance and Functional mobility	7 items	x			
Short form of the wolf motor function test (S-WMFT)	Dexterity, Strength, Upper extremity function	6 items	x			
Short Form Postural Assessment scale for stroke patients-6 items (6 SFPASS)	Balance	6 items	x			
Sollerman hand function test	Functional mobility	20 items	x	x		
Three point postural assessment scale for stroke patients (PASS-3P)	Balance	6 items	x	x		
Timed up and go test (TUG)	Balance, Functional mobility, Gait, Vestibular	2 trials	x			
Timed walk	Gait, Balance	3 trials	x	x		
Wolf motor function test (WMFT)	Dexterity, Strength, Upper extremity function	21 items	x			
Patient-reported outcomes (PROs)						
ABILHAND	ADL, Dexterity, Upper extremity function	23 items	x			
Activity Card Sort (ACS)	ADL, Life participation	4 domains and 89 items (IADL: 20; low physical demand leisure activities: 35; high physical demand leisure activities: 17; social activities: 17)	x			
Beck depression inventory (BDI)	Depression	21 items		x		
Brain injury community rehabilitation outcome scale (BICRO)	Community functioning in areas of Activity, Social participation and Psychological components	39 items	x	x		
Canadian occupational performance measure (COPM)	ADL, Functional mobility, Life participation	3 domains and 9 items	x			
Centre for epidemiological studies depression	Depression	20 items		x		
Climbing stairs questionnaire (CSQ)	Climbing stairs	15 items	x	x		
Coded activity diary	Physical activity and energy expenditure		x	x		
European quality of life scale-EQ5D	Functional mobility, ADL, Pain, Depression	5 domains and 6 items	x			
Geriatric depression scale-long form (GDS)	Depression	30 items		x		
Human activity profile (HAP)	ADL	94 items	x			
Leeds adults spasticity impact scale (LASIS)	Arm Function	12 items		x		
London handicap scale (LHS)	ADL, Functional mobility, Life participation, Quality of life, Social relationships	6 items	x			
Mayo-Portland adaptability inventory (MPAI-4)	Physical, cognition, emotional, behavioural, social and community re-integration	35 items	x			
Medical outcomes study 36-Item short form health survey (SF-36)	ADL, Quality of life	8 domains and 36 items	x	x		x
Modified rankin handicap Scale	ADL and functional mobility	1 item	x			
Nottingham extended ADL index (N-ADL)	ADL, independence, Functional mobility, leisure	4 domains and 22 items	x			
Nottingham leisure activity (NLA)	Leisure activities	38 items	x			
Outpatient physical therapy Improvement in movement assessment log (OPTIMAL)	Balance, Coordination, Dexterity, Functional mobility, Gait, Upper extremity function	22 items	x	x		
Physical ability scale (PAS)	ADL and Life participation	12 items	x			
Reintegration to normal living index (RNLI)	ADL, Social relationships	8 domains and 11 items	x			
Satisfaction with life scale (SWLS)	Life participation and Quality of life	5 items	x			
Sickness impact profile (SIP)	Behaviour, Life participation, Mental health, Social relationships	3 domains and 68 items	x			
Stroke impact scale (SIS)	ADL, Cognition, Communication, Depression, Functional mobility, Gait, General health, Life participation, Quality of life, Social relationships, Social support, Upper extremity function	8 domains and 59 items	x			
Stroke-specific quality of life scale (SSQOL)	Behaviour, Cognition, Functional mobility, Language, Personality, Negative affect, Quality of life, Social relationships, Upper extremity function	12 domains and 49 items	x			
Subjective index of physical and social outcome (SIPSO)	Domestic life, Major life areas, Transportation, Interpersonal interactions and relationship, Community, Recreational and civic life	5 domains and 26 items	x			
Self-reported outcomes (SROs)						
Disabilities of the Arm, Shoulder and Hand (DASH)	Upper extremity function	30 items	x			x
Duruoz hand index (DHI)	ADL, Coordination, Dexterity, Functional mobility, General health, Life participation, Upper extremity function	18 items	x			
Frenchay activities index (FAI)	ADL	3 domains and 15 items	x			
Hand function survey (HFS)	Hand Function	13 items	x	x		
International classification of functioning, health and disability-measure of participation and activities screener	Life participation	32 items	x			
Motor activity log (MAL-14)	Upper extremity function	14 items	x			
Motor activity log-26 items	Upper extremity function	26 items	x			
Motor activity log-28 items	Upper extremity function	14 items	x			
Multimedia activity recall for children and adults (MARCA)	Physical activity and energy expenditure	10 domains	x	x		
Rivermead mobility index (RMI)	Balance, Functional mobility and Gait	15 items	x			
Technology-Reported Outcomes (TechOs)						
Accelerometer (ActiGraph)	Activity		x			
Actical	Activity		x			
Actiwatch	Activity		x			
Ambulatory Monitoring (AM Accelerometer)	Activity		x			
Biaxial accelerometer	Activity		x			
Caltrac accelerometer	Activity		x			
Computer Science and Applications Inc. Model 7164 activity monitors × 4	Activity		x			
Dimensional gait analysis (3-DGA)	Activity		x			
Finger tapping (uniaxial accelerometer)	Activity		x			
Fitbit Ulta	Activity		x			
Footswitches	Activity		x			
Kinematics	Activity		x			
Nike + Fuelband	Activity		x			
OMRON HJ-113-E Piezoelectric Pedometers	Activity		x			
PAL2 (Gorman ProMed Pty. Ltd)	Activity		x			
Pedometers	Activity		x			
Sensewear Pro 3 Armband	Activity		x			
Smart Balance Master (SBM)	Activity		x			
SmartShoe	Activity		x			
StepWatch activity monitor or Step activity monitor (SAM)	Activity	1 item	x			
Stride analyzer system (SAS)	Activity		x			
The Intelligent Device for Energy expenditure and activity (IDEEA)	Activity		x			
Triaxial accelerometer/ RT3	Activity		x			
Uniaxial accelerometer	Activity		x			
Wireless triaxial accelerometers	Activity		x			

### Methodological quality

Based on JBI guidelines checklist, nine (26%) systematic reviews used a standardized methodology, either PRISMA guidelines [21, 49, 52–54, 57] or standardized accepted guidelines from previously published work [25, 46, 63]. Although the quality of evidence of the literature search and evaluation of measurement properties of the review was generally acceptable, a minority of systematic reviews (17%) [21, 26, 49, 52–54] used the COSMIN Risk of Bias checklist, which resulted in low quality of evidence (SI. 3). We have applied the 4-point COSMIN rating scale to evaluate the quality of studies in each included systematic review. Among the 147 mobility measures, we found that the quality for content/structural validity was rated as adequate or higher for 16 (11%), internal consistency for 45 (30%), reliability for 54 (36%), construct validity for 101 (68%) and responsiveness for 46 (67%) of measures (SI. 4). Many measurement properties were not reported, and there was inconsistent reporting between studies. None of the included systematic reviews reported cross-cultural validity, or criterion validity.

## Levels of evidence of measurement properties

Table 3 presents the level of evidence for the 147 mobility measures among individuals with ABI, categorized by population, sources of information and settings (For more information about the analysis, please refer to SI. 5, 6 & 7). Here, we present the overall level of evidence on measurement properties among all mobility measures across all settings, ABI population and source of information (*n* = 185).

**Table 3:** The overall rating of summarized measurement properties and the quality of evidence

Name of the measure	Content validity		Internal consistency		Reliability		Measurement error		Construct validity		Responsiveness	
	Overall rating	Quality of evidence	Overall rating	Quality of evidence	Overall rating	Quality of evidence	Overall rating	Quality of evidence	Overall rating	Quality of evidence	Overall rating	Quality of evidence
*Stroke at acute setting*												
*Clinician-reported outcomes (ClinROs)*												
Chedoke McMaster stroke assessment scale (CMSA) [43]					+	Low (− 2)			+	Low (− 2)	+	Low (− 2)
Fugl-Meyer assessment (FMA) [16, 47, 56]			+	Moderate (− 1)	+	Moderate (− 1)			+	Moderate (− 1)	+	Moderate (− 1)
Fugl-Meyer test-balance subscale (FM-B) [53]											+	High
Functional ambulation category (FAC) [46, 53]					+	Low (− 2)			–	Low (− 2)	+	High
Functional independence measure (FIM) [18]			+	Moderate (− 1)	+	Moderate (-1)			+	Moderate (− 1)	+	Moderate (− 1)
Manual function test (MFT) [48]			+	Moderate (− 1)	+	Moderate (-1)			+	Moderate (− 1)		
Mini mental state examination (MMSE) [43]					+	High			-	Moderate (− 1)		
Modified Ashworth scale (m-AS) [16, 27, 43, 56]					+	Low (− 2)			-	Low (− 2)		
Modified emory functional ambulation profile (M-EFAM) [53]											+	Low (− 2)
Motor assessment scale (MAS) [53]											+	Moderate (− 1)
Motor status scale (MSS) [56, 63]					+	Low (− 2)			+	Low (− 2)		
*Performance-reported outcomes (PerfOs)*												
6-Minute walking test (6MWT) [25, 52]	+	High			+	Moderate (− 1)	+	Moderate (− 1)	+	Moderate (− 1)		
10-Metre walking test (10MWT) [25, 53]					+	High					+	High
12-Metre Walking Test (12MWT) [25]					+	Low (− 2)						
2-Metre Waling Test (2MWT) [53]					+	Low (− 2)					+	Low (− 2)
Barthel index (BI) [18]			+	Low (− 2)	+	Low (− 2)			+	Low (− 2)	+	Low (− 2)
Berg balance scale (BBS) [18, 53]			+	Low (− 2)	+	Low (− 2)			+	Low (− 2)	+	High
Berg balance scale three point (BBS-3P) [53]											+	High
Function in sitting test (FIST) [57]	+	Low (− 2)	+	Low (− 2)			+	Low (− 2)	+	Low (− 2)		
Postural assessment scale for Stroke Patients (PASS) [53]											+	High
Postural assessment scale for Stroke Patients trunk control (PASS-TC) [53]											+	High
Postural control and Balance for stroke (PCBS) [53]											+	Moderate (− 1)
Rivermead mobility assessment (RMA) [18]					-	Moderate (− 1)			+	Moderate (− 1)		
Short form Berg balance scale (SFBBS) [53]											+	Moderate (− 1)
Short form postural assessment scale for Stroke patients-6 items (6 SFPASS) [53]											+	High
Three point postural assessment scale for Stroke patients (PASS-3P) [53]											+	High
*Patient-reported outcomes (PROs)*												
Beck depression inventory (BDI) [43]					+	High			+	High	-	Moderate (− 1)
European quality of life scale (EQ5D) [56]					+	Low (− 2)			−	Low (− 2)		
London handicap scale (LHS) [58]	+	High			+	High			+	High		
Modified Rankin Handicap scale (m-RHS) [43]					+	High			-	Moderate (− 1)	-	Moderate (− 1)
*Self-reported outcomes (SROs)*												
Frenchay Activities Index (FAI) [18]			+	Low (− 2)	+	Low (− 2)			+	Low (− 2)		
Rivermead mobility index (RMI) [18, 21]	+	Low (-2)	+	Low (− 2)	+	Low (− 2 )			+	Low (− 2)	+	Low (− 2)
*Technology-based outcomes (TechOs)*												
Actiwatch [45]									+	Moderate (− 1)		
Ambulatory monitoring (AM Accelerometer) [45]									+	Low (− 2)		
Smart balance master (SBM) [53]											+	Low (− 2)
Uniaxial accelerometer [45]									+	Moderate (− 1)		
Stroke at sub-acute setting												
*Clinician-reported outcomes (ClinROs)*												
Functional Ambulation Category (FAC) [25, 26, 53]	+	Moderate (− 1)			+	Moderate (− 1)			+	Moderate (− 1)	+	Moderate (− 1)
*Performance-reported outcomes (PerfOs)*												
10-Metre walking test (10MWT) [26, 46, 65]	+	Moderate (− 1)			+	Low (− 2)	+	Low (− 2)	+	Low (− 2)	+	Low (− 2)
12-Metre walking test (12MWT) [52]	+	Low (− 2)			−	Low (− 2)			+	Low (− 2)		
3-Metre walking test (3MWT) [52]	+	Low (− 2)			+	Low (− 2)			+	Low (− 2)		
6-Minute walking test (6MWT) [26, 52]	+	High			+	High	+	High	+	High		
Arm Motor Ability Test (AMAT) [24]			+	Low (− 2)	+	Low (− 2)			+	Low (− 2)	+	Low (− 2)
Berg Balance Scale (BBS) [46]					+	Low (− 2)			-	Low (− 2)	+	Low (− 2)
Dynamic Gait Index (DGI) [26]	+	Low (− 2)			+	Low (− 2)			+	Low (− 2)		
*Patient-reported outcomes (PROs)*												
Physical Ability Scale (PAS) [57]					−	Low − 2)						
Stroke impact scale (SIS) [59]	+	Low (− 2)	+	Low − 2)	+	Low − 2)			+	Low (− 2)		
*Self-reported outcomes (SROs)*												
Motor Activity Log-28 items (MAL-28) [24, 62]			+	High					−	Moderate − 1)		
Rivermead mobility index (RMI) [46]			+	Moderate (− 1)	+	Moderate (− 1)			−	Moderate − 1)	+	Moderate − 1)
*Technology-based outcomes (TechOs)*												
ActiGraph [45]					+	Low − 2)			+	Low − 2)		
Footswitches [26]	+	Low (− 2)			+	Low (− 2)	+	Low (− 2)	+	Low − 2)		
Stroke at chronic setting												
Clinician-reported outcomes (ClinROs)												
Action Research Arm test (ARAT) [23, 24, 27, 42, 44, 48, 55, 56, 63]	+	High	+	High	+	High			+	High	+	Low (− 2)
Actual amount of use test (AAUT) [48]					+	Low (− 2)			+	Low (− 2)		
Balance assessment in sitting and standing position (BASSP) [57]					+	High			+	High	+	High
Box and block test (BBT) [56, 63]					+	Moderate (− 1)			+	Low (− 2)	-	Low (− 2)
Brunel balance assessment (BBA) [51]	+	Moderate (− 1)			+	Moderate (− 1)			+	Moderate (− 1)		
Chedoke McMaster Stroke assessment scale (CMSA) [18, 44, 56]			+	High	+	High			+	High	+	High
Four Square Step (FSS) [51]	−	Low − 2)							+	Low (− 2)	−	Low (− 2)
Frenchay Arm Test (FAT) [43, 48, 56]					+	Moderate − 1)			+	Moderate (-1)	−	Low (− 2)
Fugl-Meyer Assessment (FMA) [27, 43, 44]					+	High			+	Low − 2)	+	Low (− 2)
Fugl-Meyer Assessment-Upper extremity (FM-UE) [23]	+	High			+	High			+	High		
Functional Ambulation Category (FAC) [25, 26, 60]	+	Low − 2)			+	Moderate (− 1)			+	Moderate (− 1)		
Functional Independence measure (FIM) [27, 28, 43, 50, 56]	+	High			+	Moderate (-1)			−	Moderate (− 1)	+	Moderate (− 1)
Functional Test for the Hemiplegic Upper Extremity (FTHUE) [48]					+	Moderate − 1)						
Functional Ambulation Classification Hospital (FACHS) [26]	+	Low − 2)							+	Low (− 2)		
Grip strength [56]					+	Low − 2)						
Mini Mental State Examination (MMSE) [16]			−	Moderate − 1)	+	Moderate (− 1)			+	Moderate (− 1)		
Modified Emory Functional Ambulation Profile (M-EFAM) [25, 51]					+	Moderate (− 1)			+	High	+	Low (− 2)
Motor Assessment Scale (MAS) [43, 44, 47, 56]					+	Low (− 2)			+	Low (− 2)	−	Low (− 2)
Motor Evaluation Scale for Upper Extremity in Stroke Patients (MESUPES) [23, 48]	+	High			+	High			+	High		
Motor Free Visual Perception Test [16, 43]			−	Low (− 2)	+	Low (− 2)			-	Low − 2)		
Motricity index (MI) [43, 44, 46, 47, 60]			+	Moderate − 1)	+	Moderate (− 1)			+	Moderate (− 1)		
National institute of health stroke scale (NIHSS) [43]					+	Moderate (− 1)			+	Moderate (− 1)	-	Moderate (− 1)
Neurobehavioural Cognition Status Exam (NCSE) [43]					−	Low (− 2)			+	Low (− 2)	+	Low (− 2)
Nine-Hole Peg test (NHPT) [44, 56, 63]					+	Moderate (− 1)			+	Moderate (− 1)		
Ottawa Sitting Scale (OSS) [57]					+	Moderate (− 1)						
Quadriplegia Index of Function [27]					+	Moderate (− 1)					−	Moderate (− 1)
Sitting Rising Test (SRT) [57]					+	Moderate (− 1)			+	Moderate (− 1)		
Sodring motor evaluation for stroke patients [47]			+	High	+	High			+	High	+	High
Step test [51]	+	Low (− 2)			+	Low − 2)			+	Low − 2)	+	Low (− 2)
Stroke Arm Ladder (SAL) [23]	+	High			+	High			+	High		
Stroke Rehabilitation assessment of movement (STREAM) [23, 42, 47, 63]	+	High	+	High	+	High			+	High	+	High
Trunk Control Test (TCT) [57, 60, 61]			+	Low (− 2)	+	Low (− 2)			+	Low − 2)		
Trunk Impairment Scale (TIS) [57, 61]	+	Moderate (− 1)	+	Moderate (− 1)	+	Moderate (− 1)	+	Low (− 2)	+	Low (− 2)	+	Moderate − 1)
Upper Body Dressing Scale (UBDS) [48]					+	Moderate (− 1)			+	Moderate (− 1)	+	Moderate (− 1)
Upper Extremity Functional Index (UEFI) [23]					+	High			+	High		
Upper Extremity Performance Test for Elderly (Test d’Evaluation des Membres supérieurs de Personnes Agées (TEMPA) [48]					+	Low (− 2)			+	Low (− 2)		
Upper Limb-Motor Assessment Scale (UL-MAS) [23, 24]	+	Moderate (− 1)	+	Moderate (− 1)	+	Moderate (− 1)			+	Moderate (− 1)	+	Low (− 2)
Van Lieshout Test Short Form [27]					−	Low (− 2)					−	Moderate (− 1)
*Observation-reported outcome (ObserO)*												
Activities of Daily Living scale (ADL scale) [48]					+	Moderate (− 1)			+	Moderate − 1)		
*Performance-reported outcomes (PerfOs)*												
10-Metre Walking Test (10MWT) [25, 26, 46, 60, 65]	+	High			+	High	+	Moderate (− 1)	+	High	+	Low (− 2)
12-Metre Walking Test (12MWT) [26, 52, 53]	+	Moderate (− 1)			+	Low (− 2)			+	Moderate (− 1)	+	Low (− 2)
2-Metre Walking Test (2MWT) [25, 26, 52]	+	High			+	High	+	High				
300-Metre Walking Test (300MWT) [26]	+	Low (− 2)			+	Low (− 2)			+	Low (− 2)		
30-Metre Walking Test (30MWT) [26]	+	Low (− 2)							+	Low (− 2)		
4-Metre Comfortable Walking Test (4MCWT) [26]	+	Low − 2)							+	Low (− 2)		
5-Metre Walking Test (5MWT) [25, 26, 52]	+	Moderate (− 1)			+	Low (− 2)	+	Low (-2)	+	Moderate (-1)	+	Moderate (-1)
6-Minute Walking Test (6MWT) [25, 26, 46, 52, 53, 65]	+	High			+	High	+	High	+	High	+	Low (-2)
Arm Motor Ability Test (AMAT) [23, 48, 56]	+	Low (− 2)			+	Low (− 2)						
Assessment of Life Habits (LIFE-H) [50, 59]	+	Moderate (− 1)			+	High			+	Moderate − 1)		
Assessment of Motor and Process Skills (AMPS) [48]					+	Moderate (− 1)			+	Moderate (− 1)		
Balance Evaluation System test (BESTest) [51]	+	High			+	High			+	High	+	High
Barthel Index (BI) [28, 43, 56, 60]					+	High			+	High	+	High
Berg Balance Scale (BBS) [43, 60]					+	Moderate (− 1)			+	Moderate (− 1)	+	Moderate (− 1)
Chedoke Arm and Hand Inventory (CAHAI) [24, 42, 48, 56]	+	High	+	High	+	High			+	High	+	Low (− 2)
Community balance and mobility scale (CB&M) [51]									+	Low (− 2)	+	Low (− 2)
Dynamic Gait Index (DGI) [26, 51]	+	Low (− 2)			+	Low (− 2)						
Fitts Reaching test [63]					+	Low (− 2)			−	Low (− 2)		
Five times Sit to Stand test (5xSTST) [54]	+	Moderate (− 1)			+	Moderate (− 1)	?	Low (− 2)	+	Low (− 2)	+	Low (− 2)
Functional Gait Assessment (FGA) [26]	+	Low (− 2)			+	Low (− 2)			+	Low (− 2)		
Grasp− Release test [27]			+	Low (− 2)							+	Moderate (− 1)
Jebsen Hand Function Test [27, 48]					+	Low (− 2)			+	Low (− 2)	+	Low (− 2)
Modified Functional Reach test (MFRT) [53, 57]					+	Low (− 2)					+	Low (− 2)
Modified Rankin Handicap Scale (m-RHS) [18]					+	Moderate (-1)			+	Moderate (− 1)	+	Moderate (− 1)
Postural Assessment Scale for Stroke Patients (PASS) [53, 57]											+	High
Postural Assessment Scale for Stroke Patients Trunk Control (PASS-TC) [53]											+	High
Rivermead mobility Assessment (RMA) [23, 43, 44, 47, 56, 63]			+	High	+	High	+	Low (− 2)	+	Low (− 2)	−	Low (− 2)
Sollerman hand function test [63]					+	Low (− 2)						
Timed Up and Go test (TUG) [18, 25, 43, 51, 65]	−	Low (− 2)			+	High			+	Moderate (− 1)	+	Moderate (− 1)
Timed walk [28]	+	Low (− 2)			+	Low (− 2)			+	Low (− 2)	+	Low (− 2)
Wolf Motor Function Test (WMFT) [23, 24, 27, 43, 48, 55, 56]	+	High	+	High	+	High			+	High	+	Low (− 2)
Patient-reported outcomes (PROs)												
ABILHAND [23, 24, 42, 48, 55, 56, 62, 63]	+	High	+	Low (− 2)	+	High			+	High	+	Low (− 2)
Activity Cart Sort (ACS) [49, 59]	+	Low (− 2)	+	Moderate (− 1)	+	Moderate (− 1)			+	Moderate (− 1)		
Beck Depression Inventory (BDI) [16]	+	High	+	High	−	Moderate (− 1)			+	High	−	High
Canadian Occupational Performance Measure (COPM) [48]					+	Low (− 2)			−	Low (− 2)	+	Low (− 2)
Centre for Epidemiological Studies Depression [43]			+	Low (− 2)	+	Low (− 2)			+	Low (− 2)	+	Low (− 2)
Climbing stairs questionnaire (CSQ) [21]	+	Low (-2)	+	Low (− 2)	+	Low (− 2)			+	Low (− 2)		
Coded activity diary [49]									−	Low (− 2)		
European Quality of life scale (EQ5D) [17, 43, 50, 58]					+	Low (− 2)			+	Low (− 2)	+	Low (− 2)
Frenchay Activities Index (FAI) [49, 50, 59, 60]	+	High	+	High	+	High			+	High	−	High
Geriatric Depression scale− long form (GDS) [43]			+	Low (− 2)	+	Low (− 2)			+	Low (− 2)	+	Low (− 2)
Human activity profile (HAP) [21, 49]			+	Low (− 2)	+	Low (− 2)			+	Low (− 2)	−	Low (− 2)
London Handicap scale (LHS) [58]	+	Low (− 2)	+	Low (− 2)	+	Low (− 2)			−	Low (− 2)		
Medical Outcomes Study 36-Item Short Form Health Survey (SF-36) [17, 27, 28, 43, 50]	+	Low (− 2)	+	Low (− 2)	−	Moderate (− 1)			+	Low (− 2)	−	Low (− 2)
Nottingham Extended ADL index (N-ADL) [21]	+	Moderate (− 1)	+	Moderate (− 1)	+	Moderate (− 1)			+	Moderate (− 1)	+	Moderate (− 1)
Nottingham leisure activity (NLA) [17, 49]			+	Low (− 2)	+	Low (− 2)			+	Low (− 2)		
Outpatient Physical Therapy Improvement in Movement Assessment Log (OPTIMAL) [23]									+	High		
Reintegration to normal living index (RNLI) [58]					+	Moderate (-1)			+	Moderate (− 1)		
Sickness Impact profile (SIP) [17, 43, 50]			+	Low (− 2)	+	High			+	Moderate (− 1)		
Stroke Impact Scale (SIS) [17, 21, 43, 50, 55, 56, 58]	+	Low (− 2)	+	Low (− 2)	−	Moderate (− 1)			+	High	+	Moderate (− 1)
Stroke-Specific Quality of Life Scale (SSQOL) [17, 43, 50]			+	Moderate (− 1)	−	Moderate (− 1)			+	Moderate (− 1)	+	Moderate (− 1)
Self− reported outcomes (SROs)												
Disabilities of the Arm, Shoulder and Hand (DASH) [23]			+	High	+	High			+	High		
Duruoz Hand Index (DHI) [48]					+	Moderate (− 1)			+	Moderate (− 1)		
Hand Function Survey (HFS) [48]					+	Low (− 2)			+	Low (− 2)		
International classification of functioning, health and disability-Activity measure (ICF-AM) [23]					+	High			+	High		
Motor activity log-14 items (MAL-14) [24, 27, 48, 62, 63]	+	Low (− 2)	+	Low (− 2)	+	Low (− 2)			+	High	+	Low (− 2)
Multimedia activity recall for children and adults (MARCA) [49]					+	Low (− 2)			−	Low (− 2)		
Rivermead mobility index (RMI) [25, 28, 47, 53]			+	Low (− 2)	+	Low (− 2)			+	High	+	Moderate (− 1)
Technology-based outcomes (TechOs)												
Actical [22]					+	Low (− 2)						
Actiwatch [45]									+	Low (− 2)		
Ambulatory Monitoring (AM Accelerometer) [26]									+	Low (− 2)		
Biaxial accelerometer [45]					+	Low (− 2)			+	Low (− 2)		
Caltrac accelerometer [22, 45]					−	Low (− 2)						
Computer Science and Applications Inc. Model 7164 activity monitors × 4 [22]									+	Low (− 2)		
Dimensional gait analysis (3-DGA) [45]									+	Low (− 2)		
Finger Tapping [45]									−	Moderate (− 1)		
Fitbit Ulta [22]									+	Low (− 2)	+	Low (− 2)
Footswitches [45]									+	Low (− 2)	+	Low (− 2)
Kinematics [56]					+	Low (− 2)			−	Low (− 2)	+	Low (− 2)
Nike + Fuelband [22]									+	Low (− 2)	+	Low (− 2)
PAL2 (Gorman ProMed Pty. Ltd) [22]									+	Low (− 2)		
Pedometers [22, 26, 45]					−	Low (− 2)			−	Low (− 2)	+	Low (− 2)
Sensewear Pro 3 Armband [22]									−	Low (− 2)	+	Low (− 2)
SmartShoe [22]									+	Low (− 2)	+	Low (− 2)
StepWatch Activity Monitor or Step Activity Monitor (SAM) [22, 25, 45]					?	Low (− 2)			+	High	+	Moderate (− 1)
Stride analyzer system (SAS) [45]					+	Low (− 2)			+	Low (− 2)		
The Intelligent Device for Energy Expenditure and Activity (IDEEA) [22, 45]					+	Low (− 2)						
Triaxial accelerometer/ RT3[22, 45]					+	Moderate (− 1)			+	Low (− 2)		
Wireless Triaxial Accelerometers [22]									+	Low (− 2)		
Traumatic brain injury												
Clinician− reported outcomes (ClinROs)												
Functional Independence measure (FIM) [64]	+	High			+	High						
Grooved Pegboard Test (GPT) [25]					+	Low (− 2)			−	Low (− 2)		
Pens taped to feet (PTF) [25]					+	Low (− 2)			+	Low (− 2)		
Trunk Recovery Scale (TRS) [57]			+	Moderate (− 1)	+	Moderate (− 1)	+	Moderate (− 1)	+	Moderate (− 1)		
*Observation-reported outcome (ObserO)*												
Functional Arm Activity Behavioural Observation System (FAABOS) [48]					+	Low (− 2)						
*Performance-reported outcomes (PerfOs)*												
10-Metre Walking Test (10MWT) [25, 65]					+	High			+	Low (− 2)		
6-Minute Walking test (6MWT) [25, 65]					+	Low (− 2)						
Community balance and mobility scale (CB&M) [25]					+	Low (− 2)			−	Low (− 2)		
High Level Mobility Assessment (HiMAT) [25]					+	High			+	High		
Timed Up and Go test (TUG) [65]					+	Low (− 2)						
*Patient-reported outcomes (PROs)*												
Brain injury community rehabilitation outcome scale (BICRO) [21]	+	High	+	High	+	High			+	High		
European Quality of life scale (EQ5D) [64]			+	Moderate (− 1)								
Mayo-Portland Adaptability Inventory (MPAI-4) [64]			+	High	+	High			+	High		
Medical Outcomes Study 36-Item Short Form Health Survey (SF-36) [64]			+	Low (− 2)					+	Low (− 2)		
Satisfaction With Life Scale (SWLS) [64]	+	Low (− 2)			+	Low (− 2)						
Sickness Impact profile (SIP) [21]	+	Low (− 2)	+	Low (− 2)	+	Low (− 2)			+	Low (− 2)	+	Low (− 2)
*Self-reported outcomes (SRO)*												
Rivermead mobility index (RMI) [25]									+	Low (− 2)		

*Blanks refer to Inconsistent (±) ratings and were not graded to the modified-GRADE approach

Overall ratings: sufficient (+), insufficient (−), inconsistent (±), or indeterminate (?)

Modified-GRADE approach: high, moderate (− 1), low (− 2), very low (− 3); in which − 1 level refers to serious; − 2 level refers to very serious; and -3 level refers to very serious risk of bias, inconsistency, indirectness and imprecision [31]

Acute rehabilitation phase refers to a duration of 24 h after stroke onset and for medically stable patients, lasts 5–7 days [83]; Sub-acute rehabilitation phase refers to a duration of 1 to 6 months where the functional recovery and long-term health status are more affected [84]; Chronic rehabilitation phase begins once the person is discharged home [83]. We did not include the recovery phase for TBI because it was not defined clearly in the literature

*Content validity* was reported for 57 measures, with overall rating of sufficient for 55 and insufficient for 2 of measures. Of the 57, 20 were rated as high, 10 as moderate and 27 as low quality. Content validity was not reported for most of the measures, given that there was no evidence was indicated.

*Internal consistency* was reported for 50 measures, with overall rating of sufficient for 48 and insufficient for 2 of measures. Of the 50, 13 were rated as high, 13 as moderate and 24 as low quality. Internal consistency was not reported for most of the measures, as no evidence was indicated.

*Reliability* was reported for 144 measures, with overall rating of sufficient for 132, insufficient for 11 and indeterminate for 1 of measures. Of the 144, 36 were rated as high, 39 as moderate and 69 as low quality.

*Measurement error* was reported for 13 measures, with overall rating of sufficient for 12 and indeterminate for 1 of measures. Of the 13, 3 were rated as high, 3 as moderate and 7 as low quality. Measurement error was not reported for most of the measures, given that there was no evidence was indicated.

*Construct validity* was reported for 148 measures, with overall rating of sufficient for 127 and insufficient for 21 of measures. Of the 148, 32 were rated as high, 39 as moderate and 77 as low quality.

*Responsiveness* was reported for 90 measures, with overall rating of sufficient for 76 and insufficient for 14 of measures. Of the 90, 19 were rated as high, 25 as moderate and 46 as low quality.

## Description of interpretability and feasibility

Fifty-seven (39%) of mobility measures (9 ClinROs, 29 PerfOs, 10 PROs, 8 SROs, a TechO) met the standards and criteria for interpretability and feasibility. For most of the measures, evaluating the distribution of scores in the study population, the availability of scores and change scores for relevant groups and the minimal important change or minimal important difference was limited. Information about floor and ceiling effects was limited and only reported in 7 (5%) of measures (Table 4 in Supplementary).

## Summary of evidence

Results identified several mobility measures that were rated as ‘sufficient’ for most measurement properties as well as interpretability and feasibility, including Rivermead Mobility Index (RMI), six-minute walking test (6MWT), ten-metre walking test (10MWT), Barthel Index (BI), Berg Balance Scale (BBS), Frenchay Activity Index (FAI) and Stroke Impact Scale (SIS) among individuals with stroke and RMI and 6MWT among individuals with TBI.

## Discussion

This umbrella review aimed to synthesize the measurement properties, the interpretability and the feasibility of mobility measures evaluated using clinician, patient and technology-derived information among individuals with ABI. Additionally, unified results from several reviews can provide a larger body of evidence and strengthen the recommendations based on these findings. In this review, 85% of 147 mobility measures among 35 systematic reviews were mapped mainly to the ICF component of Activity and Participation. This finding is consistent with previous studies that mapped the construct of mobility measures into the component of activity and participation [23, 26, 27, 46, 48, 56, 66, 67]. Also, our results showed that current mobility measures lack information on environmental factors. Identifying environmental factors a physical and societal levels, as a potential determinant that influences mobility, is crucial for maintaining independent mobility and fully integrates the patient's perspectives, experiences and needs into every phase of medical consultation, evaluation, treatment and follow-up. For example, participation of individuals with disabilities in society is dependent on the use of accessible designs to remove physical environmental barriers in public and private facilities [68]. Therefore, we recommend increasing the coverage of environmental factors when evaluating mobility, especially as evidence accumulates about how to tailor interventions to specific individual profiles [67].

Without published guidelines for umbrella reviews for measurement properties, we applied the COSMIN guidelines for systematic reviews of outcome measures to guide the methodology of this review [31]. This facilitated comparing the evidence supporting the measures' measurement properties across systematic reviews, identifying strengths and limitations of mobility measures and supporting the selection of outcome measures for a specific purpose. Our findings showed that the systematic reviews' methodological quality using the JBI critical appraisal tool was relatively low as 83% of systematic reviews did not apply COSMIN Risk of Bias checklist. The use of clear, unified criteria for the evaluation of measurement properties enable a reasonable comparison between the findings and are recommended for future systematic reviews.

Although content validity is considered the most important measurement property [31], only 11% of measures were evaluated as ‘adequate‘. High-quality content validity systematic reviews include studies with representative samples of target users who could attest to the relevance, comprehensiveness and comprehensibility of the measurement tool [31]. Future systematic reviews should report measures’ content validity, as the appropriate content as perceived by target users imperative to support the use of the measure in clinical care and research. None of the included systematic reviews reported cross-cultural validity, meaning it is unknown if the tool's content validity is maintained at a conceptual level across cultures and languages. Also, criterion validity was not reported in any study due to lack of a ‘gold standard’, according to the COSMIN definition [31]. Therefore, future systematic reviews should include cross-cultural validity and criterion validity when evidence is available according to COSMIN guidelines [31].

Results showed an acceptable overall "sufficient" rating for reliability, construct validity and responsiveness for 132 (90%), 127 (86%) and 76 (52%) of the measures, respectively; however, among these measures ≤ 25% of the methods for evaluating these properties were rated as 'high; quality of evidence. One reason was related to the sample size, as the majority of systematic reviews included studies with sample size either < 50 or unreported. Recruiting an adequate sample size to detect modest but important effect sizes is a challenge in the current state of training and funding in rehabilitation research [69]. The synthesis of the sample size used to evaluate the measurement properties of each measure in this review can be used to inform the sample size that is ideal for future evaluation of mobility measures.

Only 39% of mobility measures contained information on interpretability and feasibility. For each source of information, there are different reasons for lack of feasibility which should be reported in the future studies. For ClinRO/PerfO, feasibility is primarily expressed as the proportion of missing data for participants that cannot be assessed [66]. For PRO/SRO, whether participants required assistance is considered while evaluating feasibility [70]; and for TechO, the complexity of tracking motion while carrying out daily activities may influence feasibility [71]. Less information was provided in terms of scoring interpretability. Future studies should evaluate the minimal important difference or minimal important change, and floor and ceiling effects to help guide clinical interpretation.

Results identified several mobility measures that were rated as "sufficient" for most measurement properties as well as interpretability and feasibility. RMI and 6MWT have been used across the continuum of care; SIS and 10MWT were used in both sub-acute and chronic settings; and FAI, BI and BBS were used at both acute and chronic settings. These widely used measures, however, have limitations in certain contexts; for example, a patient with cognitive impairment or unable to change body position. Decisions for selection of a mobility measure need to consider applicability to all patients and clinical contexts [72].

Few reviews of mobility measures focused on TBI as compared to stroke. Many of the outcome measures that were developed for individuals with TBI are either related to injury severity (e.g. Glasgow Coma Scale) or reflect global outcome (e.g. Disability Rating Scale). Multidimensional tools reflecting complex ranges of factors affecting TBI outcomes may be required for assessment across the continuum of care depending on the level of recovery and context of practice and the need to evaluate community activities.

Terminologies for sources of information were used interchangeably with no distinctions if patients or clinicians reported on a domain in a measure. For example, in a systematic review of PRO measures for functional performance [21] in the lower limb, they did not distinguish between SROs and PROs. Distinction between different sources of information is important as, in addition to the items and scale, the respondent influences the interpretation of the scores. Thus, a common language for the sources of information needs to be standardized to facilitate the selection of measures ensuring that evaluations of change within and between patients can be compared. In this review, we used sources of information definitions published by Mayo et al. [19].

Moreover, to capture the quality of movement, technological measures are required. For example, accelerometry provides kinematic data that can provide an opportunity to extend the quality and accuracy of measurement, filling the gaps not covered by the ClinRO, PRO and SRO scales. However, we found variations in evaluation of measurement properties between the different technologies. Two systematic reviews [22, 45] incorporated technology measures, without a standardized evaluation of the measurement properties. Standardization of how TechO measurement properties are tested is needed to increase applicability of rapidly emerging technologies in research and clinical care.

### Study limitations

The main strength of this umbrella review is that we have independently applied COSMIN guidelines to synthesize measurement properties, interpretability and feasibility of ABI mobility measures. The main limitations included the following: (1) data on measurement properties relied on what was in the reviews and were not retrieved or evaluated from primary studies; (2) articles before the year 2000 were not included. This decision was based on the rationale that the recommendations for appropriate statistical methods and interpretation of the results changed over time; (3) articles with low methodological quality were not excluded, as this review intended to be a comprehensive review of measures of mobility among individuals with ABI; (4) according to the standards at the time of publication, many studies used different terms and statistical methods to examine measurement properties. Applying modern measurement standards often requires "translation" between the author's terminology and COSMIN terms; (5) systematic reviews of measures that only evaluated determinants were not included to limit the scope of this review. However, some measures included determinants of mobility as part of the content, and these are reported in this review; and (6) this review is still limited in capturing all mobility measures, as we only included systematic reviews reporting measurement properties and used systematic literature searches to enable an unbiased selection of the outcome measures. It is possible that we have missed tools that are used in clinical practice but have not been applied in research. Therefore, we missed studies that mapped mobility measures to the ICF without considering the measurement properties [12, 73–82]. Some of these domains may become important for a Core Outcome Set for mobility to standardize mobility measures among individuals with ABI.

## Conclusion

This study presented a comprehensive synthesis of existing evidence on the measurement properties, the interpretability and the feasibility of mobility measures from various sources of information (patients, clinicians, technology) using an umbrella review of published systematic reviews among individuals with ABI. We expect the results to be a resource for researchers and clinicians to assist them in selecting mobility outcome measure based on the evidence supporting their psychometric properties. RMI, BI, FAI, BBS, 6MWT, 10MWT and SIS had the strongest measurement properties and support for their interpretability and feasibility. However, each measure was limited in evaluating mobility comprehensively. Also, considering tools which comprehensively capture the degree of complexity and variety of deficits experienced by individuals surviving TBI was limited in this review. The included systematic reviews were limited in reporting measures’ content validity. Also, they were limited in evaluating the minimal important difference or minimal important change, and floor and ceiling effects. Reporting these properties are essential to help guide clinical interpretation and to support the use of the measure in clinical care and research. Finally, identifying the most critical domains for mobility based on the ICF is critical to guide the development of the Core Outcome Set among individuals with ABI.

## Supplementary Information

Below is the link to the electronic supplementary material.

Supplementary file1 (DOCX 26 kb)

Supplementary file2 (DOCX 35 kb)

Supplementary file3 (DOCX 25 kb)

Supplementary file4 (DOCX 41 kb)

Supplementary file5 (DOCX 70 kb)

Supplementary file6 (DOCX 166 kb)

Supplementary file7 (DOCX 73 kb)

Supplementary file8 (DOCX 19 kb)

Supplementary file9 (DOCX 43 kb)

## Data Availability

Data are available as Supplementary Information.

## Abbreviations

10MWT: Ten-metre walking test
6MWT: Six-minute walking test
ABI: Acquired brain injury
BBS: Berg balance scale
BI: Barthel index
ClinRO: Clinician-reported outcome
COSMIN: Consensus-based standards for the selection of health measurement instrument
FAI: Frenchay activities index
GRADE: Grading of recommendations assessment, development and evaluation
ICF: International classification, functioning, disability and health
JBI: Joanna Briggs institute
PerfO: Performance-reported outcome
PRISMA: Preferred reporting items for systematic reviews and meta-analyses
PRO: Patient-reported outcome
RMI: Rivermead mobility index
SI: Supplementary information
SIS: Stroke impact scale
SRO: Self-report outcome
TBI: Traumatic brain injury
TechO: Technology-reported outcome
