# Supplementary material for: Quality of mobility measures among individuals with acquired brain injury: an umbrella review
Source: Qual Life Res. 2022 Mar 11;31(9):2567–99. doi: 10.1007/s11136-022-03103-4 (PMC9356944; doi:10.1007/s11136-022-03103-4)
Supplement: Supplementary file 1 — Supplementary file1 (DOCX 26 kb) [file 11136_2022_3103_MOESM1_ESM.docx]

**Quality of Mobility Measures among Individuals with Acquired Brain Injury: An Umbrella Review**

Rehab Alhasani, MSc,^1,2,6^ Claudine Auger, PhD,^2,4,5^ Matheus de Paiva Azevedo, BSc,^1^ Sara Ahmed, PhD ^1-3^

**Author affiliations:**

1. School of Physical and Occupation Therapy, Faculty of Medicine, McGill University, Montreal, Canada
2. Centre de Recherche Interdisciplinaire en Réadaptation (CRIR), Montreal, Canada
3. Constance Lethbridge Rehabilitation Center, CIUSSS Centre Ouest de l’ile de Montreal, Montreal, Canada
4. School of Rehabilitation, Faculty of Medicine, University of Montreal, Montreal, Canada
5. Site Institut Universitaire sur la Réadaptation en Déficience Physique de Montréal (IURDPM), CIUSSS Centre-Sud-de-l’Ile-de-Montréal, Montréal, Canada
6. Department of Rehabilitation Sciences, College of Health and Rehabilitation Sciences, Princess Nourah bint Abdulrahman University, Riyadh, Saudi Arabia

**Corresponding author:** Sara Ahmed, PhD, School of Physical and Occupation Therapy, Faculty of Medicine, McGill University, 3655 Sir William-Osler, Montreal, QC, Canada H3G 1Y6. Tel.: 514-398-4400 ext 00531.E-mail: sara.ahmed@mcgill.ca

**Supplementary file 1: Ovid Medline search strategy**

| **#** | **Searches** | **Results** |
| --- | --- | --- |
| 1 | Brain Injuries/ or Acquired brain injury.mp. | 50721 |
| 2 | stroke.mp. or exp Stroke/ | 270322 |
| 3 | traumatic brain injury.mp. or exp Brain Injuries, Traumatic/ | 35483 |
| 4 | Brain Injuries/ or non-traumatic brain injury.mp. | 50222 |
| 5 | Brain Injuries/ or non traumatic brain injury.mp. | 50222 |
| 6 | 1 or 2 or 3 or 4 or 5 | 334129 |
| 7 | performance based outcome*.mp. | 74 |
| 8 | performance-based outcome.mp. | 38 |
| 9 | "Process Assessment (Health Care)"/ or process assessment*.mp. | 29528 |
| 10 | process measure*.mp. or "Outcome and Process Assessment (Health Care)"/ | 26774 |
| 11 | "Outcome Assessment (Health Care)"/ or clinician report outcome*.mp. | 63956 |
| 12 | clinician report measure*.mp. | 13 |
| 13 | "Surveys and Questionnaires"/ or Patient report measure*.mp. | 400844 |
| 14 | "Surveys and Questionnaires"/ or patient report measure*.mp. | 400844 |
| 15 | "Surveys and Questionnaires"/ or Self-report measure*.mp. | 407827 |
| 16 | "Outcome Assessment (Health Care)"/ or Self-report outcome*.mp. or "Surveys and Questionnaires"/ | 458479 |
| 17 | "Outcome Assessment (Health Care)"/ or technology based measure*.mp. | 63961 |
| 18 | technology-based measure*.mp. | 5 |
| 19 | "Outcome Assessment (Health Care)"/ or technology based outcome*.mp. | 63957 |
| 20 | Accelerometry/ or Acceleromet*.mp. or Monitoring, Ambulatory/ | 19895 |
| 21 | Monitoring, Ambulatory/ or Pedometer.mp. or Monitoring, Physiologic/ | 59907 |
| 22 | Monitoring, Ambulatory/ or Gyroscope wearable system.mp. or Biofeedback, Psychology/ | 14272 |
| 23 | Activity monitor.mp. | 1257 |
| 24 | "Outcome Assessment (Health Care)"/ or Physical science technique*.mp. | 63960 |
| 25 | Clinimetry.mp. | 9 |
| 26 | Observational technique*.mp. | 130 |
| 27 | "Surveys and Questionnaires"/ or Diaries.mp. | 407596 |
| 28 | "Surveys and Questionnaires"/ or Questionnaire*.mp. | 633534 |
| 29 | Physiological technique*.mp. | 258 |
| 30 | Actigraphy.mp. or Actigraphy/ | 4714 |
| 31 | Monitoring, Physiologic/ or Ambulatory activity monitor*.mp. | 51290 |
| 32 | Accelerometry/ or Monitoring, Physiologic/ or Activity monitor*.mp. or Monitoring, Ambulatory/ | 63895 |
| 33 | Patient Outcome Assessment/ or Patient outcome assessment*.mp. | 4033 |
| 34 | Patient Reported Outcome Measures/ or "Outcome Assessment (Health Care)"/ or "Surveys and Questionnaires"/ or Patient report outcome  measure*.mp. | 459542 |
| 35 | Telemedicine/ or telemonitoring.mp. or Monitoring, Ambulatory/ or Monitoring, Physiologic/ | 75213 |
| 36 | Telemedicine/ or tele-monitoring.mp. or Monitoring, Ambulatory/ | 24415 |
| 37 | Monitoring, Ambulatory/ or Home monitoring.mp. | 8746 |
| 38 | Digital monitoring.mp. | 45 |
| 39 | Monitoring, Ambulatory/ or Web based monitoring.mp. | 7460 |
| 40 | Web-based monitoring.mp. or Monitoring, Ambulatory/ | 7460 |
| 41 | Internet based monitoring.mp. | 29 |
| 42 | mobility.mp. | 146819 |
| 43 | mobilit*.mp. | 151687 |
| 44 | activity.mp. | 2551450 |
| 45 | gait.mp. or Gait/ | 51056 |
| 46 | walk*.mp. or Walking/ | 109205 |
| 47 | lower limb.mp. or Lower Extremity/ | 39175 |
| 48 | Lower limb activity.mp. or Lower Extremity/ | 14998 |
| 49 | Movement/ or sit to stand.mp. | 70042 |
| 50 | Climb* stair*.mp. | 761 |
| 51 | Wheelchair.mp. or Wheelchairs/ | 7195 |
| 52 | performance based measure*.mp. | 688 |
| 53 | Movement/ or movement.mp. | 365518 |
| 54 | 7 or 8 or 9 or 10 or 11 or 12 or 13 or 14 or 15 or 16 or 17 or 18 or 19 or 20 or 21 or 22 or 23 or 24 or 25 or 26 or 27 or 28 or 29 or 30 or 31 or 32 or 33  or 34 or 35 or 36 or 37 or 38 or 39 or 40 or 41 or 52 | 826617 |
| 55 | balance.mp. or POSTURAL BALANCE/ | 223878 |
| 56 | Walking/ or community ambulation.mp. | 28610 |
| 57 | 42 or 43 or 44 or 45 or 46 or 47 or 48 or 49 or 50 or 51 or 52 or 53 or 55 or 56 | 3276423 |
| 58 | STROKE REHABILITATION/ or rehabilitation.mp. or NEUROLOGICAL REHABILITATION/ or REHABILITATION/ | 286767 |
| 59 | instrumentation.mp. [mp=title, abstract, original title, name of substance word, subject heading word, floating sub-heading word, keyword heading  word, protocol supplementary concept word, rare disease supplementary concept word, unique identifier, synonyms] | 631636 |
| 60 | methods.mp. [mp=title, abstract, original title, name of substance word, subject heading word, floating sub-heading word, keyword heading word,  protocol supplementary concept word, rare disease supplementary concept word, unique identifier, synonyms] | 6625341 |
| 61 | Validation Studies.mp. [mp=title, abstract, original title, name of substance word, subject heading word, floating sub-heading word, keyword heading  word, protocol supplementary concept word, rare disease supplementary concept word, unique identifier, synonyms] | 96216 |
| 62 | Comparative Study.mp. [mp=title, abstract, original title, name of substance word, subject heading word, floating sub-heading word, keyword heading  word, protocol supplementary concept word, rare disease supplementary concept word, unique identifier, synonyms] | 1829711 |
| 63 | psychometrics.mp. [mp=title, abstract, original title, name of substance word, subject heading word, floating sub-heading word, keyword heading  word, protocol supplementary concept word, rare disease supplementary concept word, unique identifier, synonyms] | 68799 |
| 64 | (psychometr* or clinimetr* or clinometr*).mp. [mp=title, abstract, original title, name of substance word, subject heading word, floating sub-heading  word, keyword heading word, protocol supplementary concept word, rare disease supplementary concept word, unique identifier, synonyms] | 85815 |
| 65 | (outcome assessment health care or outcome assessment or outcome measure*).mp. [mp=title, abstract, original title, name of substance word,  subject heading word, floating sub-heading word, keyword heading word, protocol supplementary concept word, rare disease supplementary concept word, unique identifier, synonyms] | 259515 |
| 66 | (observer variation or observer variation or Health Status Indicators or reproducibility of results or reproducib* or discriminant analysis or reliab* or unreliab* or valid* or coefficient or homogeneity or homogeneous or internal consistency).mp. [mp=title, abstract, original title, name of substance word, subject heading word, floating sub-heading word, keyword heading word, protocol supplementary concept word, rare disease supplementary  concept word, unique identifier, synonyms] | 1540652 |
| 67 | (cronbach* alpha or alphas or item correlation* or selection* or reduction* or agreement or precision or imprecision or precise values or test-retest or test retest).mp. [mp=title, abstract, original title, name of substance word, subject heading word, floating sub-heading word, keyword heading word,  protocol supplementary concept word, rare disease supplementary concept word, unique identifier, synonyms] | 1938724 |
| 68 | (reliab* test or retest or stability or interrater or inter-rater or intrarater or intra-rater or intertester or inter-tester or intratester or intra-tester or interobserver or inter-observer or intraobserver or intraobserver or intertechnician or inter-technician or intratechnician or intra-technician).mp. [mp=title, abstract, original title, name of substance word, subject heading word, floating sub-heading word, keyword heading word, protocol  supplementary concept word, rare disease supplementary concept word, unique identifier, synonyms] | 461820 |
| 69 | (interexaminer or inter-examiner or intraexaminer or intra-examiner or interassay or inter-assay or intraassay or intra-assay or interindividual or inter- individual or intraindividual or intra-individual).mp. [mp=title, abstract, original title, name of substance word, subject heading word, floating sub-  heading word, keyword heading word, protocol supplementary concept word, rare disease supplementary concept word, unique identifier, synonyms] | 41687 |
| 70 | (interparticipant or inter-participant or intraparticipant or intra-participant or kappa or kappas or repeatab).mp. [mp=title, abstract, original title, name of substance word, subject heading word, floating sub-heading word, keyword heading word, protocol supplementary concept word, rare disease  supplementary concept word, unique identifier, synonyms] | 132266 |
| 71 | (replicab or repeated measure or measures or findings or result or results or test or tests or generaliza*).mp. [mp=title, abstract, original title, name of substance word, subject heading word, floating sub-heading word, keyword heading word, protocol supplementary concept word, rare disease  supplementary concept word, unique identifier, synonyms] | 10418234 |
| 72 | (generalisa* or concordance or intraclass correlation* or discriminative or known group or factor analysis or factor analyses).mp. [mp=title, abstract, original title, name of substance word, subject heading word, floating sub-heading word, keyword heading word, protocol supplementary concept  word, rare disease supplementary concept word, unique identifier, synonyms] | 127347 |
| 73 | (dimension* or subscale* or multitrait scaling analysis or analyses or item discriminant).mp. [mp=title, abstract, original title, name of substance word,  subject heading word, floating sub-heading word, keyword heading word, protocol supplementary concept word, rare disease supplementary concept word, unique identifier, synonyms] | 1308767 |
| 74 | (interscale correlation* or error or errors or individual variability or variability analysis or values or uncertainty measurement or measuring).mp. [mp=title, abstract, original title, name of substance word, subject heading word, floating sub-heading word, keyword heading word, protocol  supplementary concept word, rare disease supplementary concept word, unique identifier, synonyms] | 1622787 |
| 75 | (standard error of measurement or sensitiv* or responsive* or minimal or minimally or clinical).mp. [mp=title, abstract, original title, name of substance word, subject heading word, floating sub-heading word, keyword heading word, protocol supplementary concept word, rare disease  supplementary concept word, unique identifier, synonyms] | 5294004 |
| 76 | (clinically important or significant or detectable change or difference or small*real or detectable change).mp. [mp=title, abstract, original title, name of substance word, subject heading word, floating sub-heading word, keyword heading word, protocol supplementary concept word, rare disease  supplementary concept word, unique identifier, synonyms] | 3430257 |
| 77 | (difference or meaningful change or ceiling effect or floor effect or Item response model).mp. [mp=title, abstract, original title, name of substance  word, subject heading word, floating sub-heading word, keyword heading word, protocol supplementary concept word, rare disease supplementary concept word, unique identifier, synonyms] | 952375 |
| 78 | (IRT or Rasch or Differential item functioning or DIF or computer adaptive testing or item bank or cross-cultural equivalence).mp. [mp=title, abstract,  original title, name of substance word, subject heading word, floating sub-heading word, keyword heading word, protocol supplementary concept word, rare disease supplementary concept word, unique identifier, synonyms] | 8655 |
| 79 | 58 or 59 or 60 or 61 or 62 or 63 or 64 or 65 or 66 or 67 or 68 or 69 or 70 or 71 or 72 or 73 or 74 or 75 or 76 or 77 or 78 | 16827007 |
| 80 | (instrumentation or Validation Studiies or reproducibility of results or reproducib* or psychometrics).mp. [mp=title, abstract, original title, name of  substance word, subject heading word, floating sub-heading word, keyword heading word, protocol supplementary concept word, rare disease supplementary concept word, unique identifier, synonyms] | 1081784 |
| 81 | (psychometr* or clinimetr* or clinometr* or observer variation or observer variation or discriminant analysis).mp. [mp=title, abstract, original title, name of substance word, subject heading word, floating sub-heading word, keyword heading word, protocol supplementary concept word, rare  disease supplementary concept word, unique identifier, synonyms] | 143788 |
| 82 | (reliab* or valid* or coefficient or internal consistency or cronbach* alpha or alphas or item correlation or item correlations).mp. [mp=title, abstract, original title, name of substance word, subject heading word, floating sub-heading word, keyword heading word, protocol supplementary concept  word, rare disease supplementary concept word, unique identifier, synonyms] | 1106012 |
| 83 | (item selection or item selections or item reduction or item reductions or agreement or precision or imprecision).mp. [mp=title, abstract, original title,  name of substance word, subject heading word, floating sub-heading word, keyword heading word, protocol supplementary concept word, rare disease supplementary concept word, unique identifier, synonyms] | 359742 |
| 84 | (precise values or test-retest or test retest or reliabtest or retest or stability or interrater or inter-rater).mp. [mp=title, abstract, original title, name of substance word, subject heading word, floating sub-heading word, keyword heading word, protocol supplementary concept word, rare disease  supplementary concept word, unique identifier, synonyms] | 436899 |
| 85 | (intrarater or intra-rater or intertester or inter-tester or intratester or intra-tester or interobserver or inter-observer).mp. [mp=title, abstract, original title,  name of substance word, subject heading word, floating sub-heading word, keyword heading word, protocol supplementary concept word, rare disease supplementary concept word, unique identifier, synonyms] | 25824 |
| 86 | (intraobserver or intra-observer or intertechnician or intertechnician or intratechnician or intra-technician or interexaminer or inter-examiner or intraexaminer).mp. [mp=title, abstract, original title, name of substance word, subject heading word, floating sub-heading word, keyword heading  word, protocol supplementary concept word, rare disease supplementary concept word, unique identifier, synonyms] | 11004 |
| 87 | (intra-examiner or interassay or inter-assay or intraassay or intra-assay or interindividual or inter-individual or intraindividual or intra-individual).mp. [mp=title, abstract, original title, name of substance word, subject heading word, floating sub-heading word, keyword heading word, protocol  supplementary concept word, rare disease supplementary concept word, unique identifier, synonyms] | 40346 |
| 88 | (interparticipant or inter-participant or intraparticipant or intra-participant or kappa or kappas or coefficient of variation).mp. [mp=title, abstract, original title, name of substance word, subject heading word, floating sub-heading word, keyword heading word, protocol supplementary concept word, rare  disease supplementary concept word, unique identifier, synonyms] | 152787 |
| 89 | (repeatab* or replicab* or repeated measure or measures or findings or result or results or test or tests).mp. [mp=title, abstract, original title, name of  substance word, subject heading word, floating sub-heading word, keyword heading word, protocol supplementary concept word, rare disease supplementary concept word, unique identifier, synonyms] | 10414057 |
| 90 | (generaliza* or generalisa* or concordance or intraclass correlation* or discriminative or known group or factor analysis).mp. [mp=title, abstract, original title, name of substance word, subject heading word, floating sub-heading word, keyword heading word, protocol supplementary concept  word, rare disease supplementary concept word, unique identifier, synonyms] | 159663 |
| 91 | (factor analyses or factor structure or factor structures or dimensionality or subscale* or multitrait scaling analysis).mp. [mp=title, abstract, original title, name of substance word, subject heading word, floating sub-heading word, keyword heading word, protocol supplementary concept word, rare  disease supplementary concept word, unique identifier, synonyms] | 58747 |
| 92 | (multitrait scaling analyses or item discriminant or interscale correlation or interscale correlations).mp. [mp=title, abstract, original title, name of substance word, subject heading word, floating sub-heading word, keyword heading word, protocol supplementary concept word, rare disease  supplementary concept word, unique identifier, synonyms] | 282 |
| 93 | (error or errors measure* or correlat* or evaluat* or accuracy or accurate or precision or mean).mp. [mp=title, abstract, original title, name of substance word, subject heading word, floating sub-heading word, keyword heading word, protocol supplementary concept word, rare disease  supplementary concept word, unique identifier, synonyms] | 5943737 |
| 94 | (individual variability or interval variability or rate variability or variability analysis or uncertainty measurement or measuring).mp. [mp=title, abstract, original title, name of substance word, subject heading word, floating sub-heading word, keyword heading word, protocol supplementary concept  word, rare disease supplementary concept word, unique identifier, synonyms] | 274144 |
| 95 | (standard error of measurement or sensitiv* or responsive* or limit detection or minimal detectable concentration).mp. [mp=title, abstract, original  title, name of substance word, subject heading word, floating sub-heading word, keyword heading word, protocol supplementary concept word, rare disease supplementary concept word, unique identifier, synonyms] | 1698114 |
| 96 | (interpretab* or small* real or detectable change or difference).mp. [mp=title, abstract, original title, name of substance word, subject heading word, floating sub-heading word, keyword heading word, protocol supplementary concept word, rare disease supplementary concept word, unique  identifier, synonyms] | 959743 |
| 97 | (meaningful change or minimal important change or minimal important difference or minimally important change).mp. [mp=title, abstract, original title, name of substance word, subject heading word, floating sub-heading word, keyword heading word, protocol supplementary concept word, rare  disease supplementary concept word, unique identifier, synonyms] | 1215 |
| 98 | (minimally important difference or minimal detectable change or minimal detectable difference).mp. [mp=title, abstract, original title, name of substance word, subject heading word, floating sub-heading word, keyword heading word, protocol supplementary concept word, rare disease  supplementary concept word, unique identifier, synonyms] | 1171 |
| 99 | (minimally detectable change or minimally detectable difference or minimal real change).mp. [mp=title, abstract, original title, name of substance word, subject heading word, floating sub-heading word, keyword heading word, protocol supplementary concept word, rare disease supplementary  concept word, unique identifier, synonyms] | 23 |
| 100 | (minimal real difference or minimally real change or minimally real difference).mp. [mp=title, abstract, original title, name of substance word, subject heading word, floating sub-heading word, keyword heading word, protocol supplementary concept word, rare disease supplementary concept word,  unique identifier, synonyms] | 4 |
| 101 | (ceiling effect or floor effect or Item response model or IRT or Rasch).mp. [mp=title, abstract, original title, name of substance word, subject heading  word, floating sub-heading word, keyword heading word, protocol supplementary concept word, rare disease supplementary concept word, unique identifier, synonyms] | 7840 |
| 102 | (Differential item functioning or DIF or computer adaptive testing or item bank or cross-cultural equivalence).mp. [mp=title, abstract, original title, name of substance word, subject heading word, floating sub-heading word, keyword heading word, protocol supplementary concept word, rare  disease supplementary concept word, unique identifier, synonyms] | 3589 |
| 103 | 80 or 81 or 82 or 83 or 84 or 85 or 86 or 87 or 88 or 89 or 90 or 91 or 92 or 93 or 94 or 95 or 96 or 97 or 98 or 99 or 100 or 101 or 102 | 13729406 |
| 104 | 79 or 103 | 17444095 |
| 105 | 6 and 54 and 57 and 104 | 3139 |
| 106 | limit 105 to (meta analysis or "review" or systematic reviews) | 1290 |
