# Supplementary material for: Quality of mobility measures among individuals with acquired brain injury: an umbrella review
Source: Qual Life Res. 2022 Mar 11;31(9):2567–99. doi: 10.1007/s11136-022-03103-4 (PMC9356944; doi:10.1007/s11136-022-03103-4)
Supplement: Supplementary file 2 — Supplementary file2 (DOCX 35 kb) [file 11136_2022_3103_MOESM2_ESM.docx]

**Quality of Mobility Measures among Individuals with Acquired Brain Injury: An Umbrella Review**

Rehab Alhasani, MSc,^1,2,6^ Claudine Auger, PhD,^2,4,5^ Matheus de Paiva Azevedo, BSc,^1^ Sara Ahmed, PhD ^1-3^

**Author affiliations:**

1. School of Physical and Occupation Therapy, Faculty of Medicine, McGill University, Montreal, Canada
2. Centre de Recherche Interdisciplinaire en Réadaptation (CRIR), Montreal, Canada
3. Constance Lethbridge Rehabilitation Center, CIUSSS Centre Ouest de l’ile de Montreal, Montreal, Canada
4. School of Rehabilitation, Faculty of Medicine, University of Montreal, Montreal, Canada
5. Site Institut Universitaire sur la Réadaptation en Déficience Physique de Montréal (IURDPM), CIUSSS Centre-Sud-de-l’Ile-de-Montréal, Montréal, Canada
6. Department of Rehabilitation Sciences, College of Health and Rehabilitation Sciences, Princess Nourah bint Abdulrahman University, Riyadh, Saudi Arabia

**Corresponding author:** Sara Ahmed, PhD, School of Physical and Occupation Therapy, Faculty of Medicine, McGill University, 3655 Sir William-Osler, Montreal, QC, Canada H3G 1Y6. Tel.: 514-398-4400 ext 00531.E-mail: sara.ahmed@mcgill.ca

**Supplementary file 2: The characteristics of the included systematic reviews**

| **Author (year)** | **Country** | **Objective or research question** | **Population** | **SOI** | **Search strategy data source/date range of included studies** | **Number of sub-studies** | **Method for conducting the systematic review** | **Number of identified measures** | **Critical appraisal** | **Apply the ICF framework** | **Recommended Outcome measures** |
| --- | --- | --- | --- | --- | --- | --- | --- | --- | --- | --- | --- |
| Ashford (2008)[1] | UK | To identify valid and reliable outcome measures that have been applied to assess changes following focal rehabilitation interventions in the hemiparetic upper limb in the context of stroke or brain injury, and are reflective of “real-life” function, for both active and passive tasks. | Stroke & TBI | PRO  SRO | Medline, CINAHL, BIDS Science Citation Index, EMBASE, Specialized Register of Stroke Trials, National Health Service National Research, MRC Clinical Trials Directory, the Cochrane Database of Systematic Reviews, Database of Abstracts of Reviews of Effects (DARE), Google, ProFusion and SIGLE (medical/rehabilitation grey literature). Other sources were reference lists from papers identified, conference proceedings, books and book chapters and communication with lead authors of published studies and other researchers/Inception to May 2008 | 84 | NR | 5 | No | No | ABILIHAND |
| Ashford (2015)[2] | UK | To identify valid and reliable patient (and/or carer) reported outcome measures that have been applied to assess changes following focal rehabilitation.  Interventions in the lower limb in the context of stroke or brain injury, and are reflective of ‘real-life’ function (activity according to the ICF for both active and passive function tasks. | **S**troke & TBI | PRO  SRO | Ovid MEDLINE, CINAHL, Embase, Web of Science, Pubmed, National Health Service National Research Register, MRC Clinical Trial directory, Database of Abstracts of Reviews of effect (DARE), Google Scholar, Cochrane Database of Systematic Reviews/NR | 22 | PRISMA | 7 | Yes /COSMIN | Yes | Rivermead Mobility Index |
| Baker (2011)[3] | UK | To present a scale selection strategy for evidence-based scale selection in stroke research. | Stroke | ClinRO  PerfO  PRO  SRO | Medline, Embase (Excerpta Medica), CINAHL, and PsycINFO/from 1966 to 2011 | NR | NR | 5 | No | Yes | Chedoke Arm and Hand Inventory Stroke Rehabilitation Assessment of Movement upper limb section ABILHAND |
| Barak (2006)[4] | USA | To provide a comprehensive overview of the issues in selecting stroke outcome measures and to characterize existing measures relative to these issues. | Stroke | ClinRO  PerfO  PRO  SRO | NR/NR | NR | NR | 27 | No | Yes | NR |
| Connel (2012)[5] | UK | To review the psychometric properties and clinical utility of upper limb impairment and activity in people with neurologic conditions. | Stroke & TBI | ClinRO  PerfO  PRO  SRO | MEDLINE, CINAHL, EMBASE, PEDro, AMED/ inception to September 2009 | NR | Standardized accepted guidelines from previous published | 10 | No | Yes | Box and Block test  Action Research  Arm test |
| Croarkin (2004)[6] | USA | To review all available literature relative to upper extremity motor function tests used for people during rehabilitation following a stroke; to develop and use criteria to select tests and relevant literature; to rate tests relative to available psychometric evidence that supports the use of upper extremity motor function testing following a stroke. | Stroke | ClinRO  PerfO | Pubmed, CINAHL/from 1983 to December 1999 | 13 | NR | 9 | No | No | Box and Block Test  Jebsen Hand Function Test |
| Fini (2015)[7] | Australia | To describe how physical activity is monitored following stroke, including identification of methods and devices used across the stroke pathway (from acute care to community). The secondary aim was to describe the reported psychometric properties (reliability and validity) of these measurements. | Stroke | TechO | MEDLINE, CINAHL, EMBASE, Cochrane Library, PEDro/Inception to February 2014 | 91 | NR | 14 | No | No | NR |
| Gebruers (2010)[8] | Belgium | To assess the clinometric properties and clinical applicability of different accelerometer-based measurement techniques in persons with stroke. | Stroke | TechO | PubMed, EMBASE, CINAHL, Cochrane Library of Clinical Trials/Inception to September 2008 | 25 | NR | 14 | No | No | NR |
| Geroin (2013)[9] | Italy | To identify appropriate selection criteria of clinical scales for future trials, starting from those most commonly used in the literature, according to their psychometric properties and ICF domains | Stroke | ClinRO  PerfO  PRO  SRO | MEDLINE, EMBASE, CINALH, Pub-Med, PsychINFO and Scopus databases/ from January 2000 to January 2012 | 27 | Standardized accepted guidelines from previous published | 6 | No | Yes | 10-meter walking test  Motricity index  6-meter walking test  Berg Balance Scale  Rivermead Mobility Index  Functional Ambulation Category |
| Gor-Garcı´a-Fogeda (2014)[10] | Spain | To compile all scales available in the scientific literature that assess gross motor function in stroke; to establish which specific aspects of gross motor function they assess; to study their psychometric properties. | Stroke | ClinRO  PerfO  PRO  SRO | MEDLINE, PEDro, ISI Web of Knowledge, and Cumulative Index to Nursing and Allied Health (CINAHL)/ from March 2011 to January 2014 | 19 | NR | 7 | No | No | Stroke rehabilitation assessment of movement  Fugl-Meyer Assessment |
| Hong (2016)[11] | USA | To provide the psychometric properties of upper extremity outcome measures validated by the Rasch model and assess the extent to which their measurement areas cover the domains of the International Classification of Functioning, Disability and Health model. | Stroke & neurological conditions | ClinRO  PerfO  PRO  SRO | PubMed, CINAHL, Scopus, PsycINFO, Ovid/MEDLINE, ERIC, and Cochrane library/ from January 1966 to  March 2014 | 22 | NR | 15 | No | Yes | NR |
| [Lemmens (2012)](#RANGE!_ENREF_4)[12] | Netherland | To identify and evaluate the available instruments to assess arm-hand skilled performance in patients with stroke; to categorize the available instruments into the category’s capacity, perceived performance, and actual performance. Instruments for which no data about the validity and reliability was available were not included in this study. | Stroke | ClinRO  PerfO | PubMed, CINAHL, EMBASE, Cochrane, PsychINFO, IEEE and Scopus/Inception to November 2010 | 747 | NR | 21 | No | Yes | NR |
| Martins (2019)[13] | Brazil | To summarize both the measurement properties and clinical utility of self-report measures of physical activity levels of subjects with stroke and to evaluate both the methodological quality of the studies on measurement properties and the quality of the measurement properties. | Stroke | PRO  SRO | MEDLINE, EMBASE, PEDro, LILACS, and SCIELO/Inception to December 2018 | 19 | PRISMA | 6 | Yes /COSMIN | No | NR |
| Oczkowski (2010)[14] | Canada | To identify all studies that evaluated the reliability of proxy respondents for patients with stroke. | Stroke | PRO  SRO | MEDLINE, Google, and the Cochrane Library/ from 1969 to June 2008 | 14 | NR | 8 | No | No | NR |
| Pearson (2004)[15] | UK | To review some current methods of assessing mobility in terms of reliability, validity, responsiveness and whether they can inform objectively on performance. | Stroke & neurological conditions | ClinRO  PerfO  PRO  SRO  TechO | NR/NR | NR | NR | 5 | No | Yes | NR |
| Pollock (2011)[16] | Canada | To identify and evaluate walking balance measures that have been established for use with people post stroke at the level of community walking. Outcome measures will be evaluated for content validity in the context of current physiotherapy practice. Construct validity, reliability and aspects of clinical interpretability will be explored with recommendations for clinical use of the outcome measure. | Stroke | ClinRO  PerfO | MEDLINE, Embase, AMED/Inception to April 2010 | 24 | NR | 9 | No | No | NR |
| Rowland (2008)[17] | Australia | To review the literature of upper limb ability assessments following stroke; to analyze the assessments against criteria that will assist clinicians to determine the utility of the tools; to rate the assessment’s psychometric properties. | Stroke | ClinRO  PerfO | MEDLINE, CINAHL, Cochrane Library/ from 1965 to  2008 | NR | NR | 7 | No | No | NR |
| Salbach (2017)[18] | Canada | To appraise and synthesize the research literature describing: reliability, measurement error, construct validity, and sensitivity to change; the effect of walk test protocol elements on test performance for time-limited walk tests in adult’s post stroke; to identify gaps in the evaluation of measurement properties of time-limited walk tests; to identify considerations for the administration and interpretation of performance on time-limited walk tests post-stroke to enhance acceptance, utility, and value for practicing clinicians. | Stroke | ClinRO  PerfO | MEDLINE, EMBASE, PubMed, CINAHL, Scopus, PEDro, Cochrane Library/Inception to July 2013 | 43 | PRISMA | 5 | Yes/COSMIN | Yes | 6-minute walking test |
| Salter (2005)[19] | Canada | To evaluate the psychometric and administrative properties of outcome measures assigned to the ICF Body Functions category, and commonly used in stroke rehabilitation research. | Stroke | ClinRO  PerfO  PRO  SRO | NR/NR | NR | NR | 5 | No | Yes | NR |
| Salter (2005)[20] | Canada | To evaluate the psychometric and administrative properties of outcome measures in the ICF Participation category, which are used in stroke rehabilitation research and reported in the published literature. | Stroke | PRO  SRO | NR/NR | NR | NR | 6 | No | Yes | NR |
| Salter (2005)[21] | Canada | To evaluate the psychometric and administrative properties of outcome measures in the ICF Activity category used in stroke rehabilitation research and reported in the published literature. | Stroke | ClinRO  PerfO  PRO  SRO | NR/NR | NR | NR | 9 | No | Yes | NR |
| Scrivener (2013)[22] | Australia | How responsive are measurement tools that measure any aspect of lower limb physical performance in stroke survivors when the use of the measure commences in inpatient care that is early after stroke. | Stroke | ClinRO  PerfO  PRO  SRO  TechO | Medline, CINAHL and EMBASE and CINAHL/Inception to April 2012 | 21 | PRISMA | 19 | Yes /COSMIN | No | Berg Balance Scale 5-metre walk test 2, 6- and 12-meter walking tests  Functional Ambulation  Category Rivermead Mobility  Index |
| [Silva (2014)](#RANGE!_ENREF_5) [23] | Brazil | To determine the measurement properties and feasibility previously investigated for clinical tests that evaluate sit-to-stand and stand-to-sit in subjects with neurological disease. | Stroke | ClinRO  PerfO | MEDLINE, SCIELO, LILACS and PEDro/Inception to December 2012 | 11 | PRISMA | 1 | Yes/COSMIN | No | Five times sit to stand test |
| Simpson (2013)[24] | Canada | To synthesize and critically review the research evidence that captures responsiveness as defined by three types of change (observed, important, and detectable). This systematic review provides an understanding of the responsiveness of outcome measures used in stroke research, specifically within the context of upper extremity functional recovery. | Stroke | ClinRO  PerfO  PRO  SRO | MEDLINE, EMBASE, CINAHL, PsycINFO, Cochrance CENTRAL/Inception to March 2012 | 68 | NR | 4 | No | No | Action Research Arm Test  Motor Activity Log Wolf Motor Function test  Stroke Impact Scale |
| Sivan (2011)[25] | UK | To identify the outcome measures and classify them using the ICF and report on their psychometric properties. | Stroke | ClinRO  PerfO  PRO  SRO | MEDLINE, EMBASE, CINAHL, PubMed, PsychINFO/NR | 28 | NR | 20 | No | Yes | Fugl-Mayer  kinematic measures Action  Action Research Arm Test  Wolf Motor Function Test Functional independence measure  ABILHAND |
| [Sorrentino (2018)](#RANGE!_ENREF_2)[26] | Italy | To conduct an updated systematic literature review in order to identify psychometrically sound clinical measurement scales for assessing trunk control in stroke and to analyze the strength of their psychometric characteristics to obtain accurate and meaningful indicators of the treatment outcome, thus improving decision-making in clinical practice. | Stroke | ClinRO  PerfO | NR/January 2006 to April 2017 | 19 | PRISMA | 10 | No | No | NR |
| Stevens (2010)[27] | USA | To enable the orthotics and prosthetics practitioners to better use established timed ambulatory outcome measures. | Stroke, TBI and neurological conditions | ClinRO  PerfO | PubMed/Inception to April 2009 | NR | NR | 4 | No | No | NR |
| Teale (2010)[28] | UK | To identify outcome measures that has been demonstrated as valid and reliable for postal administration and acceptable to patients with stroke and their careers across a spectrum of domains and impairments. | Stroke | PRO  SRO | Cochrane Controlled Trials Register, MEDLINE, EMBASE, CINAHL psychINFO, AMED and British Nursing Index/Inception to January 2009 | 60 | NR | 5 | No | No | Frenchay Activities Index  Subjective Index of Physical and Social Outcome  European Quality of life test |
| Tse (2013)[29] | Australia | To identify and critique the tools frequently used to measure participation in clinical stroke studies and to identify the ICF Activities and Participation domains sampled within these most frequently used participation measures. | Stroke | PRO  SRO | Medline, CINAHL, ProQuest Central Database/from January 2001 to April 2012 | 119 | NR | 5 | No | Yes | Stroke Impact Scale  London Handicap Scale  Assessment of Life Habits  Frenchay Activities Index Activity Card Sort |
| Tyson (2009)[30] | UK | To identify and recommend the best measures to use with neurological and stroke patients in the clinical setting. | Stroke, TBI and neurological conditions | ClinRO  PerfO  PRO  SRO  TechO | MEDLINE, CINAHL, EMBASE, PEDro, and AMED/Inception to October 2008 | NR | Standardized accepted guidelines from previous published | 15 | No | Yes | 5-meter walking test  10-meter walking test  6-minute walk test  High Level Mobility Assessment Tool Rivermead Mobility Index |
| Van Bloemendaal (2012)[31] | Netherland | To provide an overview of walking tests used in stroke survivors including information about the tests’ measurement properties in terms of reliability, validity, and responsiveness. | Stroke | ClinRO  PerfO TechO | PubMed, CINAHL, EMBASE and Cochrane Controlled Trial Register/from 1966 to January 2011 | 32 | NR | 15 | Yes /COSMIN | Yes | NR |
| Van Peppen (2007)[32] | Netherland | To describe the available evidence to guide the clinical decision-making process of physiotherapists dealing with the rehabilitation of patients with stroke regarding: the best determinants of the ultimate functional recovery of patients diagnosed with stroke; the effectiveness of applicable physiotherapy interventions; and the core set of reliable, valid, and responsive outcome measures to assess patients’ progress in functional health at fixed moments after stroke. | Stroke | ClinRO  PerfO  PRO  SRO | MEDLINE, CINAHL and EMBASE databases, Cochrane Central Register of Controlled Trials, Cochrane Database of Systematic Reviews, DocOnline (Database of the Dutch Institute of Allied Health Care)/Inception to January 2004 | 32 | NR | 7 | No | Yes | Motricity index  trunk control test  Berg Balance scale Functional Ambulation Category  10-meter walking test  Frenchay Arm test  Barthel Index |
| Velstra (2011)[33] | Swizerland | To identify outcome measures that address functioning and disability in studies that involve persons with impairments in upper extremity function; to compare the content of the identified outcome measures with the ICF as a reference; to report the reliability and responsiveness data of the identified outcome measures when these data are available. | Stroke & neurological conditions | ClinRO  PerfO  PRO  SRO | MEDLINE, CINAHL, PsycINFO, and EMBASE/from July 1997 to July 2010 | 44 | NR | 13 | No | Yes | Modified Ashworth Scale Action Research Arm Test Motor Activity Log Functional Independence Measure Fugl-Meyer Assessment Short Form 36 Health Survey  Questionnaire |
| Verheyden (2007)[34] | Belgium | To give a systematic review of clinical tools designed to evaluate trunk performance after stroke. | Stroke | ClinRO  PerfO | CINAHL, Cochrane, Pedro, and PubMed/Inception to January 2006 | 32 | NR | 3 | No | No | Trunk control test Trunk impairment scales |
| [Wilde (2010)](#RANGE!_ENREF_6) [35] | USA | To summarize the selection of outcome measures by the interchangeably TBI outcomes workgroup to address primary clinical research objectives including documentation of the natural course of recovery from TBI, prediction of later outcome, measurement of treatment effects, and comparison of outcomes across studies. | TBI | ClinRO  PerfO  PRO  SRO | NR/NR | NR | NR | 6 | No | No | NR |

_ClinRO: Clinician-reported outcome, COSMIN: COnsensus-based Standards for the Selection of Health Measurement Instrument, ICF: International Classification of Functioning, Health and Disability framework, PerfO: Performance-reported outcome, PRO: Patient-reported outcome, PRISMA: Preferred Reporting Items for Systematic reviews and Meta-Analyse, SRO: Self-reported outcome, TechO: Technology-reported outcome; TBI: Traumatic Brain Injury, NR: not reported, SOI: source of information._

**References**

1. Ashford, S., Slade, M., Malaprade, F., & Turner-Stokes, L. (2008). Evaluation of functional outcome measures for the hemiparetic upper limb: a systematic review. *Journal of rehabilitation medicine, 40*(10), 787-795.
2. Ashford, S., Brown, S., & Turner-Stokes, L. (2015). Systematic review of patient-reported outcome measures for functional performance in the lower limb. *Journal of rehabilitation medicine, 47*(1), 9-17.
3. Baker, K., Cano, S. J., & Playford, E. D. (2011). Outcome measurement in stroke: a scale selection strategy. *Stroke, 42*(6), 1787-1794.
4. Barak, S., & Duncan, P. W. (2006). Issues in selecting outcome measures to assess functional recovery after stroke. *NeuroRx, 3*(4), 505-524.
5. Connell, L. A., & Tyson, S. F. (2012). Clinical reality of measuring upper-limb ability in neurologic conditions: a systematic review. *Archives of physical medicine and rehabilitation, 93*(2), 221-228.
6. Croarkin, E., Danoff, J., & Barnes, C. (2004). Evidence-based rating of upper-extremity motor function tests used for people following a stroke. *Physical therapy, 84*(1), 62-74.
7. Fini, N. A., Holland, A. E., Keating, J., Simek, J., & Bernhardt, J. (2015). How is physical activity monitored in people following stroke? *Disability and Rehabilitation, 37*(19), 1717-1731.
8. Gebruers, N., Vanroy, C., Truijen, S., Engelborghs, S., & De Deyn, P. P. (2010). Monitoring of physical activity after stroke: a systematic review of accelerometry-based measures. *Archives of physical medicine and rehabilitation, 91*(2), 288-297.
9. Geroin, C., Mazzoleni, S., Smania, N., Gandolfi, M., Bonaiuti, D., Gasperini, G., et al. (2013). Systematic review of outcome measures of walking training using electromechanical and robotic devices in patients with stroke. *Journal of rehabilitation medicine, 45*(10), 987-996.
10. Gor-García-Fogeda, M. D., Molina-Rueda, F., Cuesta-Gómez, A., Carratalá-Tejada, M., Alguacil-Diego, I. M., & Miangolarra-Page, J. C. (2014). Scales to assess gross motor function in stroke patients: a systematic review. *Archives of physical medicine and rehabilitation, 95*(6), 1174-1183.
11. Hong, I., & Bonilha, H. S. (2017). Psychometric properties of upper extremity outcome measures validated by Rasch analysis: a systematic review. *International Journal of Rehabilitation Research, 40*(1), 1-10.
12. Lemmens, R. J., Timmermans, A. A., Janssen-Potten, Y. J., Smeets, R. J., & Seelen, H. A. (2012). Valid and reliable instruments for arm-hand assessment at ICF activity level in persons with hemiplegia: a systematic review. *BMC neurology, 12*(1), 21.
13. Martins, J. C., Aguiar, L. T., Nadeau, S., Scianni, A. A., Teixeira-Salmela, L. F., & Faria, C. D. C. D. M. (2019). Measurement properties of self-report physical activity assessment tools for patients with stroke: a systematic review. *Brazilian journal of physical therapy, 23*(6), 476-490.
14. Oczkowski, C., & O'Donnell, M. (2010). Reliability of proxy respondents for patients with stroke: a systematic review. *Journal of Stroke and Cerebrovascular Diseases, 19*(5), 410-416.
15. Pearson, O. R., Busse, M., Van Deursen, R. W. M., & Wiles, C. M. (2004). Quantification of walking mobility in neurological disorders. *Qjm, 97*(8), 463-475.
16. Pollock, C., Eng, J., & Garland, S. (2011). Clinical measurement of walking balance in people post stroke: a systematic review. *Clinical rehabilitation, 25*(8), 693-708.
17. Rowland, T. J., & Gustafsson, L. (2008). Assessments of upper limb ability following stroke: a review. *British Journal of Occupational Therapy, 71*(10), 427-437.
18. Salbach, N. M., O'brien, K. K., Brooks, D., Irvin, E., Martino, R., Takhar, P., et al. (2017). Considerations for the selection of time-limited walk tests poststroke: a systematic review of test protocols and measurement properties. *Journal of Neurologic Physical Therapy, 41*(1), 3-17.
19. Salter, K., Jutai, J., Teasell, R., Foley, N., & Bitensky, J. (2005). Issues for selection of outcome measures in stroke rehabilitation: ICF Body Functions. *Disability and Rehabilitation, 27*(4), 191-207.
20. Salter, K., Jutai, J., Teasell, R., Foley, N., Bitensky, J., & Bayley, M. (2005). Issues for selection of outcome measures in stroke rehabilitation: ICF Participation. *Disability and Rehabilitation, 27*(9), 507-528.
21. Salter, K., Jutai, J., Teasell, R., Foley, N., Bitensky, J., & Bayley, M. (2005). Issues for selection of outcome measures in stroke rehabilitation: ICF activity. *Disability and Rehabilitation, 27*(6), 315-340.
22. Scrivener, K., Sherrington, C., & Schurr, K. (2013). A systematic review of the responsiveness of lower limb physical performance measures in inpatient care after stroke. *BMC neurology, 13*(1), 4.
23. Silva, P. F., Quintino, L. F., Franco, J., & Faria, C. D. (2014). Measurement properties and feasibility of clinical tests to assess sit-to-stand/stand-to-sit tasks in subjects with neurological disease: a systematic review. *Brazilian journal of physical therapy, 18*(2), 99-110.
24. Simpson, L. A., & Eng, J. J. (2013). Functional recovery following stroke: capturing changes in upper-extremity function. *Neurorehabilitation and neural repair, 27*(3), 240-250.
25. Sivan, M., O'Connor, R. J., Makower, S., Levesley, M., & Bhakta, B. (2011). Systematic review of outcome measures used in the evaluation of robot-assisted upper limb exercise in stroke. *Journal of Rehabilitation Medicine, 43*(3), 181-189.
26. Sorrentino G., S. P., Solaro C., Rabini A., Cerri C., Ferriero G. (2018). Clinical measurement tools to assess trunk performance after stroke: a systematic review. *European journal of physical and rehabilitation medicine*.
27. Stevens, P. M. (2010). Clinimetric properties of timed walking events among patient populations commonly encountered in orthotic and prosthetic rehabilitation. *JPO: Journal of Prosthetics and Orthotics, 22*(1), 62-74.
28. Teale, E. A., & Young, J. B. (2010). A review of stroke outcome measures valid and reliable for administration by postal survey. *Reviews in Clinical Gerontology, 20*(4), 338-353.
29. Tse, T., Douglas, J., Lentin, P., & Carey, L. (2013). Measuring participation after stroke: a review of frequently used tools. *Archives of physical medicine and rehabilitation, 94*(1), 177-192.
30. Tyson, S., & Connell, L. (2009). The psychometric properties and clinical utility of measures of walking and mobility in neurological conditions: a systematic review. *Clinical rehabilitation, 23*(11), 1018-1033.
31. van Bloemendaal, M., van de Water, A. T., & van de Port, I. G. (2012). Walking tests for stroke survivors: a systematic review of their measurement properties. *Disability and Rehabilitation, 34*(26), 2207-2221.
32. Van Peppen, R. P., Hendriks, H., Van Meeteren, N. L., Helders, P. J., & Kwakkel, G. (2007). The development of a clinical practice stroke guideline for physiotherapists in The Netherlands: a systematic review of available evidence. *Disability and Rehabilitation, 29*(10), 767-783.
33. Velstra, I.-M., Ballert, C. S., & Cieza, A. (2011). A systematic literature review of outcome measures for upper extremity function using the international classification of functioning, disability, and health as reference. *PM&R, 3*(9), 846-860.
34. Verheyden, G., Nieuwboer, A., Van de Winckel, A., & De Weerdt, W. (2007). Clinical tools to measure trunk performance after stroke: a systematic review of the literature. *Clinical rehabilitation, 21*(5), 387-394.
35. Wilde, E. A., Whiteneck, G. G., Bogner, J., Bushnik, T., Cifu, D. X., Dikmen, S., et al. (2010). Recommendations for the use of common outcome measures in traumatic brain injury research. *Archives of physical medicine and rehabilitation, 91*(11), 1650-1660. e1617.
