# Supplementary material for: Quality of mobility measures among individuals with acquired brain injury: an umbrella review
Source: Qual Life Res. 2022 Mar 11;31(9):2567–99. doi: 10.1007/s11136-022-03103-4 (PMC9356944; doi:10.1007/s11136-022-03103-4)
Supplement: Supplementary file 3 — Supplementary file3 (DOCX 25 kb) [file 11136_2022_3103_MOESM3_ESM.docx]

**Quality of Mobility Measures among Individuals with Acquired Brain Injury: An Umbrella Review**

Rehab Alhasani, MSc,^1,2,6^ Claudine Auger, PhD,^2,4,5^ Matheus de Paiva Azevedo, BSc,^1^ Sara Ahmed, PhD ^1-3^

**Author affiliations:**

1. School of Physical and Occupation Therapy, Faculty of Medicine, McGill University, Montreal, Canada
2. Centre de Recherche Interdisciplinaire en Réadaptation (CRIR), Montreal, Canada
3. Constance Lethbridge Rehabilitation Center, CIUSSS Centre Ouest de l’ile de Montreal, Montreal, Canada
4. School of Rehabilitation, Faculty of Medicine, University of Montreal, Montreal, Canada
5. Site Institut Universitaire sur la Réadaptation en Déficience Physique de Montréal (IURDPM), CIUSSS Centre-Sud-de-l’Ile-de-Montréal, Montréal, Canada
6. Department of Rehabilitation Sciences, College of Health and Rehabilitation Sciences, Princess Nourah bint Abdulrahman University, Riyadh, Saudi Arabia

**Corresponding author:** Sara Ahmed, PhD, School of Physical and Occupation Therapy, Faculty of Medicine, McGill University, 3655 Sir William-Osler, Montreal, QC, Canada H3G 1Y6. Tel.: 514-398-4400 ext 00531.E-mail: sara.ahmed@mcgill.ca

**Supplementary file 3: Methodological quality of the included systematic reviews using the Joanna Briggs Institute (JBI) critical appraisal checklist for Systematic Reviews and Research Syntheses**

| **JBI items** | Ashford (2008)[1] | Ashford (2015)[2] | Baker (2011)[3] | Barak (2006)[4] | Connel (2012)[5] | Croarkin ( 2004)[6] | Fini (2015)[7] | Gebruers (2010)[8] | Geroin (2013)[9] | Gor-Garcı´a-Fogeda (2014)[10] | Hong (2016)[11] | [Lemmens (2012)](#RANGE!_ENREF_4)[12] |
| --- | --- | --- | --- | --- | --- | --- | --- | --- | --- | --- | --- | --- |
| 1. Is the review question clearly and explicitly stated? | √ | √ | √ | √ | √ | √ | √ | √ | √ | √ | √ | √ |
| 2. Were the inclusion criteria appropriate for the review question? | √ | √ | √ | √ | √ | √ | √ | √ | √ | √ | √ | √ |
| 3. Was the search strategy appropriate? | √ | √ | √ | - | √ | √ | √ | √ | √ | √ | √ | √ |
| 4. Were the sources of studies adequate? | √ | √ | √ | - | √ | ? | √ | ? | √ | √ | √ | ? |
| 5. Were the criteria for appraising studies appropriate? | - | √ | - | - | - | - | - | - | - | - | - | - |
| 6. Was critical appraisal conducted by two or more reviewers independently? | - | √ | - | - | - | - | - | - | - | - | - | - |
| 7. Were there methods to minimize errors in data  extraction? | √ | √ | √ | - | √ | ? | √ | ? | √ | √ | √ | ? |
| 8. Were the methods used to combine studies appropriate? | √ | √ | √ | √ | √ | √ | √ | √ | √ | √ | √ | √ |
| 9. Was the likelihood of publication bias assessed? | - | - | - | - | - | - | - | - | - | - | - | - |
| 10. Were recommendations for policy and/or practice  supported by the reported data? | √ | √ | √ | - | √ | √ | - | - | √ | √ | - | - |
| 11. Were the specific directives for new research  appropriate? | √ | √ | √ | - | √ | √ | - | - | √ | √ | - | - |
| **JBI items** | Martins (2019)[13] | Oczkowski (2010)[14] | Pearson (2004)[15] | Pollock (2011)[16] | Rowland (2008)[17] | Salbach (2017)[18] | Salter (2005)[19] | Salter (2005)[20] | Salter (2005)[21] | Scrivener (2013)[22] | [Silva (2014)](#RANGE!_ENREF_5)[23] | Simpson (2013)[24] |
| 1. Is the review question clearly and explicitly stated? | √ | √ | √ | √ | √ | √ | √ | √ | √ | √ | √ | √ |
| 2. Were the inclusion criteria appropriate for the review question? | √ | √ | √ | √ | √ | √ | √ | √ | √ | √ | √ | √ |
| 3. Was the search strategy appropriate? | √ | √ | - | √ | √ | √ | - | - | - | √ | √ | √ |
| 4. Were the sources of studies adequate? | √ | √ | - | √ | √ | √ | - | - | - | √ | √ | - |
| 5. Were the criteria for appraising studies appropriate? | √ | - | - | - | - | √ | - | - | - | √ | √ | - |
| 6. Was critical appraisal conducted by two or more  reviewers independently? | √ | - | - | - | - | √ | - | - | - | √ | √ | - |
| 7. Were there methods to minimize errors in data  extraction? | √ | √ | - | √ | √ | √ | - | - | - | √ | √ | - |
| 8. Were the methods used to combine studies  appropriate? | √ | √ | √ | √ | √ | √ | √ | √ | √ | √ | √ | √ |
| 9. Was the likelihood of publication bias assessed? | - | - | - | - | - | - | - | - | - | - | - | - |
| 10. Were recommendations for policy and/or practice  supported by the reported data? | - | - | - | - | - | √ | - | - | - | √ | √ | √ |
| 11. Were the specific directives for new research  appropriate? | - | - | - | - | - | √ | - | - | - | √ | √ | √ |
| **JBI items** | Sivan (2011)[25] | [Sorrentino (2018)](#RANGE!_ENREF_2)[26] | Stevens (2010)[27] | Teale (2010)[28] | Tse (2013)[29] | Tyson (2009)[30] | Van Bloemendaal (2012)[31] | Van Peppen (2007)[32] | Velstra (2011)[33] | Verheyden (2007)[34] | [Wilde (2010)](#RANGE!_ENREF_6)[35] |  |
| 1. Is the review question clearly and explicitly stated? | √ | √ | √ | √ | √ | √ | √ | √ | √ | √ | √ |  |
| 2. Were the inclusion criteria appropriate for the review question? | √ | √ | √ | √ | √ | √ | √ | √ | √ | √ | √ |  |
| 3. Was the search strategy appropriate? | √ | - | √ | √ | √ | √ | √ | √ | √ | √ | - |  |
| 4. Were the sources of studies adequate? | √ | - | √ | √ | - | √ | √ | √ | ? | √ | - |  |
| 5. Were the criteria for appraising studies appropriate? | - | - | - | - | - | - | √ | - | - | - | - |  |
| 6. Was critical appraisal conducted by two or more  reviewers independently? | - | - | - | - | - | - | √ | - | - | - | - |  |
| 7. Were there methods to minimize errors in data  extraction? | √ | - | √ | √ | - | √ | √ | √ | ? | √ | - |  |
| 8. Were the methods used to combine studies  appropriate? | √ | √ | √ | √ | √ | √ | √ | √ | √ | √ | √ |  |
| 9. Was the likelihood of publication bias assessed? | - | - | - | - | - | - | - | - | - | - | - |  |
| 10. Were recommendations for policy and/or practice  supported by the reported data? | √ | - | - | √ | √ | √ | - | √ | √ | √ | - |  |
| 11. Were the specific directives for new research  appropriate? | √ | - | - | √ | √ | √ | - | √ | √ | √ | - |  |

**References**

1. Ashford, S., Slade, M., Malaprade, F., & Turner-Stokes, L. (2008). Evaluation of functional outcome measures for the hemiparetic upper limb: a systematic review. *Journal of rehabilitation medicine, 40*(10), 787-795.
2. Ashford, S., Brown, S., & Turner-Stokes, L. (2015). Systematic review of patient-reported outcome measures for functional performance in the lower limb. *Journal of rehabilitation medicine, 47*(1), 9-17.
3. Baker, K., Cano, S. J., & Playford, E. D. (2011). Outcome measurement in stroke: a scale selection strategy. *Stroke, 42*(6), 1787-1794.
4. Barak, S., & Duncan, P. W. (2006). Issues in selecting outcome measures to assess functional recovery after stroke. *NeuroRx, 3*(4), 505-524.
5. Connell, L. A., & Tyson, S. F. (2012). Clinical reality of measuring upper-limb ability in neurologic conditions: a systematic review. *Archives of physical medicine and rehabilitation, 93*(2), 221-228.
6. Croarkin, E., Danoff, J., & Barnes, C. (2004). Evidence-based rating of upper-extremity motor function tests used for people following a stroke. *Physical therapy, 84*(1), 62-74.
7. Fini, N. A., Holland, A. E., Keating, J., Simek, J., & Bernhardt, J. (2015). How is physical activity monitored in people following stroke? *Disability and Rehabilitation, 37*(19), 1717-1731.
8. Gebruers, N., Vanroy, C., Truijen, S., Engelborghs, S., & De Deyn, P. P. (2010). Monitoring of physical activity after stroke: a systematic review of accelerometry-based measures. *Archives of physical medicine and rehabilitation, 91*(2), 288-297.
9. Geroin, C., Mazzoleni, S., Smania, N., Gandolfi, M., Bonaiuti, D., Gasperini, G., et al. (2013). Systematic review of outcome measures of walking training using electromechanical and robotic devices in patients with stroke. *Journal of rehabilitation medicine, 45*(10), 987-996.
10. Gor-García-Fogeda, M. D., Molina-Rueda, F., Cuesta-Gómez, A., Carratalá-Tejada, M., Alguacil-Diego, I. M., & Miangolarra-Page, J. C. (2014). Scales to assess gross motor function in stroke patients: a systematic review. *Archives of physical medicine and rehabilitation, 95*(6), 1174-1183.
11. Hong, I., & Bonilha, H. S. (2017). Psychometric properties of upper extremity outcome measures validated by Rasch analysis: a systematic review. *International Journal of Rehabilitation Research, 40*(1), 1-10.
12. Lemmens, R. J., Timmermans, A. A., Janssen-Potten, Y. J., Smeets, R. J., & Seelen, H. A. (2012). Valid and reliable instruments for arm-hand assessment at ICF activity level in persons with hemiplegia: a systematic review. *BMC neurology, 12*(1), 21.
13. Martins, J. C., Aguiar, L. T., Nadeau, S., Scianni, A. A., Teixeira-Salmela, L. F., & Faria, C. D. C. D. M. (2019). Measurement properties of self-report physical activity assessment tools for patients with stroke: a systematic review. *Brazilian journal of physical therapy, 23*(6), 476-490.
14. Oczkowski, C., & O'Donnell, M. (2010). Reliability of proxy respondents for patients with stroke: a systematic review. *Journal of Stroke and Cerebrovascular Diseases, 19*(5), 410-416.
15. Pearson, O. R., Busse, M., Van Deursen, R. W. M., & Wiles, C. M. (2004). Quantification of walking mobility in neurological disorders. *Qjm, 97*(8), 463-475.
16. Pollock, C., Eng, J., & Garland, S. (2011). Clinical measurement of walking balance in people post stroke: a systematic review. *Clinical rehabilitation, 25*(8), 693-708.
17. Rowland, T. J., & Gustafsson, L. (2008). Assessments of upper limb ability following stroke: a review. *British Journal of Occupational Therapy, 71*(10), 427-437.
18. Salbach, N. M., O'brien, K. K., Brooks, D., Irvin, E., Martino, R., Takhar, P., et al. (2017). Considerations for the selection of time-limited walk tests poststroke: a systematic review of test protocols and measurement properties. *Journal of Neurologic Physical Therapy, 41*(1), 3-17.
19. Salter, K., Jutai, J., Teasell, R., Foley, N., & Bitensky, J. (2005). Issues for selection of outcome measures in stroke rehabilitation: ICF Body Functions. *Disability and Rehabilitation, 27*(4), 191-207.
20. Salter, K., Jutai, J., Teasell, R., Foley, N., Bitensky, J., & Bayley, M. (2005). Issues for selection of outcome measures in stroke rehabilitation: ICF Participation. *Disability and Rehabilitation, 27*(9), 507-528.
21. Salter, K., Jutai, J., Teasell, R., Foley, N., Bitensky, J., & Bayley, M. (2005). Issues for selection of outcome measures in stroke rehabilitation: ICF activity. *Disability and Rehabilitation, 27*(6), 315-340.
22. Scrivener, K., Sherrington, C., & Schurr, K. (2013). A systematic review of the responsiveness of lower limb physical performance measures in inpatient care after stroke. *BMC neurology, 13*(1), 4.
23. Silva, P. F., Quintino, L. F., Franco, J., & Faria, C. D. (2014). Measurement properties and feasibility of clinical tests to assess sit-to-stand/stand-to-sit tasks in subjects with neurological disease: a systematic review. *Brazilian journal of physical therapy, 18*(2), 99-110.
24. Simpson, L. A., & Eng, J. J. (2013). Functional recovery following stroke: capturing changes in upper-extremity function. *Neurorehabilitation and neural repair, 27*(3), 240-250.
25. Sivan, M., O'Connor, R. J., Makower, S., Levesley, M., & Bhakta, B. (2011). Systematic review of outcome measures used in the evaluation of robot-assisted upper limb exercise in stroke. *Journal of Rehabilitation Medicine, 43*(3), 181-189.
26. Sorrentino G., S. P., Solaro C., Rabini A., Cerri C., Ferriero G. (2018). Clinical measurement tools to assess trunk performance after stroke: a systematic review. *European journal of physical and rehabilitation medicine*.
27. Stevens, P. M. (2010). Clinimetric properties of timed walking events among patient populations commonly encountered in orthotic and prosthetic rehabilitation. *JPO: Journal of Prosthetics and Orthotics, 22*(1), 62-74.
28. Teale, E. A., & Young, J. B. (2010). A review of stroke outcome measures valid and reliable for administration by postal survey. *Reviews in Clinical Gerontology, 20*(4), 338-353.
29. Tse, T., Douglas, J., Lentin, P., & Carey, L. (2013). Measuring participation after stroke: a review of frequently used tools. *Archives of physical medicine and rehabilitation, 94*(1), 177-192.
30. Tyson, S., & Connell, L. (2009). The psychometric properties and clinical utility of measures of walking and mobility in neurological conditions: a systematic review. *Clinical rehabilitation, 23*(11), 1018-1033.
31. van Bloemendaal, M., van de Water, A. T., & van de Port, I. G. (2012). Walking tests for stroke survivors: a systematic review of their measurement properties. *Disability and Rehabilitation, 34*(26), 2207-2221.
32. Van Peppen, R. P., Hendriks, H., Van Meeteren, N. L., Helders, P. J., & Kwakkel, G. (2007). The development of a clinical practice stroke guideline for physiotherapists in The Netherlands: a systematic review of available evidence. *Disability and Rehabilitation, 29*(10), 767-783.
33. Velstra, I.-M., Ballert, C. S., & Cieza, A. (2011). A systematic literature review of outcome measures for upper extremity function using the international classification of functioning, disability, and health as reference. *PM&R, 3*(9), 846-860.
34. Verheyden, G., Nieuwboer, A., Van de Winckel, A., & De Weerdt, W. (2007). Clinical tools to measure trunk performance after stroke: a systematic review of the literature. *Clinical rehabilitation, 21*(5), 387-394.
35. Wilde, E. A., Whiteneck, G. G., Bogner, J., Bushnik, T., Cifu, D. X., Dikmen, S., et al. (2010). Recommendations for the use of common outcome measures in traumatic brain injury research. *Archives of physical medicine and rehabilitation, 91*(11), 1650-1660. e1617.
