# Supplementary material for: Quality of mobility measures among individuals with acquired brain injury: an umbrella review
Source: Qual Life Res. 2022 Mar 11;31(9):2567–99. doi: 10.1007/s11136-022-03103-4 (PMC9356944; doi:10.1007/s11136-022-03103-4)
Supplement: Supplementary file 4 — Supplementary file4 (DOCX 41 kb) [file 11136_2022_3103_MOESM4_ESM.docx]

**Quality of Mobility Measures among Individuals with Acquired Brain Injury: An Umbrella Review**

Rehab Alhasani, MSc,^1,2,6^ Claudine Auger, PhD,^2,4,5^ Matheus de Paiva Azevedo, BSc,^1^ Sara Ahmed, PhD ^1-3^

**Author affiliations:**

1. School of Physical and Occupation Therapy, Faculty of Medicine, McGill University, Montreal, Canada
2. Centre de Recherche Interdisciplinaire en Réadaptation (CRIR), Montreal, Canada
3. Constance Lethbridge Rehabilitation Center, CIUSSS Centre Ouest de l’ile de Montreal, Montreal, Canada
4. School of Rehabilitation, Faculty of Medicine, University of Montreal, Montreal, Canada
5. Site Institut Universitaire sur la Réadaptation en Déficience Physique de Montréal (IURDPM), CIUSSS Centre-Sud-de-l’Ile-de-Montréal, Montréal, Canada
6. Department of Rehabilitation Sciences, College of Health and Rehabilitation Sciences, Princess Nourah bint Abdulrahman University, Riyadh, Saudi Arabia

**Corresponding author:** Sara Ahmed, PhD, School of Physical and Occupation Therapy, Faculty of Medicine, McGill University, 3655 Sir William-Osler, Montreal, QC, Canada H3G 1Y6. Tel.: 514-398-4400 ext 00531.E-mail: sara.ahmed@mcgill.ca

**Supplementary file 4: Assessment of methodological quality using 4-point COSMIN Risk of Bias rating scale**

| **Name of measure** | **Population** | **Setting** | **SOI** | **Content validity**** | **Internal consistency** | **Test-retest** | **Inter-rater** | **Intra-rater** | **Measurement error** | **Construct validity** | **Responsiveness** |
| --- | --- | --- | --- | --- | --- | --- | --- | --- | --- | --- | --- |
| ABILHAND [1-8] | Stroke | Chronic | PRO | Very good | Very good | Adequate | Adequate | Inadequate | Inadequate | Very good | Very good |
| Accelerometer (ActiGraph) [9] | Stroke | Sub-acute | TechO | Inadequate | Inadequate | Adequate | Inadequate | Inadequate | Inadequate | Adequate | Inadequate |
| Actical [10] | Stroke | Chronic | TechO | Inadequate | Inadequate | Adequate | Inadequate | Inadequate | Inadequate | Inadequate | Inadequate |
| Action Research arm test (ARAT) [2,3,11,4-8,12] | Stroke | Chronic | ClinRO | Very good | Very good | Very good | Very good | Inadequate | Inadequate | Very good | Very good |
| Activities of Daily Living scale [5] | Stroke | Chronic | ObserO | Inadequate | Inadequate | Inadequate | Adequate | Inadequate | Inadequate | Adequate | Inadequate |
| Activity Cart Sort (ACS) [13,14] | Stroke | Chronic | PRO | Adequate | Very good | Adequate | Inadequate | Inadequate | Inadequate | Adequate | Inadequate |
| Actiwatch [9] | Stroke | Acute, Chronic | TechO | Inadequate | Inadequate | Inadequate | Inadequate | Inadequate | Inadequate | Adequate | Inadequate |
| Actual Amount of Use Test (AAUT) [5] | Stroke | Chronic | ClinRO | Inadequate | Inadequate | Adequate | Inadequate | Inadequate | Inadequate | Adequate | Inadequate |
| Ambulatory Monitoring (AM Accelerometer) [9,15] | Stroke | Acute, Chronic | TechO | Inadequate | Inadequate | Inadequate | Inadequate | Inadequate | Inadequate | Adequate | Inadequate |
| Arm Motor Ability Test (AMAT) [4-6,8] | Stroke | Sub-acute, Chronic | PerfO | Adequate | Very good | Very good | Very good | Inadequate | Inadequate | Adequate | Very good |
| Assessment of Life Habits (LIFE-H) [16,14] | Stroke | Chronic | PerfO | Adequate | Inadequate | Adequate | Inadequate | Inadequate | Inadequate | Adequate | Inadequate |
| Assessment of Motor and Process Skills (AMPS) [5] | Stroke | Chronic | PerfO | Inadequate | Inadequate | Adequate | Adequate | Inadequate | Inadequate | Adequate | Inadequate |
| Balance Assessment in Sitting and Standing Position (BASSP) [17] | Stroke | Chronic | ClinRO | Inadequate | Inadequate | Inadequate | Inadequate | Adequate | Inadequate | Adequate | Adequate |
| Balance Evaluation System test (BESTest) [18] | Stroke | Chronic | PerfO | Inadequate | Inadequate | Adequate | Inadequate | Inadequate | Inadequate | Adequate | Very good |
| Barthel Index (BI) [19-23] | Stroke | Chronic, Acute | PerfO | Inadequate | Very good | Very good | Adequate | Adequate | Inadequate | Adequate | Very good |
| Beck Depression Inventory (BDI) [19,24] | Stroke | Chronic, Acute | PRO | Inadequate | Very good | Very good | Inadequate | Inadequate | Inadequate | Very good | Very good |
| Berg Balance Scale (BBS) [19-23] | Stroke | Acute, Sub-acute, Chronic | PerfO | Inadequate | Very good | Very good | Very good | Inadequate | Inadequate | Very good | Very good |
| Berg Balance Scale three point (BBS-3P) [22] | Stroke | Acute | PerfO | Inadequate | Inadequate | Inadequate | Inadequate | Inadequate | Inadequate | Inadequate | Very good |
| Biaxial accelerometer [9] | Stroke | Chronic | TechO | Inadequate | Inadequate | Adequate | Inadequate | Inadequate | Inadequate | Adequate | Inadequate |
| Box and Block test [3,8] | Stroke | Chronic | ClinRO | Inadequate | Inadequate | Very good | Very good | Very good | Inadequate | Very good | Inadequate |
| Brain injury community rehabilitation outcome scale (BICRO) [25] | BI |  | PRO | Inadequate | Very good | Adequate | Adequate | Inadequate | Inadequate | Adequate | Inadequate |
| Brunel Balance Assessment [18] | Stroke | Chronic | ClinRO | Adequate | Inadequate | Adequate | Inadequate | Inadequate | Inadequate | Adequate | Inadequate |
| Caltrac accelerometer [10,9] | Stroke | Chronic | TechO | Inadequate | Inadequate | Inadequate | Inadequate | Inadequate | Inadequate | Inadequate | Inadequate |
| Canadian Occupational Performance Measure (COPM) [5] | Stroke | Chronic | PRO | Inadequate | Inadequate | Adequate | Inadequate | Inadequate | Inadequate | Adequate | Very good |
| Centre for Epidemiological Studies Depression [19] | Stroke | Chronic | PRO | Inadequate | Adequate | Adequate | Adequate | Inadequate | Inadequate | Adequate | Adequate |
| Chedoke Arm and Hand Inventory (CAHAI) [2,5,6,8] | Stroke | Chronic | PerfO | Adequate | Very good | Very good | Adequate | Inadequate | Inadequate | Adequate | Very good |
| Chedoke McMaster Stroke assessment scale (CMSA) [19,11,21,8] | Stroke | Chronic | ClinRO | Inadequate | Very good | Very good | Very good | Adequate | Inadequate | Very good | Very good |
| Climbing stairs questionnaire (CSQ) [25] | Stroke | Chronic | ClinRO | Inadequate | Very good | Adequate | Adequate | Inadequate | Inadequate | Adequate | Inadequate |
| Coded activity diary [13] | Stroke | Chronic | PRO | Inadequate | Inadequate | Inadequate | Inadequate | Inadequate | Inadequate | Adequate | Inadequate |
| Community balance and mobility scale (CB&M) [18,26] | Stroke, TBI | Chronic | PRO | Inadequate | Inadequate | Inadequate | Adequate | Adequate | Inadequate | Adequate | Very good |
| Computer Science and Applications Inc. Model 7164 activity monitors x 4 [10] | Stroke | Chronic |  | Inadequate | Inadequate | Inadequate | Inadequate | Inadequate | Inadequate | Adequate | Inadequate |
| Dimensional gait analysis (3-DGA) [9] | Stroke | Chronic | TechO | Inadequate | Inadequate | Inadequate | Inadequate | Inadequate | Inadequate | Adequate | Inadequate |
| Disabilities of the Arm, Shoulder and Hand (DASH) [4] | Stroke | Chronic | TechO | Inadequate | Very good | Adequate | Inadequate | Inadequate | Inadequate | Adequate | Inadequate |
| Duruoz Hand Index (DHI) [5] | Stroke | Chronic | SRO | Inadequate | Very good | Adequate | Inadequate | Inadequate | Inadequate | Adequate | Inadequate |
| Dynamic Gait Index (DGI) [18,26,15] | Stroke | Sub-acute, Chronic | SRO | Inadequate | Inadequate | Adequate | Adequate | Adequate | Inadequate | Adequate | Inadequate |
| European Quality of life scale (EQ5D) [19,16,27,8,28,29] | Stroke, TBI | Acute, Chronic | PerfO | Inadequate | Inadequate | Adequate | Inadequate | Inadequate | Inadequate | Adequate | Very good |
| Fitbit Ulta [10] | Stroke | Chronic | PRO | Inadequate | Inadequate | Inadequate | Inadequate | Inadequate | Inadequate | Adequate | Very good |
| Fitts Reaching test [3] | Stroke | Chronic | TechO | Inadequate | Inadequate | Adequate | Inadequate | Inadequate | Inadequate | Very good | Inadequate |
| Five meter walking test (5MWT) [30,26,15] | Stroke | Chronic | PerfO | Inadequate | Inadequate | Adequate | Adequate | Adequate | Adequate | Adequate | Inadequate |
| Five times Sit to Stand test [31] | Stroke | Chronic | PerfO | Inadequate | Inadequate | Very good | Very good | Very good | Adequate | Adequate | Very good |
| Footswitches [9,15] | Stroke | Sub-acute, Chronic | PerfO | Inadequate | Inadequate | Adequate | Inadequate | Inadequate | Adequate | Adequate | Inadequate |
| Four metre Comfortable Walk Test (4mCWT) [15] | Stroke | Chronic | TechO | Inadequate | Inadequate | Inadequate | Inadequate | Inadequate | Inadequate | Adequate | Inadequate |
| Four Square Step [18] | Stroke | Chronic | PerfO | Inadequate | Inadequate | Inadequate | Inadequate | Inadequate | Inadequate | Adequate | Very good |
| Frenchay Activities Index (FAI) [13,16,21,14,23] | Stroke | Acute, Chronic | ClinRO | Adequate | Very good | Adequate | Adequate | Inadequate | Inadequate | Adequate | Very good |
| Frenchay Arm Test (FAT) [19,5,8] | Stroke | Chronic | SRO | Inadequate | Inadequate | Adequate | Adequate | Inadequate | Inadequate | Very good | Inadequate |
| Fugl-Meyer Assessment (FMA) [19,32,24,8,12] | Stroke | Acute, Chronic | ClinRO | Inadequate | Very good | Very good | Very good | Inadequate | Inadequate | Very good | Very good |
| Fugl-Meyer Assessment-Upper extremity [4] | Stroke | Chronic | ClinRO | Very good | Inadequate | Adequate | Inadequate | Inadequate | Inadequate | Adequate | Inadequate |
| Fugl-Meyer test-Balance subscale (FM-B) [22] | Stroke | Acute | ClinRO | Inadequate | Inadequate | Inadequate | Inadequate | Inadequate | Inadequate | Inadequate | Very good |
| Function in Sitting Test (FIST) [17] | Stroke | Acute | ClinRO | Inadequate | Very good | Inadequate | Inadequate | Inadequate | Adequate | Adequate | Inadequate |
| Functional Ambulation Category (FAC) [20,22,26,15,23] | Stroke | Acute, Sub-acute, Chronic | PerfO | Inadequate | Inadequate | Adequate | Adequate | Adequate | Inadequate | Adequate | Very good |
| Functional Ambulation Classification Hospital (FACHS) [15] | Stroke | Chronic | ClinRO | Inadequate | Inadequate | Inadequate | Inadequate | Inadequate | Inadequate | Adequate | Inadequate |
| Functional Arm Activity Behavioral Observation System (FAABOS) [5] | ABI |  | ObseRO | Inadequate | Inadequate | Inadequate | Adequate | Inadequate | Inadequate | Inadequate | Inadequate |
| Functional Gait Assessment (FGA) [15] | Stroke | Chronic |  | Inadequate | Inadequate | Inadequate | Adequate | Adequate | Adequate | Inadequate | Inadequate |
| Functional Independence measure (FIM) [19,16,33,21,8,12,29] | Stroke, TBI | Acute, Chronic | PerfO | Inadequate Adequate (TBI) | Very good | Very good | Very good | Inadequate | Inadequate | Very good | Very good |
| Functional Test for the Hemiplegic Upper Extremity (FTHUE) [5] | Stroke | Chronic | ClinRO | Inadequate | Inadequate | Inadequate | Adequate | Inadequate | Inadequate | Inadequate | Inadequate |
| Geriatric Depression scale-long form (GDS) [19] | Stroke | Chronic | ClinRO | Inadequate | Very good | Very good | Inadequate | Inadequate | Inadequate | Adequate | Very good |
| Grasp-Release test [12] | Stroke | Chronic | PRO | Inadequate | Very good | Inadequate | Adequate | Inadequate | Inadequate | Inadequate | Inadequate |
| Grip strength [8] | Stroke | Chronic | PerfO | Inadequate | Inadequate | Adequate | Adequate | Inadequate | Inadequate | Inadequate | Inadequate |
| Grooved Pegboard Test (GPT) [29] | TBI |  | ClinRO | Inadequate | Inadequate | Adequate | Inadequate | Inadequate | Inadequate | Inadequate | Inadequate |
| Hand Function Survey (HFS) [5] | Stroke | Chronic |  | Inadequate | Inadequate | Adequate | Inadequate | Inadequate | Inadequate | Adequate | Inadequate |
| High Level Mobility Assessment (HiMAT) [26] | TBI |  | PerfO | Inadequate | Inadequate | Inadequate | Adequate | Adequate | Inadequate | Adequate | Inadequate |
| Human activity profile (HAP) [25,13] | Stroke | Chronic |  | Inadequate | Very good | Adequate | Adequate | Inadequate | Inadequate | Adequate | Inadequate |
| Intelligent Device for Energy Expenditure and Activity (IDEEA) [10,9] | Stroke | Chronic | PRO | Inadequate | Inadequate | Adequate | Inadequate | Inadequate | Inadequate | Inadequate | Inadequate |
| International classification of functioning, health and disability-Activity measure (ICF-AM) [4] | Stroke | Chronic | TechO | Inadequate | Inadequate | Inadequate | Adequate | Inadequate | Inadequate | Adequate | Inadequate |
| Jebsen Hand Function Test [5,12] | Stroke | Chronic | SRO | Inadequate | Inadequate | Adequate | Inadequate | Inadequate | Inadequate | Adequate | Very good |
| Kinematics [8] | Stroke | Chronic | PerfO | Inadequate | Inadequate | Adequate | Inadequate | Inadequate | Inadequate | Inadequate | Very good |
| London Handicap scale (LHS) [28,14] | Stroke | Acute, Chronic | PRO | Adequate | Very good | Adequate | Adequate | Inadequate | Inadequate | Adequate | Inadequate |
| Manual Function Test (MFT) [5] | Stroke | Acute | PRO | Inadequate | Very good | Adequate | Inadequate | Inadequate | Inadequate | Adequate | Inadequate |
| Mayo-Portland Adaptability Inventory (MPAI-4) [29] | TBI | PRO | ClinRO | Very good | Very good | Inadequate | Very good | Inadequate | Inadequate | Very good | Inadequate |
| Medical Outcomes Study 36-Item Short Form Health Survey (SF-36) [19,16,33,27,12,29] | Stroke, TBI | Chronic |  | Inadequate | Very good | Adequate | Adequate | Inadequate | Inadequate | Very good | Very good |
| Mini Mental State Examination (MMSE) [19,24] | Stroke | Acute, Chronic | PRO | Inadequate | Very good | Adequate | Adequate | Adequate | Inadequate | Adequate | Inadequate |
| Modified Ashworth Scale (AS) [19,24,8,12] | Stroke | Acute | ClinRO | Inadequate | Inadequate | Inadequate | Adequate | Adequate | Inadequate | Adequate | Inadequate |
| Modified Emory Functional Ambulation Profile (M-EFAM) [18,22,26] | Stroke | Acute, Chronic | ClinRO | Inadequate | Inadequate | Adequate | Inadequate | Adequate | Inadequate | Adequate | Very good |
| Modified Functional Reach test (MFRT) [22] | Stroke | Chronic | ClinRO | Inadequate | Inadequate | Inadequate | Inadequate | Inadequate | Inadequate | Inadequate | Very good |
| Modified Rankin Handicap scale [19,21] | Stroke | Acute, Chronic | PerfO | Inadequate | Inadequate | Very good | Adequate | Inadequate | Inadequate | Adequate | Very good |
| Motor activity log (MAL-14) [1,3,5,6,12] | Stroke | Chronic | PRO | Inadequate | Very good | Very good | Inadequate | Inadequate | Inadequate | Very good | Very good |
| Motor Activity Log-28 items [1] | Stroke | Sub-acute | SRO | Inadequate | Very good | Inadequate | Inadequate | Inadequate | Inadequate | Inadequate | Inadequate |
| Motor Assessment Scale (MAS) [19,11,32,22,8] | Stroke | Acute, Chronic | SRO | Inadequate | Inadequate | Very good | Very good | Inadequate | Inadequate | Very good | Very good |
| Motor Evaluation Scale for Upper Extremity in Stroke Patients (MESUPES) [4,5] | Stroke | Chronic | ClinRO | Inadequate | Inadequate | Adequate | Inadequate | Inadequate | Inadequate | Adequate | Inadequate |
| Motor Free Visual Perception Test (MVPT) [19,24] | Stroke | Chronic | ClinRO | Inadequate | Very good | Very good | Inadequate | Inadequate | Inadequate | Adequate | Inadequate |
| Motor status scale (MSS) [3,8] | Stroke | Acute | ClinRO | Inadequate | Inadequate | Adequate | Adequate | Inadequate | Inadequate | Very good | Inadequate |
| Motricity index (MI) [19,11,20,32,23] | Stroke | Chronic | ClinRO | Inadequate | Very good | Adequate | Very good | Inadequate | Inadequate | Very good | Very good |
| Multimedia activity recall for children and adults (MARCA) [13] | Stroke | Chronic | ClinRO | Inadequate | Inadequate | Adequate | Inadequate | Inadequate | Inadequate | Adequate | Inadequate |
| National institute for health Stroke scale (NIHSS) [19] | Stroke | Chronic | SRO | Inadequate | Inadequate | Very good | Inadequate | Inadequate | Inadequate | Very good | Inadequate |
| Neurobehavioral Cognition Status Exam (NCSE) [19] | Stroke | Chronic | ClinRO | Inadequate | Inadequate | Adequate | Inadequate | Inadequate | Inadequate | Adequate | Very good |
| Nike+Fuelband [10] | Stroke | Chronic | ClinRO | Inadequate | Inadequate | Inadequate | Inadequate | Inadequate | Inadequate | Adequate | Very good |
| Nine-Hole Peg test (NHPT) [3,11,8] | Stroke | Chronic | TechO | Inadequate | Inadequate | Very good | Adequate | Inadequate | Inadequate | Very good | Inadequate |
| Nottingham Extended ADL index (N-ADL) [25] | Stroke | Chronic | ClinRO | Inadequate | Very good | Adequate | Adequate | Inadequate | Inadequate | Adequate | Very good |
| Nottingham leisure activity (NLA) [13,27] | Stroke | Chronic | PRO | Inadequate | Very good | Adequate | Adequate | Inadequate | Inadequate | Adequate | Inadequate |
| OMRON HJ-113-E Piezoelectric Pedometers [10] | Stroke | Chronic | PRO | Inadequate | Inadequate | Inadequate | Inadequate | Inadequate | Inadequate |  | Very good |
| Ottawa Sitting Scale (OSS) [17] | Stroke | Chronic | TechO | Inadequate | Inadequate | Inadequate | Adequate | Adequate | Inadequate | Inadequate | Inadequate |
| Outpatient Physical Therapy Improvement in Movement Assessment Log (OPTIMAL) [4] | Stroke | Chronic | ClinRO | Inadequate | Inadequate | Inadequate | Inadequate | Inadequate | Inadequate | Adequate | Inadequate |
| PAL2 (Gorman ProMed Pty. Ltd) [10] | Stroke | Chronic | PRO | Inadequate | Inadequate | Inadequate | Inadequate | Inadequate | Inadequate | Adequate | Inadequate |
| Pedometers [10,9,15] | Stroke | Chronic | TechO | Inadequate | Inadequate | Inadequate | Inadequate | Inadequate | Inadequate | Adequate | Very good |
| Pens taped to feet [26] | TBI |  | TechO | Inadequate | Inadequate | Inadequate | Inadequate | Adequate | Inadequate | Adequate | Inadequate |
| Physical Ability Scale (PAS) [17] | Stroke | Sub-acute |  | Inadequate | Inadequate | Inadequate | Adequate | Adequate | Inadequate | Inadequate | Inadequate |
| Postural Assessment Scale for Stroke Patients (PASS) [22] | Stroke | Chronic | PRO | Inadequate | Inadequate | Inadequate | Inadequate | Inadequate | Inadequate | Inadequate | Very good |
| Postural Assessment Scale for Stroke Patients Trunk Control (PASS-TC) [22] | Stroke | Chronic | PerfO | Inadequate | Inadequate | Inadequate | Inadequate | Inadequate | Inadequate | Inadequate | Very good |
| Postural Control And Balance for Stroke (PCBS) [22] | Stroke | Acute | PerfO | Inadequate | Inadequate | Inadequate | Inadequate | Inadequate | Inadequate | Inadequate | Very good |
| Quadriplegia Index of Function [12] | Stroke | Chronic | PerfO | Inadequate | Inadequate | Very good | Inadequate | Inadequate | Inadequate | Inadequate | Very good |
| Reintegration to normal living index (RNLI) [28] | Stroke | Chronic | ClinRO | Inadequate | Inadequate | Adequate | Inadequate | Inadequate | Inadequate | Adequate | Inadequate |
| Rivermead mobility Index (RMI) [25,20,32,33,21,22,26] | Stroke, TBI | Acute, Sub-acute, Chronic | SRO | Inadequate | Very good | Very good | Very good | Inadequate | Inadequate | Very good | Very good |
| Rivermead mobility Assessment (RMA) [19,3,11,32,4,21,8] | Stroke | Acute, Chronic | PerfO | Inadequate | Very good | Adequate | Inadequate | Inadequate | Adequate | Very good | Inadequate |
| Satisfaction With Life Scale (SWLS) [29] | TBI | PRO | SRO | Inadequate | Inadequate | Very good | Inadequate | Inadequate | Inadequate | Inadequate | Inadequate |
| Sensewear Pro 3 Armband [10] | Stroke | Chronic |  | Inadequate | Inadequate | Inadequate | Inadequate | Inadequate | Inadequate | Adequate | Very good |
| Short Form Berg Balance Scale (SFBBS) [22] | Stroke | Acute | TechO | Inadequate | Inadequate | Inadequate | Inadequate | Inadequate | Inadequate | Inadequate | Very good |
| Short Form Postural Assessment Scale for Stroke Patients-6 items (6 SFPASS) [22] | Stroke | Acute | PerfO | Inadequate | Inadequate | Inadequate | Inadequate | Inadequate | Inadequate | Inadequate | Very good |
| Sickness Impact profile (SIP) [25,19,16,27] | Stroke, TBI | Chronic | PerfO | Inadequate | Very good | Adequate | Inadequate | Inadequate | Inadequate | Adequate | Very good |
| Sitting Rising Test (SRT) [17] | Stroke | Chronic | PRO | Inadequate | Inadequate | Adequate | Adequate | Inadequate | Inadequate | Adequate | Inadequate |
| Six meter walking test (6MWT) [19,20,30,22,26,15] | Stroke, TBI | Acute, Sub-acute, Chronic | ClinRO | Inadequate | Inadequate | Adequate | Adequate | Adequate | Adequate | Adequate | Very good |
| Smart Balance Master (SBM) [22] | Stroke | Acute | PerfO | Inadequate | Inadequate | Inadequate | Inadequate | Inadequate | Inadequate | Inadequate | Very good |
| SmartShoe [10] | Stroke | Chronic | TechO | Inadequate | Inadequate | Inadequate | Inadequate | Inadequate | Inadequate | Adequate | Very good |
| Sodring motor evaluation for Stroke patients [32] | Stroke | Chronic | TechO | Inadequate | Very good | Inadequate | Very good | Inadequate | Inadequate | Very good | Very good |
| Sollerman hand function test [3] | Stroke | Chronic | ClinRO | Inadequate | Inadequate | Very good | Very good | Inadequate | Inadequate | Inadequate | Inadequate |
| Step test [18] | Stroke | Chronic | PerfO | Inadequate | Inadequate | Adequate | Inadequate | Inadequate | Inadequate | Adequate | Very good |
| StepWatch Activity Monitor or Step Activity Monitor (SAM) [10,9,26] | Stroke | Chronic | ClinRO | Inadequate | Inadequate | Adequate | Adequate | Inadequate | Inadequate | Adequate | Very good |
| Stride analyzer system (SAS) [9] | Stroke | Chronic | TechO | Inadequate | Inadequate | Adequate | Inadequate | Inadequate | Inadequate | Adequate | Inadequate |
| Stroke Arm Ladder (SAL) [4] | Stroke | Chronic | TechO | Adequate | Inadequate | Adequate | Inadequate | Inadequate | Inadequate | Adequate | Inadequate |
| Stroke impact scale (SIS) [25,19,16,27,7,8,28,14] | Stroke | Sub-acute, Chronic | ClinRO | Inadequate | Very good | Adequate | Adequate | Inadequate | Inadequate | Very good | Very good |
| Stroke Rehabilitation assessment of movement (STREAM) [19,3,32,4] | Stroke | Chronic | PRO | Adequate | Very good | Very good | Very good | Very good | Inadequate | Very good | Very good |
| Stroke Specific Quality of Life Scale (SSQOL) [19,16,27] | Stroke | Chronic | ClinRO | Inadequate | Very good | Very good | Adequate | Inadequate | Inadequate | Adequate | Very good |
| Subjective index of physical and social outcome (SIPSO) [28] | Stroke | Chronic | PRO | Inadequate | Inadequate | Inadequate | Inadequate | Inadequate | Inadequate | Inadequate | Inadequate |
| Ten meter walking test (10MWT) [19,20,22,34,26,15,23] | Stroke, TBI | Acute, Sub-acute, Chronic | PRO | Inadequate | Inadequate | Very good | Adequate | Adequate | Adequate | Adequate | Very good |
| Thirty metre Comfortable Walk Test (30mCWT) [15] | Stroke | Chronic | PerfO | Inadequate | Inadequate | Inadequate | Inadequate | Inadequate | Inadequate | Adequate | Inadequate |
| Three hundred metre Walk Test in community (300mWT) [15] | Stroke | Chronic | PerfO | Inadequate | Inadequate | Adequate | Inadequate | Inadequate | Inadequate | Adequate | Inadequate |
| Three meter walking test (3MWT) [30] | Stroke | Sub-acute | PerfO | Inadequate | Inadequate | Inadequate | Inadequate | Adequate | Inadequate | Adequate | Inadequate |
| Three Point Postural Assessment Scale for Stroke Patients (PASS-3P) [22] | Stroke | Acute | PerfO | Inadequate | Inadequate | Inadequate | Inadequate | Inadequate | Inadequate | Inadequate | Very good |
| Timed Up and Go test (TUG) [19,18,21,34,26] | Stroke, TBI | Chronic | PerfO | Inadequate | Inadequate | Very good | Adequate | Inadequate | Inadequate | Adequate | Very good |
| Timed walk [33] | Stroke | Chronic | PerfO | Inadequate | Inadequate | Inadequate | Inadequate | Inadequate | Inadequate | Adequate | Very good |
| Triaxial accelerometer/ RT3 [10,9] | Stroke | Chronic | PerfO | Inadequate | Inadequate | Adequate | Inadequate | Inadequate | Inadequate | Adequate | Inadequate |
| Trunk Control Test (TCT) [17,23,35] | Stroke | Chronic | TechO | Inadequate | Very good | Inadequate | Inadequate | Inadequate | Inadequate | Very good | Inadequate |
| Trunk Impairment Scale [17,35] | Stroke | Chronic | ClinRO | Adequate | Very good | Adequate | Very good | Very good | Adequate | Adequate | Very good |
| Trunk Recovery Scale (TRS) [17] | BI |  | ClinRO | Inadequate | Very good | Inadequate | Adequate | Inadequate | Adequate | Adequate | Inadequate |
| Twelve meter walking test (12MWT) [30,26,15] | Stroke | Acute, Sub-acute, Chronic |  | Inadequate | Inadequate | Inadequate | Inadequate | Adequate | Inadequate | Inadequate | Very good |
| Two meter walking test (2MWT) [30,22,26,15] | Stroke | Acute, Chronic | PerfO | Inadequate | Inadequate | Adequate | Adequate | Adequate | Adequate | Inadequate | Very good |
| Uniaxial accelerometer [9] | Stroke | Acute, Chronic | PerfO | Inadequate | Inadequate | Inadequate | Inadequate | Inadequate | Inadequate | Adequate | Inadequate |
| Upper Body Dressing Scale (UBDS) [5] | Stroke | Chronic | TechO | Inadequate | Very good | Inadequate | Adequate | Inadequate | Inadequate | Adequate | Very good |
| Upper Extremity Functional Index (UEFI) [4] | Stroke | Chronic | ClinRO | Inadequate | Inadequate | Inadequate | Adequate | Inadequate | Inadequate | Adequate | Inadequate |
| Upper Extremity Performance Test for Elderly (Test d’Evaluation des Membres supérieurs de Personnes Agées (TEMPA) [5] | Stroke | Chronic | ClinRO | Inadequate | Inadequate | Adequate | Inadequate | Inadequate | Inadequate | Adequate | Inadequate |
| Upper Limb-Motor Assessment Scale (UL-MAS) [4,6] | Stroke | Chronic | ClinRO | Very good | Very good | Very good | Adequate | Adequate | Inadequate | Adequate | Very good |
| Van Lieshout Test Short Form [12] | Stroke | Chronic | ClinRO | Inadequate | Inadequate | Inadequate | Adequate | Inadequate | Inadequate | Inadequate | Very good |
| Wireless Triaxial Accelerometers [10] | Stroke | Chronic | ClinRO | Inadequate | Inadequate | Inadequate | Inadequate | Inadequate | Inadequate | Adequate | Inadequate |
| Wolf Motor Function Test (WMFT) [19,4-8,12] | Stroke | Chronic | PerfO | Very good | Very good | Very good | Very good | Inadequate | Adequate | Adequate | Very good |

_ABI: acquired brain injury, CliInRO: clinicians-reported outcome, COSMIN: COnsensus-based Standards for the selection of health Measurement Instruments, PerfO: performance-reported outcome, PRO: patient-reported outcome, SRO: self-reported outcome, SOI: source of information, OberRO: observation-reported outcome, TechO: technology-reported outcome, TBI: traumatic brain injury
*Inadequate in all measurement properties mean that there is no calculation has been done or no information was reported in the included systematic reviews_

_**Rating of Inadequate was given to content validity, because none of the systematic reviews evaluate the content validity of mobility measures based on Prinsen and Terwee et al. [33] describe three aspects of content validity: (1) relevance (the items of the measure are relevant for the construct of interest within the specific population and context of use); (2) comprehensiveness (all key items are included); and (3) comprehensibility (patients understand all items as intended)._

**References**

1. Ashford, S., Slade, M., Malaprade, F., & Turner-Stokes, L. (2008). Evaluation of functional outcome measures for the hemiparetic upper limb: a systematic review. *Journal of rehabilitation medicine, 40*(10), 787-795.
2. Baker, K., Cano, S. J., & Playford, E. D. (2011). Outcome measurement in stroke: a scale selection strategy. *Stroke, 42*(6), 1787-1794.
3. Connell, L. A., & Tyson, S. F. (2012). Clinical reality of measuring upper-limb ability in neurologic conditions: a systematic review. *Archives of physical medicine and rehabilitation, 93*(2), 221-228.
4. Hong, I., & Bonilha, H. S. (2017). Psychometric properties of upper extremity outcome measures validated by Rasch analysis: a systematic review. *International Journal of Rehabilitation Research, 40*(1), 1-10.
5. Lemmens, R. J., Timmermans, A. A., Janssen-Potten, Y. J., Smeets, R. J., & Seelen, H. A. (2012). Valid and reliable instruments for arm-hand assessment at ICF activity level in persons with hemiplegia: a systematic review. *BMC neurology, 12*(1), 21.
6. Rowland, T. J., & Gustafsson, L. (2008). Assessments of upper limb ability following stroke: a review. *British Journal of Occupational Therapy, 71*(10), 427-437.
7. Simpson, L. A., & Eng, J. J. (2013). Functional recovery following stroke: capturing changes in upper-extremity function. *Neurorehabilitation and neural repair, 27*(3), 240-250.
8. Sivan, M., O'Connor, R. J., Makower, S., Levesley, M., & Bhakta, B. (2011). Systematic review of outcome measures used in the evaluation of robot-assisted upper limb exercise in stroke. *Journal of Rehabilitation Medicine, 43*(3), 181-189.
9. Gebruers, N., Vanroy, C., Truijen, S., Engelborghs, S., & De Deyn, P. P. (2010). Monitoring of physical activity after stroke: a systematic review of accelerometry-based measures. *Archives of physical medicine and rehabilitation, 91*(2), 288-297.
10. Fini, N. A., Holland, A. E., Keating, J., Simek, J., & Bernhardt, J. (2015). How is physical activity monitored in people following stroke? *Disability and Rehabilitation, 37*(19), 1717-1731.
11. Croarkin, E., Danoff, J., & Barnes, C. (2004). Evidence-based rating of upper-extremity motor function tests used for people following a stroke. *Physical therapy, 84*(1), 62-74.
12. Velstra, I.-M., Ballert, C. S., & Cieza, A. (2011). A systematic literature review of outcome measures for upper extremity function using the international classification of functioning, disability, and health as reference. *PM&R, 3*(9), 846-860.
13. Martins, J. C., Aguiar, L. T., Nadeau, S., Scianni, A. A., Teixeira-Salmela, L. F., & Faria, C. D. C. D. M. (2019). Measurement properties of self-report physical activity assessment tools for patients with stroke: a systematic review. *Brazilian journal of physical therapy, 23*(6), 476-490.
14. Tse, T., Douglas, J., Lentin, P., & Carey, L. (2013). Measuring participation after stroke: a review of frequently used tools. *Archives of physical medicine and rehabilitation, 94*(1), 177-192.
15. van Bloemendaal, M., van de Water, A. T., & van de Port, I. G. (2012). Walking tests for stroke survivors: a systematic review of their measurement properties. *Disability and Rehabilitation, 34*(26), 2207-2221.
16. Oczkowski, C., & O'Donnell, M. (2010). Reliability of proxy respondents for patients with stroke: a systematic review. *Journal of Stroke and Cerebrovascular Diseases, 19*(5), 410-416.
17. Sorrentino G., S. P., Solaro C., Rabini A., Cerri C., Ferriero G. (2018). Clinical measurement tools to assess trunk performance after stroke: a systematic review. *European journal of physical and rehabilitation medicine*.
18. Pollock, C., Eng, J., & Garland, S. (2011). Clinical measurement of walking balance in people post stroke: a systematic review. *Clinical rehabilitation, 25*(8), 693-708.
19. Barak, S., & Duncan, P. W. (2006). Issues in selecting outcome measures to assess functional recovery after stroke. *NeuroRx, 3*(4), 505-524.
20. Geroin, C., Mazzoleni, S., Smania, N., Gandolfi, M., Bonaiuti, D., Gasperini, G., et al. (2013). Systematic review of outcome measures of walking training using electromechanical and robotic devices in patients with stroke. *Journal of rehabilitation medicine, 45*(10), 987-996.
21. Salter, K., Jutai, J., Teasell, R., Foley, N., Bitensky, J., & Bayley, M. (2005). Issues for selection of outcome measures in stroke rehabilitation: ICF activity. *Disability and Rehabilitation, 27*(6), 315-340.
22. Scrivener, K., Sherrington, C., & Schurr, K. (2013). A systematic review of the responsiveness of lower limb physical performance measures in inpatient care after stroke. *BMC neurology, 13*(1), 4.
23. Van Peppen, R. P., Hendriks, H., Van Meeteren, N. L., Helders, P. J., & Kwakkel, G. (2007). The development of a clinical practice stroke guideline for physiotherapists in The Netherlands: a systematic review of available evidence. *Disability and Rehabilitation, 29*(10), 767-783.
24. Salter, K., Jutai, J., Teasell, R., Foley, N., & Bitensky, J. (2005). Issues for selection of outcome measures in stroke rehabilitation: ICF Body Functions. *Disability and Rehabilitation, 27*(4), 191-207.
25. Ashford, S., Brown, S., & Turner-Stokes, L. (2015). Systematic review of patient-reported outcome measures for functional performance in the lower limb. *Journal of rehabilitation medicine, 47*(1), 9-17.
26. Tyson, S., & Connell, L. (2009). The psychometric properties and clinical utility of measures of walking and mobility in neurological conditions: a systematic review. *Clinical rehabilitation, 23*(11), 1018-1033.
27. Salter, K., Jutai, J., Teasell, R., Foley, N., Bitensky, J., & Bayley, M. (2005). Issues for selection of outcome measures in stroke rehabilitation: ICF Participation. *Disability and Rehabilitation, 27*(9), 507-528.
28. Teale, E. A., & Young, J. B. (2010). A review of stroke outcome measures valid and reliable for administration by postal survey. *Reviews in Clinical Gerontology, 20*(4), 338-353.
29. Wilde, E. A., Whiteneck, G. G., Bogner, J., Bushnik, T., Cifu, D. X., Dikmen, S., et al. (2010). Recommendations for the use of common outcome measures in traumatic brain injury research. *Archives of physical medicine and rehabilitation, 91*(11), 1650-1660. e1617.
30. Salbach, N. M., O'brien, K. K., Brooks, D., Irvin, E., Martino, R., Takhar, P., et al. (2017). Considerations for the selection of time-limited walk tests poststroke: a systematic review of test protocols and measurement properties. *Journal of Neurologic Physical Therapy, 41*(1), 3-17.
31. Silva, P. F., Quintino, L. F., Franco, J., & Faria, C. D. (2014). Measurement properties and feasibility of clinical tests to assess sit-to-stand/stand-to-sit tasks in subjects with neurological disease: a systematic review. *Brazilian journal of physical therapy, 18*(2), 99-110.
32. Gor-García-Fogeda, M. D., Molina-Rueda, F., Cuesta-Gómez, A., Carratalá-Tejada, M., Alguacil-Diego, I. M., & Miangolarra-Page, J. C. (2014). Scales to assess gross motor function in stroke patients: a systematic review. *Archives of physical medicine and rehabilitation, 95*(6), 1174-1183.
33. Pearson, O. R., Busse, M., Van Deursen, R. W. M., & Wiles, C. M. (2004). Quantification of walking mobility in neurological disorders. *Qjm, 97*(8), 463-475.
34. Stevens, P. M. (2010). Clinimetric properties of timed walking events among patient populations commonly encountered in orthotic and prosthetic rehabilitation. *JPO: Journal of Prosthetics and Orthotics, 22*(1), 62-74.
35. Verheyden, G., Nieuwboer, A., Van de Winckel, A., & De Weerdt, W. (2007). Clinical tools to measure trunk performance after stroke: a systematic review of the literature. *Clinical rehabilitation, 21*(5), 387-394.
