# Supplementary material for: Quality of mobility measures among individuals with acquired brain injury: an umbrella review
Source: Qual Life Res. 2022 Mar 11;31(9):2567–99. doi: 10.1007/s11136-022-03103-4 (PMC9356944; doi:10.1007/s11136-022-03103-4)
Supplement: Supplementary file 5 — Supplementary file5 (DOCX 70 kb) [file 11136_2022_3103_MOESM5_ESM.docx]

**Mobility Measures among Individuals with Acquired Brain Injury: An Umbrella Review**

Rehab Alhasani, MSc,^1,2,6^ Cluadine Auger, PhD,^2,4,5^ Matheus de Paiva Azevedo, BSc,^1^ Sara Ahmed, PhD ^1-3^

**Author affiliations:**

1. School of Physical and Occupation Therapy, Faculty of Medicine, McGill University, Montreal, Canada
2. Centre de Recherche Interdisciplinaire en Réadaptation (CRIR), Montreal, Canada
3. Constance Lethbridge Rehabilitation Center, CIUSSS Centre Ouest de l’ile de Montreal, Montreal, Canada
4. School of Rehabilitation, Faculty of Medicine, University of Montreal, Montreal, Canada
5. Site Institut Universitaire sur la Réadaptation en Déficience Physique de Montréal (IURDPM), CIUSSS Centre-Sud-de-l’Ile-de-Montréal, Montréal, Canada
6. Department of Rehabilitation Sciences, Faculty of Health and Rehabilitation Sciences, Princess Nourah bint Abdulrahman University, Riyadh, Saudi Arabia

**Corresponding author:** Sara Ahmed, PhD, School of Physical and Occupation Therapy, Faculty of Medicine, McGill University, 3655 Sir William-Osler, Montreal, QC, Canada H3G 1Y6. Tel.: 514-398-4400 ext 00531.E-mail: sara.ahmed@mcgill.ca

**Supplementary file 5: Updated criteria for good measurement properties**

| Name of the measure | Type of population | Setting | Sample | Content validity | Internal consistency | test-retest | inter-rater | intra-rater | Measurement error | Construct validity | Responsiveness |
| --- | --- | --- | --- | --- | --- | --- | --- | --- | --- | --- | --- |
| Ashfod (2008) [1] | | | | | | | | | | | |
| ABILHAND | Stroke | Chronic |  | + | + | + | + | ? | ? | + | + |
| Motor activity log (MAL-14) | Stroke | Chronic |  | ? | + | - | - | ? | ? | - | ? |
| Motor Activity Log-28 items | Stroke | Sub-acute | 222 | ? | + | ? | ? | ? | ? | - | ? |
| Ashford (2015) [2] | | | | | | | | | | | |
| Brain injury community rehabilitation outcome scale (BICRO) | Brain Injury |  | 127 | + | + | + | + | ? | ? | + | ? |
| Climbing stairs questionnaire (CSQ) | Stroke | Chronic | 15 | + | + | + | + | ? | ? | - | ? |
| Human activity profile (HAP) | Stroke | Chronic |  | + | + | + | + | ? | ? | - | - |
| Nottingham Extended ADL index (N-ADL) | Stroke | Chronic | 78 | + | + | + | + | ? | ? | + | + |
| Rivermead mobility index (RMI) | Stroke | Acute | 38 | + | + | + | + | ? | ? | + | + |
| Sickness Impact profile (SIP) | Traumatic brain injury |  | 25 | + | + | + | ? | ? | ? | + | + |
| Stroke impact scale (SIS) | Stroke | Chronic |  | + | + | + | + | ? | ? | + | - |
| Baker (2011) [3] | | | | | | | | | | | |
| ABILHAND | Stroke | Chronic |  | ? | ? | + | ? | ? | ? | + | + |
| Action Research Arm test (ARAT) | Stroke | Chronic |  | + | - | ? | ? | ? | ? | - | - |
| Chedoke Arm and Hand Inventory (CAHAI) | Stroke | Chronic | 109 | + | + | + | + | ? | ? | + | ? |
| Fugl-Meyer Assessment (FMA) | Stroke | Chronic |  | ? | - | ? | ? | ? | ? | - | - |
| Stroke Rehabilitation assessment of movement (STREAM) | Stroke | Chronic | 80 | ? | + | + | + | + | ? | + | + |
| Barak (2006) [4] | | | | | | | | | | | |
| Ten Meter Walking Test | Stroke | Sub-acute |  | ? | ? | + | ? | ? | ? | - | + |
| Six Minute Walking Test | Stroke | Sub-acute |  | ? | ? | + | ? | ? | + | + | ? |
| Barthel Index (BI) | Stroke | Chronic | 30 | ? | ? | + | ? | ? | ? | + | + |
| Beck Depression Inventory (BDI) | Stroke | Acute | 202 | ? | ? | + | ? | ? | ? | + | - |
| Berg Balance Scale (BBS) | Stroke | Chronic | 70 | ? | ? | + | ? | ? | ? | + | + |
| Boston diagnostic aphasia exam | Stroke | Chronic |  | ? | ? | + |  |  |  | ? | ? |
| Chedoke McMaster Stroke assessment scale (CMSA) | Stroke | Acute | 32 | ? | ? | + | ? | ? | ? | + | + |
| European Quality of life scale (EQ5D) | Stroke | Chronic |  | ? | ? | - | ? | ? | ? | - | + |
| Frenchay Arm Test (FAT) | Stroke | Chronic | 38 | ? | ? | - | ? | ? | ? | + | - |
| Fugl-Meyer Assessment (FMA) | Stroke | Chronic |  | ? | ? | + | ? | ? | ? | + | + |
| Functional Independance measure (FIM) | Stroke | Chronic |  | ? | ? | + | ? | ? | ? | - | + |
| Geriatric Depression scale-long form (GDS) | Stroke | Chronic |  | ? | + | + | ? | ? | ? | + | + |
| Medical Outcomes Study 36-Item Short Form Health Survey (SF-36) | Stroke | Chronic |  | ? | ? | - | ? | ? | ? | + | + |
| Mini Mental State Examination (MMSE) | Stroke | Acute | 116 | ? | ? | + | ? | ? | ? | - | ? |
| Modified Ashworth scale | Stroke | Acute |  | ? | ? | - | ? | ? | ? | - | ? |
| Modified Rankin Handicap scale | Stroke | Acute | 1034 | ? | ? | + | ? | ? | ? | - | - |
| Motor Assessment Scale (MAS) | Stroke | Chronic |  | ? | ? | + | ? | ? | ? | + | + |
| Motor Free Visual Perception Test | Stroke | Acute |  | ? | ? | + | ? | ? | ? | - | ? |
| Motricity index (MI) | Stroke | Chronic |  | ? | ? | - | ? | ? | ? | + | ? |
| National Institute of Health Stroke Scale | Stroke | Chronic | 65 | ? | ? | + | ? | ? | ? | + | - |
| Neurobehavioral Cognition Status Exam (NCSE) | Stroke | Chronic |  | ? | ? | - | - | ? | ? | + | + |
| Rivermead mobility Assessment (RMA) | Stroke | Chronic |  | ? | ? | - | ? | ? | ? | - | - |
| Sickness Impact profile (SIP) | Stroke | Chronic |  | ? | ? | - | ? | ? | ? | - | ? |
| Stroke impact scale (SIS) | Stroke | Chronic | 696 | ? | ? | - | ? | ? | ? | + | - |
| Stroke Specific Quality of Life Scale (SSQOL) | Stroke | Chronic |  | ? | + | + | ? | ? | ? | - | + |
| Timed Up and Go test (TUG) | Stroke | Chronic |  | ? | ? | + | ? | ? | ? | - | ? |
| Wolf Motor Function Test (WMFT) | Stroke | Chronic |  | ? | + | + | + | ? | ? | - | + |
| Connell (2012) [5] | | | | | | | | | | | |
| ABILHAND | Stroke | Chronic | 103 | ? | ? | ? | ? | ? | ? | + | ? |
| Action Research Arm test (ARAT) | Stroke | Chronic |  | ? | ? | + | + | ? | ? | + | ? |
| Box and Block test | Stroke | Chronic | 15 | ? | ? | + | + | + | ? | + | ? |
| Fitts Reaching test | Stroke | Chronic | 18 | ? | ? | + | ? | ? | ? | + | ? |
| Motor activity log | Stroke | Chronic | 56 | ? | ? | ? | ? | ? | ? | + | ? |
| Motor activity log | Stroke | Chronic | 20 | ? | ? | ? | ? | ? | ? | + | ? |
| Motor activity log | Stroke | Chronic | 41 | ? | ? | ? | ? | ? | ? | + | ? |
| Motor activity log | Stroke | Chronic | 27 | ? | ? | + | ? | ? | ? | ? | ? |
| Motor status score-MSS | Stroke | Acute | 18 | ? | ? | ? | ? | ? | ? | + | ? |
| Nine-Hole Peg test (NHPT) | Stroke | Chronic | 62 | ? | ? | + | ? | ? | ? | + | ? |
| Rivermead mobility Assessment (RMA) | Stroke | Chronic |  | ? | ? | ? | ? | ? | + | + | ? |
| Sollerman hand function test | Stroke | Chronic | 24 | ? | ? | + | + | ? | ? | ? | ? |
| Stroke Rehabilitation assessment of movement (STREAM) | Stroke | Chronic |  | ? | ? | + | ? | ? | ? | + | ? |
| Croarkin (2004) [6] | | | | | | | | | | | |
| Action Research Arm test (ARAT) | Stroke | Chronic |  | ? | ? | ? | + | ? | ? | + | ? |
| Chedoke McMaster Stroke assessment scale (CMSA) | Stroke | Chronic |  | ? | ? | ? | + | ? | ? | + | ? |
| Fugl-Meyer Assessment (FMA) | Stroke | Chronic |  | ? | ? | ? | + | ? | ? | + | ? |
| Motor Assessment Scale (MAS) | Stroke | Chronic |  | ? | ? | ? | + | ? | ? | + | ? |
| Motricity index (MI) | Stroke | Chronic |  | ? | ? | ? | + | ? | ? | + | ? |
| Nine-Hole Peg test (NHPT) | Stroke | Chronic |  | ? | ? | + | - | ? | ? | + | ? |
| Rivermead mobility Assessment (RMA) | Stroke | Chronic |  | ? | ? | ? | - | ? | ? | + | ? |
| Fini (2014) [7] | | | | | | | | | | | |
| Actical | Stroke | Chronic | 40 | ? | ? | + | ? | ? | ? | ? | ? |
| Caltrac accelerometer | Stroke | Chronic | 17 | ? | ? | - | ? | ? | ? | ? | ? |
| Computer Science and Applications Inc. Model 7164 activity monitors x 4 | Stroke | Chronic | 9 | ? | ? | ? | ? | ? | ? | + | ? |
| Fitbit Ulta | Stroke | Chronic | 30 | ? | ? | ? | ? | ? | ? | - | + |
| IDEEA-the Intelligent Device for Energy Expenditure and Activity | Stroke | Chronic | 42 | ? | ? | - | ? | ? | ? | ? | ? |
| Nike+Fuelband | Stroke | Chronic | 30 | ? | ? | ? | ? | ? | ? | - | + |
| OMRON HJ-113-E Piezoelectric Pedometers | Stroke | Chronic | 50 | ? | ? | ? | ? | ? | ? | ? | + |
| PAL2 (Gorman ProMed Pty. Ltd) | Stroke | Chronic | 20 | ? | ? | ? | ? | ? | ? | - | ? |
| Pedometer (Conventional) | Stroke | Chronic | 16 | ? | ? | - | ? | ? | ? | ? | ? |
| Pedometer (model 650 Yamasa Tokei Co., Yamax Digi Walker) | Stroke | Chronic | 20 | ? | ? | ? | ? | ? | ? | - | + |
| Sensewear Pro 3 Armband | Stroke | Chronic | 12 | ? | ? | ? | ? | ? | ? | - | + |
| SmartShoe | Stroke | Chronic | 12 | ? | ? | ? | ? | ? | ? | + | + |
| StepWatch Activity Monitor or Step Activity Monitor (SAM) | Stroke | Chronic | 17 | ? | ? | + | ? | ? | ? | ? | ? |
| StepWatch Activity Monitor or Step Activity Monitor (SAM) | Stroke | Chronic | 16 | ? | ? | + | ? | ? | ? | ? | ? |
| StepWatch Activity Monitor or Step Activity Monitor (SAM) | Stroke | Chronic | 40 | ? | ? | + | ? | ? | ? | ? | ? |
| StepWatch Activity Monitor or Step Activity Monitor (SAM) | Stroke | Chronic | 30 | ? | ? | ? | ? | ? | ? | + | + |
| StepWatch Activity Monitor or Step Activity Monitor (SAM) | Stroke | Chronic | 25 | ? | ? | ? | ? | ? | ? | + | + |
| StepWatch Activity Monitor or Step Activity Monitor (SAM) | Stroke | Chronic | 16 | ? | ? | ? | ? | ? | ? | ? | + |
| Triaxial accelerometer/ RT3 | Stroke | Chronic | 20 | ? | ? | - | ? | ? | ? | ? | ? |
| Wireless Triaxial Accelerometers | Stroke | Chronic | 12 | ? | ? | ? | ? | ? | ? | - | ? |
| Gebruers (2010) [8] | | | | | | | | | | | |
| Accelerometer (ActiGraph) | Stroke | Sub-acute | 20 | ? | ? | + | ? | ? | ? | + | ? |
| Actiwatch | Stroke | Acute | 52 | ? | ? | ? | ? | ? | ? | + | ? |
| Actiwatch | Stroke | Chronic | 11 | ? | ? | ? | ? | ? | ? | + | ? |
| Ambulatory Monitoring (AM Accelerometer) | Stroke | Acute | 43 | ? | ? | ? | ? | ? | ? | + | ? |
| Biaxial accelerometer | Stroke | Chronic | 6 | ? | ? | + | ? | ? | ? | + | ? |
| Caltrac accelerometer | Stroke | Chronic | 27 | ? | ? | - | ? | ? | ? | ? | ? |
| Dimensional gait analysis (3-DGA) | Stroke | Chronic | 25 | ? | ? | ? | ? | ? | ? | + | ? |
| Finger Tapping (uniaxial accelrometer) | Stroke | Chronic | 60 | ? | ? | ? | ? | ? | ? | + | ? |
| Footswitches | Stroke | Chronic | 25 | ? | ? | ? | ? | ? | ? | + | ? |
| Intelligent Device for Energy Expenditure and Activity | Stroke | Chronic | 6 | ? | ? | + | ? | ? | ? | ? | ? |
| Pedometers | Stroke | Chronic | 16 | ? | ? | ? | ? | ? | ? | + | ? |
| StepWatch Activity Monitor or Step Activity Monitor (SAM) | Stroke | Chronic | 16 | ? | ? | + | ? | ? | ? | + | ? |
| StepWatch Activity Monitor or Step Activity Monitor (SAM) | Stroke | Chronic | 21 | ? | ? | ? | ? | ? | ? | + | ? |
| StepWatch Activity Monitor or Step Activity Monitor (SAM) | Stroke | Chronic | 53 | ? | ? | ? | ? | ? | ? | + | ? |
| StepWatch Activity Monitor or Step Activity Monitor (SAM) | Stroke | Chronic | 17 | ? | ? | + | ? | ? | ? | ? | ? |
| StepWatch Activity Monitor or Step Activity Monitor (SAM) | Stroke | Chronic | 50 | ? | ? | ? | ? | ? | ? | + | + |
| StepWatch Activity Monitor or Step Activity Monitor (SAM) | Stroke | Chronic | 25 | ? | ? | ? | ? | ? | ? | + | ? |
| StepWatch Activity Monitor or Step Activity Monitor (SAM) | Stroke | Chronic | 27 | ? | ? | ? | ? | ? | ? | + | ? |
| Stride analyzer system (SAS) | Stroke | Chronic | 6 | ? | ? | + | ? | ? | ? | + | ? |
| Triaxial accelerometer/ RT3 | Stroke | Chronic | 20 | ? | ? | + | ? | ? | ? | + | ? |
| Triaxial accelerometer/ RT3 | Stroke | Chronic | 52 | ? | ? | + | ? | ? | ? | ? | ? |
| Uniaxial accelerometer | Stroke | Acute | 34 | ? | ? | ? | ? | ? | ? | + | ? |
| Uniaxial accelerometer | Stroke | Acute | 45 | ? | ? | ? | ? | ? | ? | + | ? |
| Geroin (2013) [9] | | | | | | | | | | | |
| Ten Meter Waling Test | Stroke | Chronic |  | ? | ? | + | + | ? | ? | - | + |
| Six Minute Walking Test | Stroke | Chronic |  | ? | ? | ? | + | + | ? | - | ? |
| Berg Balance Scale (BBS) | Stroke | Sub-acute | 15 | ? | ? | + | + | ? | ? | - | + |
| Functional Ambulation Category (FAC) | Stroke | Acute |  | ? | ? | + | + | ? | ? | - | + |
| Motricity index (MI) | Stroke | Chronic |  | ? | ? | ? | + | ? | ? | - | ? |
| Rivermead mobility index (RMI) | Stroke | Sub-acute | 73 | ? | + | + | + | ? | ? | - | + |
| Gor-Garcı´a-Fogeda (2014) [10] | | | | | | | | | | | |
| Fugl-Meyer Assessment (FMA) | Stroke | Acute | 78 | ? | + | + | + | ? | ? | + | + |
| Motor Assessment Scale (MAS) | Stroke | Chronic | 37 | ? | ? | + | + | ? | ? | + | ? |
| Stroke Rehabilitation assessment of movement (STREAM) | Stroke | Chronic | 134 | ? | + | + | + | ? | ? | ? | + |
| Motricity index (MI) | Stroke | Chronic | 55 | ? | + | ? | + | ? | ? | + | ? |
| Rivermead mobility Assessment (RMA) | Stroke | Chronic | 158 | ? | + | + | ? | ? | ? | ? | ? |
| Rivermead mobility index (RMI) | Stroke | Chronic |  | ? | + | ? | ? | ? | ? | + | ? |
| Sodring motor evaluation for stroke patients | Stroke | Chronic | 123 | ? | + | ? | + | ? | ? | + | + |
| Hong (2017) [11] | | | | | | | | | | | |
| ABILHAND | Stroke | Chronic | 103 | + | ? | + | ? | ? | ? | + | ? |
| Action Research Arm test (ARAT) | Stroke | Chronic | 191 | + | + | + | ? | ? | ? | + | ? |
| Action Research Arm test (ARAT) | Stroke | Chronic | 351 | + | ? | ? | ? | ? | ? | + | ? |
| Arm Motor Ability Test (AMAT) | Stroke | Chronic | 36 | + | ? | ? | ? | ? | ? | ? | ? |
| DASH (Disabilities of the Arm, Shoulder and Hand) | Stroke | Chronic | 300 | ? | + | + | ? | ? | ? | + | ? |
| Fugl-Meyer Assessment-Upper extremity | Stroke | Chronic | 512 | + | ? | + | ? | ? | ? | + | ? |
| International classification of functioning, health and disability-Activity measure (ICF-AM) | Stroke | Chronic | 317 | ? | ? | ? | + | ? | ? | + | ? |
| MESUPES (Motor Evaluation Scale for Upper Extremity in Stroke Patients) | Stroke | Chronic | 396 | + | ? | + | ? | ? | ? | + | ? |
| OPTIMAL (Outpatient Physical Therapy Improvement in Movement Assessment Log) | Stroke | Chronic | 3138 | ? | ? | ? | ? | ? | ? | + | ? |
| Rivermead mobility Assessment (RMA) | Stroke | Chronic |  | ? | ? | ? | ? | ? | ? | ? | ? |
| Stroke Arm Ladder (SAL) | Stroke | Chronic | 942 | + | ? | + | ? | ? | ? | + | ? |
| Stroke Rehabilitation assessment of movement (STREAM) | Stroke | Chronic | 351 | + | ? | + | ? | ? | ? | + | ? |
| Upper Extremity Functional Index (UEFI) | Stroke | Chronic | 239 | ? | ? | ? | + | ? | ? | + | ? |
| Upper Limb-Motor Assessment Scale (UL-MAS) | Stroke | Chronic | 80 | + | ? | + | ? | ? | ? | + | ? |
| Wolf Motor Function Test (WMFT) | Stroke | Chronic | 189 | + | + | + | ? | ? | ? | + | ? |
| Lemmens (2012) [12] | | | | | | | | | | | |
| ABILHAND | Stroke | Chronic |  | + | ? | ? | + | ? | ? | ? | ? |
| Action Research Arm test (ARAT) | Stroke | Chronic |  | ? | ? | ? | ? | ? | ? | ? | ? |
| Action Research Arm test (ARAT) | Stroke | Chronic |  | ? | ? | ? | ? | ? | ? | ? | ? |
| Activities of Daily Living observation | Stroke | Chronic | 81 | ? | ? | ? | + | ? | ? | + | ? |
| Actual Amount of Use Test (AAUT) | Stroke | Chronic | 11 | ? | ? | + | ? | ? | ? | + | ? |
| Arm Motor Ability Test (AMAT) | Stroke | Chronic |  | ? | ? | ? | ? | ? | ? | ? | ? |
| Assessment of Motor and Process Skills (AMPS) | Stroke | Chronic | 76 | ? | ? | + | + | ? | ? | + | ? |
| Canadian Occupational Performance Measure (COPM) | Stroke | Chronic | 26 | ? | ? | + | ? | ? | ? | + | + |
| Chedoke Arm and Hand Inventory (CAHAI) | Stroke | Chronic |  | ? | ? | ? | ? | ? | ? | ? | ? |
| Duruoz Hand Index (DHI) | Stroke | Chronic | 56 | ? | ? | + | ? | ? | ? | + | ? |
| Frenchay Arm Test (FAT) | Stroke | Chronic | 45 | ? | ? | ? | + | ? | ? | ? | ? |
| Functional Arm Activity Behavioral Observation System (FAABOS) | ABI |  | 9 | ? | ? | ? | + | ? | ? | ? | ? |
| Functional Test for the Hemiplegic Upper Extremity (FTHUE) | Stroke | Chronic | 82 | ? | ? | ? | + | ? | ? | ? | ? |
| Hand Function Survey (HFS) | Stroke | Chronic | 45 | ? | ? | + | ? | ? | ? | + | ? |
| Jebsen Hand Function Test | Stroke | chronic |  | ? | ? | + | ? | ? | ? | + | + |
| Manual Function Test (MFT) | Stroke | Acute | 51 | ? | + | + | ? | ? | ? | + | ? |
| MESUPES (Motor Evaluation Scale for Upper Extremity in Stroke Patients) | Stroke | Chronic |  | ? | ? | ? | ? | ? | ? | ? | ? |
| Motor activity log (MAL-14) | Stroke | Chronic | 56 | ? | ? | ? | ? | ? | ? | ? | ? |
| Upper Body Dressing Scale (UBDS) | Stroke | Chronic | 51 | ? | ? | ? | + | ? | ? | + | + |
| Upper Extremity Performance Test for Elderly (Test d’Evaluation des Membres supérieurs de Personnes Agées (TEMPA) | Stroke | Chronic | 29 | ? | ? | + | ? | ? | ? | + | ? |
| Wolf Motor Function Test (WMFT) | Stroke | Chronic |  | ? | ? | ? | ? | ? | ? | ? | ? |
| Martin (2018) [13] | | | | | | | | | | | |
| Activity Cart Sort (ACS) | Stroke | Chronic | 29 | ? | ? | ? | ? | ? | ? | + | ? |
| Activity Cart Sort (ACS) | Stroke | Chronic | 60 | ? | + | + | ? | ? | ? | + | ? |
| Coded activity diary | Stroke | chronic | 16 | ? | ? | ? | ? | ? | ? | + | ? |
| Frenchay Activities Index (FAI) | Stroke | Chronic | 36 | ? | ? | ? | + | ? | ? | + | ? |
| Frenchay Activities Index (FAI) | Stroke | Chronic | 238 | ? | ? | ? | ? | ? | ? | + | ? |
| Frenchay Activities Index (FAI) | Stroke | Chronic | 52 | ? | ? | + | ? | ? | ? | ? | ? |
| Frenchay Activities Index (FAI) | Stroke | Chronic | 127 | ? | + | ? | ? | ? | ? | ? | ? |
| Frenchay Activities Index (FAI) | Stroke | Chronic | 70 | ? | ? | ? | ? | ? | ? | + | + |
| Frenchay Activities Index (FAI) | Stroke | Chronic | 163 | ? | ? | ? | ? | ? | ? | ? | + |
| Frenchay Activities Index (FAI) | Stroke | Chronic | 45 | ? | ? | ? | + | ? | ? | ? | ? |
| Frenchay Activities Index (FAI) | Stroke | Chronic | 22 | ? | ? | + | ? | ? | ? | ? | ? |
| Frenchay Activities Index (FAI) | Stroke | Chronic | 68 | ? | ? | ? | + | ? | ? | ? | ? |
| Frenchay Activities Index (FAI) | Stroke | Chronic | 188 | ? | + | ? | ? | ? | ? | + | ? |
| Frenchay Activities Index (FAI) | Stroke | Chronic | 14 | ? | ? | ? | + | ? | ? | ? | ? |
| Frenchay Activities Index (FAI) | Stroke | Chronic | 581 | + | ? | ? | ? | ? | ? | ? | ? |
| Frenchay Activities Index (FAI) | Stroke | Chronic | 935 | ? | ? | ? | ? | ? | ? | + | ? |
| Frenchay Activities Index (FAI) | Stroke | Chronic | 383 | ? | ? | ? | ? | ? | ? | ? | + |
| Frenchay Activities Index (FAI) | Stroke | Chronic |  | ? | ? | + | ? | ? | ? | + | ? |
| Human activity profile (HAP) | Stroke | Chronic | 24 | ? | ? | ? | ? | ? | ? | + | ? |
| Multimedia activity recall for children and adults (MARCA) | Stroke | Chronic | 40 | ? | ? | + | ? | ? | ? | + | ? |
| Nottingham leisure activity (NLA) | Stroke | Chronic | 21 | ? | ? | - | ? | ? | ? | ? | ? |
| Nottingham leisure activity (NLA) | Stroke | Chronic | 20 | ? | ? | ? | - | ? | ? | ? | ? |
| Oczkowski (2010) [14] | | | | | | | | | | | |
| Assessment of Life Habits (LIFE-H) | Stroke | Chronic | 80 | ? | ? | + | ? | ? | ? | ? | ? |
| European Quality of life scale-EQ5D | Stroke | Chronic | 15 | ? | ? | + | ? | ? | ? | ? | ? |
| Frenchay Activities Index (FAI) | Stroke | Chronic |  | ? | ? | - | ? | ? | ? | ? | ? |
| Functional Independence measure (FIM) | Stroke | Chronic |  | ? | ? | + | ? | ? | ? | ? | ? |
| Medical Outcomes Study 36-Item Short Form Health Survey (SF-36) | Stroke | Chronic | 60 | ? | ? | - | ? | ? | ? | ? | ? |
| Sickness Impact profile (SIP) | Stroke | Chronic | 574 | ? | ? | + | ? | ? | ? | - | ? |
| Sickness impact profile (SIP) | Stroke | Chronic |  | ? | ? | + | ? | ? | ? | ? | ? |
| Stroke impact scale (SIS) | Stroke | Chronic |  | ? | ? | - | ? | ? | ? | ? | ? |
| Stroke Specific Quality of Life Scale (SSQOL) | Stroke | Chronic |  | ? | ? | - | ? | ? | ? | ? | ? |
| Pearson (2004) [15] | | | | | | | | | | | |
| Barthel Index (BI) | Stroke | Chronic |  | ? | ? | + | ? | + | ? | + | + |
| Functional Independence measure (FIM) | Stroke | Chronic |  | ? | ? | ? | + | + | ? | + | + |
| Medical Outcomes Study 36-Item Short Form Health Survey (SF-36) | Stroke | Chronic |  | ? | + | ? | ? | ? | ? | + | ? |
| Rivermead mobility index (RMI) | Stroke | Chronic |  | ? | ? | + | ? | ? | ? | + | ? |
| Timed walk | Stroke | Chronic | 22 | + | ? | ? | ? | + | ? | + | + |
| Poolack (2015) [16] | | | | | | | | | | | |
| Balance Evaluation System test (BESTest) | Stroke | Chronic | 115 | + | ? | + | ? | ? | ? | + | + |
| Brunel Balance Assessment | Stroke | Chronic | 92 | + | ? | + | ? | ? | ? | + | ? |
| Community balance and mobility scale (CB&M) | Stroke | Chronic | 44 | ? | ? | ? | ? | ? | ? | + | + |
| Dynamic Gait Index (DGI) | Stroke | Chronic |  | ? | ? | + | - | - | ? | ? | ? |
| Four Square Step | Stroke | Chronic | 37 | - | ? | ? | ? | ? | ? | + | + |
| Modified Emory Functional Ambulation Profile (M-EFAM) | Stroke | Chronic | 26 | ? | ? | + | ? | ? | ? | + | + |
| Step test | Stroke | Chronic | 41 | + | ? | + | ? | ? | ? | + | + |
| Timed Up and Go test (TUG) | Stroke | Chronic | 50 | - | ? | + | ? | ? | ? | ? | + |
| Timed Up and Go test (TUG) | Stroke | Chronic | 11 | ? | ? | + | ? | ? | ? | ? | ? |
| Timed Up and Go test (TUG) | Stroke | Chronic | 44 | ? | ? | ? | ? | ? | ? | + | + |
| Rowland (2008) [17] | | | | | | | | | | | |
| ABILHAND | Stroke | Chronic |  | + | ? | + | ? | ? | ? | - | ? |
| Action Research Arm test (ARAT) | Stroke | Chronic |  | ? | + | + | + | ? | ? | + | + |
| Arm Motor Ability Test (AMAT) | Stroke | Sub-acute | 32 | ? | + | + | - | ? | ? | - | + |
| Chedoke Arm and Hand Inventory (CAHAI) | Stroke | Chronic |  | + | + | + | - | ? | ? | + | + |
| Motor Activity Log | Stroke | Chronic |  | + | + | - | ? | ? | ? | - | + |
| Upper Limb-Motor Assessment Scale (UL-MAS) | Stroke | Chronic |  | + | + | + | + | + | ? | + | + |
| Wolf Motor Function Test (WMFT) | Stroke | Chronic |  | ? | + | + | + | ? | ? | - | ? |
| Salbach (2017) [18] | | | | | | | | | | | |
| Twelve Meter Walking Test | Stroke | Chronic | 25 | + | ? | ? | ? | ? | ? | + | ? |
| Twelve Meter Walking Test | Stroke | Sub-acute | 20 | + | ? | ? | ? | + | ? | + | ? |
| Two Meter Walking Test | Stroke | Chronic | 61 | + | ? | ? | ? | + | + | ? | ? |
| Two Meter Walking Test | Stroke | Chronic | 12 | + | ? | ? | ? | + | + | ? | ? |
| Two Meter Walking Test | Stroke | Chronic | 32 | + | ? | ? | ? | + | + | ? | ? |
| Two Meter Walking Test | Stroke | Chronic | 17 | + | ? | ? | ? | + | + | ? | ? |
| Three Meter Walking Test | Stroke | Sub-acute | 14 | + | ? | ? | ? | + | ? | + | ? |
| Five Meter Walking Test | Stroke | Chronic | 9 | + | ? | ? | ? | + | + | ? | ? |
| Five Meter Walking Test | Stroke | Chronic | 20 | + | ? | ? | ? | ? | ? | + | ? |
| Five Meter Walking Test | Stroke | Chronic | 10 | + | ? | ? | ? | ? | ? | + | ? |
| Six Minute Walking Test | Stroke | Acute | 37 | + | ? | ? | ? | + | + | ? | ? |
| Six Minute Walking Test | Stroke | Acute | 24 | + | ? | ? | ? | + | + | ? | ? |
| Six Minute Walking Test | Stroke | Acute | 13 | + | ? | ? | ? | + | + | ? | ? |
| Six Minute Walking Test | Stroke | Acute | 41 | + | ? | ? | ? | ? | ? | + | ? |
| Six Minute Walking Test | Stroke | Acute | 30 | + | ? | ? | ? | ? | ? | + | ? |
| Six Minute Walking Test | Stroke | Chronic | 83 | + | ? | ? | ? | + | ? | ? | ? |
| Six Minute Walking Test | Stroke | Chronic | 12 | + | ? | ? | ? | + | + | ? | ? |
| Six Minute Walking Test | Stroke | Chronic | 10 | + | ? | ? | ? | + | ? | ? | ? |
| Six Minute Walking Test | Stroke | Chronic | 50 | + | ? | ? | ? | + | + | ? | ? |
| Six Minute Walking Test | Stroke | Chronic | 27 | + | ? | ? | ? | + | + | ? | ? |
| Six Minute Walking Test | Stroke | Chronic | 27 | + | ? | ? | ? | ? | ? | + | ? |
| Six Minute Walking Test | Stroke | Chronic | 36 | + | ? | ? | ? | ? | ? | + | ? |
| Six Minute Walking Test | Stroke | Chronic | 34 | + | ? | ? | ? | ? | ? | + | ? |
| Six Minute Walking Test | Stroke | Chronic | 50 | + | ? | ? | ? | ? | ? | + | ? |
| Six Minute Walking Test | Stroke | Chronic | 61 | + | ? | ? | ? | ? | ? | + | ? |
| Six Minute Walking Test | Stroke | Chronic | 64 | + | ? | ? | ? | ? | ? | + | ? |
| Six Minute Walking Test | Stroke | Chronic | 12 | + | ? | ? | ? | ? | ? | + | ? |
| Six Minute Walking Test | Stroke | Chronic | 34 | + | ? | ? | ? | ? | ? | + | ? |
| Six Minute Walking Test | Stroke | Chronic | 25 | + | ? | ? | ? | ? | ? | + | ? |
| Six Minute Walking Test | Stroke | Chronic | 48 | + | ? | ? | ? | ? | ? | + | ? |
| Six Minute Walking Test | Stroke | Chronic | 30 | + | ? | ? | ? | ? | ? | + | ? |
| Six Minute Walking Test | Stroke | Chronic | 21 | + | ? | ? | ? | ? | ? | + | ? |
| Six Minute Walking Test | Stroke | Chronic | 40 | + | ? | ? | ? | ? | ? | + | ? |
| Six Minute Walking Test | Stroke | Chronic | 77 | + | ? | ? | ? | ? | ? | + | ? |
| Six Minute Walking Test | Stroke | Chronic | 40 | + | ? | ? | ? | ? | ? | + | ? |
| Six Minute Walking Test | Stroke | Chronic | 49 | + | ? | ? | ? | ? | ? | + | ? |
| Six Minute Walking Test | Stroke | Chronic | 17 | + | ? | ? | ? | ? | ? | + | ? |
| Six Minute Walking Test | Stroke | Chronic | 42 | + | ? | ? | ? | ? | ? | + | ? |
| Six Minute Walking Test | Stroke | Chronic | 50 | + | ? | ? | ? | ? | ? | + | ? |
| Six Minute Walking Test | Stroke | Chronic | 68 | + | ? | ? | ? | ? | ? | + | ? |
| Six Minute Walking Test | Stroke | Chronic | 25 | + | ? | ? | ? | ? | ? | + | ? |
| Six Minute Walking Test | Stroke | Chronic | 25 | + | ? | ? | ? | ? | ? | + | ? |
| Six Minute Walking Test | Stroke | Chronic | 30 | + | ? | ? | ? | ? | ? | + | ? |
| Six Minute Walking Test | Stroke | Chronic | 77 | + | ? | ? | ? | ? | ? | + | ? |
| Six Minute Walking Test | Stroke | Sub- acute | 50 | + | ? | ? | ? | ? | ? | + | ? |
| Six Minute Walking Test | Stroke | Sub-acute | 63 | + | ? | ? | ? | ? | ? | + | ? |
| Six Minute Walking Test | Stroke | Sub-acute | 48 | + | ? | ? | ? | ? | ? | + | ? |
| Six Minute Walking Test | Stroke | Sub-acute | 25 | + | ? | ? | ? | ? | ? | + | ? |
| Salter (2005) [19] | | | | | | | | | | | |
| Barthel Index (BI) | Stroke | Acute | 22 | ? | + | + | + | + | ? | + | + |
| Berg Balance Scale (BBS) | Stroke | Acute |  | ? | + | + | + | ? | ? | + | + |
| Frenchay Activities Index (FAI) | stroke | Acute | 35 | ? | + | + | + | ? | ? | - | ? |
| Functional Independence measure (FIM) | Stroke | Acute | 52 | ? | + | + | + | ? | ? | + | + |
| Modified Rankin Handicap Scale | Stroke | Chronic | 63 | ? | ? | + | - | ? | ? | + | + |
| Rivermead mobility Assessment (RMA) | Stroke | Acute | 51 | ? | ? | - | ? | ? | ? | + | ? |
| Rivermead mobility index (RMI) | Stroke | Acute |  | + | + | + | + | ? | ? | + | + |
| Timed Up and Go test (TUG) | Stroke | Chronic |  | ? | ? | + | + | ? | ? | - | + |
| Chedoke McMaster Stroke assessment scale (CMSA) | Stroke | Chronic | 127 | ? | + | + | + | + | ? | + | + |
| Salter (2005) [20] | | | | | | | | | | | |
| Beck Depression Inventory (BDI) | Stroke | Chronic | 202 | + | + | - | ? | ? | ? | + | + |
| Fugl-Meyer Assessment (FMA) | Stroke | Acute |  | ? | + | ? | + | + | ? | + | + |
| Mini-Mental State Examination (MMSE) | Stroke | Chronic | 75 | ? | - | - | - | + | ? | + | ? |
| Modified Ashworth scale | Stroke | Acute |  | ? | ? | ? | - | - | ? | + | ? |
| Motor-free Visual Perception Test (MVPT) | Stroke | Acute | 30 | ? | - | + | ? | ? | ? | + | ? |
| Salter (2005) [21] | | | | | | | | | | | |
| European Quality of life scale (EQ5D) | Stroke | Chronic |  | ? | ? | - | ? | ? | ? | + | ? |
| Medical Outcomes Study 36-Item Short Form Health Survey (SF-36) | Stroke | Chronic |  | ? | + | + | ? | ? | ? | + | + |
| Nottingham leisure activity (NLA) | Stroke | Chronic |  | ? | + | + | - | ? | ? | + | ? |
| Sickness Impact profile (SIP) | Stroke | Chronic |  | ? | + | ? | ? | ? | ? | + | ? |
| Stroke Impact Scale (SIS) | Stroke | Chronic |  | ? | + | + | ? | - | ? | + | + |
| Stroke Specific Quality of Life Scale (SSQOL) | Stroke | Chronic | 71 | ? | + | ? | - | ? | ? | + | + |
| Scrivener (2013) [22] | | | | | | | | | | | |
| 10MWT | Stroke | Acute | 42 | ? | ? | ? | ? | ? | ? | ? | + |
| 10MWT | Stroke | Acute | 50 | ? | ? | ? | ? | ? | ? | ? | + |
| 12MWT | Stroke | Chronic | 18 | ? | ? | ? | ? | ? | ? | ? | + |
| 2MWT | Stroke | Acute | 18 | ? | ? | + | + | ? | ? | ? | + |
| 6-item Short Form Postural Assessment Scale for Stroke Patients (6 SFPASS) | Stroke | Acute | 262 | ? | ? | ? | ? | ? | ? | ? | + |
| 6MWT | Stroke | Chronic | 18 | ? | ? | ? | ? | ? | ? | ? | + |
| Berg Balance Scale (BBS) | Stroke | Acute | 50 | ? | ? | ? | ? | ? | ? | ? | + |
| Berg Balance Scale (BBS) | Stroke | Acute | 110 | ? | ? | ? | ? | ? | ? | ? | + |
| Berg Balance Scale (BBS) | Stroke | Acute | 93 | ? | ? | ? | ? | ? | ? | ? | + |
| Berg Balance Scale (BBS) | Stroke | Acute | 80 | ? | ? | ? | ? | ? | ? | ? | + |
| Berg Balance Scale (BBS) | Stroke | Acute | 93 | ? | ? | ? | ? | ? | ? | ? | + |
| Berg Balance Scale (BBS) | Stroke | Acute | 80 | ? | ? | ? | ? | ? | ? | ? | + |
| Berg Balance Scale (BBS) | Stroke | Acute | 60 | ? | ? | ? | ? | ? | ? | ? | + |
| Berg Balance Scale three point (BBS-3P) | Stroke | Acute | 202 | ? | ? | ? | ? | ? | ? | ? | + |
| Berg Balance Scale three point (BBS-3P) | Stroke | Acute | 167 | ? | ? | ? | ? | ? | ? | ? | + |
| Berg Balance Scale three point (BBS-3P) | Stroke | Acute | 167 | ? | ? | ? | ? | ? | ? | ? | + |
| Fugl-Meyer test-Balance subscale (FM-B) | Stroke | Acute | 110 | ? | ? | ? | ? | ? | ? | ? | + |
| Fugl-Meyer test-Balance subscale (FM-B) | Stroke | Acute | 93 | ? | ? | ? | ? | ? | ? | ? | + |
| Fugl-Meyer test-Balance subscale (FM-B) | Stroke | Acute | 80 | ? | ? | ? | ? | ? | ? | ? | + |
| Fugl-Meyer test-Balance subscale (FM-B) | Stroke | Acute | 93 | ? | ? | ? | ? | ? | ? | ? | + |
| Fugl-Meyer test-Balance subscale (FM-B) | Stroke | Acute | 80 | ? | ? | ? | ? | ? | ? | ? | + |
| Functional Ambulation Category (FAC) | Stroke | Acute | 101 | ? | ? | ? | ? | ? | ? | ? | + |
| Functional Ambulation Category (FAC) | Stroke | Sub-acute | 55 | ? | ? | ? | ? | ? | ? | ? | + |
| Modified Emory Functional Ambulation Profile (M-EFAM) | Stroke | Acute | 40 | ? | ? | ? | ? | ? | ? | ? | + |
| Modified Functional Reach test (MFRT) | Stroke | Chronic | 35 | ? | ? | ? | ? | ? | ? | ? | + |
| Motor Assessment Scale (MAS) | Stroke | Acute | 61 | ? | ? | ? | ? | ? | ? | ? | + |
| Postural Assessment Scale for Stroke Patients (PASS) | Stroke | Acute | 202 | ? | ? | ? | ? | ? | ? | ? | + |
| Postural Assessment Scale for Stroke Patients (PASS) | Stroke | Chronic | 167 | ? | ? | ? | ? | ? | ? | ? | + |
| Postural Assessment Scale for Stroke Patients (PASS) | Stroke | Chronic | 167 | ? | ? | ? | ? | ? | ? | ? | + |
| Postural Assessment Scale for Stroke Patients Trunk Control (PASS-TC) | Stroke | Acute | 110 | ? | ? | ? | ? | ? | ? | ? | + |
| Postural Assessment Scale for Stroke Patients Trunk Control (PASS-TC) | Stroke | Chronic | 93 | ? | ? | ? | ? | ? | ? | ? | + |
| Postural Assessment Scale for Stroke Patients Trunk Control (PASS-TC) | Stroke | Chronic | 80 | ? | ? | ? | ? | ? | ? | ? | + |
| Postural Assessment Scale for Stroke Patients Trunk Control (PASS-TC) | Stroke | Chronic | 93 | ? | ? | ? | ? | ? | ? | ? | + |
| Postural Assessment Scale for Stroke Patients Trunk Control (PASS-TC) | Stroke | Chronic | 80 | ? | ? | ? | ? | ? | ? | ? | + |
| Postural Assessment Scale for Stroke Patients Trunk Control (PASS-TC) | Stroke | Acute | 246 | ? | ? | ? | ? | ? | ? | ? | + |
| Postural Assessment Scale for Stroke Patients Trunk Control (PASS-TC) | Stroke | Chronic | 203 | ? | ? | ? | ? | ? | ? | ? | + |
| Postural Assessment Scale for Stroke Patients Trunk Control (PASS-TC) | Stroke | Chronic | 189 | ? | ? | ? | ? | ? | ? | ? | + |
| Postural Control And Balance for Stroke (PCBS) | Stroke | Acute | 50 | ? | ? | ? | ? | ? | ? | ? | + |
| Rivermead mobility index (RMI) | Stroke | Chronic |  | ? | ? | ? | ? | ? | ? | ? | ? |
| Rivermead mobility index (RMI) | Stroke | Chronic |  | ? | ? | ? | ? | ? | ? | ? | ? |
| Short Form Berg Balance Scale (SFBBS) | Stroke | Acute | 81 | ? | ? | ? | ? | ? | ? | ? | + |
| Smart Balance Master (SBM) | Stroke | Acute | 40 | ? | ? | ? | ? | ? | ? | ? | + |
| Three Point Postural Assessment Scale for Stroke Patients (PASS-3P) | Stroke | Acute | 202 | ? | ? | ? | ? | ? | ? | ? | + |
| Three Point Postural Assessment Scale for Stroke Patients (PASS-3P) | Stroke | Acute | 167 | ? | ? | ? | ? | ? | ? | ? | + |
| Three Point Postural Assessment Scale for Stroke Patients (PASS-3P) | Stroke | Acute | 167 | ? | ? | ? | ? | ? | ? | ? | + |
| Silva (2013) [23] | | | | | | | | | | | |
| Five times Sit to Stand test | Stroke | Chronic | 19 | + | ? | + | ? | ? | + | ? | ? |
| Five times Sit to Stand test | Stroke | Chronic | 12 | + | ? | + | + | + | ? | + | + |
| Five times Sit to Stand test | Stroke | Chronic | 27 | + | ? | ? | ? | ? | ? | + | ? |
| Simpson (2013) [24] | | | | | | | | | | | |
| ABILHAND | Stroke | Chronic |  | ? | ? | ? | ? | ? | ? | ? | + |
| Action Research Arm test (ARAT) | Stroke | Chronic |  | ? | ? | ? | ? | ? | ? | + | ? |
| Stroke impact scale (SIS) | Stroke | Chronic |  | ? | ? | ? | ? | ? | ? | ? | + |
| Wolf Motor Function Test (WMFT) | Stroke | Chronic |  | ? | ? | ? | ? | ? | ? | + | ? |
| Sivan (2001) [25] | | | | | | | | | | | |
| ABILHAND | Stroke | Chronic |  | ? | ? | + | ? | ? | ? | - | ? |
| Action Research Arm test | Stroke | Chronic |  | ? | ? | + | + | ? | ? | + | + |
| Arm Motor Ability Test (AMAT) | Stroke | Chronic |  | ? | ? | + | + | ? | ? | ? | ? |
| Barthel Index (BI) | Stroke | Chronic | 167 | ? | ? | + | + | ? | ? | - | + |
| Box and Block test | Stroke | Chronic | 37 | ? | ? | + | ? | ? | ? | - | - |
| Chedoke Arm and Hand Inventory (CAHAI) | Stroke | Chronic |  | ? | ? | ? | + | ? | ? | ? | + |
| Chedoke McMaster Stroke assessment scale (CMSA) | Stroke | Chronic |  | ? | ? | ? | + | ? | ? | - | ? |
| European Quality of life scale (EQ5D) | Stroke | Acute |  | ? | ? | + | ? | ? | ? | - | ? |
| Frenchay Arm Test (FAT) | Stroke | Chronic | 10 | ? | ? | + | + | ? | ? | ? | ? |
| Fugl-Meyer Assessment (FMA) | Stroke | Acute |  | ? | ? | + | + | ? | ? | - | + |
| Grip strength | stroke | Chronic | 27 | ? | ? | + | + | ? | ? | ? | ? |
| Kinematics | stroke | Chronic | 8 | ? | ? | + | ? | ? | ? | - | + |
| Modified Ashworth scale | Stroke | Acute |  | ? | ? | + | + | ? | ? | - | ? |
| Motor Assessment Scale (MAS) | Stroke | Chronic |  | ? | ? | + | + | ? | ? | - | ? |
| Motor status score-MSS | Stroke | Acute |  | ? | ? | + | + | ? | ? | - | ? |
| Nine-Hole Peg test (NHPT) | Stroke | Chronic |  | ? | ? | ? | + | ? | ? | - | ? |
| Rivermead mobility Assessment (RMA) | Stroke | Chronic |  | ? | ? | + | ? | ? | ? | - | ? |
| Stroke impact scale (SIS) | Stroke | Chronic |  | ? | ? | + | ? | ? | ? | - | ? |
| Wolf Motor Function Test (WMFT) | Stroke | Chronic |  | ? | ? | + | + | ? | ? | - | + |
| Functional Independence measure (FIM) | Stroke | Chronic | 83 | ? | ? | + | + | ? | ? | - | + |
| Sorrentino (2018) [26] | | | | | | | | | | | |
| Balance Assessment in Sitting and Standing Position (BASSP) | Stroke | Chronic | 1193 | ? | ? | ? | ? | + | ? | + | + |
| Function in Sitting Test (FIST) | Stroke | Acute | 31 | + | + | ? | ? | ? | + | + | ? |
| Modified Functional Reach test (MFRT) | Stroke | Chronic |  | ? | ? | + | ? | + | ? | ? | + |
| Ottawa Sitting Scale (OSS) | Stroke | Chronic | 71 | ? | ? | ? | + | + | ? | ? | ? |
| Physical Ability Scale (PAS) | Stroke | Sub-acute | 10 | ? | ? | ? | - | - | ? | ? | ? |
| Postural Assessment Scale for Stroke Patients (PASS) | Stroke | Chronic |  | ? | ? | ? | ? | ? | ? | ? | + |
| Sitting Rising Test (SRT) | Stroke | Chronic | 60 | ? | ? | + | + | ? | ? | + | ? |
| Trunk Control test | Stroke | Chronic |  | ? | + | ? | + | ? | ? | + | ? |
| Trunk Impairment Scale | Stroke | Chronic |  | ? | + | ? | + | + | ? | ? | ? |
| Trunk Impairment Scale | Stroke | Chronic |  | ? | + | + | + | + | ? | ? | ? |
| Trunk Impairment Scale (TIS)- Verheyden version | Stroke | Chronic |  | ? | - | + | + | ? | ? | + | ? |
| Trunk Impairment Scale - Fujiwara version | Stroke | Chronic |  | ? | + | ? | + | ? | ? | + | + |
| Trunk Recovery Scale (TRS) | Brain Injury |  | 59 | ? | + | ? | + | ? | + | + | ? |
| Stevens (2010) [27] | | | | | | | | | | | |
| Ten Meter Walking test | Stroke | Chronic |  | ? | ? | + | ? | ? | ? | ? | + |
| Ten Meter Walking test | Stroke | Sub-acute |  | ? | ? | + | ? | ? | ? | ? | ? |
| Ten Meter Walking test | Traumatic brain injury |  | 94 | ? | ? | + | ? | ? | ? | ? | ? |
| Five Meter Walking Test | Stroke | Chronic |  | ? | ? | + | ? | ? | ? | ? | ? |
| Six Minute Walking Test | Stroke | chronic |  | ? | ? | + | + | ? | ? | ? | ? |
| Six Minute Walking Test | Traumatic brain injury |  | 36 | ? | ? | + | ? | ? | ? | ? | ? |
| Timed Up and Go test (TUG) | Stroke | Chronic | 343 | ? | ? | + | ? | ? | ? | ? | ? |
| Timed Up and Go test (TUG) | Traumatic brain injury |  | 24 | ? | ? | + | ? | ? | ? | ? | ? |
| Teale (2010) [28] | | | | | | | | | | | |
| European Quality of life scale (EQ5D) | Stroke | Chronic |  | ? | ? | + | ? | ? | ? | ? | ? |
| London handicap score-LHS | Stroke | Acute | 361 | + | ? | + | + | ? | ? | + | ? |
| Reintegeration to normal living index (RNLI) | Stroke | Chronic | 57 | ? | ? | + | ? | ? | ? | + | ? |
| Stroke impact scale (SIS) | Stroke | Chronic |  | ? | ? | ? | ? | ? | ? | + | ? |
| Subjective index of physical and social outcome (SIPSO) | Stroke | Chronic | 260 | ? | ? | ? | ? | ? | ? | ? | ? |
| Tse (2013) [29] | | | | | | | | | | | |
| Activity Cart Sort (ACS) | Stroke | Chronic |  | + | - | + | ? | ? | ? | + | ? |
| Assessment of Life Habits (LIFE-H) | Stroke | Chronic | 84 | + | ? | + | ? | ? | ? | + | ? |
| Frenchay Activities Index (FAI) | Stroke | Chronic |  | + | + | ? | + | ? | ? | + | ? |
| London Handicap scale (LHS) | Stroke | Chronic | 37 | + | + | + | ? | ? | ? | + | ? |
| Stroke impact scale (SIS) | Stroke | Sub-acute | 25 | + | + | + | ? | ? | ? | + | ? |
| Tyson (2009) [30] | | | | | | | | | | | |
| Ten Meter Walking Test | Stroke | Acute | 81 | ? | ? | ? | + | ? | ? | ? | ? |
| Ten Meter Walking Test | Stroke | Chronic | 22 | ? | ? | ? | + | ? | ? | ? | ? |
| Ten Meter Walking Test | Stroke | Chronic | 40 | ? | ? | ? | ? | ? | ? | + | ? |
| Ten Meter Walking Test | Stroke | Chronic | 60 | ? | ? | ? | + | + | ? | ? | ? |
| Ten Meter Walking Test | Stroke | Chronic | 19 | ? | ? | ? | ? | ? | ? | ? | + |
| Ten Meter Walking Test | Traumatic brain injury |  | 12 | ? | ? | ? | ? | + | ? | + | ? |
| Ten Meter Walking Test | Traumatic brain injury |  | 13 | ? | ? | ? | + | ? | ? | ? | ? |
| Twelve Meter Walking Test | Stroke | Acute | 18 | ? | ? | ? | + | + | ? | ? | ? |
| Two Meter Walking Test | Stroke | Chronic |  | ? | ? | + | + | ? | ? | ? | ? |
| Five Meter Walking Test | Stroke | Chronic | 61 | ? | ? | ? | ? | ? | ? | ? | + |
| Five Meter Walking Test | Stroke | Chronic | 35 | ? | ? | ? | + | + | ? | + | ? |
| Six Minute Walking Test | Stroke | Acute | 18 | ? | ? | ? | + | + | ? | ? | ? |
| Six Minute Walking Test | Stroke | Chronic | 37 | ? | ? | ? | + | ? | ? | + | ? |
| Six Minute Walking Test | Traumatic brain injury |  | 23 | ? | ? | ? | + | ? | ? | ? | ? |
| Six Minute Walking Test | Traumatic brain injury |  | 13 | ? | ? | ? | + | ? | ? | ? | ? |
| Community balance and mobility scale (CB&M) | Traumatic brain injury |  | 32 | ? | ? | ? | + | + | ? | + | ? |
| Functional Ambulation Category (FAC) | Stroke | Chronic | 31 | ? | ? | ? | ? | + | ? | + | ? |
| Functional Ambulation Category (FAC) | Stroke | Chronic | 55 | ? | ? | ? | + | + | ? | + | ? |
| Functional Ambulation Category (FAC) | Stroke | Sub-acute | 20 | ? | ? | ? | ? | ? | ? | + | ? |
| High Level Mobility Assessment (HiMAT) | Traumatic brain injury |  | 103 | ? | ? | ? | + | + | ? | + | ? |
| Modified Emory Functional Ambulation Profile (M-EFAM) | Stroke | Chronic | 28 | ? | ? | ? | ? | + | ? | + | ? |
| Modified Emory Functional Ambulation Profile (M-EFAM) | Stroke | Chronic | 26 | ? | ? | ? | + | + | ? | + | ? |
| Modified Emory Functional Ambulation Profile (M-EFAM) | Stroke | Chronic | 40 | ? | ? | ? | + | ? | ? | + | ? |
| Pens taped to feet | Traumatic brain injury |  | 12 | ? | ? | ? | ? | + | ? | + | ? |
| Rivermead mobility index (RMI) | Stroke | Chronic | 73 | ? | ? | ? | ? | ? | ? | + | + |
| Rivermead mobility index (RMI) | Stroke | Chronic | 38 | ? | ? | ? | ? | ? | ? | + | ? |
| Rivermead mobility index (RMI) | Traumatic brain injury |  | 20 | ? | ? | ? | ? | ? | ? | + | ? |
| StepWatch Activity Monitor or Step Activity Monitor (SAM) | Stroke | Chronic | 19 | ? | ? | ? | ? | ? | ? | + | ? |
| StepWatch Activity Monitor or Step Activity Monitor (SAM) | Stroke | Chronic | 17 | ? | ? | ? | + | ? | ? | ? | ? |
| Timed Up and Go test (TUG) | Stroke | Chronic | 11 | ? | ? | ? | + | ? | ? | + | ? |
| Timed Up and Go test (TUG) | Stroke | Chronic | 50 | ? | ? | ? | + | ? | ? | ? | ? |
| Van Bloemendaal (2012) [31] | | | | | | | | | | | |
| Ten Meter Walking Test | Stroke | Chronic | 50 | + | ? | + | ? | ? | + | ? | ? |
| Ten Meter Walking Test | Stroke | Chronic | 28 | + | ? | ? | ? | ? | ? | + | ? |
| Ten Meter Walking Test | Stroke | Chronic | 18 | + | ? | ? | ? | ? | + | ? | ? |
| Ten Meter Walking Test | Stroke | Chronic | 50 | + | ? | ? | + | ? | + | ? | ? |
| Ten Meter Walking Test | Stroke | Chronic | 20 | + | ? | + | ? | ? | ? | + | ? |
| Ten Meter Walking Test | Stroke | Sub-acute | 12 | + | ? | + | ? | ? | + | + | ? |
| Ten Meter Walking Test | Stroke | Sub-acute | 12 | + | ? | + | ? | ? | ? | ? | ? |
| Ten Meter Walking Test | Stroke | Sub-acute | 43 | + | ? | ? | ? | ? | ? | + | ? |
| Twelve Meter Walking Test | Stroke | Chronic | 18 | + | ? | ? | + | + | ? | ? | ? |
| Twelve Meter Walking Test | Stroke | Chronic | 25 | + | ? | ? | ? | ? | ? | + | ? |
| Two Meter Waling Test | Stroke | Chronic | 18 | + | ? | ? | + | + | ? | ? | ? |
| Three Hundred Metre Walking Test in Community | Stroke | Chronic | 28 | + | ? | + | ? | ? | ? | + | ? |
| Thirty Metre Comfortable Walking Test | Stroke | Chronic | 18 | + | ? | ? | ? | ? | ? | + | ? |
| Four Metre Comfortable Walking Test | Stroke | Chronic | 25 | + | ? | ? | ? | ? | ? | + | ? |
| Five Meter Walking Test | Stroke | Chronic | 35 | + | ? | + | ? | ? | ? | ? | ? |
| Six Minute Walking Test | Stroke | Chronic | 15 | + | ? | + | ? | ? | + | ? | ? |
| Six Minute Walking Test | Stroke | Chronic | 18 | + | ? | + | + | ? | ? | ? | ? |
| Six Minute Walking Test | Stroke | Chronic | 25 | + | ? | ? | ? | ? | ? | + | ? |
| Six Minute Walking Test | Stroke | Chronic | 12 | + | ? | + | ? | ? | ? | ? | ? |
| Six Minute Walking Test | Stroke | Sub-acute | 24 | + | ? | + | ? | ? | ? | + | ? |
| Six Minute Walking Test | Stroke | Sub-acute | 37 | + | ? | + | ? | ? | + | + | ? |
| Six Minute Walking Test | Stroke | Sub-acute | 83 | + | ? | + | ? | ? | + | ? | ? |
| Six Minute Walking Test | Stroke | Sub-acute | 45 | + | ? | + | + | ? | + | + | ? |
| Dynamic Gait Index (DGI) | Stroke | Chronic | 25 | + | ? | + | + | ? | ? | ? | ? |
| Dynamic Gait Index (DGI) | Stroke | Sub-acute | 45 | + | ? | + | ? | ? | ? | + | ? |
| Footswitches | Stroke | Sub-acute | 25 | + | ? | + | ? | ? | + | + | ? |
| Functional Ambulation Category (FAC) | Stroke | Chronic | 25 | + | ? | + | ? | ? | ? | ? | ? |
| Functional Ambulation Category (FAC) | Stroke | Sub-acute | 55 | + | ? | + | ? | ? | ? | + | ? |
| Functional Ambulation Classification Hospital (FACHS) | Stroke | Chronic | 31 | + | ? | ? | ? | ? | ? | + | ? |
| Functional Gait Assessment (FGA) | Stroke | Chronic | 45 | + | ? | ? | + | + | ? | ? | ? |
| Pedometers | Stroke | Chronic |  | + | ? | ? | ? | ? | ? | ? | ? |
| Ambulatory Monitoring (AM Accelerometer) | Stroke | Chronic | 25 | + | ? | ? | ? | ? | ? | + | ? |
| Van Peppen (2007) [32] | | | | | | | | | | | |
| Ten Meter Waling Test | Stroke | Chronic |  | ? | ? | ? | + | + | ? | + | ? |
| Barthel Index (BI) | Stroke | Chronic |  | ? | ? | + | + | ? | ? | + | ? |
| Berg Balance Scale (BBS) | Stroke | Chronic |  | ? | ? | + | + | ? | ? | + | ? |
| Frenchay Activities Index (FAI) | Stroke | Chronic |  | ? | ? | + | ? | ? | ? | + | ? |
| Functional Ambulation Category (FAC) | Stroke | Chronic |  | ? | ? | ? | + | + | ? | + | ? |
| Motricity index (MI) | Stroke | Chronic |  | ? | ? | + | + | ? | ? | + | ? |
| Trunk Control Test | Stroke | Chronic |  | ? | ? | ? | + | + | ? | + | ? |
| Velstra (2011) [33] | | | | | | | | | | | |
| Action Research Arm test | Stroke | Chronic | 53 | ? | ? | + | + | ? | ? | ? | - |
| Action Research Arm test (ARAT) | Stroke | Chronic | 40 | + | + | + | + | ? | ? | ? | - |
| Fugl-Meyer Assessment (FMA) | Stroke | Chronic | 377 | ? | ? | + | + | ? | ? | ? | ? |
| Functional Independence measure (FIM) | Stroke | Chronic | 18 | + | ? | ? | - | ? | ? | ? | - |
| Grasp-Release test | Stroke | Chronic | 60 | ? | ? | ? | ? | ? | ? | ? | - |
| Grasp-Release test | Stroke | Chronic | 12 | ? | + | ? | ? | ? | ? | ? | + |
| Jebsen Hand Function Test | Stroke | Chronic | 33 | ? | ? | ? | ? | ? | ? | ? | - |
| Medical Outcomes Study 36-Item Short Form Health Survey (SF-36) | Stroke | Chronic | 19 | + | ? | ? | + | ? | ? | ? | - |
| Modified Ashworth scale | Stroke | Acute | 36 | ? | ? | ? | + | ? | ? | ? | ? |
| Motor Activity Log | Stroke | Chronic |  | + | + | - | ? | ? | ? | ? | - |
| Quadriplegia Index of Function | Stroke | Chronic | 60 | ? | ? | + | ? | ? | ? | ? | - |
| Van Lieshout Test Short Form | Stroke | Chronic | 60 | ? | ? | ? | ? | ? | ? | ? | - |
| Van Lieshout Test Short Form | Stroke | Chronic | 30 | ? | ? | ? | - | ? | ? | ? | - |
| Wolf Motor Function Test (WMFT) | Stroke | Chronic | 24 | ? | + | + | + | ? | ? | ? | ? |
| Verheyden (2006) [34] | | | | | | | | | | | |
| Trunk Control test | Stroke | Chronic | 20 | ? | + | ? | ? | - | ? | + | ? |
| Trunk Impairment Scale | Stroke | Chronic |  | + | + | ? | + | + | + | + | ? |
| Trunk Impairment Scale | Stroke | Chronic | 73 | ? | + | ? | ? | + | ? | ? | + |
| Wilde (2010) [35] | | | | | | | | | | | |
| EuroQol Qulaity of life scale-EQ5D | Traumatic brain injury |  | 86 | ? | ? | + | ? | ? | ? | ? | ? |
| Functional Independence measure (FIM) | Traumatic brain injury |  | 332 | + | ? | + | ? | ? | ? | ? | ? |
| Functional Independence measure (FIM) | Traumatic brain injury |  |  | ? | ? | ? | + | ? | ? | ? | ? |
| Grooved Pegboard Test (GPT) | Traumatic brain injury |  |  | ? | ? | + | ? | ? | ? | - | ? |
| Mayo-Portland Adaptability Inventory (MPAI-4) | Traumatic brain injury |  | 339 | ? | + | ? | + | ? | ? | + | ? |
| Medical Outcomes Study 36-Item Short Form Health Survey (SF-36) | Traumatic brain injury |  |  | ? | + | ? | ? | ? | ? | + | ? |
| Satisfaction With Life Scale (SWLS) | Traumatic brain injury |  |  | + | ? | + | ? | ? | ? | ? | ? |

**References**

1. Ashford, S., Slade, M., Malaprade, F., & Turner-Stokes, L. (2008). Evaluation of functional outcome measures for the hemiparetic upper limb: a systematic review. *Journal of rehabilitation medicine, 40*(10), 787-795.
2. Ashford, S., Brown, S., & Turner-Stokes, L. (2015). Systematic review of patient-reported outcome measures for functional performance in the lower limb. *Journal of rehabilitation medicine, 47*(1), 9-17.
3. Baker, K., Cano, S. J., & Playford, E. D. (2011). Outcome measurement in stroke: a scale selection strategy. *Stroke, 42*(6), 1787-1794.
4. Barak, S., & Duncan, P. W. (2006). Issues in selecting outcome measures to assess functional recovery after stroke. *NeuroRx, 3*(4), 505-524.
5. Connell, L. A., & Tyson, S. F. (2012). Clinical reality of measuring upper-limb ability in neurologic conditions: a systematic review. *Archives of physical medicine and rehabilitation, 93*(2), 221-228.
6. Croarkin, E., Danoff, J., & Barnes, C. (2004). Evidence-based rating of upper-extremity motor function tests used for people following a stroke. *Physical therapy, 84*(1), 62-74.
7. Fini, N. A., Holland, A. E., Keating, J., Simek, J., & Bernhardt, J. (2015). How is physical activity monitored in people following stroke? *Disability and Rehabilitation, 37*(19), 1717-1731.
8. Gebruers, N., Vanroy, C., Truijen, S., Engelborghs, S., & De Deyn, P. P. (2010). Monitoring of physical activity after stroke: a systematic review of accelerometry-based measures. *Archives of physical medicine and rehabilitation, 91*(2), 288-297.
9. Geroin, C., Mazzoleni, S., Smania, N., Gandolfi, M., Bonaiuti, D., Gasperini, G., et al. (2013). Systematic review of outcome measures of walking training using electromechanical and robotic devices in patients with stroke. *Journal of rehabilitation medicine, 45*(10), 987-996.
10. Gor-García-Fogeda, M. D., Molina-Rueda, F., Cuesta-Gómez, A., Carratalá-Tejada, M., Alguacil-Diego, I. M., & Miangolarra-Page, J. C. (2014). Scales to assess gross motor function in stroke patients: a systematic review. *Archives of physical medicine and rehabilitation, 95*(6), 1174-1183.
11. Hong, I., & Bonilha, H. S. (2017). Psychometric properties of upper extremity outcome measures validated by Rasch analysis: a systematic review. *International Journal of Rehabilitation Research, 40*(1), 1-10.
12. Lemmens, R. J., Timmermans, A. A., Janssen-Potten, Y. J., Smeets, R. J., & Seelen, H. A. (2012). Valid and reliable instruments for arm-hand assessment at ICF activity level in persons with hemiplegia: a systematic review. *BMC neurology, 12*(1), 21.
13. Martins, J. C., Aguiar, L. T., Nadeau, S., Scianni, A. A., Teixeira-Salmela, L. F., & Faria, C. D. C. D. M. (2019). Measurement properties of self-report physical activity assessment tools for patients with stroke: a systematic review. *Brazilian journal of physical therapy, 23*(6), 476-490.
14. Oczkowski, C., & O'Donnell, M. (2010). Reliability of proxy respondents for patients with stroke: a systematic review. *Journal of Stroke and Cerebrovascular Diseases, 19*(5), 410-416.
15. Pearson, O. R., Busse, M., Van Deursen, R. W. M., & Wiles, C. M. (2004). Quantification of walking mobility in neurological disorders. *Qjm, 97*(8), 463-475.
16. Pollock, C., Eng, J., & Garland, S. (2011). Clinical measurement of walking balance in people post stroke: a systematic review. *Clinical rehabilitation, 25*(8), 693-708.
17. Rowland, T. J., & Gustafsson, L. (2008). Assessments of upper limb ability following stroke: a review. *British Journal of Occupational Therapy, 71*(10), 427-437.
18. Salbach, N. M., O'brien, K. K., Brooks, D., Irvin, E., Martino, R., Takhar, P., et al. (2017). Considerations for the selection of time-limited walk tests poststroke: a systematic review of test protocols and measurement properties. *Journal of Neurologic Physical Therapy, 41*(1), 3-17.
19. Salter, K., Jutai, J., Teasell, R., Foley, N., Bitensky, J., & Bayley, M. (2005). Issues for selection of outcome measures in stroke rehabilitation: ICF activity. *Disability and Rehabilitation, 27*(6), 315-340.
20. Salter, K., Jutai, J., Teasell, R., Foley, N., & Bitensky, J. (2005). Issues for selection of outcome measures in stroke rehabilitation: ICF Body Functions. *Disability and Rehabilitation, 27*(4), 191-207.
21. Salter, K., Jutai, J., Teasell, R., Foley, N., Bitensky, J., & Bayley, M. (2005). Issues for selection of outcome measures in stroke rehabilitation: ICF Participation. *Disability and Rehabilitation, 27*(9), 507-528.
22. Scrivener, K., Sherrington, C., & Schurr, K. (2013). A systematic review of the responsiveness of lower limb physical performance measures in inpatient care after stroke. *BMC neurology, 13*(1), 4.
23. Silva, P. F., Quintino, L. F., Franco, J., & Faria, C. D. (2014). Measurement properties and feasibility of clinical tests to assess sit-to-stand/stand-to-sit tasks in subjects with neurological disease: a systematic review. *Brazilian journal of physical therapy, 18*(2), 99-110.
24. Simpson, L. A., & Eng, J. J. (2013). Functional recovery following stroke: capturing changes in upper-extremity function. *Neurorehabilitation and neural repair, 27*(3), 240-250.
25. Sivan, M., O'Connor, R. J., Makower, S., Levesley, M., & Bhakta, B. (2011). Systematic review of outcome measures used in the evaluation of robot-assisted upper limb exercise in stroke. *Journal of Rehabilitation Medicine, 43*(3), 181-189.
26. Sorrentino G., S. P., Solaro C., Rabini A., Cerri C., Ferriero G. (2018). Clinical measurement tools to assess trunk performance after stroke: a systematic review. *European journal of physical and rehabilitation medicine*.
27. Stevens, P. M. (2010). Clinimetric properties of timed walking events among patient populations commonly encountered in orthotic and prosthetic rehabilitation. *JPO: Journal of Prosthetics and Orthotics, 22*(1), 62-74.
28. Teale, E. A., & Young, J. B. (2010). A review of stroke outcome measures valid and reliable for administration by postal survey. *Reviews in Clinical Gerontology, 20*(4), 338-353.
29. Tse, T., Douglas, J., Lentin, P., & Carey, L. (2013). Measuring participation after stroke: a review of frequently used tools. *Archives of physical medicine and rehabilitation, 94*(1), 177-192.
30. Tyson, S., & Connell, L. (2009). The psychometric properties and clinical utility of measures of walking and mobility in neurological conditions: a systematic review. *Clinical rehabilitation, 23*(11), 1018-1033.
31. van Bloemendaal, M., van de Water, A. T., & van de Port, I. G. (2012). Walking tests for stroke survivors: a systematic review of their measurement properties. *Disability and Rehabilitation, 34*(26), 2207-2221.
32. Van Peppen, R. P., Hendriks, H., Van Meeteren, N. L., Helders, P. J., & Kwakkel, G. (2007). The development of a clinical practice stroke guideline for physiotherapists in The Netherlands: a systematic review of available evidence. *Disability and Rehabilitation, 29*(10), 767-783.
33. Velstra, I.-M., Ballert, C. S., & Cieza, A. (2011). A systematic literature review of outcome measures for upper extremity function using the international classification of functioning, disability, and health as reference. *PM&R, 3*(9), 846-860.
34. Verheyden, G., Nieuwboer, A., Van de Winckel, A., & De Weerdt, W. (2007). Clinical tools to measure trunk performance after stroke: a systematic review of the literature. *Clinical rehabilitation, 21*(5), 387-394.
35. Wilde, E. A., Whiteneck, G. G., Bogner, J., Bushnik, T., Cifu, D. X., Dikmen, S., et al. (2010). Recommendations for the use of common outcome measures in traumatic brain injury research. *Archives of physical medicine and rehabilitation, 91*(11), 1650-1660. e1617.
