# Supplementary material for: Quality of mobility measures among individuals with acquired brain injury: an umbrella review
Source: Qual Life Res. 2022 Mar 11;31(9):2567–99. doi: 10.1007/s11136-022-03103-4 (PMC9356944; doi:10.1007/s11136-022-03103-4)
Supplement: Supplementary file 6 — Supplementary file6 (DOCX 166 kb) [file 11136_2022_3103_MOESM6_ESM.docx]

**Mobility Measures among Individuals with Acquired Brain Injury: An Umbrella Review**

Rehab Alhasani, MSc,^1,2,6^ Cluadine Auger, PhD,^2,4,5^ Matheus de Paiva Azevedo, BSc,^1^ Sara Ahmed, PhD ^1-3^

**Author affiliations:**

1. School of Physical and Occupation Therapy, Faculty of Medicine, McGill University, Montreal, Canada
2. Centre de Recherche Interdisciplinaire en Réadaptation (CRIR), Montreal, Canada
3. Constance Lethbridge Rehabilitation Center, CIUSSS Centre Ouest de l’ile de Montreal, Montreal, Canada
4. School of Rehabilitation, Faculty of Medicine, University of Montreal, Montreal, Canada
5. Site Institut Universitaire sur la Réadaptation en Déficience Physique de Montréal (IURDPM), CIUSSS Centre-Sud-de-l’Ile-de-Montréal, Montréal, Canada
6. Department of Rehabilitation Sciences, Faculty of Health and Rehabilitation Sciences, Princess Nourah bint Abdulrahman University, Riyadh, Saudi Arabia

**Corresponding author:** Sara Ahmed, PhD, School of Physical and Occupation Therapy, Faculty of Medicine, McGill University, 3655 Sir William-Osler, Montreal, QC, Canada H3G 1Y6. Tel.: 514-398-4400 ext 00531.E-mail: sara.ahmed@mcgill.ca

**Supplementary file 6: Synthesis the results of measurement properties (Overall rating and modified-GRADE approach)**

| **Name of the measure** | **Type of population** | **Setting** | **Sample** | **Content validity** | **Internal consistency** | **Test-retest** | **Inter-rater** | **Intra-rater** | **Measurement error** | **Construct validity** | **Responsiveness** |
| --- | --- | --- | --- | --- | --- | --- | --- | --- | --- | --- | --- |
| 10MWT [1] | Stroke | Acute | 42 | ? | ? | ? | ? | ? | ? | ? | + |
| 10MWT [1] | Stroke | Acute | 50 | ? | ? | ? | ? | ? | ? | ? | + |
| 10MWT [2] | Stroke | Acute | 81 | ? | ? | ? | + | ? | ? | ? | ? |
| **Overall rating** | **Stroke** | **Acute** |  | ? | ? | ? | + | ? | ? | ? | + |
| **Quality of evidence** | **Stroke** | **Acute** |  | Inconsistent | Inconsistent | Inconsistent | High | Inconsistent | Inconsistent | Inconsistent | High |
| 10MWT [3] | Stroke | Chronic |  | ? | ? | + | + | ? | ? | - | + |
| 10MWT [4] | Stroke | Chronic |  | ? | ? | + | ? | ? | ? | ? | + |
| 10MWT [2] | Stroke | Chronic | 22 | ? | ? | ? | + | ? | ? | ? | ? |
| 10MWT [2] | Stroke | Chronic | 40 | ? | ? | ? | ? | ? | ? | + | ? |
| 10MWT [2] | Stroke | Chronic | 60 | ? | ? | ? | + | + | ? | ? | ? |
| 10MWT [2] | Stroke | Chronic | 19 | ? | ? | ? | ? | ? | ? | ? | + |
| 10MWT [5] | Stroke | Chronic | 50 | + | ? | + | ? | ? | + | ? | ? |
| 10MWT [5] | Stroke | Chronic | 28 | + | ? | ? | ? | ? | ? | + | ? |
| 10MWT [5] | Stroke | Chronic | 18 | + | ? | ? | ? | ? | + | ? | ? |
| 10MWT [5] | Stroke | Chronic | 50 | + | ? | ? | + | ? | + | ? | ? |
| 10MWT [5] | Stroke | Chronic | 20 | + | ? | + | ? | ? | ? | + | ? |
| 10MWT [6] | Stroke | Chronic |  | ? | ? | ? | + | + | ? | + | ? |
| **Overall rating** | **Stroke** | **Chronic** |  | + | ? | + | + | ? | + | + | + |
| **Quality of evidence** | **Stroke** | **Chronic** |  | High | Inconsistent | High | High | Inconsistent | Moderate (-1) | High | Low (-2) |
| 10MWT [7] | Stroke | Sub-acute |  | ? | ? | + | ? | ? | ? | - | + |
| 10MWT [4] | Stroke | Sub-acute |  | ? | ? | + | ? | ? | ? | ? | ? |
| 10MWT [5] | Stroke | Sub-acute | 12 | + | ? | + | ? | ? | + | + | ? |
| 10MWT [5] | Stroke | Sub-acute | 12 | + | ? | + | ? | ? | ? | ? | ? |
| 10MWT [5] | Stroke | Sub-acute | 43 | + | ? | ? | ? | ? | ? | + | ? |
| **Overall rating** | **Stroke** | **Sub-acute** |  | + | ? | + | ? | ? | + | + | + |
| **Quality of evidence** | **Stroke** | **Sub-acute** |  | Moderate (-1) | Inconsistent | Low (-2) | Inconsistent | Inconsistent | Low (-2) | Low (-2) | Low (-2) |
| 10MWT [4] | TBI |  | 94 | ? | ? | + | ? | ? | ? | ? | ? |
| 10MWT [2] | TBI |  | 12 | ? | ? | ? | ? | + | ? | + | ? |
| 10MWT [2] | TBI |  | 13 | ? | ? | + | + | ? | ? | ? | ? |
| **10MWT** | **TBI** |  |  | ? | ? | + | + | + | ? | + | ? |
| **10MWT** | **TBI** |  |  | Inconsistent | Inconsistent | High | Low (-2) | Low (-2) | Inconsistent | Low (-2) | inconsistent |
| 12MWT [2] | Stroke | Acute | 18 | ? | ? | ? | + | + | ? | ? | ? |
| **Overall rating** | **Stroke** | **Acute** |  | ? | ? | ? | + | + | ? | ? | ? |
| **Quality of evidence** | **Stroke** | **Acute** |  | Inconsistent | Inconsistent | Inconsistent | Low (-2) | Low (-2) | Inconsistent | Inconsistent | Inconsistent |
| 12MWT [8] | Stroke | Chronic | 25 | + | ? | ? | ? | ? | ? | + | ? |
| 12MWT [1] | Stroke | Chronic | 18 | ? | ? | ? | ? | ? | ? | ? | + |
| 12MWT [5] | Stroke | Chronic | 18 | + | ? | ? | + | + | ? | ? | ? |
| 12MWT [5] | Stroke | Chronic | 25 | + | ? | ? | ? | ? | ? | + | ? |
| **Overall rating** | **Stroke** | **Chronic** |  | + | ? | ? | + | + | ? | + | + |
| **Quality of evidence** | **Stroke** | **Chronic** |  | Moderate (-1) | Inconsistent | Inconsistent | Low (-2) | Low (-2) | Inconsistent | Moderate (-1) | Low (-2) |
| 12MWT [8] | Stroke | Sub-acute | 20 | + | ? | ? | ? | + | ? | + | ? |
| **Overall rating** | **Stroke** | **Sub-acute** |  | + | ? | ? | ? | + | ? | + | ? |
| **Quality of evidence** | **Stroke** | **Sub-acute** |  | Low (-2) | Inconsistent | Inconsistent | Inconsistent | Low (-2) | Inconsistent | Low (-2) | Inconsistent |
| 2MWT [1] | Stroke | Acute | 18 | ? | ? | + | + | ? | ? | ? | + |
| **2MWT** | **Stroke** | **Acute** |  | ? | ? | + | + | ? | ? | ? | + |
| **2MWT** | **Stroke** | **Acute** |  | Inconsistent | Inconsistent | Low (-2) | Low (-2) | Inconsistent | Inconsistent | Inconsistent | Low (-2) |
| 2MWT [8] | Stroke | Chronic | 61 | + | ? | ? | ? | + | + | ? | ? |
| 2MWT [8] | Stroke | Chronic | 12 | + | ? | ? | ? | + | + | ? | ? |
| 2MWT [8] | Stroke | Chronic | 32 | + | ? | ? | ? | + | + | ? | ? |
| 2MWT [8] | Stroke | Chronic | 17 | + | ? | ? | ? | + | + | ? | ? |
| 2MWT [2] | Stroke | Chronic |  | ? | ? | + | + | ? | ? | ? | ? |
| 2MWT [5] | Stroke | Chronic | 18 | + | ? | ? | + | + | ? | ? | ? |
| **Overall rating** | **Stroke** | **Chronic** |  | + | ? | + | + | + | + | ? | ? |
| **Quality of evidence** | **Stroke** | **Chronic** |  | High | Inconsistent | Low (-2) | Low (-2) | High | High | Inconsistent | Inconsistent |
| 5MWT [8] | Stroke | Chronic | 9 | + | ? | ? | ? | + | + | ? | ? |
| 5MWT [8] | Stroke | Chronic | 20 | + | ? | ? | ? | ? | ? | + | ? |
| 5MWT [8] | Stroke | Chronic | 10 | + | ? | ? | ? | ? | ? | + | ? |
| 5MWT [4] | Stroke | Chronic |  | ? | ? | + | ? | ? | ? | ? | ? |
| 5MWT [2] | Stroke | Chronic | 61 | ? | ? | ? | ? | ? | ? | ? | + |
| 5MWT [2] | Stroke | Chronic | 35 | ? | ? | ? | + | + | ? | + | ? |
| 5MWT [5] | Stroke | Chronic | 35 | + | ? | + | ? | ? | ? | ? | ? |
| **Overall rating** | **Stroke** | **Chronic** |  | + | ? | + | ? | + | + | + | + |
| **Quality of evidence** | **Stroke** | **Chronic** |  | Moderate (-1) | Inconsistent | Low (-2) | Low (-2) | Low (-2) | Low (-2) | Moderate (-1) | Moderate (-1) |
| 6MWT [8] | Stroke | Acute | 37 | + | ? | ? | ? | + | + | ? | ? |
| 6MWT [8] | Stroke | Acute | 24 | + | ? | ? | ? | + | + | ? | ? |
| 6MWT [8] | Stroke | Acute | 13 | + | ? | ? | ? | + | + | ? | ? |
| 6MWT [8] | Stroke | Acute | 41 | + | ? | ? | ? | ? | ? | + | ? |
| 6MWT [8] | Stroke | Acute | 30 | + | ? | ? | ? | ? | ? | + | ? |
| 6MWT [2] | Stroke | Acute | 18 | ? | ? | ? | + | + | ? | ? | ? |
| **Overall rating** | **Stroke** | **Acute** |  | + | ? | ? | + | + | + | + | ? |
| **Quality of evidence** | **Stroke** | **Acute** |  | High | Inconsistent | Inconsistent | Low (-2) | Moderate (-1) | Moderate (-1) | Moderate (-1) | Inconsistent |
| 6MWT [3] | Stroke | Chronic |  | ? | ? | ? | + | + | ? | - | ? |
| 6MWT [8] | Stroke | Chronic | 83 | + | ? | ? | ? | + | ? | ? | ? |
| 6MWT [8] | Stroke | Chronic | 12 | + | ? | ? | ? | + | + | ? | ? |
| 6MWT [8] | Stroke | Chronic | 10 | + | ? | ? | ? | + | ? | ? | ? |
| 6MWT [8] | Stroke | Chronic | 50 | + | ? | ? | ? | + | + | ? | ? |
| 6MWT [8] | Stroke | Chronic | 27 | + | ? | ? | ? | + | + | ? | ? |
| 6MWT [8] | Stroke | Chronic | 27 | + | ? | ? | ? | ? | ? | + | ? |
| 6MWT [8] | Stroke | Chronic | 36 | + | ? | ? | ? | ? | ? | + | ? |
| 6MWT [8] | Stroke | Chronic | 34 | + | ? | ? | ? | ? | ? | + | ? |
| 6MWT [8] | Stroke | Chronic | 50 | + | ? | ? | ? | ? | ? | + | ? |
| 6MWT [8] | Stroke | Chronic | 61 | + | ? | ? | ? | ? | ? | + | ? |
| 6MWT [8] | Stroke | Chronic | 64 | + | ? | ? | ? | ? | ? | + | ? |
| 6MWT [8] | Stroke | Chronic | 12 | + | ? | ? | ? | ? | ? | + | ? |
| 6MWT [8] | Stroke | Chronic | 34 | + | ? | ? | ? | ? | ? | + | ? |
| 6MWT [8] | Stroke | Chronic | 25 | + | ? | ? | ? | ? | ? | + | ? |
| 6MWT [8] | Stroke | Chronic | 48 | + | ? | ? | ? | ? | ? | + | ? |
| 6MWT [8] | Stroke | Chronic | 30 | + | ? | ? | ? | ? | ? | + | ? |
| 6MWT [8] | Stroke | Chronic | 21 | + | ? | ? | ? | ? | ? | + | ? |
| 6MWT [8] | Stroke | Chronic | 40 | + | ? | ? | ? | ? | ? | + | ? |
| 6MWT [8] | Stroke | Chronic | 77 | + | ? | ? | ? | ? | ? | + | ? |
| 6MWT [8] | Stroke | Chronic | 40 | + | ? | ? | ? | ? | ? | + | ? |
| 6MWT [8] | Stroke | Chronic | 49 | + | ? | ? | ? | ? | ? | + | ? |
| 6MWT [8] | Stroke | Chronic | 17 | + | ? | ? | ? | ? | ? | + | ? |
| 6MWT [8] | Stroke | Chronic | 42 | + | ? | ? | ? | ? | ? | + | ? |
| 6MWT [8] | Stroke | Chronic | 50 | + | ? | ? | ? | ? | ? | + | ? |
| 6MWT [8] | Stroke | Chronic | 68 | + | ? | ? | ? | ? | ? | + | ? |
| 6MWT [8] | Stroke | Chronic | 25 | + | ? | ? | ? | ? | ? | + | ? |
| 6MWT [8] | Stroke | Chronic | 25 | + | ? | ? | ? | ? | ? | + | ? |
| 6MWT [8] | Stroke | Chronic | 30 | + | ? | ? | ? | ? | ? | + | ? |
| 6MWT [8] | Stroke | Chronic | 77 | + | ? | ? | ? | ? | ? | + | ? |
| 6MWT [1] | Stroke | Chronic | 18 | ? | ? | ? | ? | ? | ? | ? | + |
| 6MWT [4] | Stroke | Chronic |  | ? | ? | + | + | ? | ? | ? | ? |
| 6MWT [2] | Stroke | Chronic | 37 | ? | ? | ? | + | ? | ? | + | ? |
| 6MWT [5] | Stroke | Chronic | 15 | + | ? | + | ? | ? | + | ? | ? |
| 6MWT [5] | Stroke | Chronic | 18 | + | ? | + | + | ? | ? | ? | ? |
| 6MWT [5] | Stroke | Chronic | 25 | + | ? | ? | ? | ? | ? | + | ? |
| 6MWT [5] | Stroke | Chronic | 12 | + | ? | + | ? | ? | ? | ? | ? |
| **Overall rating** | **Stroke** | **Chronic** |  | + | ? | + | + | + | + | + | + |
| **Quality of evidence** | **Stroke** | **Chronic** |  | High | Inconsistent | High | High | High | High | High | Low (-2) |
| 6MWT [5] | Stroke | Sub-acute | 24 | + | ? | + | ? | ? | ? | + | ? |
| 6MWT [8] | Stroke | Sub-acute | 50 | + | ? | ? | ? | ? | ? | + | ? |
| 6MWT [8] | Stroke | Sub-acute | 63 | + | ? | ? | ? | ? | ? | + | ? |
| 6MWT [8] | Stroke | Sub-acute | 48 | + | ? | ? | ? | ? | ? | + | ? |
| 6MWT [8] | Stroke | Sub-acute | 25 | + | ? | ? | ? | ? | ? | + | ? |
| 6MWT [5] | Stroke | Sub-acute | 37 | + | ? | + | ? | ? | + | + | ? |
| 6MWT [5] | Stroke | Sub-acute | 83 | + | ? | + | ? | ? | + | ? | ? |
| 6MWT [5] | Stroke | Sub-acute | 45 | + | ? | + | + | ? | + | + | ? |
| 6MWT [7] | Stroke | Sub-acute |  | ? | ? | + | ? | ? | + | + | ? |
| **Overall rating** | **Stroke** | **Sub-acute** |  | + | ? | + | + | ? | + | + | ? |
| **Quality of evidence** | **Stroke** | **Sub-acute** |  | High | Inconsistent | High | Low (-2) | Inconsistent | High | High | Inconsistent |
| 6MWT [4] | TBI |  | 36 | ? | ? | + | ? | ? | ? | ? | ? |
| 6MWT [2] | TBI |  | 23 | ? | ? | ? | + | ? | ? | ? | ? |
| 6MWT [2] | TBI |  | 13 | ? | ? | ? | + | ? | ? | ? | ? |
| **Overall rating** | **TBI** |  |  | ? | ? | + | + | ? | ? | ? | ? |
| **Quality of evidence** | **TBI** |  |  | Inconsistent | Inconsistent | Low (-2) | Low (-2) | Inconsistent | Inconsistent | Inconsistent | Inconsistent |
| ABILHAND [9] | Stroke | Chronic |  | + | + | + | + | ? | ? | + | + |
| ABILHAND [10] | Stroke | Chronic |  | ? | ? | + | ? | ? | ? | + | + |
| ABILHAND [11] | stroke | Chronic | 103 | ? | ? | ? | ? | ? | ? | + | ? |
| ABILHAND [12] | Stroke | Chronic | 103 | + | ? | + | ? | ? | ? | + | ? |
| ABILHAND [13] | Stroke | Chronic |  | + | ? | ? | + | ? | ? | ? | ? |
| ABILHAND [14] | Stroke | Chronic |  | + | ? | + | ? | ? | ? | - | ? |
| ABILHAND [15] | Stroke | Chronic |  | ? | ? | ? | ? | ? | ? | ? | + |
| ABILHAND [16] | Stroke | Chronic |  | ? | ? | + | ? | ? | ? | - | ? |
| **Overall rating** | **Stroke** | **Chronic** |  | + | + | + | + | ? | ? | + | + |
| **Quality of evidence** | **Stroke** | **Chronic** |  | High | Low (-2) | High | Low (-2 ) | Inconsistent | Inconsistent | High | Low (-2) |
| Action Research Arm test (ARAT) [17] | Stroke | Chronic |  | ? | ? | ? | + | ? | ? | + | ? |
| Action Research Arm test (ARAT) [10] | Stroke | Chronic |  | + | - | ? | ? | ? | ? | - | - |
| Action Research Arm test (ARAT) [11] | stroke | Chronic |  | ? | ? | + | + | ? | ? | + | ? |
| Action Research Arm test (ARAT) [12] | Stroke | Chronic | 191 | + | + | + | ? | ? | ? | + | ? |
| Action Research Arm test (ARAT) [12] | Stroke | Chronic | 351 | + | ? | ? | ? | ? | ? | + | ? |
| Action Research Arm test (ARAT) [14] | Stroke | Chronic |  | ? | + | + | + | ? | ? | + | + |
| Action Research Arm test (ARAT) [15] | Stroke | Chronic |  | ? | ? | ? | ? | ? | ? | + | ? |
| Action Research Arm test [16] | Stroke | Chronic |  | ? | ? | + | + | ? | ? | + | + |
| Action Research Arm test (ARAT) [18] | Stroke | Chronic | 40 | + | + | + | + | ? | ? | ? | - |
| Action Research Arm test (ARAT) [13] | Stroke | Chronic |  | ? | ? | ? | ? | ? | ? | ? | ? |
| Action Research Arm test (ARAT) [13] | Stroke | Chronic |  | ? | ? | ? | ? | ? | ? | ? | ? |
| Action Research Arm test [18] | Stroke | Chronic | 53 | ? | ? | + | + | ? | ? | ? | - |
| **Overall rating** | **Stroke** | **Chronic** |  | + | + | + | + | ? | ? | + | + |
| **Quality of evidence** | **Stroke** | **Chronic** |  | High | High | High | Low (-2) | Inconsistent | Inconsistent | High | Low (-2) |
| Activity Cart Sort (ACS) [19] | Stroke | Chronic | 29 | ? | ? | ? | ? | ? | ? | + | ? |
| Activity Cart Sort (ACS) [20] | Stroke | Chronic |  | + | - | + | ? | ? | ? | + | ? |
| Activity Cart Sort (ACS) [19] | Stroke | Chronic | 60 | ? | + | + | ? | ? | ? | + | ? |
| **Overall rating** | **Stroke** | **Chronic** |  | + | + | + | ? | ? | ? | + | ? |
| **Quality of evidence** | **Stroke** | **Chronic** |  | Low (-2) | Moderate (-1) | Moderate (-1) | Inconsistent | Inconsistent | Inconsistent | Moderate (-1) | Inconsistent |
| Actiwatch [21] | Stroke | Acute | 52 | ? | ? | ? | ? | ? | ? | + | ? |
| **Overall rating** | **Stroke** | **Acute** |  | ? | ? | ? | ? | ? | ? | + | ? |
| **Quality of evidence** | **Stroke** | **Acute** |  | Inconsistent | Inconsistent | Inconsistent | Inconsistent | Inconsistent | Inconsistent | Moderate (-1) | Inconsistent |
| Actiwatch [21] | Stroke | Chronic | 11 | ? | ? | ? | ? | ? | ? | + | ? |
| **Overall rating** | **Stroke** | **Chronic** |  | ? | ? | ? | ? | ? | ? | + | ? |
| **Quality of evidence** | **Stroke** | **Chronic** |  | Inconsistent | Inconsistent | Inconsistent | Inconsistent | Inconsistent | Inconsistent | Low (-2) | Inconsistent |
| Ambulatory Monitoring (AM Accelerometer) [21] | Stroke | Acute | 43 | ? | ? | ? | ? | ? | ? | + | ? |
| **Overall rating** | **Stroke** | **Acute** |  | ? | ? | ? | ? | ? | ? | + | ? |
| **Quality of evidence** | **Stroke** | **Acute** |  | Inconsistent | Inconsistent | Inconsistent | Inconsistent | Inconsistent | Inconsistent | Low (-2) | Inconsistent |
| Ambulatory Monitoring (AM Accelerometer) [5] | Stroke | Chronic | 25 | + | ? | ? | ? | ? | ? | + | ? |
| **Overall rating** | **Stroke** | **Chronic** |  | + | ? | ? | ? | ? | ? | + | ? |
| **Quality of evidence** | **Stroke** | **Chronic** |  | Low (-2) | Inconsistent | Inconsistent | Inconsistent | Inconsistent | Inconsistent | Low (-2) | Inconsistent |
| Arm Motor Ability Test (AMAT) [14] | Stroke | Sub-acute | 32 | ? | + | + | - | ? | ? | - | + |
| **Overall rating** | **Stroke** | **Sub-acute** |  | ? | + | + | - | ? | ? | - | + |
| **Quality of evidence** | **Stroke** | **Sub-acute** |  | Inconsistent | Low (-2) | Low (-2) | Low (-2) | Inconsistent | Inconsistent | Low (-2) | Low (-2) |
| Arm Motor Ability Test (AMAT) [12] | Stroke | Chronic | 36 | + | ? | ? | ? | ? | ? | ? | ? |
| Arm Motor Ability Test (AMAT) [13] | Stroke | Chronic |  | ? | ? | ? | ? | ? | ? | ? | ? |
| Arm Motor Ability Test (AMAT) [16] | Stroke | Chronic |  | ? | ? | + | + | ? | ? | ? | ? |
| **Overall rating** | **Stroke** | **Chronic** |  | + | ? | + | + | ? | ? | ? | ? |
| **Quality of evidence** | **Stroke** | **Chronic** |  | Low (-2) | Inconsistent | Inconsistent | Low (-2) | Inconsistent | inconsistent | inconsistent | inconsistent |
| Barthel Index (BI) [22] | Stroke | Acute | 22 | ? | + | + | + | + | ? | + | + |
| **Overall rating** | **Stroke** | **Acute** |  | ? | + | + | + | + | ? | + | + |
| **Quality of evidence** | **Stroke** | **Acute** |  | inconsistent | Low (-2) | Low (-2) | Low (-2) | Low (-2) | Inconsistent | Low (-2) | Low (-2) |
| Barthel Index (BI) [7] | Stroke | Chronic | 30 | ? | ? | + | ? | ? | ? | + | + |
| Barthel Index (BI) [6] | Stroke | Chronic |  | ? | ? | + | + | ? | ? | + | ? |
| Barthel Index (BI) [23] | Stroke | Chronic |  | ? | ? | + | ? | + | ? | + | + |
| Barthel Index (BI) [16] | Stroke | Chronic | 167 | ? | ? | + | + | ? | ? | - | + |
| **Overall rating** | **Stroke** | **Chronic** |  | ? | ? | + | + | + | ? | + | + |
| **Quality of evidence** | **Stroke** | **Chronic** |  | Inconsistent | Inconsistent | High | High | Low (-2) | Inconsistent | High | High |
| Beck Depression Inventory (BDI) [7] | Stroke | Acute | 202 | ? | ? | + | ? | ? | ? | + | - |
| **Overall rating** | **Stroke** | **Acute** |  | ? | ? | + | ? | ? | ? | + | - |
| **Quality of evidence** | **Stroke** | **Acute** |  | Inconsistent | Inconsistent | High | Inconsistent | Inconsistent | Inconsistent | High | Moderate (-1) |
| Beck Depression Inventory (BDI) [24] | Stroke | Chronic | 202 | + | + | - | ? | ? | ? | + | + |
| **Overall rating** | **Stroke** | **Chronic** |  | + | + | - | ? | ? | ? | + | + |
| **Quality of evidence** | **Stroke** | **Chronic** |  | High | High | Moderate (-1) | Inconsistent | Inconsistent | Inconsistent | High | High |
| Berg Balance Scale (BBS) [22] | Stroke | Acute |  | ? | + | + | + | ? | ? | + | + |
| Berg Balance Scale (BBS) [1] | Stroke | Acute | 50 | ? | ? | ? | ? | ? | ? | ? | + |
| Berg Balance Scale (BBS) [1] | Stroke | Acute | 110 | ? | ? | ? | ? | ? | ? | ? | + |
| Berg Balance Scale (BBS) [1] | Stroke | Acute | 93 | ? | ? | ? | ? | ? | ? | ? | + |
| Berg Balance Scale (BBS) [1] | Stroke | Acute | 80 | ? | ? | ? | ? | ? | ? | ? | + |
| Berg Balance Scale (BBS) [1] | Stroke | Acute | 93 | ? | ? | ? | ? | ? | ? | ? | + |
| Berg Balance Scale (BBS) [1] | Stroke | Acute | 80 | ? | ? | ? | ? | ? | ? | ? | + |
| Berg Balance Scale (BBS) [1] | Stroke | Acute | 60 | ? | ? | ? | ? | ? | ? | ? | + |
| **Overall rating** | **Stroke** | **Acute** |  | ? | + | + | + | ? | ? | + | + |
| **Quality of evidence** | **Stroke** | **Acute** |  | Inconsistent | Low (-2) | Low (-2) | Low (-2) | Inconsistent | inconsistent | Low (-2) | High |
| Berg Balance Scale (BBS) [7] | Stroke | Chronic | 70 | ? | ? | + | ? | ? | ? | + | + |
| Berg Balance Scale (BBS) [6] | Stroke | Chronic |  | ? | ? | + | + | ? | ? | + | ? |
| **Overall rating** | **Stroke** | **Chronic** |  | ? | ? | + | + | ? | ? | + | + |
| **Quality of evidence** | **Stroke** | **Chronic** |  | inconsistent | inconsistent | Moderate (-1) | Low (-2) | Inconsistent | inconsistent | Moderate (-1) | Moderate (-1) |
| Berg Balance Scale (BBS) [3] | Stroke | Sub-acute | 15 | ? | ? | + | + | ? | ? | - | + |
| **Overall rating** | **Stroke** | **Sub-acute** |  | ? | ? | + | + | ? | ? | - | + |
| **Quality of evidence** | **Stroke** | **Sub-acute** |  | inconsistent | inconsistent | Low (-2) | Low (-2) | Inconsistent | inconsistent | Low (-2) | Low (-2) |
| Berg Balance Scale three point (BBS-3P) [1] | Stroke | Acute | 202 | ? | ? | ? | ? | ? | ? | ? | + |
| Berg Balance Scale three point (BBS-3P) [1] | Stroke | Acute | 167 | ? | ? | ? | ? | ? | ? | ? | + |
| Berg Balance Scale three point (BBS-3P) [1] | Stroke | Acute | 167 | ? | ? | ? | ? | ? | ? | ? | + |
| **Overall rating** | **Stroke** | **Acute** |  | ? | ? | ? | ? | ? | ? | ? | + |
| **Quality of evidence** | **Stroke** | **Acute** |  | Inconsistent | Inconsistent | Inconsistent | Inconsistent | Inconsistent | Inconsistent | Inconsistent | High |
| Box and Block test [11] | Stroke | Chronic | 15 | ? | ? | + | + | + | ? | + | ? |
| Box and Block test [16] | Stroke | Chronic | 37 | ? | ? | + | ? | ? | ? | - | - |
| **Overall rating** | **Stroke** | **Chronic** |  | ? | ? | + | + | + | ? | + | - |
| **Quality of evidence** | **Stroke** | **Chronic** |  | Inconsistent | Inconsistent | Moderate (-1) | Low (-2) | Low (-2) | Inconsistent | Low (-2) | Low (-2) |
| Caltrac accelerometer [25] | Stroke | Chronic | 17 | ? | ? | - | ? | ? | ? | ? | ? |
| Caltrac accelerometer [21] | Stroke | Chronic | 27 | ? | ? | - | ? | ? | ? | ? | ? |
| **Overall rating** | **Stroke** | **Chronic** |  | ? | ? | - | ? | ? | ? | ? | ? |
| **Quality of evidence** | **Stroke** | **Chronic** |  | Inconsistent | Inconsistent | Low (-2) | Inconsistent | Inconsistent | Inconsistent | Inconsistent | Inconsistent |
| Chedoke Arm and Hand Inventory (CAHAI) [14] | Stroke | Chronic |  | + | + | + | - | ? | ? | + | + |
| Chedoke Arm and Hand Inventory (CAHAI) [13] | Stroke | Chronic |  | ? | ? | ? | ? | ? | ? | ? | ? |
| Chedoke Arm and Hand Inventory (CAHAI) [16] | Stroke | Chronic |  | ? | ? | ? | + | ? | ? | ? | + |
| Chedoke Arm and Hand Inventory (CAHAI) [10] | Stroke | Chronic | 109 | + | + | + | + | ? | ? | + | ? |
| **Overall rating** | **Stroke** | **Chronic** |  | + | + | + | + | ? | ? | + | + |
| **Quality of evidence** | **Stroke** | **Chronic** |  | High | High | High | High | Inconsistent | Inconsistent | High | Low (-2) |
| Chedoke McMaster Stroke assessment scale (CMSA) [7] | Stroke | Acute | 32 | ? | ? | + | ? | ? | ? | + | + |
| **Overall rating** | **Stroke** | **Acute** |  | ? | ? | + | ? | ? | ? | + | + |
| **Quality of evidence** | **Stroke** | **Acute** |  | Inconsistent | Inconsistent | Low (-2) | Inconsistent | Inconsistent | Inconsistent | Low (-2) | Low (-2) |
| Chedoke McMaster Stroke assessment scale (CMSA) [22] | Stroke | Chronic | 127 | ? | + | + | + | + | ? | + | + |
| Chedoke McMaster Stroke assessment scale (CMSA) [16] | Stroke | Chronic |  | ? | ? | ? | + | ? | ? | - | ? |
| Chedoke McMaster Stroke assessment scale (CMSA) [17] | Stroke | Chronic |  | ? | ? | ? | + | ? | ? | + | ? |
| **Overall rating** | **Stroke** | **Chronic** |  | ? | + | + | + | + | ? | - | + |
| **Quality of evidence** | **Stroke** | **Chronic** |  | Inconsistent | High | High | High | High | Inconsistent | High | High |
| Community balance and mobility scale (CB&M) [26] | Stroke | Chronic | 44 | ? | ? | ? | ? | ? | ? | + | + |
| **Overall rating** | **Stroke** | **Chronic** |  | ? | ? | ? | ? | ? | ? | + | + |
| **Quality of evidence** | **Stroke** | **Chronic** |  | Inconsistent | Inconsistent | Inconsistent | Inconsistent | Inconsistent | Inconsistent | Low (-2) | Low (-2) |
| Community balance and mobility scale (CB&M) [2] | TBI |  | 32 | ? | ? | ? | + | + | ? | + | ? |
| **Overall rating** | **TBI** |  |  | ? | ? | ? | + | + | ? | + | ? |
| **Quality of evidence** | **TBI** |  |  | Inconsistent | Inconsistent | Inconsistent | Low (-2) | Low (-2) | Inconsistent | Low (-2) | Inconsistent |
| Dynamic Gait Index (DGI) [26] | Stroke | Chronic |  | ? | ? | + | - | - | ? | ? | ? |
| Dynamic Gait Index (DGI) [5] | Stroke | Chronic | 25 | + | ? | + | + | ? | ? | ? | ? |
| **Overall rating** | **Stroke** | **Chronic** |  | + | ? | + | + | - | ? | ? | ? |
| **Quality of evidence** | **Stroke** | **Chronic** |  | Low (-2) | Inconsistent | Low (-2) | Low (-2) | Low (-2) | Inconsistent | Inconsistent | Inconsistent |
| Dynamic Gait Index (DGI) [5] | Stroke | Sub-acute | 45 | + | ? | + | ? | ? | ? | + | ? |
| **Overall rating** | **Stroke** | **Sub-acute** |  | + | ? | + | ? | ? | ? | + | ? |
| **Quality of evidence** | **Stroke** | **Sub-acute** |  | Low (-2) | Inconsistent | Low (-2) | Inconsistent | Inconsistent | Inconsistent | Low (-2) | Inconsistent |
| European Quality of life scale (EQ5D) [27] | TBI |  | 86 | ? | ? | + | ? | ? | ? | ? | ? |
| **Overall rating** | **TBI** |  |  | ? | ? | + | ? | ? | ? | ? | ? |
| **Quality of evidence** | **TBI** |  |  | Inconsistent | Inconsistent | Moderate (-1) | Inconsistent | Inconsistent | Inconsistent | Inconsistent | Inconsistent |
| European Qulaity of life scale (EQ5D) [16] | Stroke | Acute |  | ? | ? | + | ? | ? | ? | - | ? |
| **Overall rating** | **Stroke** | **Acute** |  | ? | ? | + | ? | ? | ? | - | ? |
| **Quality of evidence** | **Stroke** | **Acute** |  | Inconsistent | Inconsistent | Low (-2) | Inconsistent | Inconsistent | Inconsistent | Low (-2) | Inconsistent |
| European Qulaity of life scale (EQ5D) [7] | Stroke | Chronic |  | ? | ? | - | ? | ? | ? | - | + |
| European Qulaity of life scale (EQ5D) [28] | Stroke | Chronic | 15 | ? | ? | + | ? | ? | ? | ? | ? |
| European Qulaity of life scale (EQ5D) [29] | Stroke | Chronic |  | ? | ? | - | ? | ? | ? | + | ? |
| European Qulaity of life scale (EQ5D) [30] | Stroke | Chronic |  | ? | ? | + | ? | ? | ? | ? | ? |
| **Overall rating** | **Stroke** | **Chronic** |  | ? | ? | + | ? | ? | ? | + | + |
| **Quality of evidence** | **Stroke** | **Chronic** |  | Inconsistent | Inconsistent | Low (-2) | Inconsistent | Inconsistent | Inconsistent | Low (-2) | Low (-2) |
| Five times Sit to Stand test [31] | Stroke | Chronic | 19 | + | ? | + | ? | ? | + | ? | ? |
| Five times Sit to Stand test [31] | Stroke | Chronic | 12 | + | ? | + | + | + | ? | + | + |
| Five times Sit to Stand test [31] | Stroke | Chronic | 27 | + | ? | ? | ? | ? | ? | + | ? |
| **Overall rating** | **Stroke** | **Chronic** |  | + | ? | + | + | + | + | + | + |
| **Quality of evidence** | **Stroke** | **Chronic** |  | Moderate (-1) | Inconsistent | Moderate (-1) | Low (-2) | Low (-2) | Low (-2) | Low (-2) | Low (-2) |
| Footswitches [21] | Stroke | Chronic | 25 | ? | ? | ? | ? | ? | ? | + | ? |
| **Overall rating** | **Stroke** | **Chronic** |  | ? | ? | ? | ? | ? | ? | + | ? |
| **Quality of evidence** | **Stroke** | **Chronic** |  | Inconsistent | Inconsistent | Inconsistent | Inconsistent | Inconsistent | Inconsistent | Low (-2) | Inconsistent |
| Footswitches [5] | Stroke | Sub-acute | 25 | + | ? | + | ? | ? | + | + | ? |
| **Overall rating** | **Stroke** | **Sub-acute** |  | + | ? | + | ? | ? | + | + | ? |
| **Quality of evidence** | **Stroke** | **Sub-acute** |  | Low (-2) | Inconsistent | Low (-2) | Inconsistent | Inconsistent | Low (-2) | Low (-2) | Inconsistent |
| Frenchay Activities Index (FAI) [22] | Stroke | Acute | 35 | ? | + | + | + | ? | ? | - | ? |
| **Overall rating** | **Stroke** | **Acute** |  | ? | + | + | + | ? | ? | - | ? |
| **Quality of evidence** | **Stroke** | **Acute** |  | Inconsistent | Low (-2) | Low (-2) | Low (-2) | Inconsistent | Inconsistent | Low (-2) | Inconsistent |
| Frenchay Activities Index (FAI) [19] | Stroke | Chronic | 36 | ? | ? | ? | + | ? | ? | + | ? |
| Frenchay Activities Index (FAI) [19] | Stroke | Chronic | 238 | ? | ? | ? | ? | ? | ? | + | ? |
| Frenchay Activities Index (FAI) [19] | Stroke | Chronic | 52 | ? | ? | + | ? | ? | ? | ? | ? |
| Frenchay Activities Index (FAI) [19] | Stroke | Chronic | 127 | ? | + | ? | ? | ? | ? | ? | ? |
| Frenchay Activities Index (FAI) [19] | Stroke | Chronic | 70 | ? | ? | ? | ? | ? | ? | + | + |
| Frenchay Activities Index (FAI) [19] | Stroke | Chronic | 163 | ? | ? | ? | ? | ? | ? | ? | + |
| Frenchay Activities Index (FAI) [19] | Stroke | Chronic | 45 | ? | ? | ? | + | ? | ? | ? | ? |
| Frenchay Activities Index (FAI) [19] | Stroke | Chronic | 22 | ? | ? | + | ? | ? | ? | ? | ? |
| Frenchay Activities Index (FAI) [19] | Stroke | Chronic | 68 | ? | ? | ? | + | ? | ? | ? | ? |
| Frenchay Activities Index (FAI) [19] | Stroke | Chronic | 188 | ? | + | ? | ? | ? | ? | + | ? |
| Frenchay Activities Index (FAI) [19] | Stroke | Chronic | 14 | ? | ? | ? | + | ? | ? | ? | ? |
| Frenchay Activities Index (FAI) [19] | Stroke | Chronic | 581 | + | ? | ? | ? | ? | ? | ? | ? |
| Frenchay Activities Index (FAI) [19] | Stroke | Chronic | 935 | ? | ? | ? | ? | ? | ? | + | ? |
| Frenchay Activities Index (FAI) [19] | Stroke | Chronic | 383 | ? | ? | ? | ? | ? | ? | ? | + |
| Frenchay Activities Index (FAI) [20] | Stroke | Chronic |  | + | + | ? | + | ? | ? | + | ? |
| Frenchay Activities Index (FAI) [6] | Stroke | Chronic |  | ? | ? | + | ? | ? | ? | + | ? |
| Frenchay Activities Index (FAI) [19] | Stroke | Chronic |  | ? | ? | + | ? | ? | ? | + | ? |
| Frenchay Activities Index (FAI) [28] | Stroke | Chronic |  | ? | ? | - | ? | ? | ? | ? | ? |
| **Overall rating** | **Stroke** | **Chronic** |  | + | + | + | + | ? | ? | + | + |
| **Quality of evidence** | **Stroke** | **Chronic** |  | High | High | Moderate (-1) | High | Inconsistent | Inconsistent | High | High |
| Frenchay Arm Test (FAT) [13] | Stroke | Chronic | 45 | ? | ? | ? | + | ? | ? | ? | ? |
| Frenchay Arm Test (FAT) [7] | Stroke | Chronic | 38 | ? | ? | - | ? | ? | ? | + | - |
| Frenchay Arm Test (FAT) [16] | Stroke | Chronic | 10 | ? | ? | + | + | ? | ? | ? | ? |
| **Overall rating** | **Stroke** | **Chronic** |  | ? | ? | + | + | ? | ? | + | - |
| **Quality of evidence** | **Stroke** | **Chronic** |  | Inconsistent | Inconsistent | Low (-2) | Moderate (-1) | Inconsistent | Inconsistent | Moderate (-1) | Low (-2) |
| Fugl-Meyer Assessment (FMA) [16] | Stroke | Acute |  | ? | ? | + | + | ? | ? | - | + |
| Fugl-Meyer Assessment (FMA) [32] | Stroke | Acute | 78 | ? | + | + | + | ? | ? | + | + |
| Fugl-Meyer Assessment (FMA) [24] | Stroke | Acute |  | ? | + | ? | + | + | ? | + | + |
| **Overall rating** | **Stroke** | **Acute** |  | ? | + | + | + | + | ? | + | + |
| **Quality of evidence** | **Stroke** | **Acute** |  | Inconsistent | Moderate (-1) | Moderate (-1) | Moderate (-1) | Low (-2) | Inconsistent | Moderate (-1) | Moderate (-1) |
| Fugl-Meyer Assessment (FMA) [10] | Stroke | Chronic |  | ? | - | ? | ? | ? | ? | - | - |
| Fugl-Meyer Assessment (FMA) [7] | Stroke | Chronic |  | ? | ? | + | ? | ? | ? | + | + |
| Fugl-Meyer Assessment (FMA) [18] | Stroke | Chronic | 377 | ? | ? | + | + | ? | ? | ? | ? |
| Fugl-Meyer Assessment (FMA) [17] | Stroke | Chronic |  | ? | ? | ? | + | ? | ? | + | ? |
| **Overall rating** | **Stroke** | **Chronic** |  | ? | - | + | + | ? | ? | + | + |
| **Quality of evidence** | **Stroke** | **Chronic** |  | Inconsistent | Inconsistent | High | High | Inconsistent | Inconsistent | Low (-2) | Low (-2) |
| Fugl-Meyer test-Balance subscale (FM-B) [1] | Stroke | Acute | 110 | ? | ? | ? | ? | ? | ? | ? | + |
| Fugl-Meyer test-Balance subscale (FM-B) [1] | Stroke | Acute | 93 | ? | ? | ? | ? | ? | ? | ? | + |
| Fugl-Meyer test-Balance subscale (FM-B) [1] | Stroke | Acute | 80 | ? | ? | ? | ? | ? | ? | ? | + |
| Fugl-Meyer test-Balance subscale (FM-B) [1] | Stroke | Acute | 93 | ? | ? | ? | ? | ? | ? | ? | + |
| Fugl-Meyer test-Balance subscale (FM-B) [1] | Stroke | Acute | 80 | ? | ? | ? | ? | ? | ? | ? | + |
| **Overall rating** | **Stroke** | **Acute** |  | ? | ? | ? | ? | ? | ? | ? | + |
| **Quality of evidence** | **Stroke** | **Acute** |  | Inconsistent | Inconsistent | Inconsistent | Inconsistent | Inconsistent | Inconsistent | Inconsistent | High |
| Functional Ambulation Category (FAC) [3] | Stroke | Acute |  | ? | ? | + | + | ? | ? | - | + |
| Functional Ambulation Category (FAC) [1] | Stroke | Acute | 101 | ? | ? | ? | ? | ? | ? | ? | + |
| **Overall rating** | **Stroke** | **Acute** |  | ? | ? | + | + | ? | ? | - | + |
| **Quality of evidence** | **Stroke** | **Acute** |  | Inconsistent | Inconsistent | Low (-2) | Low (-2) | Inconsistent | Inconsistent | Low (-2) | High |
| Functional Ambulation Category (FAC) [2] | Stroke | Chronic | 31 | ? | ? | ? | ? | + | ? | + | ? |
| Functional Ambulation Category (FAC) [2] | Stroke | Chronic | 55 | ? | ? | ? | + | + | ? | + | ? |
| Functional Ambulation Category (FAC) [5] | Stroke | Chronic | 25 | + | ? | + | ? | ? | ? | ? | ? |
| Functional Ambulation Category (FAC) [6] | Stroke | Chronic |  | ? | ? | ? | + | + | ? | + | ? |
| **Overall rating** | **Stroke** | **Chronic** |  | + | ? | + | + | + | ? | + | ? |
| **Quality of evidence** | **Stroke** | **Chronic** |  | Low (-2) | Inconsistent | Low (-2) | Moderate (-1) | Moderate (-1) | Inconsistent | Moderate (-1) | Inconsistent |
| Functional Ambulation Category (FAC) [1] | Stroke | Sub-acute | 55 | ? | ? | ? | ? | ? | ? | ? | + |
| Functional Ambulation Category (FAC) [2] | Stroke | Sub-acute | 20 | ? | ? | ? | ? | ? | ? | + | ? |
| Functional Ambulation Category (FAC) [5] | Stroke | Sub-acute | 55 | + | ? | + | ? | ? | ? | + | ? |
| **Overall rating** | **Stroke** | **Sub-acute** |  | + | ? | + | ? | ? | ? | + | + |
| **Quality of evidence** | **Stroke** | **Sub-acute** |  | Moderate (-1) | Inconsistent | Moderate (-1) | Inconsistent | Inconsistent | Inconsistent | Moderate (-1) | Moderate (-1) |
| Functional Independence measure (FIM) [22] | Stroke | Acute | 52 | ? | + | + | + | ? | ? | + | + |
| **Overall rating** | **Stroke** | **Acute** |  | ? | + | + | + | ? | ? | + | + |
| **Quality of evidence** | **Stroke** | **Acute** |  | Inconsistent | Moderate (-1) | Moderate (-1) | Moderate (-1) | Inconsistent | Inconsistent | Moderate (-1) | Moderate (-1) |
| Functional Independence measure (FIM) [18] | Stroke | Chronic | 18 | + | ? | ? | - | ? | ? | ? | - |
| Functional Independence measure (FIM) [23] | stroke | Chronic |  | ? | ? | ? | + | + | ? | + | + |
| Functional Independence measure (FIM) [7] | stroke | Chronic |  | ? | ? | + | ? | ? | ? | - | + |
| Functional Independence measure (FIM) [16] | Stroke | Chronic | 83 | + | ? | + | + | ? | ? | - | + |
| Functional Independence measure (FIM) [28] | Stroke | Chronic |  | ? | ? | + | ? | ? | ? | ? | ? |
| **Overall rating** | **Stroke** | **Chronic** |  | + | ? | + | + | + | ? | + | + |
| **Quality of evidence** | **Stroke** | **Chronic** |  | High | Inconsistent | Moderate (-1) | Moderate (-1) | Low (-2) | Inconsistent | Moderate (-1) | Moderate (-1) |
| Functional Independence measure (FIM) [27] | TBI | 332 |  | + | ? | + | ? | ? | ? | ? | ? |
| Functional Independence measure (FIM) [27] | TBI |  |  | ? | ? | ? | + | ? | ? | ? | ? |
| **Overall rating** | **TBI** |  |  | + | ? | + | + | ? | ? | ? | ? |
| **Quality of evidence** | **TBI** |  |  | High | Inconsistent | High | Low (-2) | Inconsistent | Inconsistent | Inconsistent | Inconsistent |
| Grasp-Release test [18] | Stroke | Chronic | 60 | ? | ? | ? | ? | ? | ? | ? | - |
| Grasp-Release test [18] | Stroke | Chronic | 12 | ? | + | ? | ? | ? | ? | ? | + |
| **Overall rating** | **Stroke** | **Chronic** |  | ? | + | ? | ? | ? | ? | ? | + |
| **Quality of evidence** | **Stroke** | **Chronic** |  | Inconsistent | Low (-2) | Inconsistent | Inconsistent | Inconsistent | Inconsistent | Inconsistent | Moderate (-1) |
| Human activity profile (HAP) [33] | Stroke | Chronic |  | + | + | + | + | ? | ? | - | - |
| Human activity profile (HAP) [19] | Stroke | Chronic | 24 | ? | ? | ? | ? | ? | ? | + | ? |
| **Overall rating** | **Stroke** | **Chronic** |  | + | + | + | + | ? | ? | + | - |
| **Quality of evidence** | **Stroke** | **Chronic** |  | Low (-2) | Low (-2) | Low (-2) | Low (-2) | Inconsistent | Inconsistent | Low (-2) | Low (-2) |
| Jebsen Hand Function Test [18] | Stroke | Chronic | 33 | ? | ? | ? | ? | ? | ? | ? | - |
| Jebsen Hand Function Test [13] | Stroke | Chronic |  | ? | ? | + | ? | ? | ? | + | + |
| **Overall rating** | **Stroke** | **Chronic** |  | ? | ? | + | ? | ? | ? | + | + |
| **Quality of evidence** | **Stroke** | **Chronic** |  | Inconsistent | Inconsistent | Low (-2) | Inconsistent | Inconsistent | Inconsistent | Low (-2) | Low (-2) |
| London Handicap scale (LHS) [20] | Stroke | Chronic | 37 | + | + | + | ? | ? | ? | + | ? |
| **Overall rating** | **Stroke** | **Chronic** |  | + | + | + | ? | ? | ? | + | ? |
| **Quality of evidence** | **Stroke** | **Chronic** |  | Low (-2) | Low (-2) | Low (-2) | Inconsistent | Inconsistent | Inconsistent | Low (-2) | Inconsistent |
| London handicap score-LHS [30] | Stroke | Acute | 361 | + | ? | + | + | ? | ? | + | ? |
| **Overall rating** | **Stroke** | **Acute** |  | + | ? | + | + | ? | ? | + | ? |
| **Quality of evidence** | **Stroke** | **Acute** |  | High | inconsistent | High | High | Inconsistent | Inconsistent | High | Inconsistent |
| MESUPES (Motor Evaluation Scale for Upper Extremity in Stroke Patients) [13] | Stroke | Chronic |  | ? | ? | ? | ? | ? | ? | ? | ? |
| MESUPES (Motor Evaluation Scale for Upper Extremity in Stroke Patients) [12] | Stroke | Chronic | 396 | + | ? | + | ? | ? | ? | + | ? |
| **Overall rating** | **Stroke** | **Chronic** |  | + | ? | + | ? | ? | ? | + | ? |
| **Quality of evidence** | **Stroke** | **Chronic** |  | High | Inconsistent | High | Inconsistent | Inconsistent | Inconsistent | High | Inconsistent |
| Mini Mental State Examination (MMSE) [7] | Stroke | Acute | 116 | ? | ? | + | ? | ? | ? | - | ? |
| **Overall rating** | **Stroke** | **Acute** |  | ? | ? | + | ? | ? | ? | - | ? |
| **Quality of evidence** | **Stroke** | **Acute** |  | Inconsistent | Inconsistent | High | Inconsistent | Inconsistent | Inconsistent | Moderate (-1) | Inconsistent |
| Mini-Mental State Examination (MMSE) [24] | Stroke | Chronic | 75 | ? | - | - | - | + | ? | + | ? |
| **Overall rating** | **Stroke** | **Chronic** |  | ? | - | - | - | + | ? | + | ? |
| **Quality of evidence** | **Stroke** | **Chronic** |  | Inconsistent | Moderate (-1) | Moderate (-1) | Moderate (-1) | Moderate (-1) | Inconsistent | Moderate (-1) | Inconsistent |
| Modified Ashworth scale [7] | Stroke | Acute |  | ? | ? | - | ? | ? | ? | - | ? |
| Modified Ashworth scale [16] | Stroke | Acute |  | ? | ? | + | + | ? | ? | - | ? |
| Modified Ashworth scale [18] | Stroke | Acute | 36 | ? | ? | ? | + | ? | ? | ? | ? |
| Modified Ashworth scale [24] | Stroke | Acute |  | ? | ? | ? | - | - | ? | + | ? |
| **Overall rating** | **Stroke** | **Acute** |  | ? | ? | + | + | - | ? | + | ? |
| **Quality of evidence** | **Stroke** | **Acute** |  | Inconsistent | Inconsistent | Low (-2) | Low (-2) | Low (-2) | Inconsistent | Low (-2) | Inconsistent |
| Modified Emory Functional Ambulation Profile (M-EFAM) [1] | Stroke | Acute | 40 | ? | ? | ? | ? | ? | ? | ? | + |
| **Overall rating** | **Stroke** | **Acute** |  | ? | ? | ? | ? | ? | ? | ? | + |
| **Quality of evidence** | **Stroke** | **Acute** |  | Inconsistent | Inconsistent | Inconsistent | Inconsistent | Inconsistent | Inconsistent | Inconsistent | Low (-2) |
| Modified Emory Functional Ambulation Profile (M-EFAM) [26] | Stroke | Chronic | 26 | ? | ? | + | ? | ? | ? | + | + |
| Modified Emory Functional Ambulation Profile (M-EFAM) [2] | Stroke | Chronic | 28 | ? | ? | ? | ? | + | ? | + | ? |
| Modified Emory Functional Ambulation Profile (M-EFAM) [2] | Stroke | Chronic | 26 | ? | ? | ? | + | + | ? | + | ? |
| Modified Emory Functional Ambulation Profile (M-EFAM) [2] | Stroke | Chronic | 40 | ? | ? | ? | + | ? | ? | + | ? |
| **Overall rating** | **Stroke** | **Chronic** |  | ? | ? | + | + | + | ? | + | + |
| **Quality of evidence** | **Stroke** | **Chronic** |  | Inconsistent | Inconsistent | Low (-2) | Moderate (-1) | Moderate (-1) | Inconsistent | High | Low (-2) |
| Modified Functional Reach test (MFRT) [34] | Stroke | Chronic |  | ? | ? | + | ? | + | ? | ? | + |
| Modified Functional Reach test (MFRT) [1] | Stroke | Chronic | 35 | ? | ? | ? | ? | ? | ? | ? | + |
| **Overall rating** | **Stroke** | **Chronic** |  | ? | ? | + | ? | + | ? | ? | + |
| **Quality of evidence** | **Stroke** | **Chronic** |  | Inconsistent | Inconsistent | Low (-2) | Inconsistent | Low (-2) | Inconsistent | Inconsistent | Low (-2) |
| Modified Rankin Handicap scale [7] | Stroke | Acute | 1034 | ? | ? | + | ? | ? | ? | - | - |
| **Overall rating** | **Stroke** | **Acute** |  | ? | ? | + | ? | ? | ? | - | - |
| **Quality of evidence** | **Stroke** | **Acute** |  | Inconsistent | Inconsistent | High | Inconsistent | Inconsistent | Inconsistent | Moderate (-1) | Moderate (-1) |
| Modified Rankin Handicap Scale | Stroke | Chronic | 63 | ? | ? | + | - | ? | ? | + | + |
| **Overall rating** | **Stroke** | **Chronic** |  | ? | ? | + | - | ? | ? | + | + |
| **Quality of evidence** | **Stroke** | **Chronic** |  | Inconsistent | Inconsistent | Moderate (-1) | Moderate (-1) | Inconsistent | Inconsistent | Moderate (-1) | Moderate (-1) |
| Motor activity log [11] | Stroke | Chronic | 56 | ? | ? | ? | ? | ? | ? | + | ? |
| Motor activity log [11] | Stroke | Chronic | 20 | ? | ? | ? | ? | ? | ? | + | ? |
| Motor activity log [11] | Stroke | Chronic | 41 | ? | ? | ? | ? | ? | ? | + | ? |
| Motor activity log [11] | Stroke | Chronic | 27 | ? | ? | + | ? | ? | ? | ? | ? |
| Motor activity log (MAL-14)[9] | Stroke | Chronic |  | ? | + | - | - | ? | ? | - | ? |
| Motor activity log (MAL-14) [13] | Stroke | Chronic | 56 | ? | ? | ? | ? | ? | ? | ? | ? |
| Motor Activity Log [18] | Stroke | Chronic |  | + | + | - | ? | ? | ? | ? | - |
| Motor Activity Log [14] | Stroke | Chronic |  | + | + | - | ? | ? | ? | - | + |
| **Overall rating** | **Stroke** | **Chronic** |  | + | + | + | ? | ? | ? | + | + |
| **Quality of evidence** | **Stroke** | **Chronic** |  | Low (-2) | Low (-2) | Low (-2) | Low (-2) | Inconsistent | Inconsistent | High | Low (-2) |
| Motor Assessment Scale (MAS) [1] | Stroke | Acute | 61 | ? | ? | ? | ? | ? | ? | ? | + |
| **Overall rating** | **Stroke** | **Acute** |  | ? | ? | ? | ? | ? | ? | ? | + |
| **Quality of evidence** | **Stroke** | **Acute** |  | Inconsistent | Inconsistent | Inconsistent | Inconsistent | Inconsistent | Inconsistent | Inconsistent | Moderate (-1) |
| Motor Assessment Scale (MAS) [7] | Stroke | Chronic |  | ? | ? | + | ? | ? | ? | + | + |
| Motor Assessment Scale (MAS) [17] | Stroke | Chronic |  | ? | ? | ? | + | ? | ? | + | ? |
| Motor Assessment Scale (MAS) [32] | Stroke | Chronic | 37 | ? | ? | + | + | ? | ? | + | ? |
| Motor Assessment Scale (MAS) [16] | Stroke | Chronic |  | ? | ? | + | + | ? | ? | - | ? |
| **Overall rating** | **Stroke** | **Chronic** |  | ? | ? | + | + | ? | ? | + | + |
| **Quality of evidence** | **Stroke** | **Chronic** |  | Inconsistent | Inconsistent | Low (-2) | Low (-2) | Inconsistent | Inconsistent | Low (-2) | Low (-2) |
| Motor status score-MSS [16] | Stroke | Acute |  | ? | ? | + | + | ? | ? | - | ? |
| Motor status score-MSS [11] | Stroke | Acute | 18 | ? | ? | ? | ? | ? | ? | + | ? |
| **Overall rating** | **Stroke** | **Acute** |  | ? | ? | ? | ? | ? | ? | + | ? |
| **Quality of evidence** | **Stroke** | **Acute** |  | Inconsistent | Inconsistent | Low (-2) | Low (-2) | Inconsistent | Inconsistent | Low (-2) | Inconsistent |
| Motricity index (MI) [3] | Stroke | Chronic |  | ? | ? | ? | + | ? | ? | - | ? |
| Motricity index (MI) [7] | Stroke | Chronic |  | ? | ? | - | ? | ? | ? | + | ? |
| Motricity index (MI) [17] | Stroke | Chronic |  | ? | ? | ? | + | ? | ? | + | ? |
| Motricity index (MI) [6] | Stroke | Chronic |  | ? | ? | + | + | ? | ? | + | ? |
| Motricity index (MI) [32] | Stroke | Chronic | 55 | ? | + | ? | + | ? | ? | + | ? |
| **Overall rating** | **Stroke** | **Chronic** |  | ? | + | + | + | ? | ? | + | ? |
| **Quality of evidence** | **Stroke** | **Chronic** |  | Inconsistent | Moderate (-1) | Low (-2) | Moderate (-1) | Inconsistent | Inconsistent | Moderate (-1) | Inconsistent |
| Nine-Hole Peg test (NHPT) [17] | Stroke | Chronic |  | ? | ? | + | - | ? | ? | + | ? |
| Nine-Hole Peg test (NHPT) [11] | Stroke | Chronic | 62 | ? | ? | + | ? | ? | ? | + | ? |
| Nine-Hole Peg test (NHPT) [16] | Stroke | Chronic |  | ? | ? | ? | + | ? | ? | - | ? |
| **Overall rating** | **Stroke** | **Chronic** |  | ? | ? | + | + | ? | ? | + | ? |
| **Quality of evidence** | **Stroke** | **Chronic** |  | Inconsistent | Inconsistent | Moderate (-1) | Low (-2) | Inconsistent | Inconsistent | Moderate (-1) | Inconsistent |
| Nottingham leisure activity (NLA) [29] | Stroke | Chronic |  | ? | + | + | - | ? | ? | + | ? |
| Nottingham leisure activity (NLA) [19] | Stroke | Chronic | 21 | ? | ? | - | ? | ? | ? | ? | ? |
| Nottingham leisure activity (NLA) [19] | Stroke | Chronic | 20 | ? | ? | ? | - | ? | ? | ? | ? |
| **Overall rating** | **Stroke** | **Chronic** |  | ? | ? | + | - | ? | ? | + | ? |
| **Quality of evidence** | **Stroke** | **Chronic** |  | Inconsistent | Low (-2) | Low (-2) | Low (-2) | Inconsistent | Inconsistent | Low (-2) | Inconsistent |
| Pedometer (Conventional) [25] | Stroke | Chronic | 16 | ? | ? | - | ? | ? | ? | ? | ? |
| Pedometer (model 650 Yamasa Tokei Co., Yamax Digi Walker) [25] | Stroke | Chronic | 20 | ? | ? | ? | ? | ? | ? | - | + |
| Pedometers [21] | Stroke | Chronic | 16 | ? | ? | ? | ? | ? | ? | + | ? |
| Pedometers [5] | Stroke | Chronic |  | ? | ? | ? | ? | ? | ? | ? | ? |
| **Overall rating** | **Stroke** | **Chronic** |  | ? | ? | - | ? | ? | ? | + | + |
| **Quality of evidence** | **Stroke** | **Chronic** |  | Inconsistent | Inconsistent | Low (-2) | Inconsistent | Inconsistent | Inconsistent | Low (-2) | Low (-2) |
| Postural Assessment Scale for Stroke Patients (PASS) [1] | Stroke | Acute | 202 | ? | ? | ? | ? | ? | ? | ? | + |
| **Overall rating** | **Stroke** | **Acute** |  | ? | ? | ? | ? | ? | ? | ? | + |
| **Quality of evidence** | **Stroke** | **Acute** |  | Inconsistent | Inconsistent | Inconsistent | Inconsistent | Inconsistent | Inconsistent | Inconsistent | High |
| Postural Assessment Scale for Stroke Patients (PASS) [1] | Stroke | Chronic | 167 | ? | ? | ? | ? | ? | ? | ? | + |
| Postural Assessment Scale for Stroke Patients (PASS) [1] | Stroke | Chronic | 167 | ? | ? | ? | ? | ? | ? | ? | + |
| Postural Assessment Scale for Stroke Patients (PASS) [34] | Stroke | Chronic |  | ? | ? | ? | ? | ? | ? | ? | + |
| **Overall rating** | **Stroke** | **Chronic** |  | ? | ? | ? | ? | ? | ? | ? | + |
| **Quality of evidence** | **Stroke** | **Chronic** |  | Inconsistent | Inconsistent | inconsistent | Inconsistent | Inconsistent | Inconsistent | Inconsistent | High |
| Postural Assessment Scale for Stroke Patients Trunk Control (PASS-TC) [1] | Stroke | Acute | 110 | ? | ? | ? | ? | ? | ? | ? | + |
| Postural Assessment Scale for Stroke Patients Trunk Control (PASS-TC) [1] | Stroke | Acute | 246 | ? | ? | ? | ? | ? | ? | ? | + |
| **Overall rating** | **Stroke** | **Acute** |  | ? | ? | ? | ? | ? | ? | ? | + |
| **Quality of evidence** | **Stroke** | **Acute** |  | Inconsistent | Inconsistent | Inconsistent | Inconsistent | Inconsistent | Inconsistent | Inconsistent | High |
| Postural Assessment Scale for Stroke Patients Trunk Control (PASS-TC) [1] | Stroke | Chronic | 93 | ? | ? | ? | ? | ? | ? | ? | + |
| Postural Assessment Scale for Stroke Patients Trunk Control (PASS-TC) [1] | Stroke | Chronic | 80 | ? | ? | ? | ? | ? | ? | ? | + |
| Postural Assessment Scale for Stroke Patients Trunk Control (PASS-TC) [1] | Stroke | Chronic | 93 | ? | ? | ? | ? | ? | ? | ? | + |
| Postural Assessment Scale for Stroke Patients Trunk Control (PASS-TC) [1] | Stroke | Chronic | 80 | ? | ? | ? | ? | ? | ? | ? | + |
| Postural Assessment Scale for Stroke Patients Trunk Control (PASS-TC) [1] | Stroke | Chronic | 203 | ? | ? | ? | ? | ? | ? | ? | + |
| Postural Assessment Scale for Stroke Patients Trunk Control (PASS-TC) [1] | Stroke | Chronic | 189 | ? | ? | ? | ? | ? | ? | ? | + |
| **Overall rating** | **Stroke** | **Chronic** |  | ? | ? | ? | ? | ? | ? | ? | + |
| **Quality of evidence** | **Stroke** | **Chronic** |  | Inconsistent | Inconsistent | Inconsistent | Inconsistent | Inconsistent | Inconsistent | Inconsistent | High |
| Rivermead mobility index (RMI) [33] | Stroke | Acute | 38 | + | + | + | + | ? | ? | + | + |
| Rivermead mobility index (RMI) [22] | Stroke | Acute |  | + | + | + | + | ? | ? | + | + |
| **Overall rating** | **Stroke** | **Acute** |  | + | + | + | + | ? | ? | + | + |
| **Quality of evidence** | **Stroke** | **Acute** |  | Low (-2) | Low (-2) | Low (-2) | Low (-2) | Inconsistent | Inconsistent | Low (-2) | Low (-2) |
| Rivermead mobility index (RMI) [32] | Stroke | Chronic |  | ? | + | ? | ? | ? | ? | + | ? |
| Rivermead mobility index (RMI) [23] | Stroke | Chronic |  | ? | ? | + | ? | ? | ? | + | ? |
| Rivermead mobility index (RMI) [1] | Stroke | Chronic |  | ? | ? | ? | ? | ? | ? | ? | ? |
| Rivermead mobility index (RMI) [1] | Stroke | Chronic |  | ? | ? | ? | ? | ? | ? | ? | ? |
| Rivermead mobility index (RMI) [2] | Stroke | Chronic | 73 | ? | ? | ? | ? | ? | ? | + | + |
| Rivermead mobility index (RMI) [2] | Stroke | Chronic | 38 | ? | ? | ? | ? | ? | ? | + | ? |
| **Overall rating** | **Stroke** | **Chronic** |  | ? | + | + | ? | ? | ? | + | + |
| **Quality of evidence** | **Stroke** | **Chronic** |  | Inconsistent | Low (-2) | Low (-2) | Inconsistent | Inconsistent | Inconsistent | High | Moderate (-1) |
| Rivermead mobility index (RMI) [3] | Stroke | Sub-acute | 73 | ? | + | + | + | ? | ? | - | + |
| **Overall rating** | **Stroke** | **Sub-acute** |  | ? | + | + | + | ? | ? | - | + |
| **Quality of evidence** | **Stroke** | **Sub-acute** |  | Inconsistent | Moderate (-1) | Moderate (-1) | Moderate (-1) | Inconsistent | Inconsistent | Moderate (-1) | Moderate (-1) |
| Rivermead mobility index (RMI) [2] | TBI |  | 20 | ? | ? | ? | ? | ? | ? | + | ? |
| **Overall rating** | **TBI** |  |  | ? | ? | ? | ? | ? | ? | + | ? |
| **Quality of evidence** | **TBI** |  |  | Inconsistent | inconsistent | Inconsistent | Inconsistent | Inconsistent | Inconsistent | Low (-2) | Inconsistent |
| Rivermead mobility Assessment (RMA) [22] | Stroke | Acute | 51 | ? | ? | - | ? | ? | ? | + | ? |
| **Overall rating** | **Stroke** | **Acute** |  | ? | ? | - | ? | ? | ? | + | ? |
| **Quality of evidence** | **Stroke** | **Acute** |  | Inconsistent | Inconsistent | Moderate (-1) | Inconsistent | Inconsistent | Inconsistent | Moderate (-1) | Inconsistent |
| Rivermead mobility Assessment (RMA) [7] | Stroke | Chronic |  | ? | ? | - | ? | ? | ? | - | - |
| Rivermead mobility Assessment (RMA) [17] | Stroke | Chronic |  | ? | ? | ? | - | ? | ? | + | ? |
| Rivermead mobility Assessment (RMA) [11] | stroke | Chronic |  | ? | ? | ? | ? | ? | + | + | ? |
| Rivermead mobility Assessment (RMA) [32] | Stroke | Chronic | 158 | ? | + | + | ? | ? | ? | ? | ? |
| Rivermead mobility Assessment (RMA) [16] | Stroke | Chronic |  | ? | ? | + | ? | ? | ? | - | ? |
| Rivermead mobility Assessment (RMA) [12] | Stroke | Chronic |  | ? | ? | ? | ? | ? | ? | ? | ? |
| **Overall rating** | **Stroke** | **Chronic** |  | ? | + | + | - | ? | + | + | - |
| **Quality of evidence** | **Stroke** | **Chronic** |  | Inconsistent | High | High | Low (-2) | Inconsistent | Low (-2) | Low (-2) | Low (-2) |
| Medical Outcomes Study 36-Item Short Form Health Survey (SF-36) [27] | TBI |  |  | ? | + | ? | ? | ? | ? | + | ? |
| **Overall rating** | **TBI** |  |  | ? | + | ? | ? | ? | ? | + | ? |
| **Quality of evidence** | **TBI** |  |  | Inconsistent | Low (-2) | Inconsistent | Inconsistent | Inconsistent | Inconsistent | Low (-2) | Inconsistent |
| Medical Outcomes Study 36-Item Short Form Health Survey (SF-36) [7] | Stroke | Chronic |  | ? | ? | - | ? | ? | ? | + | + |
| Medical Outcomes Study 36-Item Short Form Health Survey (SF-36) [28] | Stroke | Chronic | 60 | ? | ? | - | ? | ? | ? | ? | ? |
| Medical Outcomes Study 36-Item Short Form Health Survey (SF-36) [23] | Stroke | Chronic |  | ? | + | ? | ? | ? | ? | + | ? |
| Medical Outcomes Study 36-Item Short Form Health Survey (SF-36) [29] | Stroke | Chronic |  | ? | + | + | ? | ? | ? | + | + |
| Medical Outcomes Study 36-Item Short Form Health Survey (SF-36) [18] | Stroke | Chronic | 19 | + | ? | ? | + | ? | ? | ? | - |
| **Overall rating** | **Stroke** | **Chronic** |  | + | + | - | + | ? | ? | + | + |
| **Quality of evidence** | **Stroke** | **Chronic** |  | Low (-2) | Low (-2) | Moderate (-1) | Low (-2) | Inconsistent | Inconsistent | Low (-2) | Low (-2) |
| Sickness Impact profile (SIP) [33] | TBI |  | 25 | + | + | + | ? | ? | ? | + | + |
| **Overall rating** | **TBI** |  |  | + | + | + | ? | ? | ? | + | + |
| **Quality of evidence** | **TBI** |  |  | Low (-2) | Low (-2) | Low (-2) | Inconsistent | Inconsistent | Inconsistent | Low (-2) | Low (-2) |
| Sickness Impact profile (SIP) [7] | stroke | Chronic |  | ? | ? | - | ? | ? | ? | - | ? |
| Sickness Impact profile (SIP) [28] | Stroke | Chronic | 574 | ? | ? | + | ? | ? | ? | - | ? |
| Sickness impact profile (SIP) [28] | Stroke | chronic |  | ? | ? | + | ? | ? | ? | ? | ? |
| Sickness Impact profile (SIP) [29] | Stroke | Chronic |  | ? | + | ? | ? | ? | ? | + | ? |
| **Overall rating** | **Stroke** | **Chronic** |  | ? | + | + | ? | ? | ? | + | ? |
| **Quality of evidence** | **Stroke** | **Chronic** |  | Inconsistent | Low (-2) | High | Inconsistent | Inconsistent | Inconsistent | Moderate (-1) | Inconsistent |
| StepWatch Activity Monitor or Step Activity Monitor (SAM) [25] | Stroke | chronic | 17 | ? | ? | + | ? | ? | ? | ? | ? |
| StepWatch Activity Monitor or Step Activity Monitor (SAM) [25] | Stroke | Chronic | 16 | ? | ? | + | ? | ? | ? | ? | ? |
| StepWatch Activity Monitor or Step Activity Monitor (SAM) [25] | Stroke | Chronic | 40 | ? | ? | + | ? | ? | ? | ? | ? |
| StepWatch Activity Monitor or Step Activity Monitor (SAM) [25] | Stroke | Chronic | 30 | ? | ? | ? | ? | ? | ? | + | + |
| StepWatch Activity Monitor or Step Activity Monitor (SAM) [25] | Stroke | Chronic | 25 | ? | ? | ? | ? | ? | ? | + | + |
| StepWatch Activity Monitor or Step Activity Monitor (SAM) [25] | Stroke | Chronic | 16 | ? | ? | ? | ? | ? | ? | ? | + |
| StepWatch Activity Monitor or Step Activity Monitor (SAM) [21] | Stroke | Chronic | 16 | ? | ? | + | ? | ? | ? | + | ? |
| StepWatch Activity Monitor or Step Activity Monitor (SAM) [21] | Stroke | Chronic | 21 | ? | ? | ? | ? | ? | ? | + | ? |
| StepWatch Activity Monitor or Step Activity Monitor (SAM) [21] | Stroke | Chronic | 53 | ? | ? | ? | ? | ? | ? | + | ? |
| StepWatch Activity Monitor or Step Activity Monitor (SAM) [21] | Stroke | Chronic | 17 | ? | ? | + | ? | ? | ? | ? | ? |
| StepWatch Activity Monitor or Step Activity Monitor (SAM) [21] | Stroke | Chronic | 50 | ? | ? | ? | ? | ? | ? | + | + |
| StepWatch Activity Monitor or Step Activity Monitor (SAM) [21] | Stroke | Chronic | 25 | ? | ? | ? | ? | ? | ? | + | ? |
| StepWatch Activity Monitor or Step Activity Monitor (SAM) [21] | Stroke | Chronic | 27 | ? | ? | ? | ? | ? | ? | + | ? |
| StepWatch Activity Monitor or Step Activity Monitor (SAM) [2] | Stroke | Chronic | 19 | ? | ? | ? | ? | ? | ? | + | ? |
| StepWatch Activity Monitor or Step Activity Monitor (SAM) [2] | Stroke | Chronic | 17 | ? | ? | ? | + | ? | ? | ? | ? |
| **Overall rating** | **Stroke** | **Chronic** |  | ? | ? | + | + | ? | ? | + | ? |
| **Quality of evidence** | **Stroke** | **Chronic** |  | Inconsistent | Inconsistent | Low (-2) | Low (-2) | Inconsistent | Inconsistent | High | Moderate (-1) |
| Stroke impact scale (SIS) [33] | Stroke | Chronic |  | + | + | + | + | ? | ? | + | - |
| Stroke impact scale (SIS) [7] | Stroke | Chronic | 696 | ? | ? | - | ? | ? | ? | + | - |
| Stroke impact scale (SIS) [28] | Stroke | Chronic |  | ? | ? | - | ? | ? | ? | ? | ? |
| Stroke Impact Scale (SIS) [29] | Stroke | Chronic |  | ? | + | + | ? | - | ? | + | + |
| Stroke impact scale (SIS) [15] | Stroke | Chronic |  | ? | ? | ? | ? | ? | ? | ? | + |
| Stroke impact scale (SIS) [30] | Stroke | Chronic |  | ? | ? | ? | ? | ? | ? | + | ? |
| Stroke impact scale (SIS) [16] | Stroke | Chronic |  | ? | ? | + | ? | ? | ? | - | ? |
| **Overall rating** | **Stroke** | **Chronic** |  | + | + | + | + | - | ? | + | + |
| **Quality of evidence** | **Stroke** | **Chronic** |  | Low (-2) | Low (-2) | Moderate (-1) | Low (-2) | Low (-2) | Inconsistent | High | Moderate (-1) |
| Stroke impact scale (SIS) [20] | Stroke | Sub-acute | 25 | + | + | + | ? | ? | ? | + | ? |
| **Overall rating** | **Stroke** | **Sub-acute** |  | + | + | + | ? | ? | ? | + | ? |
| **Quality of evidence** | **Stroke** | **Sub-acute** |  | Low (-2) | Low (-2) | Low (-2) | Inconsistent | Inconsistent | Inconsistent | Low (-2) | Inconsistent |
| Stroke Rehabilitation assessment of movement (STREAM) [11] | Stroke | Chronic |  | ? | ? | + | ? | ? | ? | + | ? |
| Stroke Rehabilitation assessment of movement (STREAM) [32] | Stroke | Chronic | 134 | ? | + | + | + | ? | ? | ? | + |
| Stroke Rehabilitation assessment of movement (STREAM) [12] | Stroke | Chronic | 351 | + | ? | + | ? | ? | ? | + | ? |
| Stroke Rehabilitation assessment of movement (STREAM) [10] | Stroke | Chronic | 80 | ? | + | + | + | + | ? | + | + |
| **Overall rating** | **Stroke** | **Chronic** |  | + | + | + | + | + | ? | + | + |
| **Quality of evidence** | **stroke** | **Chronic** |  | High | High | High | High | Moderate (-1) | Inconsistent | High | High |
| Stroke Specific Quality of Life Scale (SSQOL) [28] | Stroke | Chronic |  | ? | ? | - | ? | ? | ? | ? | ? |
| Stroke Specific Quality of Life Scale (SSQOL) [7] | Stroke | Chronic |  | ? | + | + | ? | ? | ? | - | + |
| Stroke Specific Quality of Life Scale (SSQOL) [29] | Stroke | Chronic | 71 | ? | + | ? | - | ? | ? | + | + |
| **Overall rating** | **Stroke** | **Chronic** |  | ? | + | + | - | ? | ? | + | + |
| **Quality of evidence** | **Stroke** | **Chronic** |  | Inconsistent | Moderate (-1) | Low (-2) | Moderate (-1) | Inconsistent | Inconsistent | Moderate (-1) | Moderate (-1) |
| Three Point Postural Assessment Scale for Stroke Patients (PASS-3P) [1] | Stroke | Acute | 202 | ? | ? | ? | ? | ? | ? | ? | + |
| Three Point Postural Assessment Scale for Stroke Patients (PASS-3P) [1] | Stroke | Acute | 167 | ? | ? | ? | ? | ? | ? | ? | + |
| Three Point Postural Assessment Scale for Stroke Patients (PASS-3P) [1] | Stroke | Acute | 167 | ? | ? | ? | ? | ? | ? | ? | + |
| **Overall rating** | **Stroke** | **Acute** |  | ? | ? | ? | ? | ? | ? | ? | + |
| **Quality of evidence** | **Stroke** | **Acute** |  | Inconsistent | Inconsistent | Inconsistent | Inconsistent | Inconsistent | Inconsistent | Inconsistent | High |
| Timed Up and Go test (TUG) [22] | Stroke | Chronic |  | ? | ? | + | + | ? | ? | - | + |
| Timed Up and Go test (TUG) [7] | Stroke | Chronic |  | ? | ? | + | ? | ? | ? | - | ? |
| Timed Up and Go test (TUG) [26] | Stroke | Chronic | 50 | - | ? | + | ? | ? | ? | ? | + |
| Timed Up and Go test (TUG) [26] | Stroke | Chronic | 11 | ? | ? | + | ? | ? | ? | ? | ? |
| Timed Up and Go test (TUG) [26] | Stroke | Chronic | 44 | ? | ? | ? | ? | ? | ? | + | + |
| Timed Up and Go test (TUG) [4] | Stroke | Chronic | 343 | ? | ? | + | ? | ? | ? | ? | ? |
| Timed Up and Go test (TUG) [2] | Stroke | Chronic | 11 | ? | ? | ? | + | ? | ? | + | ? |
| Timed Up and Go test (TUG) [2] | Stroke | Chronic | 50 | ? | ? | ? | + | ? | ? | ? | ? |
| **Overall rating** | **Stroke** | **Chronic** |  | - | ? | + | + | ? | ? | + | + |
| **Quality of evidence** | **Stroke** | **Chronic** |  | Low (-2) | Inconsistent | High | Moderate (-1) | Inconsistent | Inconsistent | Moderate (-1) | Moderate (-1) |
| Timed Up and Go test (TUG) [4] | TBI |  | 24 | ? | ? | + | ? | ? | ? | ? | ? |
| **Overall rating** | **TBI** |  |  | ? | ? | + | ? | ? | ? | ? | ? |
| **Quality of evidence** | **TBI** |  |  | Inconsistent | Inconsistent | Low (-2) | Inconsistent | Inconsistent | Inconsistent | Inconsistent | Inconsistent |
| Triaxial accelerometer/ RT3 [21] | Stroke | Chronic | 20 | ? | ? | + | ? | ? | ? | + | ? |
| Triaxial accelerometer/ RT3 [21] | Stroke | Chronic | 52 | ? | ? | + | ? | ? | ? | ? | ? |
| Triaxial accelerometer/ RT3 [25] | Stroke | Chronic | 20 | ? | ? | - | ? | ? | ? | ? | ? |
| **Overall rating** | **Stroke** | **Chronic** |  | ? | ? | + | ? | ? | ? | + | ? |
| **Quality of evidence** | **Stroke** | **Chronic** |  | Inconsistent | Inconsistent | Moderate (-1) | Inconsistent | Inconsistent | Inconsistent | Low (-2) | Inconsistent |
| Trunk Control Test [6] | Stroke | Chronic |  | ? | ? | ? | + | + | ? | + | ? |
| Trunk Control test [35] | Stroke | Chronic | 20 | ? | + | ? | ? | - | ? | + | ? |
| Trunk Control test [34] | Stroke | Chronic |  | ? | + | ? | + | ? | ? | + | ? |
| **Overall rating** | **Stroke** | **Chronic** |  | ? | + | ? | + | + | ? | + | ? |
| **Quality of evidence** | **Stroke** | **Chronic** |  | Inconsistent | Low (-2) | Inconsistent | Low (-2) | Low (-2) | Inconsistent | Low (-2) | Inconsistent |
| Trunk Impairment Scale [35] | Stroke | Chronic |  | + | + | ? | + | + | + | + | ? |
| Trunk Impairment Scale [35] | Stroke | Chronic | 73 | ? | + | ? | ? | + | ? | ? | + |
| Trunk Impairment Scale (TIS)- Verheyden version [34] | Stroke | Chronic |  | ? | - | + | + | ? | ? | + | ? |
| Trunk Impairment Scale - Fujiwara version [34] | Stroke | Chronic |  | ? | + | ? | + | ? | ? | + | + |
| Trunk Impairment Scale [34] | Stroke | Chronic |  | ? | + | ? | + | + | ? | ? | ? |
| Trunk Impairment Scale [34] | Stroke | Chronic |  | ? | + | + | + | + | ? | ? | ? |
| **Overall rating** | **Stroke** | **Chronic** |  | + | + | + | + | + | + | + | + |
| **Quality of evidence** | **Stroke** | **Chronic** |  | Moderate (-1) | Moderate (-1) | Low (-2) | Low (-2) | Moderate (-1) | Low (-2) | Low (-2) | Moderate (-1) |
| Uniaxial accelerometer [21] | Stroke | Acute | 34 | ? | ? | ? | ? | ? | ? | + | ? |
| Uniaxial accelerometer [21] | Stroke | Acute | 45 | ? | ? | ? | ? | ? | ? | + | ? |
| **Overall rating** | **Stroke** | **Acute** |  | ? | ? | ? | ? | ? | ? | + | ? |
| **Quality of evidence** | **Stroke** | **Acute** |  | Inconsistent | Inconsistent | Inconsistent | Inconsistent | Inconsistent | Inconsistent | Moderate (-1) | Inconsistent |
| Upper Limb-Motor Assessment Scale (UL-MAS) [12] | Stroke | Chronic | 80 | + | ? | + | ? | ? | ? | + | ? |
| Upper Limb-Motor Assessment Scale (UL-MAS) [14] | Stroke | Chronic |  | + | + | + | + | + | ? | + | + |
| **Overall rating** | **Stroke** | **Chronic** |  | + | + | + | + | + | ? | + | + |
| **Quality of evidence** | **Stroke** | **Chronic** |  | Moderate (-1) | Low (-2) | Moderate (-1) | Low (-2) | Low (-2) | Inconsistent | Moderate (-1) | Low (-2) |
| Van Lieshout Test Short Form [18] | Stroke | Chronic | 60 | ? | ? | ? | ? | ? | ? | ? | - |
| Van Lieshout Test Short Form [18] | Stroke | Chronic | 30 | ? | ? | ? | - | ? | ? | ? | - |
| **Overall rating** | **Stroke** | **Chronic** |  | ? | ? | ? | - | ? | ? | ? | - |
| **Quality of evidence** | **Stroke** | **Chronic** |  | Inconsistent | Inconsistent | Inconsistent | Low (-2) | Inconsistent | Inconsistent | Inconsistent | Moderate (-1) |
| Wolf Motor Function Test (WMFT) [13] | Stroke | Chronic |  | ? | ? | ? | ? | ? | ? | ? | ? |
| Wolf Motor Function Test (WMFT) [7] | Stroke | Chronic |  | ? | + | + | + | ? | ? | - | + |
| Wolf Motor Function Test (WMFT) [12] | Stroke | Chronic | 189 | + | + | + | ? | ? | ? | + | ? |
| Wolf Motor Function Test (WMFT) [14] | Stroke | Chronic |  | ? | + | + | + | ? | ? | - | ? |
| Wolf Motor Function Test (WMFT) [15] | Stroke | Chronic |  | ? | ? | ? | ? | ? | ? | + | ? |
| Wolf Motor Function Test (WMFT) [18] | Stroke | Chronic | 24 | ? | + | + | + | ? | ? | ? | ? |
| Wolf Motor Function Test (WMFT) [16] | Stroke | Chronic |  | ? | ? | + | + | ? | ? | - | + |
| **Overall rating** | **Stroke** | **Chronic** |  | + | + | + | + | ? | ? | + | + |
| **Quality of evidence** | **Stroke** | **Chronic** |  | High | High | High | Low (-2) | Inconsistent | Inconsistent | High | Low (-2) |
| Motor-free Visual Perception Test (MVPT) [24] | Stroke | Chronic | 30 | ? | - | + | ? | ? | ? | + | ? |
| Motor Free Visual Perception Test [7] | Stroke | Chronic |  | ? | ? | + | ? | ? | ? | - | ? |
| **Overall rating** | **Stroke** | **Chronic** |  | ? | - | + | ? | ? | ? | + | ? |
| **Quality of evidence** | **Stroke** | **Chronic** |  | Inconsistent | Low (-2) | Low (-2) | Inconsistent | Inconsistent | Inconsistent | Low (-2) | Inconsistent |
| IDEEA-the Intelligent Device for Energy Expenditure and Activity [25] | Stroke | Chronic | 42 | ? | ? | - | ? | ? | ? | ? | ? |
| Intelligent Device for Energy Expenditure and Activity [21] | Stroke | Chronic | 6 | ? | ? | + | ? | ? | ? | ? | ? |
| **Overall rating** | **Stroke** | **Chronic** |  | ? | ? | + | ? | ? | ? | ? | ? |
| **Quality of evidence** | **Stroke** | **Chronic** |  | Inconsistent | Inconsistent | Low (-2) | Inconsistent | Inconsistent | Inconsistent | Inconsistent | Inconsistent |
| Assessment of Life Habits (LIFE-H) [20] | Stroke | Chronic | 84 | + | ? | + | ? | ? | ? | + | ? |
| Assessment of Life Habits (LIFE-H) [28] | Stroke | Chronic | 80 | ? | ? | + | ? | ? | ? | ? | ? |
| **Overall rating** | **Stroke** | **Chronic** |  | + | ? | + | ? | ? | ? | + | ? |
| **Quality of evidence** | **Stroke** | **Chronic** |  | Moderate (-1) | Inconsistent | High | Inconsistent | Inconsistent | Inconsistent | Moderate (-1) | Inconsistent |
| **Brain injury community rehabilitation outcome scale (BICRO) [33]** | BI |  | 127 | + | + | + | + | ? | ? | + | ? |
| **Overall rating** | **BI** |  | 127 | + | + | + | + | ? | ? | + | ? |
| **Quality of evidence** | **BI** |  |  | High | High | High | High | Inconsistent | Inconsistent | High | Inconsistent |
| **DASH (Disabilities of the Arm, Shoulder and Hand) [12]** | **Stroke** | Chronic | 300 | ? | + | + | ? | ? | ? | + | ? |
| **Overall rating** | **Stroke** | Chronic | 300 | ? | + | + | ? | ? | ? | + | ? |
| **Quality of evidence** | **Stroke** | Chronic |  | Inconsistent | High | High | Inconsistent | Inconsistent | Inconsistent | High | Inconsistent |
| **6-item Short Form Postural Assessment Scale for Stroke Patients (6 SFPASS) [1]** | Stroke | Acute | 262 | ? | ? | ? | ? | ? | ? | ? | + |
| **Overall rating** | **Stroke** | **Acute** | 262 | ? | ? | ? | ? | ? | ? | ? | + |
| **Quality of evidence** | **Stroke** | **Acute** |  | Inconsistent | Inconsistent | Inconsistent | Inconsistent | Inconsistent | Inconsistent | Inconsistent | High |
| **Kinematics [16]** | Stroke | Chronic | 8 | ? | ? | + | ? | ? | ? | - | + |
| **Overall rating** | **Stroke** | **Chronic** | 8 | ? | ? | + | ? | ? | ? | - | + |
| **Quality of evidence** | **Stroke** | **Chronic** |  | Inconsistent | Inconsistent | Low (-2) | Inconsistent | Inconsistent | Inconsistent | Low (-2) | Low (-2) |
| **Postural Control and Balance for Stroke (PCBS) [1]** | Stroke | Acute | 50 | ? | ? | ? | ? | ? | ? | ? | + |
| **Overall rating** | **Stroke** | **Acute** | 50 | ? | ? | ? | ? | ? | ? | ? | + |
| **Quality of evidence** | **Stroke** | **Acute** |  | Inconsistent | Inconsistent | Inconsistent | Inconsistent | Inconsistent | Inconsistent | Inconsistent | Moderate (-1) |
| **Short Form Berg Balance Scale (SFBBS) [1]** | Stroke | Acute | 81 | ? | ? | ? | ? | ? | ? | ? | + |
| **Overall rating** | **Stroke** | **Acute** | 81 | ? | ? | ? | ? | ? | ? | ? | + |
| **Quality of evidence** | **Stroke** | **Acute** |  | Inconsistent | Inconsistent | Inconsistent | Inconsistent | Inconsistent | Inconsistent | Inconsistent | Moderate (-1) |
| **Smart Balance Master (SBM) [1]** | Stroke | Acute | 40 | ? | ? | ? | ? | ? | ? | ? | + |
| **Overall rating** | **Stroke** | **Acute** | 40 | ? | ? | ? | ? | ? | ? | ? | + |
| **Quality of evidence** | **Stroke** | **Acute** |  | Inconsistent | Inconsistent | Inconsistent | Inconsistent | Inconsistent | Inconsistent | Inconsistent | Low (-2) |
| **300mWT (Three hundred metre Walk Test in community) [5]** | Stroke | Chronic | 28 | + | ? | + | ? | ? | ? | + | ? |
| **Overall rating** | **Stroke** | **Chronic** | 28 | + | ? | + | ? | ? | ? | + | ? |
| **Quality of evidence** | **Stroke** | **Chronic** |  | Low (-2) | Inconsistent | Low (-2) | Inconsistent | Inconsistent | Inconsistent | Low (-2) | Inconsistent |
| **30mCWT (Thirty metre Comfortable Walk Test) [5]** | Stroke | Chronic | 18 | + | ? | ? | ? | ? | ? | + | ? |
| **Overall rating** | **Stroke** | **Chronic** | 18 | + | ? | ? | ? | ? | ? | + | ? |
| **Quality of evidence** | **Stroke** | **Chronic** |  | Low (-2) | Inconsistent | Inconsistent | Inconsistent | Inconsistent | Inconsistent | Low (-2) | Inconsistent |
| **4mCWT (Four metre Comfortable Walk Test) [5]** | Stroke | Chronic | 25 | + | ? | ? | ? | ? | ? | + | ? |
| **Overall rating** | **Stroke** | **Chronic** | 25 | + | ? | ? | ? | ? | ? | + | ? |
| **Quality of evidence** | **Stroke** | **Chronic** |  | Low (-2) | Inconsistent | Inconsistent | Inconsistent | Inconsistent | Inconsistent | Low (-2) | Inconsistent |
| **Actical [25]** | Stroke | Chronic | 40 | ? | ? | + | ? | ? | ? | ? | ? |
| **Overall rating** | **Stroke** | **Chronic** | 40 | ? | ? | + | ? | ? | ? | ? | ? |
| **Quality of evidence** | **Stroke** | **Chronic** |  | Inconsistent | Inconsistent | Low (-2) | Inconsistent | Inconsistent | Inconsistent | Inconsistent | Inconsistent |
| **Activities of Daily Living observation [13]** | Stroke | Chronic | 81 | ? | ? | ? | + | ? | ? | + | ? |
| **Overall rating** | **Stroke** | **Chronic** | 81 | ? | ? | ? | + | ? | ? | + | ? |
| **Quality of evidence** | **Stroke** | **Chronic** |  | Inconsistent | Inconsistent | Moderate (-1) | Moderate (-1) | Inconsistent | Inconsistent | Moderate (-1) | Inconsistent |
| **Actual Amount of Use Test (AAUT) [13]** | Stroke | Chronic | 11 | ? | ? | + | ? | ? | ? | + | ? |
| **Overall rating** | **Stroke** | **Chronic** | 11 | ? | ? | + | ? | ? | ? | + | ? |
| **Quality of evidence** | **Stroke** | **Chronic** |  | Inconsistent | Inconsistent | Low (-2) | Inconsistent | Inconsistent | Inconsistent | Low (-2) | Inconsistent |
| **Assessment of Motor and Process Skills (AMPS) [13]** | Stroke | Chronic | 76 | ? | ? | + | + | ? | ? | + | ? |
| **Overall rating** | **Stroke** | **Chronic** | 76 | ? | ? | + | + | ? | ? | + | ? |
| **Quality of evidence** | **Stroke** | **Chronic** |  | Inconsistent | Inconsistent | Moderate (-1) | Moderate (-1) | Inconsistent | Inconsistent | Moderate (-1) | Inconsistent |
| **Balance Assessment in Sitting and Standing Position (BASSP) [34]** | Stroke | Chronic | 1193 | ? | ? | ? | ? | + | ? | + | + |
| **Overall rating** | **Stroke** | **Chronic** | 1193 | ? | ? | ? | ? | + | ? | + | + |
| **Quality of evidence** | **Stroke** | **Chronic** |  | Inconsistent | Inconsistent | Inconsistent | Inconsistent | High | Inconsistent | High | High |
| **Balance Evaluation System test (Bentest) [26]** | Stroke | Chronic | 115 | + | ? | + | ? | ? | ? | + | + |
| **Overall rating** | **Stroke** | **Chronic** | 115 | + | ? | + | ? | ? | ? | + | + |
| **Quality of evidence** | **Stroke** | **Chronic** |  | High | Inconsistent | High | Inconsistent | Inconsistent | Inconsistent | High | High |
| **Biaxial accelerometer [21]** | Stroke | Chronic | 6 | ? | ? | + | ? | ? | ? | + | ? |
| **Overall rating** | **Stroke** | **Chronic** | 6 | ? | ? | + | ? | ? | ? | + | ? |
| **Quality of evidence** | **Stroke** | **Chronic** |  | Inconsistent | Inconsistent | Low (-2) | Inconsistent | Inconsistent | Inconsistent | Low (-2) | Inconsistent |
| **Brunel Balance Assessment [26]** | Stroke | Chronic | 92 | + | ? | + | ? | ? | ? | + | ? |
| **Overall rating** | **Stroke** | **Chronic** | 92 | + | ? | + | ? | ? | ? | + | ? |
| **Quality of evidence** | **Stroke** | **Chronic** |  | Moderate (-1) | Inconsistent | Moderate (-1) | Inconsistent | Inconsistent | Inconsistent | Moderate (-1) | Inconsistent |
| **Canadian Occupational Performance Measure (COPM) [13]** | Stroke | Chronic | 26 | ? | ? | + | ? | ? | ? | + | + |
| **Overall rating** | **Stroke** | **Chronic** | 26 | ? | ? | + | ? | ? | ? | + | + |
| **Quality of evidence** | **Stroke** | **Chronic** |  | Inconsistent | Inconsistent | Low (-2) | Inconsistent | Inconsistent | Inconsistent | Low (-2) | Low (-2) |
| **Centre for Epidemiological Studies Depression [7]** | Stroke | Chronic | 27 | ? | + | + | + | ? | ? | + | + |
| **Overall rating** | **Stroke** | **Chronic** | 27 | ? | + | + | + | ? | ? | + | + |
| **Quality of evidence** | **Stroke** | **Chronic** |  | Inconsistent | Low (-2) | Low (-2) | Low (-2) | Inconsistent | Inconsistent | Low (-2) | Low (-2) |
| **Climbing stairs questionnaire (CSQ) [33]** | Stroke | Chronic | 15 | + | + | + | + | ? | ? | - | ? |
| **Overall rating** | **Stroke** | **Chronic** | 15 | + | + | + | + | ? | ? | - | ? |
| **Quality of evidence** | **Stroke** | **Chronic** |  | Low (-2) | Low (-2) | Low (-2) | Low (-2) | Inconsistent | Inconsistent | Low (-2) | Inconsistent |
| **Coded activity diary [19]** | Stroke | Chronic | 16 | ? | ? | ? | ? | ? | ? | + | ? |
| **Overall rating** | **Stroke** | **Chronic** | 16 | ? | ? | ? | ? | ? | ? | + | ? |
| **Quality of evidence** | **Stroke** | **Chronic** |  | Inconsistent | Inconsistent | Inconsistent | Inconsistent | Inconsistent | Inconsistent | Low (-2) | Inconsistent |
| **Computer Science and Applications Inc. Model 7164 activity monitors x 4 [25]** | Stroke | Chronic | 9 | ? | ? | ? | ? | ? | ? | + | ? |
| **Overall rating** | **Stroke** | **Chronic** | 9 | ? | ? | ? | ? | ? | ? | + | ? |
| **Quality of evidence** | **Stroke** | **Chronic** |  | Inconsistent | Inconsistent | Inconsistent | Inconsistent | Inconsistent | Inconsistent | Low (-2) | Inconsistent |
| **Dimensional gait analysis (3-DGA) [21]** | Stroke | Chronic | 25 | ? | ? | ? | ? | ? | ? | + | ? |
| **Overall rating** | **Stroke** | **Chronic** | 25 | ? | ? | ? | ? | ? | ? | + | ? |
| **Quality of evidence** | **Stroke** | **Chronic** |  | Inconsistent | Inconsistent | Inconsistent | Inconsistent | Inconsistent | Inconsistent | Low (-2) | Inconsistent |
| **Duruoz Hand Index (DHI) [13]** | Stroke | Chronic | 56 | ? | ? | + | ? | ? | ? | + | ? |
| **Overall rating** | **Stroke** | **Chronic** | 56 | ? | ? | + | ? | ? | ? | + | ? |
| **Quality of evidence** | **Stroke** | **Chronic** |  | Inconsistent | Inconsistent | Moderate (-1) | Inconsistent | Inconsistent | Inconsistent | Moderate (-1) | Inconsistent |
| **Functional Ambulation Classification Hospital (FACHS) [5]** | Stroke | Chronic | 31 | + | ? | ? | ? | ? | ? | + | ? |
| **Overall rating** | **Stroke** | **Chronic** | 31 | + | ? | ? | ? | ? | ? | + | ? |
| **Quality of evidence** | **Stroke** | **Chronic** |  | Low (-2) | Inconsistent | Inconsistent | Inconsistent | Inconsistent | Inconsistent | Low (-2) | Inconsistent |
| **Functional Gait Assessment (FGA) [5]** | Stroke | Chronic | 28 | + | ? | ? | + | + | ? | + | ? |
| **Overall rating** | **Stroke** | **Chronic** | 28 | + | ? | ? | + | + | ? | + | ? |
| **Quality of evidence** | **Stroke** | **Chronic** |  | Low (-2) | Inconsistent | Inconsistent | Low (-2) | Low (-2) | Inconsistent | Low (-2) | Inconsistent |
| **Finger Tapping (uniaxial accelerometer) [21]** | Stroke | Chronic | 60 | ? | ? | ? | ? | ? | ? | + | ? |
| **Overall rating** | **Stroke** | **Chronic** | 60 | ? | ? | ? | ? | ? | ? | + | ? |
| **Quality of evidence** | **Stroke** | **Chronic** |  | Inconsistent | Inconsistent | Inconsistent | Inconsistent | Inconsistent | Inconsistent | Moderate (-1) | Inconsistent |
| **Fitbit Ulta [25]** | Stroke | Chronic | 30 | ? | ? | ? | ? | ? | ? | - | + |
| **Overall rating** | **Stroke** | **Chronic** | 30 | ? | ? | ? | ? | ? | ? | - | + |
| **Quality of evidence** | **Stroke** | **Chronic** |  | Inconsistent | Inconsistent | Inconsistent | Inconsistent | Inconsistent | Inconsistent | Low (-2) | Low (-2) |
| **Fitts Reaching test [11]** | Stroke | Chronic | 18 | ? | ? | + | ? | ? | ? | + | ? |
| **Overall rating** | **Stroke** | **Chronic** | 18 | ? | ? | + | ? | ? | ? | + | ? |
| **Quality of evidence** | **Stroke** | **Chronic** |  | Inconsistent | Inconsistent | Low (-2) | Inconsistent | Inconsistent | Inconsistent | Low (-2) | Inconsistent |
| **Fugl-Meyer Assessment-Upper extremity [12]** | Stroke | Chronic | 512 | + | ? | + | ? | ? | ? | + | ? |
| **Overall rating** | **Stroke** | **Chronic** | 512 | + | ? | + | ? | ? | ? | + | ? |
| **Quality of evidence** | **Stroke** | **Chronic** |  | High | Inconsistent | High | Inconsistent | Inconsistent | Inconsistent | High | Inconsistent |
| **Four Square Step [26]** | Stroke | Chronic | 37 | - | ? | ? | ? | ? | ? | + | + |
| **Overall rating** | **Stroke** | **Chronic** | 37 | - | ? | ? | ? | ? | ? | + | + |
| **Quality of evidence** | **Stroke** | **Chronic** |  | Low (-2) | Inconsistent | Inconsistent | Inconsistent | Inconsistent | Inconsistent | Low (-2) | Low (-2) |
| **Function in Sitting Test (FIST) [34]** | Stroke | Acute | 31 | + | + | ? | ? | ? | + | + | ? |
| **Overall rating** | **Stroke** | **Acute** | 31 | + | + | ? | ? | ? | + | + | ? |
| **Quality of evidence** | **Stroke** | **Acute** |  | Low (-2) | Low (-2) | Inconsistent | Inconsistent | Inconsistent | Low (-2) | Low (-2) | Inconsistent |
| **Functional Arm Activity Behavioral Observation System (FAABOS) [13]** | ABI |  | 9 | ? | ? | ? | + | ? | ? | ? | ? |
| **Overall rating** | **ABI** |  | 9 | ? | ? | ? | + | ? | ? | ? | ? |
| **Quality of evidence** | **ABI** |  |  | Inconsistent | Inconsistent | Inconsistent | Low (-2) | Inconsistent | Inconsistent | Inconsistent | Inconsistent |
| **Functional Test for the Hemiplegic Upper Extremity (FTHUE) [13]** | Stroke | Chronic | 82 | ? | ? | ? | + | ? | ? | ? | ? |
| **Overall rating** | **Stroke** | **Chronic** | 82 | ? | ? | ? | + | ? | ? | ? | ? |
| **Quality of evidence** | **Stroke** | **Chronic** |  | Inconsistent | Inconsistent | Inconsistent | Moderate (-1) | Inconsistent | Inconsistent | Inconsistent | Inconsistent |
| **Geriatric Depression scale-long form (GDS) [7]** | Stroke | Chronic |  | ? | + | + | ? | ? | ? | + | + |
| **Overall rating** | **Stroke** | **Chronic** |  | ? | + | + | ? | ? | ? | + | + |
| **Quality of evidence** | **Stroke** | **Chronic** |  | Inconsistent | Low (-2) | Low (-2) | Inconsistent | Inconsistent | Inconsistent | Low (-2) | Low (-2) |
| **Grip strength [16]** | Stroke | Chronic | 27 | ? | ? | + | + | ? | ? | ? | ? |
| **Overall rating** | **Stroke** | **Chronic** | 27 | ? | ? | + | + | ? | ? | ? | ? |
| **Quality of evidence** | **Stroke** | **Chronic** |  | Inconsistent | Inconsistent | Low (-2) | Low (-2) | Inconsistent | Inconsistent | Inconsistent | Inconsistent |
| **Hand Function Survey (HFS) [13]** | Stroke | Chronic | 45 | ? | ? | + | ? | ? | ? | + | ? |
| **Overall rating** | **Stroke** | **Chronic** | 45 | ? | ? | + | ? | ? | ? | + | ? |
| **Quality of evidence** | **Stroke** | **Chronic** |  | Inconsistent | Inconsistent | Low (-2) | Inconsistent | Inconsistent | Inconsistent | Low (-2) | Inconsistent |
| **International classification of functioning, health and disability-Activity measure (ICF-AM) [12]** | Stroke | Chronic | 317 | ? | ? | ? | + | ? | ? | + | ? |
| **Overall rating** | **Stroke** | **Chronic** | 317 | ? | ? | ? | + | ? | ? | + | ? |
| **Quality of evidence** | **Stroke** | **Chronic** |  | Inconsistent | Inconsistent | Inconsistent | High | Inconsistent | Inconsistent | High | Inconsistent |
| **Motor Activity Log-28 items [9]** | Stroke | Sub-acute | 222 | ? | + | ? | ? | ? | ? | - | ? |
| **Overall rating** | **Stroke** | **Sub-acute** | 222 | ? | + | ? | ? | ? | ? | - | ? |
| **Quality of evidence** | **Stroke** | **Sub-acute** |  | Inconsistent | High | Inconsistent | Inconsistent | Inconsistent | Inconsistent | Moderate (-1) | Inconsistent |
| **Manual Function Test (MFT) [13]** | Stroke | Acute | 51 | ? | + | + | ? | ? | ? | + | ? |
| **Overall rating** | **Stroke** | **Acute** | 51 | ? | + | + | ? | ? | ? | + | ? |
| **Quality of evidence** | **Stroke** | **Acute** |  | Inconsistent | Moderate (-1) | Moderate (-1) | Inconsistent | Inconsistent | Inconsistent | Moderate (-1) | Inconsistent |
| **Multimedia activity recall for children and adults (MARCA) [19]** | Stroke | Chronic | 40 | ? | ? | + | ? | ? | ? | + | ? |
| **Overall rating** | **Stroke** | **Chronic** | 40 | ? | ? | + | ? | ? | ? | + | ? |
| **Quality of evidence** | **Stroke** | **Chronic** |  | Inconsistent | Inconsistent | Low (-2) | Inconsistent | Inconsistent | Inconsistent | Low (-2) | Inconsistent |
| **National Institute of Health Stroke Scale [7]** | Stroke | Chronic | 65 | ? | ? | + | ? | ? | ? | + | - |
| **Overall rating** | **Stroke** | **Chronic** | 65 | ? | ? | + | ? | ? | ? | + | - |
| **Quality of evidence** | **Stroke** | **Chronic** |  | Inconsistent | Inconsistent | Moderate (-1) | Inconsistent | Inconsistent | Inconsistent | Moderate (-1) | Moderate (-1) |
| **Neurobehavioral Cognition Status Exam (NCSE) [7]** | Stroke | Chronic |  | ? | ? | - | - | ? | ? | + | + |
| **Overall rating** | **Stroke** | **Chronic** |  | ? | ? | - | - | ? | ? | + | + |
| **Quality of evidence** | **Stroke** | **Chronic** |  | Inconsistent | Inconsistent | Low (-2) | Low (-2) | Inconsistent | Inconsistent | Low (-2) | Low (-2) |
| **Nike+Fuelband [25]** | Stroke | Chronic | 30 | ? | ? | ? | ? | ? | ? | - | + |
| **Overall rating** | **Stroke** | **Chronic** | 30 | ? | ? | ? | ? | ? | ? | - | + |
| **Quality of evidence** | **Stroke** | **Chronic** |  | Inconsistent | Inconsistent | Inconsistent | Inconsistent | Inconsistent | Inconsistent | Low (-2) | Low (-2) |
| **Nottingham Extended ADL index (N-ADL) [33]** | Stroke | Chronic | 78 | + | + | + | + | ? | ? | + | + |
| **Overall rating** | **Stroke** | **Chronic** | 78 | + | + | + | + | ? | ? | + | + |
| **Quality of evidence** | **Stroke** | **Chronic** |  | Moderate (-1) | Moderate (-1) | Moderate (-1) | Moderate (-1) | Inconsistent | Inconsistent | Moderate (-1) | Moderate (-1) |
| **OPTIMAL (Outpatient Physical Therapy Improvement in Movement Assessment Log) [12]** | Stroke | Chronic | 3138 | ? | ? | ? | ? | ? | ? | + | ? |
| **Overall rating** | **Stroke** | **Chronic** | 3138 | ? | ? | ? | ? | ? | ? | + | ? |
| **Quality of evidence** | **Stroke** | **Chronic** |  | Inconsistent | Inconsistent | Inconsistent | Inconsistent | Inconsistent | Inconsistent | High | Inconsistent |
| **Ottawa Sitting Scale (OSS) [34]** | Stroke | Chronic | 71 | ? | ? | ? | + | + | ? | ? | ? |
| **Overall rating** | **Stroke** | **Chronic** | 71 | ? | ? | ? | + | + | ? | ? | ? |
| **Quality of evidence** | **Stroke** | **Chronic** |  | Inconsistent | Inconsistent | Moderate (-1) | Inconsistent | Inconsistent | Inconsistent | Inconsistent | Inconsistent |
| **PAL2 (Gorman ProMed Pty. Ltd) [25]** | Stroke | Chronic | 20 | ? | ? | ? | ? | ? | ? | - | ? |
| **Overall rating** | **Stroke** | **Chronic** | 20 | ? | ? | ? | ? | ? | ? | - | ? |
| **Quality of evidence** | **Stroke** | **Chronic** |  | Inconsistent | Inconsistent | Inconsistent | Inconsistent | Inconsistent | Inconsistent | Low (-2) | Inconsistent |
| **Physical Ability Scale (PAS) [34]** | Stroke | Sub-acute | 10 | ? | ? | ? | - | - | ? | ? | ? |
| **Overall rating** | **Stroke** | **Sub-acute** | 10 | ? | ? | ? | - | - | ? | ? | ? |
| **Quality of evidence** | **Stroke** | **Sub-acute** |  | Inconsistent | Inconsistent | Inconsistent | Low (-2) | Low (-2) | Inconsistent | Inconsistent | inconsistent |
| **Quadriplegia Index of Function [18]** | Stroke | Chronic | 60 | ? | ? | + | ? | ? | ? | ? | - |
| **Overall rating** | **Stroke** | **Chronic** | 60 | ? | ? | + | ? | ? | ? | ? | - |
| **Quality of evidence** | **Stroke** | **Chronic** |  | Inconsistent | Inconsistent | Moderate (-1) | Inconsistent | Inconsistent | Inconsistent | inconsistent | Moderate (-1) |
| **Reintegration to normal living index (RNLI) [30]** | Stroke | Chronic | 57 | ? | ? | + | ? | ? | ? | + | ? |
| **Overall rating** | **Stroke** | **Chronic** | 57 | ? | ? | + | ? | ? | ? | + | ? |
| **Quality of evidence** | **Stroke** | **Chronic** |  | Inconsistent | Inconsistent | Moderate (-1) | Inconsistent | Inconsistent | Inconsistent | Moderate (-1) | Inconsistent |
| **Stroke Arm Ladder (SAL) [12]** | Stroke | Chronic | 942 | + | ? | + | ? | ? | ? | + | ? |
| **Overall rating** | **Stroke** | **Chronic** | 942 | + | ? | + | ? | ? | ? | + | ? |
| **Quality of evidence** | **Stroke** | **Chronic** |  | High | Inconsistent | High | Inconsistent | Inconsistent | Inconsistent | High | Inconsistent |
| **Sensewear Pro 3 Armband [25]** | Stroke | Chronic | 12 | ? | ? | ? | ? | ? | ? | - | + |
| **Overall rating** | **Stroke** | **Chronic** | 12 | ? | ? | ? | ? | ? | ? | - | + |
| **Quality of evidence** | **Stroke** | **Chronic** |  | Inconsistent | Inconsistent | Inconsistent | Inconsistent | Inconsistent | Inconsistent | Low (-2) | Low (-2) |
| **Sitting Rising Test (SRT) [34]** | Stroke | Chronic | 60 | ? | ? | + | + | ? | ? | + | ? |
| **Overall rating** | **Stroke** | **Chronic** | 60 | ? | ? | + | + | ? | ? | + | ? |
| **Quality of evidence** | **Stroke** | **Chronic** |  | Inconsistent | Inconsistent | Moderate (-1) | Moderate (-1) | Inconsistent | Inconsistent | Moderate (-1) | Inconsistent |
| **SmartShoe [25]** | Stroke | Chronic | 12 | ? | ? | ? | ? | ? | ? | + | + |
| **Overall rating** | **Stroke** | **Chronic** | 12 | ? | ? | ? | ? | ? | ? | + | + |
| **Quality of evidence** | **Stroke** | **Chronic** |  | Inconsistent | Inconsistent | Inconsistent | Inconsistent | Inconsistent | Inconsistent | Low (-2) | Low (-2) |
| **Sodring motor evaluation for stroke patients [32]** | Stroke | Chronic | 123 | ? | + | ? | + | ? | ? | + | + |
| **Overall rating** | **Stroke** | **Chronic** | 123 | ? | + | ? | + | ? | ? | + | + |
| **Quality of evidence** | **Stroke** | **Chronic** |  | Inconsistent | High | Inconsistent | High | Inconsistent | Inconsistent | High | High |
| **Sollerman hand function test [11]** | Stroke | Chronic | 24 | ? | ? | + | + | ? | ? | ? | ? |
| **Overall rating** | **Stroke** | **Chronic** | 24 | ? | ? | + | + | ? | ? | ? | ? |
| **Quality of evidence** | **Stroke** | **Chronic** |  | Inconsistent | Inconsistent | Low (-2) | Low (-2) | Inconsistent | Inconsistent | Inconsistent | Inconsistent |
| **Step test [26]** | Stroke | Chronic | 41 | + | ? | + | ? | ? | ? | + | + |
| **Overall rating** | **Stroke** | **Chronic** | 41 | + | ? | + | ? | ? | ? | + | + |
| **Quality of evidence** | **Stroke** | **Chronic** |  | Low (-2) | Inconsistent | Low (-2) | Inconsistent | Inconsistent | Inconsistent | Low (-2) | Low (-2) |
| **Stride analyzer system (SAS) [21]** | Stroke | Chronic | 6 | ? | ? | + | ? | ? | ? | + | ? |
| **Overall rating** | **Stroke** | **Chronic** | 6 | ? | ? | + | ? | ? | ? | + | ? |
| **Quality of evidence** | **Stroke** | **Chronic** |  | Inconsistent | Inconsistent | Low (-2) | Inconsistent | Inconsistent | Inconsistent | Low (-2) | Inconsistent |
| **Subjective index of physical and social outcome (SIPSO) [30]** | Stroke | Chronic | 260 | ? | ? | ? | ? | ? | ? | ? | ? |
| **Overall rating** | **Stroke** | **Chronic** | 260 | ? | ? | ? | ? | ? | ? | ? | ? |
| **Quality of evidence** | **Stroke** | **Chronic** |  | Inconsistent | Inconsistent | Inconsistent | Inconsistent | Inconsistent | Inconsistent | Inconsistent | Inconsistent |
| **Timed walk [23]** | Stroke | Chronic | 22 | + | ? | ? | ? | + | ? | + | + |
| **Overall rating** | **Stroke** | **Chronic** | 22 | + | ? | ? | ? | + | ? | + | + |
| **Quality of evidence** | **Stroke** | **Chronic** |  | Low (-2) | Inconsistent | Low (-2) | Inconsistent | Low (-2) | Inconsistent | Low (-2) | Low (-2) |
| **Trunk Recovery Scale (TRS) [34]** | BI |  | 59 | ? | + | ? | + | ? | + | + | ? |
| **Overall rating** | **BI** |  | 59 | ? | + | ? | + | ? | + | + | ? |
| **Quality of evidence** | **BI** |  |  | Inconsistent | Moderate (-1) | Inconsistent | Moderate (-1) | Inconsistent | Moderate (-1) | Moderate (-1) | inconsistent |
| **Upper Extremity Functional Index (UEFI) [12]** | Stroke | Chronic | 239 | ? | ? | ? | + | ? | ? | + | ? |
| **Overall rating** | **Stroke** | **Chronic** | 239 | ? | ? | ? | + | ? | ? | + | ? |
| **Quality of evidence** | **Stroke** | **Chronic** |  | Inconsistent | Inconsistent | Inconsistent | High | Inconsistent | Inconsistent | High | Inconsistent |
| **Upper Body Dressing Scale (UBDS) [13]** | Stroke | Chronic | 51 | ? | ? | ? | + | ? | ? | + | + |
| **Overall rating** | **Stroke** | **Chronic** | 51 | ? | ? | ? | + | ? | ? | + | + |
| **Quality of evidence** | **Stroke** | **Chronic** |  | Inconsistent | Inconsistent | Inconsistent | Moderate (-1) | Inconsistent | Inconsistent | Moderate (-1) | Moderate (-1) |
| **Up per Extremity Performance Test for Elderly (Test d’Evaluation des Membres supérieurs de Personnes Agées (TEMPA) [13]** | Stroke | Chronic | 29 | ? | ? | + | ? | ? | ? | + | ? |
| **Overall rating** | **Stroke** | **Chronic** | 29 | ? | ? | + | ? | ? | ? | + | ? |
| **Quality of evidence** | **Stroke** | **Chronic** |  | Inconsistent | Inconsistent | Low (-2) | Inconsistent | Inconsistent | Inconsistent | Low (-2) | Inconsistent |
| **Wireless Triaxial Accelerometers [25]** | Stroke | Chronic | 12 | ? | ? | ? | ? | ? | ? | - | ? |
| **Overall rating** | **Stroke** | **Chronic** | 12 | ? | ? | ? | ? | ? | ? | - | ? |
| **Quality of evidence** | **Stroke** | **Chronic** |  | Inconsistent | Inconsistent | Inconsistent | Inconsistent | Inconsistent | Inconsistent | Low (-2) | Inconsistent |
| **3MWT [8]** | Stroke | Sub-acute | 14 | + | ? | ? | ? | + | ? | + | ? |
| **Overall rating** | **Stroke** | **Sub-acute** | 14 | + | ? | ? | ? | + | ? | + | ? |
| **Quality of evidence** | **Stroke** | **Sub-acute** |  | Low (-2) | Inconsistent | Inconsistent | Inconsistent | Low (-2) | Inconsistent | Low (-2) | Inconsistent |
| **Accelerometer (ActiGraph) [21]** | Stroke | Sub-acute | 20 | ? | ? | + | ? | ? | ? | + | ? |
| **Overall rating** | **Stroke** | **Sub-acute** | 20 | ? | ? | + | ? | ? | ? | + | ? |
| **Quality of evidence** | **Stroke** | **Sub-acute** |  | Inconsistent | Inconsistent | Low (-2) | Inconsistent | Inconsistent | Inconsistent | Low (-2) | Inconsistent |
| **Grooved Pegboard Test (GPT) [27]** | TBI |  |  | ? | ? | + | ? | ? | ? | - | ? |
| **Overall rating** | **TBI** |  |  | ? | ? | + | ? | ? | ? | - | ? |
| **Quality of evidence** | **TBI** |  |  | Inconsistent | Inconsistent | Low (-2) | Inconsistent | Inconsistent | Inconsistent | Low (-2) | Inconsistent |
| **HiMAT-High Level Mobility Assessment [2]** | TBI |  | 103 | ? | ? | ? | + | + | ? | + | ? |
| **Overall rating** | **TBI** |  | 103 | ? | ? | ? | + | + | ? | + | ? |
| **Quality of evidence** | **TBI** |  |  | Inconsistent | Inconsistent | Inconsistent | High | High | Inconsistent | High | Inconsistent |
| **Mayo-Portland Adaptability Inventory (MPAI-4) [27]** | TBI |  | 339 | ? | + | ? | + | ? | ? | + | ? |
| **Overall rating** | **TBI** |  | 339 | ? | + | ? | + | ? | ? | + | ? |
| **Quality of evidence** | **TBI** |  |  | Inconsistent | High | Inconsistent | High | Inconsistent | Inconsistent | High | Inconsistent |
| **Pens taped to feet [2]** | TBI |  | 12 | ? | ? | ? | ? | + | ? | + | ? |
| **Overall rating** | **TBI** |  | 12 | ? | ? | ? | ? | + | ? | + | ? |
| **Quality of evidence** | **TBI** |  |  | Inconsistent | Inconsistent | Inconsistent | Inconsistent | Low (-2) | Inconsistent | Low (-2) | Inconsistent |
| **Satisfaction With Life Scale (SWLS) [27]** | TBI |  |  | + | ? | + | ? | ? | ? | ? | ? |
| **Overall rating** | **TBI** |  |  | + | ? | + | ? | ? | ? | ? | ? |
| **Quality of evidence** | **TBI** |  |  | Low (-2) | Inconsistent | Low (-2) | Inconsistent | Inconsistent | Inconsistent | Inconsistent | Inconsistent |

_10MWT: ten-meter walking test; 12MWT: twelve-meter walking test; 2MWT: two-meter walking test; 5MWT: five-meter walking test; 6MWT: six-minute walking test; TBI: traumatic brain injury
Overall ratings: sufficient (+), insufficient (-), inconsistent (±), or indeterminate (?)_

_Modified-GRADE approach: High, Moderate (-1), Low (-2), Very Low (-3); in which -1 level refers to serious; -2 level refers to very serious; and -3 level refer to very serious risk of bias, inconsistency, indirectness, and imprecision [36]_

_Acute rehabilitation phase refers to a duration of 24 hours after stroke onset and for medically stable patients, lasts 5–7 days [37]; Sub-acute rehabilitation phase refers to a duration of 1 to 6 months where the functional recovery and long-term health status are more affected [39]; Chronic rehabilitation phase begins once the person is discharged home [37]. We did not include the recovery phase for TBI because it was not defined clearly in the literature._


**References**

1. Scrivener, K., Sherrington, C., & Schurr, K. (2013). A systematic review of the responsiveness of lower limb physical performance measures in inpatient care after stroke. *BMC neurology, 13*(1), 4.
2. Tyson, S., & Connell, L. (2009). The psychometric properties and clinical utility of measures of walking and mobility in neurological conditions: a systematic review. *Clinical rehabilitation, 23*(11), 1018-1033.
3. Geroin, C., Mazzoleni, S., Smania, N., Gandolfi, M., Bonaiuti, D., Gasperini, G., et al. (2013). Systematic review of outcome measures of walking training using electromechanical and robotic devices in patients with stroke. *Journal of rehabilitation medicine, 45*(10), 987-996.
4. Stevens, P. M. (2010). Clinimetric properties of timed walking events among patient populations commonly encountered in orthotic and prosthetic rehabilitation. *JPO: Journal of Prosthetics and Orthotics, 22*(1), 62-74.
5. van Bloemendaal, M., van de Water, A. T., & van de Port, I. G. (2012). Walking tests for stroke survivors: a systematic review of their measurement properties. *Disability and Rehabilitation, 34*(26), 2207-2221.
6. Van Peppen, R. P., Hendriks, H., Van Meeteren, N. L., Helders, P. J., & Kwakkel, G. (2007). The development of a clinical practice stroke guideline for physiotherapists in The Netherlands: a systematic review of available evidence. *Disability and Rehabilitation, 29*(10), 767-783.
7. Barak, S., & Duncan, P. W. (2006). Issues in selecting outcome measures to assess functional recovery after stroke. *NeuroRx, 3*(4), 505-524.
8. Salbach, N. M., O'brien, K. K., Brooks, D., Irvin, E., Martino, R., Takhar, P., et al. (2017). Considerations for the selection of time-limited walk tests poststroke: a systematic review of test protocols and measurement properties. *Journal of Neurologic Physical Therapy, 41*(1), 3-17.
9. Ashford, S., Slade, M., Malaprade, F., & Turner-Stokes, L. (2008). Evaluation of functional outcome measures for the hemiparetic upper limb: a systematic review. *Journal of rehabilitation medicine, 40*(10), 787-795.
10. Baker, K., Cano, S. J., & Playford, E. D. (2011). Outcome measurement in stroke: a scale selection strategy. *Stroke, 42*(6), 1787-1794.
11. Connell, L. A., & Tyson, S. F. (2012). Clinical reality of measuring upper-limb ability in neurologic conditions: a systematic review. *Archives of physical medicine and rehabilitation, 93*(2), 221-228.
12. Hong, I., & Bonilha, H. S. (2017). Psychometric properties of upper extremity outcome measures validated by Rasch analysis: a systematic review. *International Journal of Rehabilitation Research, 40*(1), 1-10.
13. Lemmens, R. J., Timmermans, A. A., Janssen-Potten, Y. J., Smeets, R. J., & Seelen, H. A. (2012). Valid and reliable instruments for arm-hand assessment at ICF activity level in persons with hemiplegia: a systematic review. *BMC neurology, 12*(1), 21.
14. Rowland, T. J., & Gustafsson, L. (2008). Assessments of upper limb ability following stroke: a review. *British Journal of Occupational Therapy, 71*(10), 427-437.
15. Simpson, L. A., & Eng, J. J. (2013). Functional recovery following stroke: capturing changes in upper-extremity function. *Neurorehabilitation and neural repair, 27*(3), 240-250.
16. Sivan, M., O'Connor, R. J., Makower, S., Levesley, M., & Bhakta, B. (2011). Systematic review of outcome measures used in the evaluation of robot-assisted upper limb exercise in stroke. *Journal of Rehabilitation Medicine, 43*(3), 181-189.
17. Croarkin, E., Danoff, J., & Barnes, C. (2004). Evidence-based rating of upper-extremity motor function tests used for people following a stroke. *Physical therapy, 84*(1), 62-74.
18. Velstra, I.-M., Ballert, C. S., & Cieza, A. (2011). A systematic literature review of outcome measures for upper extremity function using the international classification of functioning, disability, and health as reference. *PM&R, 3*(9), 846-860.
19. Martins, J. C., Aguiar, L. T., Nadeau, S., Scianni, A. A., Teixeira-Salmela, L. F., & Faria, C. D. C. D. M. (2019). Measurement properties of self-report physical activity assessment tools for patients with stroke: a systematic review. *Brazilian journal of physical therapy, 23*(6), 476-490.
20. Tse, T., Douglas, J., Lentin, P., & Carey, L. (2013). Measuring participation after stroke: a review of frequently used tools. *Archives of physical medicine and rehabilitation, 94*(1), 177-192.
21. Gebruers, N., Vanroy, C., Truijen, S., Engelborghs, S., & De Deyn, P. P. (2010). Monitoring of physical activity after stroke: a systematic review of accelerometry-based measures. *Archives of physical medicine and rehabilitation, 91*(2), 288-297.
22. Salter, K., Jutai, J., Teasell, R., Foley, N., Bitensky, J., & Bayley, M. (2005). Issues for selection of outcome measures in stroke rehabilitation: ICF activity. *Disability and Rehabilitation, 27*(6), 315-340.
23. Pearson, O. R., Busse, M., Van Deursen, R. W. M., & Wiles, C. M. (2004). Quantification of walking mobility in neurological disorders. *Qjm, 97*(8), 463-475.
24. Salter, K., Jutai, J., Teasell, R., Foley, N., & Bitensky, J. (2005). Issues for selection of outcome measures in stroke rehabilitation: ICF Body Functions. *Disability and Rehabilitation, 27*(4), 191-207.
25. Fini, N. A., Holland, A. E., Keating, J., Simek, J., & Bernhardt, J. (2015). How is physical activity monitored in people following stroke? *Disability and Rehabilitation, 37*(19), 1717-1731.
26. Pollock, C., Eng, J., & Garland, S. (2011). Clinical measurement of walking balance in people post stroke: a systematic review. *Clinical rehabilitation, 25*(8), 693-708.
27. Wilde, E. A., Whiteneck, G. G., Bogner, J., Bushnik, T., Cifu, D. X., Dikmen, S., et al. (2010). Recommendations for the use of common outcome measures in traumatic brain injury research. *Archives of physical medicine and rehabilitation, 91*(11), 1650-1660. e1617.
28. Oczkowski, C., & O'Donnell, M. (2010). Reliability of proxy respondents for patients with stroke: a systematic review. *Journal of Stroke and Cerebrovascular Diseases, 19*(5), 410-416.
29. Salter, K., Jutai, J., Teasell, R., Foley, N., Bitensky, J., & Bayley, M. (2005). Issues for selection of outcome measures in stroke rehabilitation: ICF Participation. *Disability and Rehabilitation, 27*(9), 507-528.
30. Teale, E. A., & Young, J. B. (2010). A review of stroke outcome measures valid and reliable for administration by postal survey. *Reviews in Clinical Gerontology, 20*(4), 338-353.
31. Silva, P. F., Quintino, L. F., Franco, J., & Faria, C. D. (2014). Measurement properties and feasibility of clinical tests to assess sit-to-stand/stand-to-sit tasks in subjects with neurological disease: a systematic review. *Brazilian journal of physical therapy, 18*(2), 99-110.
32. Gor-García-Fogeda, M. D., Molina-Rueda, F., Cuesta-Gómez, A., Carratalá-Tejada, M., Alguacil-Diego, I. M., & Miangolarra-Page, J. C. (2014). Scales to assess gross motor function in stroke patients: a systematic review. *Archives of physical medicine and rehabilitation, 95*(6), 1174-1183.
33. Ashford, S., Brown, S., & Turner-Stokes, L. (2015). Systematic review of patient-reported outcome measures for functional performance in the lower limb. *Journal of rehabilitation medicine, 47*(1), 9-17.
34. Sorrentino G., S. P., Solaro C., Rabini A., Cerri C., Ferriero G. (2018). Clinical measurement tools to assess trunk performance after stroke: a systematic review. *European journal of physical and rehabilitation medicine*.
35. Verheyden, G., Nieuwboer, A., Van de Winckel, A., & De Weerdt, W. (2007). Clinical tools to measure trunk performance after stroke: a systematic review of the literature. *Clinical rehabilitation, 21*(5), 387-394.
36. Prinsen, C., Mokkink, L., Bouter, L., Alonso, J., Patrick, D., de Vet, H., et al. (2018). COSMIN guideline for systematic reviews of patient-reported outcome measures. Quality of Life Research, 27(5), 1147-1157.
37. Richards, C. L., Malouin, F., & Nadeau, S. (2015). Stroke rehabilitation: clinical picture, assessment, and therapeutic challenge. Progress in brain research, 218, 253-280.
38. García-Rudolph, A., Sánchez-Pinsach, D., Salleras, E. O., & Tormos, J. M. (2019). Subacute stroke physical rehabilitation evidence in activities of daily living outcomes: a systematic review of meta-analyses of randomized controlled trials. Medicine, 98(8).
