# Supplementary material for: Quality of mobility measures among individuals with acquired brain injury: an umbrella review
Source: Qual Life Res. 2022 Mar 11;31(9):2567–99. doi: 10.1007/s11136-022-03103-4 (PMC9356944; doi:10.1007/s11136-022-03103-4)
Supplement: Supplementary file 7 — Supplementary file7 (DOCX 73 kb) [file 11136_2022_3103_MOESM7_ESM.docx]

**Mobility Measures among Individuals with Acquired Brain Injury: An Umbrella Review**

Rehab Alhasani, MSc,^1,2,6^ Cluadine Auger, PhD,^2,4,5^ Matheus de Paiva Azevedo, BSc,^1^ Sara Ahmed, PhD ^1-3^

**Author affiliations:**

1. School of Physical and Occupation Therapy, Faculty of Medicine, McGill University, Montreal, Canada
2. Centre de Recherche Interdisciplinaire en Réadaptation (CRIR), Montreal, Canada
3. Constance Lethbridge Rehabilitation Center, CIUSSS Centre Ouest de l’ile de Montreal, Montreal, Canada
4. School of Rehabilitation, Faculty of Medicine, University of Montreal, Montreal, Canada
5. Site Institut Universitaire sur la Réadaptation en Déficience Physique de Montréal (IURDPM), CIUSSS Centre-Sud-de-l’Ile-de-Montréal, Montréal, Canada
6. Department of Rehabilitation Sciences, Faculty of Health and Rehabilitation Sciences, Princess Nourah bint Abdulrahman University, Riyadh, Saudi Arabia

**Corresponding author:** Sara Ahmed, PhD, School of Physical and Occupation Therapy, Faculty of Medicine, McGill University, 3655 Sir William-Osler, Montreal, QC, Canada H3G 1Y6. Tel.: 514-398-4400 ext 00531.E-mail: sara.ahmed@mcgill.ca

**Supplementary files 7: Measurement Properties**

**A. Individuals with stroke at acute Setting**

| **Name of the measure** | **SOI** | **Sample size** | **Content validity** | **Internal consistency** | **Test-retest** | **Inter-rater** | **Intra-rater** | **Measurement error** | **Construct validity** | **Responsiveness** |
| --- | --- | --- | --- | --- | --- | --- | --- | --- | --- | --- |
| 10-Meter Walking Test (10MWT) [1,2] | PerfO | <100 |  |  |  | ICC=0.98 (Comfortable) ICC=0.99 (Fast) |  |  |  | Comfortable pace: ES = 0.74, SRM = 0.92 Maximum pace: ES = 0.55, SRM = 0.83  (n=92) |
| 12-Meter Walking Test (12MWT) [2] | PerfO | 18 |  |  |  | ICC=0.71 | ICC=0.68 |  |  |  |
| 2-Meter Waling Test (2MWT) [1] | PerfO | 18 |  |  | ICC=0.85 | ICC=0.85 |  |  |  | SRM=1.34 |
| 6-Minute Walking Test (6MWT) [3,2] | PerfO | <100 |  |  |  | ICC=0.74 (n=18) | ICC=0.74-0.97 (n=18) | SEM=16.7 – 32.2 (n=74) | Timed Up and Go test: r=0.80 (admission); r=0.73 (discharge) (n=41) Peak Oxygen Consumption (VO2 Max): r=0.34 (n=30) |  |
| Actiwatch [4] | TechO | 52 |  |  |  |  |  |  | Actiwatches vs. motor score in patients with acute stroke without neglect: r =–0.88  Actiwatches vs. motor score in patients with acute stroke with neglect: r =–0.75 |  |
| Ambulatory Monitoring (AM Accelerometer) [4] | TechO | 43 |  |  |  |  |  |  | National Institute of Health Stroke Scale: r= –0.59 Activity of Impaired Arm: r=0.75 Fugel-Meyer assessment: r=0.54. Sensitivity and specificity of accelerometer 1.0 and .89, respectively. |  |
| Barthel Index (BI) [5] | PerfO | 22 | Experts | C α=0.90-0.93 | r=0.87 | r=0.88 | r=0.77-0.99 |  | Functional Independence Measure: r=0.93 London Handicap Scale: r= 0.37 36-item short form survey: r=0.22 | ES=0.71 (0-6 wks post-stroke) ES=0.25 (6-12 wks) AUC for change score=0.66 ES= 0.37 (from admission to discharge) SRM=0.63 and 0.86 for those known to change SRM=1.72 (admission to discharge) AUC/ROC=0.82 (using FIM as reference measures) |
| Beck Depression Inventory (BDI) [6] | PRO | 202 | BDI assesses 6/9 criteria delineated in the DSM-III. 2/3 remaining items are partially assessed. One criterion does not appear on the inventory | C α=0.76-0.86 | r=0.48-0.86 |  |  |  | Known groups: BDI discriminated between patients with varying levels of depression as indicated by rating of Depth of Depression. Differences between adjacent Depth of Depression categories (0 – 4—none to severe) significant at p-value=0.0004 and p-value=0.02 (moderate to severe) | Sensitivity/Specificity: via ROC analysis, determined that with the standardized/optimal cut-off of BDI=10, sensitivity was 80% and specificity 61.4%. Area under the curve was 0.89 for men and 0.69 for women (a higher rate of misdiagnosis for female stroke patients—this effect decreased when level of handicap measured on the Rankin scale was taken into consideration. |
| Berg Balance Scale (BBS) [5,1] | PerfO | >100 |  |  |  |  |  |  |  | SRM=1.04 (n=50) 14 to 30 days (ES=0.80) (n=110) 30-90 days (ES=0.69) (n=93) 90-180 days (ES=0.40) (n=80) 14 to 90 days (ES=1.07) (n=93) 14 to 180 days (ES=1.11) (n=80) 2 to 6 weeks: ES=0.66; SRM=0.81 6 to 12 weeks: ES=0.25; SRM=0.69 2 to 12 weeks: ES=0.97; SRM=1.08 (n=60) |
| Berg Balance Scale three point (BBS-3P) [1] | PerfO | >100 |  |  |  |  |  |  |  | 14 to 30 days : SRM=0.82 (n=202) 30 to 90 days: SRM=0.70 (n=167) 90 to 180 days: SRM= 1.11 (n=167) |
| Chedoke McMaster Stroke assessment scale (CMSA) [6] | ClinRO | 32 |  | r= 0.98 (total scale): (r= 0.94 (impairment) and r= 0.97 – 0.98 (disability)) | ICC=0.96-0.98 | ICC=0.85-0.96 | ICC=0.93-0.98 |  | Fugl-Meyer: r =0.76 – 0.95 Disability inventory sub scores correlated with corresponding Functional Independence Measure sub scores: r=0.85 – 0.90 | FIM and CMSA yielded significant variance ratios (p<0.001, one-tailed) from admission to discharge, however CMSA 1.92 times greater relative efficiency |
| EuroQol Quality of life scale (EQ5D) [7] | PRO | NR |  |  | ICC>0.75 |  |  |  | r=0.3-0.6 |  |
| Frenchay Activities Index (FAI) [5] | SRO | 35 |  | C α= 0.78 | r=0.80 | r=0.80 |  |  | Stroke Impact Profile: r = 70.73 to 70.56 |  |
| Fugl-Meyer Assessment (FMA) [8,9,7] | ClinRO | >100 |  | r=0.88  r =0.97, 0.90 and 0.88 for the upper extremity, lower extremity and balance sections (n=28) | ICC=0.96-0.99 (n=28) | r=0.98 (n=28) |  |  | Stroke Rehabilitation Assessment of Movement: r=0.73 (n=50) BI correlated with UE-FMA: r=0.75, with total motor-FMA: r =0.74, with balance: r =0.76, and with FMA total scores: r=0.67 | SRM=0.94-0.99 (n=78) |
| Fugl-Meyer test-Balance subscale (FM-B) [1] | ClinRO | >100 |  |  |  |  |  |  |  | 14 to 30 days, ES = 0.82 (n=110) 30 to 90 days, ES = 0.63 (n=93)  90 to 180 days, ES = 0.33 (n=80) 14 to 90 days, ES = 1.06 (n=93)  14 to 180 days, ES = 1.14 (n=80) |
| Function in Sitting Test (FIST) [10] | PerfO | 31 |  | C α=0.98 |  |  |  | SEM=2.97 | static and dynamic sitting balance: r=0.92 |  |
| Functional Ambulation Category (FAC) [11,1] | ClinRO | 101 |  |  | ICC≥0.75 | ICC≥0.75 |  |  | r≥0.60 | ES=0.5 to 0.8 Responsiveness ratios based on a 10% MCID exceeded the smallest detectable difference and ranged from 4.36 to 17.70 |
| Functional Independence measure (FIM) [5] | ClinRO | 52 |  | C α= 0.93-0.95 | ICC= 0.95 | r=0.95 |  |  | Known groups: Functional Independence Measure scores discriminated between groups based on right or left-sided involvement in stroke patients both at admission (p-value<0.005) and discharge (p-value<0.05). Most of this score difference occurred on the communication domain; on admission and discharge, FIM scores discriminated groups with and without neglect (p-value<0.001; p-value<0.02) and with or without aphasia (p-value<0.01; p-value<0.09). | ESs of 0.30, 0.34 and 0 were reported for the total-FIM, motor-FIM and cognitive-FIM respectively. ES=0.31 (0.46 in known changers) and AUC ROC curve=0.675 SRM=2.18 from admission to discharge from rehabilitation |
| London handicap score (LHS) [12] | PRO | 361 | Focus group and interviews |  | ICC=0.91 | r=0.90 |  |  | Barthel Index: r=0.56 Nottingham Health Profile: r=0.28-0.41 |  |
| Manual Function Test (MFT) [13] | ClinRO | 51 |  | C α= 0.95 | r=0.95 | r=0.95 |  |  | Brunnstrom Stage: r= 0.8 Stroke Impairment Assessment Set: r=0.8 Barthel Index: r=0.90 |  |
| Mini-Mental State Examination (MMSE) [6] | ClinRO | 116 |  | C α= 0.54 – 0.96 | ICC=0.38 – 0.99 | ICC=0.69 | ICC=0.69 |  | ADL scores and the MMSE of r= 0.40 – 0.75 Wechsler Adult Intelligence Scale verbal: r =0.78, and performance-IQ: r =0.66 scores |  |
| Modified Ashworth scale [6,9,7,14] | ClinRO | 36 |  |  | ICC>0.75 | ICC=0.5-0.75 | r =0.55-0.74 |  | Ashworth knee extensor scores related to resistance torque (r=0.53 – 0.59), stiffness (r=0.56 – 0.73) and joint angle (onset of stretch response 30, r =70.80). Ashworth knee flexor scores were related to stiffness 120 (r =0.56) and onset angle (120, r =0.58); Ashworth knee extensor scores related to functional outcomes assessed on Global Function Scale (r=70.48) and Gross Motor Function Measure (r =70.68) |  |
| Modified Emory Functional Ambulation Profile (M-EFAM) [1] | ClinRO | 40 |  |  |  |  |  |  |  | SRM=1.1 (discharge) |
| Modified Rankin Handicap Scale [6] | PRO | 1034 |  |  | Kw=0.95. | K=0.56 overall; K=0.82 and 0.51 for outpatient and inpatient groups, respectively |  |  | ADL measured on the BI (0.73) and IADL (0.65), mobility (0.60) and living arrangements (0.74) measured on subscales of the Sickness Impact Profile—The weakest associations reported were between modified-Rankin Scale and the Stroke Impact Profile subscales of cognitive alertness (0.34) and social interaction (0.37) | MRS detected change in significantly fewer patients post stroke than the FIM (P<0.005) ROC/AUC=0.29 using the FIM as reference measure |
| Motor Assessment Scale (MAS) [1] | ClinRO | 61 |  |  |  |  |  |  |  | mean time admission to discharge 56.4+/− 38.1 days: Item 1, ES = 1.03 Item 2, ES = 0.74 Item 3, ES = 0.61 Item 4, ES = 0.85 Item 5, ES = 1.02 |
| Motor status scale (MSS) [15,7,1] | ClinRO | 18 |  |  |  |  |  |  | Arm section of Motricity index at 6, 12, 18 wk after stroke: r=0.73-0.76 Fugl-Meyer assessment: r=0.96 |  |
| Postural Assessment Scale for Stroke Patients (PASS) [1] | PerfO | 202 |  |  |  |  |  |  |  | 14 to 30 days, SRM = 0.84 |
| Postural Assessment Scale for Stroke Patients Trunk Control (PASS-TC) [1] | PerfO | >100 |  |  |  |  |  |  |  | 14 to 30 days, ES = 0.89 (n=110) 14 to 30 days, SRM = 0.65 (n=246) |
| Postural Control And Balance for Stroke (PCBS) [1] | PerfO | 50 |  |  |  |  |  |  |  | 7-120 days, P<0.001 |
| Rivermead mobility index (RMI) [16,5] | SRO | 38 | Unidirectional |  | ICC=0.96 | r=0.94 |  |  | Guttmann analysis yielded a CR of 0.93 and a CS of 0.79  CR=0.95 and CS at admission and discharge of 0.74 and 0.79 respectively Rasch analysis revealed the ordering and item calibration to be consistent—trend of difficulty was the same in two groups at both admission and discharge; At admission and 5 weeks CR=0.95 and 0.93, CS=0.67—but, a larger percentage of patients were able to sit unsupported (item 3) than could move from lying to sitting or turn over in bed. RMI scores correlated with Barthel Index scores at 14, 30, 90 and 180 days post stroke (r=0.72, 0.88, 0.86 and 0.88); RMI scores at admission and at 5 weeks correlated with Motor- Functional Independence Measure (r=0.73, 0.91) Motricity Index—leg (r=0.49, 0.51) and Trunk Control Test (r=0.89, 0.83) RMI scores did not correlate significantly with cognitive Functional Independence Measure scores at either admission or at 5 weeks (r =0.10, r =0.20) | Significant change in RMI scores from admission to discharge from rehabilitation ward (P<0.001), ES=1.00 – relative efficiency as compared to the BI =1.42 Effect sizes were greatest in the interval between 14 and 30 days (SRM=1.14) and diminished the further one moved through time from stroke (30 – 90 days, SRM=0.86 and 90 – 180 days =0.24) Significant difference in RMI scores from admission to discharge (P<0.0001) and ES=0.89 |
| Rivermead Motor Assessment (RMA) [16,5] | PerfO | 51 |  |  | r=0.66 |  |  |  | CR=0.90 (leg and trunk) to 0.98 (gross function) Coefficients of scalability CS=0.79 (leg and trunk) to 0.96 (gross function)  A strong correlation between Barthel Index (ADL) scores and RMA (motor function) scores at initial (r =0.847), 1 month (r=0.777) and 1 year (r =0.627). The degree of asymmetry in weight distribution correlated significantly with RMA motor function (r= 70.45; p<0.001) RMA arm and leg scores correlated with Motricity Index arm and leg scores at 8, 12 and 18 weeks post stroke (r=0.73) and RMA gross function scale correlated with Trunk Control Test at same times post-stroke (r=0.70) |  |
| Short Form Berg Balance Scale (SFBBS) [1] | PerfO | 81 |  |  |  |  |  |  |  | 14-item BBS, ES = 0.85 7-item BBS, ES = 0.78 6-item BBS, ES = 0.78 5-item BBS, ES = 0.70 4-item BBS, ES = 0.69 |
| Short Form Postural Assessment Scale for Stroke Patients-6 items (6 SFPASS) [1] | PerfO | 262 |  |  |  |  |  |  |  | ES=0.43-0.44 |
| Smart Balance Master (SBM) [1] | TechO | 40 |  |  |  |  |  |  |  | Equilibrium score, ES = 0.63 Limits of stability time, ES = 0.27 Limits of stability path, ES = 0.33 Weight shifting, ES = 0.04-0.29 |
| Three Point Postural Assessment Scale for Stroke Patients (PASS-3P) [1] | PerfO | >100 |  |  |  |  |  |  |  | 14 to 30 days, SRM = 0.86 (n=202)  30 to 90 days, SRM = 0.67 (n=167)  90 to 180 days, SRM = 1.04 (n=167) |
| Uniaxial accelerometer [4] | TechO | <100 |  |  |  |  |  |  | 3.3h/d for the paretic arm (range, 0.8–8.1); 6h/d for the non-paretic arm (range, 3.2–9.4) Substantially (P<0.001) less than the 8.7h and 8.4h for healthy subjects’ dominant and non-dominant arms, respectively (n=34) Physical activity scale for individual with physical disabilities: r=0.3 (n=45) |  |

_ADL: activity of daily living, AUC: area under the curve, ClinRO: clinicians-reported outcome, Cα: Cronbacha alpha, CR:_ _Coefficients of reproducibility, CS: Coefficients of scalability, DSM-III: Diagnostic and Statistical Manual of Mental Disorders third edition, ES: effect size, IADL: instrumental activity of daily living, ICC: Interclass Correlation Coefficient K: Kappa, Kw: weighted Kappa, PerfO: performance-reported outcome, PRO: patients-reported outcome, r: Pearson correlation, ROC: receiver operating characteristic curve, SRO: self-reported outcome, SOI: source of information, SEM: standardized error of measurement, SRM: standardized root of mean, TechO: technology-reported outcome_

**B. Individuals with stroke at sub-acute setting**

| **Name of the measure** | **SOI** | **Sample size** | **Content validity** | **Internal consistency** | **Test-retest** | **Inter-rater** | **Intra-rater** | **Measurement error** | **construct validity** | **Responsiveness** |
| --- | --- | --- | --- | --- | --- | --- | --- | --- | --- | --- |
| 10-Meter Walking Test (10MWT) [11,17,18] | PerfO | <50 |  |  | ICC=0.74-0.98 (n=12) |  |  | SEM 0.08 m/s  LoA=-0.72 to 0.78 sec. (n=12) | 10-Meter Comfortable Walking Test: r=0.69 (n=43) |  |
| 12-Meter Walking Test (12MWT) [3] | PerfO | 20 |  |  |  |  | ICC= 0.68 |  |  |  |
| 3-Meter Walking Test (3MWT) [3] | PerfO | 14 |  |  |  |  | ICC=0.90 |  | Dynamometer: r = 0.41 |  |
| 6-Minute Walking Test (6MWT) [3,18] | PerfO | >100 |  |  | ICC=0.95-0.97 (n=24-37) | r=0.99 (n=45) |  | SEM 23.2 m (n=37) SEM: 18.6m. (n=83) SEM=12.4m. (n=45) | 10MCWT: r= 0.91 (n=24) Strength knee flexor dynamometer: r=0.71 (n=50) Strength knee extensor dynamometer: r= 0.39 (n=63) Strength dorsi-flexor dynamometer: r=0.50 (n=48) Strength ankle planter flexor dynamometer: r=0.43 (n=25) 5MCWT: r=0.89 Functional Independence Measure locomotion: r=0.69  FIM locomotion stairs: r= 0.69 (n=37) 10-Meter Walking Test: ICC=0.99 (n=45) |  |
| ActiGraph [4] | TechO | 20 |  |  | ICC=0.82-0.94 |  |  |  | Motor activity log (MAL): r=0.74 |  |
| Arm Motor Ability Test (AMAT) [19] | PerfO | 32 |  | C α=0.93-0.99 | r=0.93-0.99 | Test performance (κ = 0.68 to 0.77, r= 0.97-0.99) Performance time (κ and r = 0.99) |  |  | Fugl-Meyer Assessment: r = 0.92-0.94  upper limb section Motricity Index: r = 0.45-0.61 | Changes in scores for time, functional ability, and quality  of movement after 14 days of intensive therapy for the affected upper extremity |
| Berg Balance Scale (BBS) [11] | PerfO | 15 |  |  | ICC-0.75 | ICC=0.75 |  |  | r≥0.60 | ES≥0.8 |
| Dynamic Gait Index (DGI) [18] | PerfO | 45 |  |  | ICC=0.94 |  |  |  | 10-Meter Comfortable Walking Test: r=0.91 |  |
| Footswitches [18] | TechO | 25 |  |  | ICC=0.92-0.95 |  |  | SEMC gait speed 4.31 and 4.37 m/min SEMC cadence 7.45 and 6.16 steps/min (intra vs. inter) | Functional Ambulation Category: r=0.73 |  |
| Functional Ambulation Category (FAC) [1,2,18] | ClinRO | <100 |  |  | k=0.85-0.91 (n=20) |  |  |  | Gait speed: r=0.58 Walking distance: r=0.55 Gait energy cost: r=0.64 Functional Independence Measure: r=0.72 (n=20) 6-Minute Fast Walking Test: r= 0.91–0.95 10-Meter Fast Walking Test: r= 0.90–0.95 (n=55) | 2-week SRM=1.016 2 to 4-week SRM = 0.842 4-week to 6-month SRM = 0.699 (n=55) Sensitivity: 67-100% (n=55) |
| Motor Activity Log-28 items (MAL-28) [20,19] | SRO | 222 |  | C α = 0.80 | ICC = 0.79-0.82 |  |  |  | Patient and carer quality of movement and Accelerometer (r = 0.52-0.61) |  |
| Physical Ability Scale (PAS) [10] | PRO | 10 |  |  |  | k=0.62 | ICC=0.70 |  |  |  |
| Rivermead mobility index (RMI) [11] | SRO | 73 |  | C α=0.92 | ICC=0.75 | ICC=0.75 |  |  | r≥0.60 | ES≥0.8 |
| Stroke impact scale (SIS) [21] | PRO | 25 | The authors briefly described the development process of the SIS: literature reviews, and stroke survivors and caregivers input, indicating 2 of the 3 content validity criteria were met. | C α= 0.83-0.90 | ICC=0.70-0.92 |  |  |  | Known group methods, each SIS domain was compared with Rankin scores. The results of this analysis indicated that strength, hand function, activities of daily living (ADL), mobility, and participation were able to discriminate across Rankin scores (P<.001) |  |

_ClinRO: clinician-reported outcome, CI: confidence interval, ES: effect size, ICC: Interclass Correlation Coefficient K: Kappa, Cα: Cronbacha alpha, PerfO: performance-reported outcome, PRO: patients-reported outcome, r: Pearson correlation, LoA: limits of agreement, SRO: self-reported outcome, SOI: source of information, SEM: standardized error of measurement, SRM: standardized root of mean,TechO: technology-reported outcome_

**C. Individuals with stroke at chronic setting**

| **Name of the measure** | **SOI** | **Sample size** | **Content validity** | **Internal consistency** | **Test-retest** | **Inter-rater** | **Intra-rater** | **Measurement error** | **Construct validity** | **Responsiveness** |
| --- | --- | --- | --- | --- | --- | --- | --- | --- | --- | --- |
| 10-Meter Walking Test (10MWT) [11,17,2,18,22] | PerfO | >100 |  |  | ICC=0.85-0.98 (n=70) | ICC=0.87-1 (n=132) | ICC=0.89-0.99 (n=79) | SEM 0.07 m/s (n=50) SEM=0.08 m/s (n=20) | Barthel index: r=0.78 Instrumental activities of daily living: r=0.76 (n=40) Number of steps: r=0.97 (n=50) Infrared gating: r=1 (n=12) 300-Meter Walking Test: r=0.88 (n=28) | ES=1.17 m/s (n=19) |
| 12-Meter Walking Test (12MWT) [3,1,18] | PerfO | <50 |  |  |  | ICC=0.68 (n=18) | ICC=0.71 (n=18) |  | Chedoke-McMaster stroke assessment: r = 0.69  Berg Balance Scale: r = 0.80  8-m comfortable walk test: r = 0.91  6-Minute Walking Test distance: r=0.97 4-Meter Comfortable Walking Test: r=0.91 (n=25) | SRM=1.90 (n=18) |
| 2-Meter Walking Test (2MWT) [3,2,18] | PerfO | >100 |  |  |  | ICC=0.85 (n=18) | ICC=0.85-0.98  (n=140) | SEM=4.8 (n=61) SEM= 5.1 (n=12) SEM=4.8 (n=32) SEM=4.9 (n=17) |  |  |
| 300-Meter Walking Test (300MWT) [18] | PerfO | 28 |  |  | walking speeds: r=0.74-0.84 different walking steps: r=0.86 |  |  |  | 10-Meter Comfortable Walking Test: r=0.88 |  |
| 30-Meter Walking Test (30MCWT) [18] | PerfO | 18 |  |  |  |  |  |  | 10-Meter Comfortable Walking Test: r=0.91 |  |
| 4-Meter Comfortable Walking Test (4MCWT) [18] | PerfO | 25 |  |  |  |  |  |  | 6-Minute Walking Test distance: r=0.97 4MCWT: r=0.91 |  |
| 5-Meter Walking Test (5MWT) [3,2,18] | PerfO | <100 |  |  | ICC=0.80-0.97 (n=9) | ICC=0.99 (n=35) | ICC=0.97-0.99 (n=44) | SEM=6.9 (n=9) SEM=6.10 (n=55) | Functional Ambulation Scale: r = 0.55  5-m walk test: r = 0.80 (n=20) Dynamometer: r = 0.41 (n=10) Berg Balance Scale: r=0.64 Rivermead Mobility Index: r=0.64  (n=35) | ES=0.81 m/s (n=61) |
| 6-Minute Walking Test (6MWT) [11,3,1,17,2,18] | PerfO | >100 |  |  | ICC=0.78-0.99 (n=45) | ICC=0.74-0.97 (n=55) | ICC=0.98-0.99 (n=182) | SEM=12.4 (n=12) SEM=18.6 (n=50) SEM=18.1 (n=27) SEM=18.6 m (n=15) SEM=12.4 m (n=12) | VO2 Peak/age-predicted VO2 max: r=0.84 10-Meter Comfortable Walking Test: r=0.91 10-Meter Fast Walking Test: r=0.89 (n=27)" Max exercise test duration: r=0.60 (n=36) 5-Meter Comfortable Walking Test: r=0.79 5-Meter Fast Walking Test: r=0.82 (n=34)" 10MWT comfortable: r=0.84 10MWT fast: r=0.94 (n=50)" 3 –item short form survey-Physical: r=0.39 (n=61)" "EQ5D-Visual Analogue Scale: r=0.22 (n=64)" "Relative VO: r=0.66 (n=12)" Fugl-Meyer Lower extremity score: r=0.72 (n=34) Chedoke Mcmaster Stroke Assessment: r=0.75 (n=25) strength hip extensor dynamometer: r=0.40 (n=48) Stroke impact scale: r=0.52 (n=30) Berg Balance Scale: r=0.67 (n=21) Physical activity scale for individual with disabilities: r=0.31 (n=40) ICF measure activity: r=0.32 (n=77) Accelemeter activity: r=0.67 (n=40) Step Activity Monitor: r=0.58 (n=49) AvtivPAL: r=0.48 (n=17) The Intelligent Device for Energy Expenditure and Activity -Activity: r=0.60 (n=42) stair climbing-descent: r=0.80 stair climbing-Ascent: r=0.82 (n=50) five times sit to stand: r=0.60 (n=68) 12-Meter Walking Test : r=0.97 (n=25) 8-Meter Comfortable Walking speed: r=0.92 (n=25) Stroke impact scale- mobility: r=0.72 Stroke impact Scale- participation: r=0.56  (n=30) ICF measure participation: r=0.31 (n=77) Gait speed: r=0.89 Functional Independence Measure (walk): r= 0.69 Functional Independence Measure (motor): r=0.52 (n=37) 12-Meter Walking Test: r= 0.97 4-Meter Comfortable Walking Test: r= 0.92 (n=25) | SRM=1.52 (n=18) |
| ABILHAND [20,23,15,24,13,19,25,7] | PRO | 103 | Unidimentional | Cα = 0.80 | ICC=0.90-0.96 | r=0.90 |  |  | Grip strength: r=0.56 Box and Block test: r=0.598 Fugl-Meyer assessment-upper limb: r=0.73  grip strength (r = 0.56) and manual dexterity (r = 0.598) | ES=0.5-0.8 Obtained by distribution-based method:ES = 0.26 (in logits)  obtained by anchor-based methods:0.35 (in logits) compared to % of Recovery (10–15% or 50% recovery on the Stroke Impact Scale global recovery item) |
| Actical [26] | TechO | 40 |  |  | ICC=0.95 |  |  |  |  |  |
| Action Research Arm test (ARAT) [23,15,27,24,13,19,25,7,14] | ClinRO | 351 | Unidimentional | Cα = 0.97 | r=0.75-0.98 | ICC=0.98-0.99 |  |  | Motor assessment scale: r=0.96 Motoricity index: r=0.87 Motor assessment chart: r=0.94 Fugl-Meyer test: r=0.91-0.94 Wolf motor function test: r=0.96 Motor activity log: r=0.91-0.97 Motor assessment scale: r=0.96 Motricity index: r=0.87 Modified Motor assessment Chart: r=0.94 Fugl-Meyer Motor: r = 0.93   Ashworth Scale: r = -0.30 Box and Block Test: r= 0.95  Motricity Index: r = 0.81  Fugl-Meyer joint motion/pain: r = 0.42 Fugl-Meyer sensation: r= 0.30 Modified Barthel Index: r= 0.05 Fygl-Meyer Assessment: r=0.93 | ES=0.5-0.8 (n=30) |
| Activities of Daily Living scale [13] | ObserO | 81 |  |  |  | : ICC= 0.98 (total score) |  |  | Apraxia: r=0.43 Motor functioning: r= 0.37 Barthel Index: r=0.60 |  |
| Activity Cart Sort (ACS) [28,21] | PRO | <100 | The ACS was developed within the person environment framework, with reference to the current literature, and in consultation with carers and participants (n=29) | Cα =0.71-0.89 (n=60) | ICC=0.98 (n=60) |  |  |  | Reintegration to Normal Living Index: r=0.51 Stroke impact scale-recovery: r= 0.38 Stroke impact scale-communication: r=0.46 Stroke impact scale- participation: r=0.41 Stroke impact scale- physical domain: r=0.64 36-item short form survey (physical function): r=0.60 (n=29) Comprehensive quality of life scale: r=0.86 (n=60) |  |
| Actiwatch [4] | TechO | 11 |  |  |  |  |  |  | Validity testing of threshold filtered accelerometry worn at the wrist: Ratio determined by accelerometers for ADLs was 1:2.1:2.9 vs. the expected excellent ratio of 1:2:3 98% agreement between accelerometry and video 89% agreement between accelerometry and clinic or home setting |  |
| Actual Amount of Use Test (AAUT) [13] | ClinRO | 11 |  |  | r=0.76 |  |  |  | Motor activity log: r=0.45 |  |
| Ambulatory Monitoring (AM Accelerometer) [18] | TechO | 25 |  |  |  |  |  |  | dimensional gait analysis indoor: r= 0.87–0.96; outdoor: r=0.96–0.99 |  |
| Arm Motor Ability Test (AMAT) [24,13,7] | PerfO | 32 | Unidimentional |  | ICC=0.75 | ICC=0.75 |  |  |  |  |
| Assessment of Motor and Process Skills (AMPS) [13] | PerfO | 76 |  |  | Motor and process sub scores (r = 0.88 and r = 0.86, respectively). | Inter-rater: ICC=0.97-0.99 |  |  | the Scale of Independent Behavior: r=0.62-0.85 Functional Independence Measure: r=0.62 |  |
| Balance Assessment in Sitting and Standing Position (BASSP) [10] | ClinRO | 1193 |  |  |  |  | Rasch reliability coefficient of 0.93 |  | Postural Assessment Scale for Stroke: r=0.75 | ES-1.2 |
| Balance Evaluation System test (BESTest) [29] | PerfO | 115 | Unidimentional |  |  |  |  |  | NR Rasch results |  |
| Barthel Index (BI) [6,30,7,22] | PerfO | 167 |  |  | ICC=0.98 |  | k=0.75 |  | Office Population Censuses Surveys: r=0.84 36-items short form survey-Physical r=0.3 | ES=0.24–0.39 SRM=0.56 |
| Beck Depression Inventory (BDI) [9] | PRO | 202 |  |  | ICC=0.92 |  |  |  | r>0.70 | ES<0.5 |
| Berg Balance Scale (BBS) [6,22] | PerfO | <100 |  | Cα =0.83 and 0.97  Item to total correlations ranged from 0.67 – 0.95 (n=70) | ICC=0.96-0.99 (n=56) | ICC=0.98 (n=56) |  |  | Barthel Index: r =0.80 Fugl-Meyer scale scores: r=0.62 – 0.94 Functional Independence Measure: r =0.57 to 0.70 Timed up and go test: r=70.7  Fraters: r=0.88 (n=70) | ES= 0.66 for initial 6-week, post-stroke evaluation period, ES=0.25 for 6 – 12 weeks and overall ES of 0.97 Effect sizes were greatest in the interval between 14 and 30 days (0.80) and diminished the further one moved through time from the stroke event (90 – 100 days—effect size=0.40) (n=70) |
| Biaxial accelerometer [4] | TechO | 6 |  |  | ICC=0.85 |  |  |  | The accelerometry system showed concurrent validity because no differences in spatiotemporal measures of gait were found between the accelerometry system and the Stride Analyzer System |  |
| Box and Block test [15,7] | ClinRO | <100 |  |  | ICC=0.93-0.98 (n=52) | r=0.96 (n=52) | r=0.99 (n=52) |  | Grip strength: r=0.87 Test d’Evaluation de la performance des Membres Supérieurs des Personnes Agées: r=-0.73 to 0.78 Fugl-Meyer Assessment (motor) r= 0.92 Fugl-Meyer Assessment (joint movement/pain) r= 0.43 Action Research Arm Test r= 0.95 Motricity Index r= 0.798 Barthel Index r =0.044 (n=15) |  |
| Brunel Balance Assessment [29] | ClinRO | 92 | Each item of the Brunel Balance Assessment has been evaluated with people post stroke for validity, hierarchical positioning in the scale and redundancy of items |  | k=1 |  |  |  | Berg Balance Scale: r=0.97 Rivermead Mobility Index: r=0.95 |  |
| Caltrac accelerometer [26,4] | TechO | 27 |  |  | ICC=0.44 |  |  |  |  |  |
| Canadian Occupational Performance Measure (COPM) [13] | PRO | 26 |  |  | r=0.89 |  |  |  | Barthel Index: r=0.335 Frenchay Activities Index: r=0.115 Stroke Impact Profile: r=0.102 EuroQoL : r=0.143 Rankin Handicap scale : r=0.21 | AUC=0.79-0.85 |
| Centre for Epidemiological Studies Depression [6] | PRO | 27 |  | Cα =0.83–0.91 |  |  |  |  | r>0.70 |  |
| Chedoke Arm and Hand Inventory (CAHAI) [23,13,19,7] | PerfO | 109 | Items generated by stroke survivors and judged by a team of experts in stroke rehabilitation | Cα = 0.98 | ICC=0.96-0.98 | ICC=0.75-0.98 |  |  | Chedoke arm and hand inventory r=0.93 Action Research Arm Test: ICC = 0.95 | ROC curve areas = 0.72 ES=0.8 |
| Chedoke McMaster Stroke Assessment (CMSA) [27,5,7] | ClinRO | 127 |  |  | ICC=0.96-0.98 | ICC=0.88-0.93 |  |  | Fugl-Meter sensorimotor assessment: r=0.95 | ES>0.8 |
| Climbing stairs questionnaire (CSQ) [16] | PRO | 15 | Focus groups | Cα = 0.70-0.96 | ICC=0.77 | k>0.7 |  |  | r>0.70 |  |
| Coded activity diary [28] | PRO | 16 |  |  |  |  |  |  | Metabolic equivalent minutes (MET.min) between patient’s diaries and observer’s diaries: rs= 0.75 Metabolic equivalent minutes (MET.min) between patient’s diaries and Sensewear Pro2 armband (SWP2A): rs= 0.15 Energy expenditure (kcal/12 h) between patient’s diaries and observer’sdiaries: rs= 0.92 Energy expenditure (kcal/12 h) between patient’s diaries and Sensewear Pro2armband (SWP2A): rs= 0.29 |  |
| Community balance and mobility scale (CB&M) [29] | PerfO | 44 |  |  |  |  |  |  | Berg Balance Scale: r=0.83 Timed up and go test: r=0.75 | SRM=0.83 |
| Computer Science and Applications Inc. Model 7164 activity monitors x 4 [26] | TechO | 9 |  |  |  |  |  |  | Video: ICC=0.99 |  |
| Dimensional gait analysis (3-DGA) [4] | TechO | 25 |  |  |  |  |  |  | Step Activity Monitor: r=0.89-0.95 |  |
| Disabilities of the Arm, Shoulder and Hand (DASH) [24] | SRO | 300 |  | Cα = 0.98 | r=0.96 |  |  |  | Fit statistics =7 items misfit ‘heavy chores,’ ‘carry heavy object,’ ‘open door,’ ‘recreational activities,’ ‘stiffness,’ ‘less capable,’ ‘tingling’ |  |
| Duruoz Hand Index (DHI) [13] | SRO | 56 |  | Cα = 0.97 | ICC=0.99 |  |  |  | Functional Independence Measure-self-care item: r=0.73 |  |
| Dynamic Gait Index (DGI) [29,18] | PerfO | 25 |  |  | ICC=0.96 | ICC=0.96 |  |  |  |  |
| European Quality of life scale (EQ5D) [6,31,32,12] | PRO | 15 |  |  | ICC=0.77 |  |  |  | Barthel Index: r=0.709 Frenchay Activities Index:r=0.65 |  |
| Finger Tapping [4] | TechO | 60 |  |  |  |  |  |  | European stroke scale: r=0.526 IT-MAX (minimal index to thumb movement on 15 sec): r=0.68 |  |
| Fitbit Ulta [26] | TechO | 30 |  |  |  |  |  |  | Pedometer: ICC=0.70 | Mean inference with video: 16 steps in 2-Meter Walking Test |
| Fitts Reaching test [15] | PerfO | 18 |  |  | ICC=0.74-0.95 |  |  |  | Action Research Arm test: r=0.27-0.54 Hand motor assessment scale: r=0.64 |  |
| Five times Sit to Stand test (5xSTST) [33] | PerfO | <100 | Experts (n=58) |  | ICC=0.87-1 (n=31) | ICC=0.99 (n=12) | ICC= 0.98 (n=12) | SEM=1.8 (n=19) | Isometric strength knee: r=0.75-0.83 (n=12)  PLR=2.4 and NLR=0.46 (n=27) | Sensitivity: 0.83%; Specificity: 0.75%  (n=12) |
| Footswitches [4] | TechO | 25 |  |  |  |  |  |  | Step Activity Monitor: r=0.99-0.96 LOA: 9 (mean error, 4.5% to 2.5%) and 57 (mean error, 42% to 16%) steps for nonparetic and paretic limbs, respectively |  |
| Four Square Step [29] | ClinRO | 37 |  |  |  |  |  |  | Step test: r=0.73 - 0.86 | Ambulatory in and outpatient (9–1094 days post-stroke). Change after 4 weeks of rehabilitation, ES=0.33 |
| Frenchay Activities Index (FAI) [28,31,21,22] | PRO | >100 | Factor analysis (n=581) | Cα =0.78-0.87 (n=188) | ICC=0.89-0.91 (n=74) | ICC=0.90-0.93 (n=114) |  |  | Nottingham Extended Activities of Daily Living: r=0.90 (n=238) Stroke impact scale: r=0.40 Motor Activity Log/amount of use: r= 0.30 Motor Activity Log/quality of movement: r=0.30 Reintegration to Normal Living Index: r=0.61 Activities-specific Balance Confidence Scale: r=0.55 Timed up and go test: r=0.68 (n=383) Barthel Index: r=0.80 Stroke impact profile: r=0.14 (n=188) NIHSS: r=0.23 (n=36) | SRM=0.5 (n=70) ES=0.59 (n=163) |
| Frenchay Arm Test (FAT) [6,13,7] | ClinRO | 93 |  |  | ICC>0.75 | r = 0.75 to 0.99 |  |  |  |  |
| Fugel-Meyer Assessment (FMA) [6,27,14] | ClinRO | 377 |  |  | ICC=0.80 | r=0.99 ICC=0.97 |  |  | Chedoke McMaster Stroke Assessment: r=0.95 Motor assessment scale: r=0.91 |  |
| Fugl-Meyer Assessment-Upper extremity (FMA-UE) [24] | ClinRO | 512 | Unidimentional |  | r=0.96 |  |  |  | Fit statistics=2 items misfit/removed additional reflex item (elbow reflex) ‘biceps reflex,’ ‘triceps reflex’ | RR=0.41 compared to RR=2.03 for Action Research Arm scores following a programme of intensive forced use to improve arm function in chronic stroke patients (51 year post stroke ES=0.24 for total FMA scores from admission to 5 weeks post stroke — ES for UE, LE and balance scales were 0.20, 0.19 and 0.33 respectively |
| Functional Ambulation Category (FAC) [2,18,22] | ClinRO | <100 |  |  | ICC=0.36 (n=25) | ICC=0.95 (n=55) | ICC=0.74-0.96 (n=55) |  | Velocity: r=0.74-0.84 Number of steps: r=0.86 (n=31) Rivermead Mobility Index: r=0.69-0.89 6-Minute Walking Test: r=0.90-0.95 Velocity: r=0.90-0.95 Step length: r=0.88-0.95 (n=55) | Specificity: 16-100% (n=25) |
| Functional Independence Measure (FIM) [6,31,30,7,14] | ClinRO | 18 | Experts |  | ICC= 0.87-0.91 | ICC=0.75-0.99 | ICC=0.94-0.98 |  | Ambulatory Index: r= -0.73 Barthel Index: r=0.88 36-item short form survey-Phyiscal: r=0.88 | ES=0.27-0.46 SRM=0.48 |
| Functional Gait Assessment (FGA) [18] | PerfO | 28 | Experts |  |  |  |  |  | Barthel Index: r=0.71  Berg Balance Scale: r=0.93  Rivermead Mobility Index: r=0.85  Functional Activity Category: r=0.83  Gait speed: r=0.82 |  |
| Functional Test for the Hemiplegic Upper Extremity (FTHUE) [13] | ClinRO | 82 |  |  |  | r=0.976 |  |  |  |  |
| Geriatric Depression scale-long form (GDS) [6] | PRO | NR |  |  | ICC=0.85 |  |  |  |  |  |
| Grasp-Release test [14] | PerfO | 60 |  | Cα =0.75 |  | k=0.75 |  |  |  | ES=0.5-0.9 |
| Grip strength [7] | ClinRO | 27 |  |  | ICC=0.75 | ICC=0.75 |  |  |  |  |
| Hand Function Survey (HFS) [13] | SRO | 45 |  |  | r=0.79-0.94 |  |  |  | Motor Activity Log: r=0.93-0.98 |  |
| Human activity profile (HAP) [16,28] | PRO | 24 | Focus groups | Cα = 0.70-0.94 | ICC=0.78 | k>0.8 |  |  | Max activity score: r=0.95 Adjusted activity score: r=0.99 | ES=0.3-0.6 |
| International Classification of Functioning, Health and Disability-Activity Measure (ICF-AM) [24] | SRO | 317 |  |  |  | ICC=0.86-0.90 |  |  | Fit statistics =2 items misfit ‘pushing a shopping cart,’ ‘typing on a computer keyboard’ |  |
| Jebsen Hand Function Test [13,14] | PerfO | 33 |  |  | r=0.95 |  |  |  | Nine Hole Peg Test: r=0.86-0.88 | ES= 0.5-1.02 from 1-3 months; ES=0.56-0.86 from 1 to 6 months |
| Kinematics [7] | TechO | 8 |  |  | ICC=0.75 |  |  |  | r=0.3-0.6 | ES>0.8 |
| Assessment of Life Habits (LIFE-H) [31,21] | PerfO | 84 | The LIFE-H is model based (the Disability Creation Process Model and was developed in consultation with experts and consumers, as well as review of the literature |  | ICC=0.95 |  |  |  | Functional autonomy measurement system: r= 0.7 (total score); activity of daily living: r=0.76, social roles: r=0.43 (Discriminate validity tested the hypothesis that LIFE-H scores could differ from one living environment to another. LIFE-H scores were able to distinguish between those living in their own home compared with those living in a nursing home or in long-term care in daily activities sub-scores, but not using the social roles sub-scores. Construct validity was also tested using known group methods in 46 older stroke survivors and 46 healthy older adults.37 Scores for healthy older adults were significantly higher than stroke survivors (P values from .002 to <.001) except for the domain of “interpersonal relationships”. Hence, not all domains covered in the test met the criterion for construct validity |  |
| London Handicap scale (LHS) [12] | PRO | 37 | The development of the LHS was based on the International Classification of Impairments, Disabilities and Handicaps (ICIDH) conceptual framework, in consultation with health practitioners and consumers, and in review of the literature | Cα =0.80 | r=0.91 |  |  |  | Barthel index: r=0.56 Nottingham Extended Activities of Daily Living Scale:r=0.69 |  |
| Medical Outcomes Study 36-Item Short Form Health Survey (SF-36) [6,31,30,32,14] | PRO | 60 |  | Cα =0.94 | ICC=0.61-0.81 | r=0.75 |  |  | Functional Independence Measure: r=0.81 Barthel Index: r=0.3 | ESs (admission to outpatient rehabilitation to discharge)=0.48 |
| Mini Mental State Examination (MMSE) [9] | ClinRO | 75 |  |  | ICC>0.75 |  |  |  | r>0.60 |  |
| Modified Emory Functional Ambulation Profile (M-EFAM) [29,2] | ClinRO | <100 |  |  | ICC=0.99 (n=26) | ICC=0.97-0.99 (n=40) | ICC=0.99 (n=28) |  | 10-Meter Walking Test: r=0.78 Berg Balance Scale: r=0.59- 0.73 (n=28) Functional Independence Measure (motor): r=0.14-0.78 (n=26) 10-Meter Walking Test: r=0.88-0.93 Rivermead Mobility Index: r=0.67-0.81 (n=40) | Demonstrated decrease time scores over outpatient  physiotherapy treatment (mean 16.034.68, 45 minute mobility treatment sessions) (n=26) |
| Modified Functional Reach test (MFRT) [1,10] | PerfO | 35 |  |  | ICC=0.90-0.95 |  | ICC=0.90-0.97 |  |  | ES=0.80 6-weeks reach direction:  Paretic side: ES = 0.80 Forward, ES = 0.60 Non-paretic side, ES = 0.57 |
| Motor activity log (MAL-14) [20,15,13,19,14] | SRO | >100 | Established by examining item-total correlations, reliability and frequency with which item was deemed not appropriate for test | Cα = 0.81 (n=56) | r = 0.44-0.91 (n=27) |  |  |  | (Patient and carer quality of movement: ICC = 0.52-0.7  Action Research Arm Test: r = 0.63 (n=56) Global Change Rating: r = 0.16 Accelerometer: r=0.74 (n=20) Accelerometer: r=0.7-0.91 (n=41) | Responsiveness ratio 1.9-2.0 |
| Motor Assessment Scale (MAS) [6,27,8,7] | ClinRO | 37 |  |  | ICC=0.98 | r=0.95-1 |  |  | Fugl-Meyer Assessment; r=0.88 Action Research Arm Test: r=0.87 | Item 5 (walking) showed a large effect size; the other items have small effect sizes (d 0.36–0.5) and the majority of subjects showed no change over time |
| Motor Evaluation Scale for Upper Extremity in Stroke Patients (MESUPES) [24,13] | ClinRO | 396 | Two dimensions: arm and hand |  | ICC=0.95-0.97 |  |  |  | Fit statistics =5 items misfit (not able to identify the misfit items) |  |
| Motor Free Visual Perception Test [6,9] | ClinRO | 30 |  |  | ICC-0.98 |  |  |  | r>0.60 |  |
| Motricity index (MI) [6,27,11,8,22] | ClinRO | 55 |  | Cα = 0.77 | ICC=0.56-0.91 | r=0.88 |  |  | Rivermead Mobility Assessment (6 weeks: r=0.76; 12 weeks: r=0.73, 18 weeks: r=0.74) Nine-Hole Peg test: r=0.82 Action Research arm test: r=0.87 Fugl-Meyer-leg: r=0.69 Fugl-Mayer-arm: r=0.71 Rivermead Mobility Assessment: r=0.75-0.81 |  |
| Multimedia activity recall for children and adults (MARCA) [28] | SRO | 40 |  |  | ICC=0.83-0.95 |  |  |  | activPAL3 activity monitor (total sitting time): ICC=0.67 |  |
| National Institute of Health Stroke Scale (NIHSS) [6] | ClinRO | 65 |  |  |  |  |  |  | r=0.75 | Low Sensitivity |
| Neurobehavioral Cognition Status Exam (NCSE) [6] | ClinRO | 65 |  |  | r= 0.69 | r=0.57 |  |  |  | Patients who have scores that are lower than those in the average range on any test are impaired in that specific skill.99 For geriatric population (77.6 years 5.2 years) the normal ranges for the different tests are: Orientation 11.7  0.7; Attention test 7.7  0.9; Comprehension  5.9 0.4; Repetition  12.4 0.8; Naming  8.2 1.1; Constructions 4.4  1.5; Memory 10.1  2.2; Calculations 3.9  0.3; Similarities 5.6  1.3; Judgment 5.0  0.8 |
| Nike+Fuelband [26] | TechO | 30 |  |  |  |  |  |  | Pedometer: ICC=0.19 | Mean difference with video: 73.05 steps in 2-Meter Walking Test |
| Nine-Hole Peg test (NHPT) [15,27,7] | ClinRO | 62 |  |  | ICC=0.85-0.89 | r=0.68-0.99 |  |  | Grip strength: r=0.71 Jebson hand function: r=0.83-0.85 Upper Extremity Performance Test for Elderly (Test d’Evaluation des Membres supérieurs de Personnes Agées: r=0.79-0.90 |  |
| Nottingham Extended ADL index (N-ADL) [16] | PRO | 78 | Focus groups | Cα = 0.70-0.95 | ICC=0.77 | k>0.7 |  |  | r>0.70 | ES=0.5-0.8 |
| Nottingham leisure activity (NLA) [28,32] | PRO | 21 |  | Cα =0.87 | r=0.77 | ICC=0.3-0.57 |  |  | Nottingham Health Profile- emotional reaction scale at six months post stroke: r =0.71 Nottingham Health Profile scores (all sections) distinguished between stroke survivors and age-matched controls at one and 6 months post-stroke (p<0.01) and between those able to walk vs. unable to walk at 1 and 6 months post stroke (p<0.05) |  |
| OMRON HJ-113-E Piezoelectric Pedometers [26] | TechO | 50 |  |  |  |  |  |  |  | Underestimates step counts. Mean difference with video –32.4 steps in 6-Minute Walking Test |
| Ottawa Sitting Scale (OSS) [10] | ClinRO | 71 |  |  |  | ICC=0.96-0.98 | ICC=0.99 |  |  |  |
| Outpatient Physical Therapy Improvement in Movement Assessment Log (OPTIMAL) [24] | PRO | 3138 |  |  |  |  |  |  | Fit statistics =no item misfit |  |
| PAL2 (Gorman ProMed Pty. Ltd) [26] | TechO | 20 |  |  |  |  |  |  | Visual observation: ICC= 0.68-0.74 (measured over 8 hours) |  |
| Pedometers [26,4,18] | TechO | 20 |  |  | r=0.64 |  |  |  | Hand held counter/visual observation: ICC=0.58 Accordance for both speeds (self-selected and fastest comfortable speed) 98.7 1.2% vs. 87.2 11.3%, P<0.001; and 97.8 2.3% vs. 84.8 14.8%, P<0.01, respectively, in favor of Step Activity Monitor | Mean difference with visual observation: 31 steps in 2-Meter Walking Test |
| Postural Assessment Scale for Stroke Patients (PASS) [1,10] | PerfO | 167 |  |  |  |  |  |  |  | 30 to 90 days, SRM = 0.65 90 to 180 days, SRM = 1.02 ES=0.90 SRM=1.32 |
| Postural Assessment Scale for Stroke Patients Trunk Control (PASS-TC) [1] | PerfO | 93 |  |  |  |  |  |  |  | 30 to 90 days, ES = 0.64 (n=93)  90 to 180 days, ES = 0.31 (n=80)  14 to 90 days, ES = 1.07 (n=93)  14 to 180 days, ES = 1.12 (n=80)  30 to 90 days, SRM = 0.42 (n=203) 90 to 180 days, SRM = 0.02 (n=189) |
| Quadriplegia Index of Function [14] | ClinRO | 60 |  |  | ICC=0.80 vg |  |  |  |  | ES=0.5-0.7 |
| Reintegration to normal living index (RNLI) [12] | PRO | 57 |  |  | ICC=0.89 |  |  |  | Activity Cart Sort: r=0.56 |  |
| Rivermead mobility Assessment (RMI) [8,30,1,2] | SRO | >100 |  | Cα =0.88 - 0.95 (n=107) | r=0.79-0.98 (n=51) |  |  |  | Functional Independence Measure: r=0.38-0.86 (n=51) 10-Meter Walking Test: r=0.80 to 0.82  Ambulatory Index: r = -0.96   6-Minute Walking Test: r=0.63 Functional Independence Measure: r=0.73-0.91 Motricity Index- leg: r=0.49-0.51 Trunk Control Test: r=0.83-0.89 (n=73) Barthel Index: r=0.6 Berg Balance Scale: r=0.8 (n=38) | ES=0.89 (n=73) |
| Rivermead motor assessment (RMA) [6,15,27,8,24,7] | PerfO | 158 |  |  | ICC=0.61 |  |  |  | Motricity Index 6 weeks: r=0.76 12 weeks: r=0.73 18 weeks: r=0.74 |  |
| Sensewear Pro 3 Armband [26] | TechO | 12 |  |  |  |  |  |  | Step Activity Monitor: ICC=0.35 | Mean difference: 3.8% (measured for 10 days) |
| Short form of the Wolf Motor Function Test (S-WMFT) [24] | PerfO | 172 | Unidimentional | Cα =0.91 | r=0.89-0.91 |  |  |  | Fit statistics =no item misfit |  |
| Sickness Impact profile (SIP) [6,31,32] | PRO | 574 |  | Cα =0.85 | ICC=0.83 |  |  |  | Barthel Index: r =70.587 Rankin Handicap Scale: r=0.468 Frenchay Activities Index: r =70.426 European Quality of life: r=70.48 |  |
| Sitting Rising Test (SRT) [10] | ClinRO | 60 |  |  | ICC=0.679-0.863 | ICC=0.872-0.967 |  |  | Ankle dorsiflection: r=0.376 Ankle planterflexion: r=0.46 |  |
| SmartShoe [26] | TechO | 12 |  |  |  |  |  |  | Video: ICC=0.99 | Fast and self-selected speeds. Accuracy for correctly identifying postures: 97.2% |
| Sodring motor evaluation for stroke patients [8] | ClinRO | >100 |  | Cα =0.94-0.99 (n=123) |  | ICC=0.95 (n=30) |  |  | Brigitta Landmark motor assessment: r= 0.83-0.94 | SRM=1.10-1.26 |
| Sollerman hand function test [15] | PerfO | 24 |  |  | ICC=0.96 | r=0.92 |  |  |  |  |
| Step test [29] | ClinRO | 41 |  |  | ICC=0.93 (affected) to ICC=0.94 (unaffected) |  |  |  | Functional reach: r=0.68 (affected leg stepping up); r= 0.73 (unaffected leg)   Gait speed: r= 0.83  Stride length: r=0.82 | Ambulatory in and outpatient (9–1094 days post stroke). Change after 4 weeks of PT: ES=0.47–0.60 Inpatient rehabilitation initiated within 4 weeks post stroke. Change after 8 weeks: SEM= 0.92–0.95 (note: 41% of participants unable to perform test on admission skewing results) |
| StepWatch Activity Monitor or Step Activity Monitor (SAM) [26,4,2] | TechO | <100 |  |  | ICC=0.95 (n=40) | ICC=0.96 (n=17) |  |  | Video: ICC=0.97 (n=30) Footswitches: ICC=0.96 (non-hemi leg); ICC=0.896 (hemi leg) (n=25) Functional Independence Measure: r=0.52 at baseline; r=0.62 at 3 months) (n=21) Berg Balance Scale: r=0.58 (n=50) Dynamic gait index: r=0.89-0.95 Footswitches: r=0.96-0.99. (n=25) Gait speed: r=0.45 Rivearmead Mobility Index: r=0.3  Femur: r=0.52–0.62 (n=19) | Mean difference with video: 4.73 steps in 2-Meter Walking Test (n=30) Increased error with hemi leg outdoors. (n=25) Extreme slow stride counts (mean steps/d: 2837-1503) compared with norms for older and/or sedentary adults (5000–7000) (n=50) |
| Stride analyzer system (SAS) [4] | TechO | 6 |  |  | ICC=0.85 |  |  |  | Actual Amount of Use Test (ACS) vs. SAS, no differences between spatiotemporal parameters |  |
| Stroke Arm Ladder (SAL) [24] | ClinRO | 942 | Unidimentional |  | r=0.90-0.91 |  |  |  | Fit statistics =no item misfit in the final item bank |  |
| Stroke Impact Scale (SIS) [16,6,31,32,25,7,12] | PRO | 696 | Focus groups | Cα = 0.83-0.90 | ICC=0.7-0.82 | k>0.7 | ICC=0.50-0.83 |  | Construct validity (convergent/discriminate): SIS physical domains (strength, ADL/IADL, mobility and hand function) correlated most strongly with WHOQOL physical domain (r =0.40, 0.61, 0.63 and 0.40 respectively) and least with WHOQOL social relationships (r=0.13, 0.13, 0.18, 0.18), memory and emotion scores were more associated with WHOQOL psychological domain (r=0.49 and 0.70) and Zung Depression Scores (r=70.38 and 70.62) than with the physical domain of WHOQOL (r=0.32 and 0.41). Memory was least correlated with WHOQOL environment scores (r=0.15). Participation was correlated with all domains of WHOQOL and with Zung (r=0.45 – 0.69 and 70.56). Neither Zung nor WHOQOL assess communication—correlations with SIS communication ranged from 0.11 – 0.28 and 70.28 for the Zung Depression Scale score | SIS showed significant change in the expected direction in patients’ recovery between assessments done at 1 and 3 months and 1 and 6 months, but sensitivity was affected by severity and time post stroke. For hand function, mobility, ADL/IADL, combined physical and participation, change is significant from 1 – 3 and 1 – 6 months, but not 3 – 6 months among patients recovering from minor stroke. For moderate strokes, significant change is seen for the same two time periods in all domains and from 3 – 6 months for mobility, ADL/IADL, combined physical and participation domains |
| Stroke Rehabilitation assessment of movement (STREAM) [23,15,8,24] | ClinRO | 351 | Three dimensions: upper limb movements, lower-limb movements, and mobility | Cα = 0.98 | ICC=0.75 |  | r=0.99 |  | Fugl-Meyer assessment: r=0.91 | SRM=0.51-1.2 |
| Stroke Specific Quality of Life Scale (SSQOL) [6,31,32] | PRO | 71 |  | Cα = 0.75 | ICC=0.41 | r=0.59 |  |  | Discriminative of patients rating health-related quality of life as same vs. worse than pre-stroke (p<0.001) though only one domain (family roles) was significantly different between groups when scores were examined on the domain level | Socio-economic Status scores for the interval between 1 and 3 months post stroke were found to be between 0.20 (personality) and 0.83 (social roles). One half of SSQOL domains demonstrated less than moderate effect sizes; amount of help response set appears to lack responsiveness |
| The Intelligent Device for Energy Expenditure and Activity (IDEEA) [26,4] | TechO | 42 |  |  | ICC=0.69-0.80 |  |  |  |  |  |
| Timed Up and Go test (TUG) [6,29,5,17,2] | PerfO | 50 |  |  | ICC=0.96 | ICC=0.94 |  |  | Berg balance scale: r= -0.70 Community balance and mobility: r=-0.75 Gait parameters: r=0.62-0.90 Ankle strength: r=0.86 6-Minute Walking Test: r=0.96 | Ambulatory inpatient (post-stroke with change reported post 304 days of treatment): SRM=0.73 |
| Timed walk [30] | PerfO | 22 | Has established face validity and is relatively independent of distance |  |  |  | ICC=0.96-0.99 |  | Max walk: r= -0.79 2 min walk: r= -0.61 Rivearmead Mobility Index: r= -0.80 |  |
| Triaxial accelerometer/ RT3 [26,4] | TechO | 52 |  |  | ICC=0.70-0.93 |  |  |  | Triaxial accelerometer to measure trunk asymmetry during gait and reliability of accelerometer to determine asymmetry indexes: Significant differences between control and experimental group for anteroposterior, vertical, and mediolateral asymmetries measured by accelerometers; the vertical trunk parameter has the best discriminating ability with a sensitivity of 82.5, specificity of 85%, and an area under the curve of 0.90 (P<0.001) |  |
| Trunk Control test [10,22,34] | ClinRO | 20 |  | Cα = 0.83-0.86 |  | ICC=0.96 | r=0.87 |  | Motricity Index-leg: r=0.71 Functional Activity Category: r=0.69 Rivearmead Mobility Assessment: r=0.70 |  |
| Trunk Impairment Scale [10,34] | ClinRO | 73 | Literature review, observing stroke patients, clinical experience and discussion with specialists in stroke rehabilitation | Cα = 0.65-0.89 |  | ICC=0.87-0.96 | ICC=0.85-0.99 | Inter- and test–retest examiner measurement error (inter: 1.84 to 1.84,  test–retest: 2.90 to 3.68) | Barthel Index: r=0.86 |  |
| Trunk Impairment Scale (TIS)- Verheyden version [10] | ClinRO | 73 |  | Cα = 0.65-0.89 | ICC=0.46-1 | k=)70-1 |  |  | Barthel Index: r=0.86 Trunk Control Test: r=0.83 |  |
| Trunk Impairment Scale - Fujiwara version [10] | ClinRO | NR |  | Rasch analysis: all but 3 items showed mean square fit statistic within 1.3 |  | K=0.66-1 |  |  | Trunk Control Test: r=0.91 Functional Independence Measure (at discharge): r=0.09 | SRM=0.94 |
| Upper Body Dressing Scale (UBDS) [13] | ClinRO | 51 |  | Cα = 0.88 |  | ICC=0.87 (total score) |  |  | FIM-dressing item: r=0.72 | AUC=0.86 |
| Upper Extremity Functional Index (UEFI ) [24] | ClinRO | 239 |  |  |  | ICC=0.95 |  |  | Fit statistics =5 items misfit ‘sleeping’, ‘usual hobbies,’ ‘dressing’, ‘throwing a ball,’ ‘typing an lacing shoes’ |  |
| Upper Extremity Performance Test for Elderly (Test d’Evaluation des Membres supérieurs de Personnes Agées (TEMPA) [13] | ClinRO | 29 |  |  | ICC=0.70 - 1 |  |  |  | Acton Research Arm test : r=0.7 Box and block test: r= 0.5 functional independence to basic personal care: r=0.74 |  |
| Upper Limb-Motor Assessment Scale (UL-MAS) [24,19] | ClinRO | 80 | Unidimentional | Cα = 0.83 | r=0.98 | Kappa coefficient item 6, 7 and 8 = 0.93, 1.0, 1.0 Items 6, 7 and 8 (rs = 1.0) Items 6, 7 and 8 (rs = 1.0, 1.0, 0.98) | Kendall’s rank order correlation coefficient for items 6, 7 and 8 = 0.74, 1.0, 1.0 |  | Fugl-Meyer Assessment-Upper limb: r = 0.89-0.92 Fit statistics =1 item misfit ‘radial deviation test’ | ES = 0.45, Wilcoxon Z = 4.54, p<0.001, rho = 0.7  Ability of UL-MAS subscale items to discriminate between abilities |
| Van Lieshout Test Short Form [14] | ClinRO | 60 |  |  |  | k=0.5-0.7 |  |  |  | ES=0.5-0.7 |
| Wireless Triaxial Accelerometers [26] | TechO | 12 |  |  |  |  |  |  | Activity logs and bouts of walking: ICC=0.70 |  |
| Wolf Motor Function Test (WMFT) [6,24,13,19,25,7,14] | PerfO | 189 | Unidimentional | Cα = 0.98 | Functional ability (ICC = 0.95) Performance time (ICC = 0.90) | Functional ability (ICC >0.88) Performance time (ICC >0.97) |  |  | Fit statistics =1 item misfit ‘radial deviation test’ |  |

_ADL: activity of daily living, AUC: area under the curve, ClinRO: clinicians-reported outcome, Cα: Cronbacha alpha, ES: effect size, IADL: instrumental activity of daily living, ICC: Interclass Correlation Coefficient, ClinRO: clinician-reported outcome, CR:_ _Coefficients of reproducibility, CS: Coefficients of scalability, IADL: instrumental activity of daily living, ICF: International Classification of Functioning, Health and Disability,K: Kappa, LOA: limits of agreements, ObserO: observation-reported outcome, PerfO: performance-reported outcome, PRO: patients-reported outcome, PLR: positive likelihood ratio, r: Pearson correlation, SRO: self-reported outcome, SOI: source of information, NR: not reported, NLR: negative likelihood ratio, ROC: receiver operating characteristic curve, RR: Response Ration, SOI: source of information, SEM: standardized error of measurement, SRM: standardized root of mean, TechO: technology-reported outcome, WHOQOL: World Health Organisation Quality of Life_

**D. Individuals with traumatic brain injury**

| **Name of the measure** | **SOI** | **Sample** | **Content validity** | **Internal consistency** | **Test-retest** | **Inter-rater** | **Intra-rater** | **Measurement error** | **Construct validity** | **Responsiveness** |
| --- | --- | --- | --- | --- | --- | --- | --- | --- | --- | --- |
| 10-Meter Walking Test (10MWT) [17,2] | PerfO | >100 |  |  | ICC=0.95-0.99 (n=94) | ICC=0.91-0.98 (n=13) |  |  |  |  |
| 6-Minute Walking Test (6MWT) [17,2] | PerfO | 36 |  |  | ICC=0.94-0.96 | ICC=0.94-0.96 |  |  |  |  |
| Brain injury community rehabilitation outcome scale (BICRO) [16] | PRO | 127 | Focus groups | Cα= 0.70-0.94 | ICC=0.77 | k>0.7 |  |  | r>0.70 |  |
| Community balance and mobility scale (CB&M) [2] | PerfO | 32 |  |  |  | ICC=0.98 | ICC=0.98 |  | Gait speed r=0.64 |  |
| European Quality of life scale (EQ5D) [35] | PRO | 86 |  |  | ICC=0.78 |  |  |  |  |  |
| Functional Independence Measure (FIM) [35] | ClinRO | 332 | In a Rasch analysis of the FIM, 2 separate domains of items were defined: the motor domain consisting of 13 items and the cognitive domain consisting of 5 items. |  | r=0.90 |  |  |  |  |  |
| Grooved Pegboard Test (GPT) [35] | ClinRO | NR |  |  | r=0.67-0.86 |  |  |  | Tapping Speed: r=0.35; Near Visual Acuity: r=0.62 Reaction Time:r=0.31;  Digit Symbol: r=0.60; Block Design: r=0.34; Object Assembly: r=0.45 |  |
| High Level Mobility Assessment (HiMAT) [2] | PerfO | 103 | Experts |  |  | ICC=0.99 | ICC=0.99 |  | Functional Independence Measure: r=0.53 Rivermead Mobility Index: r=0.87 |  |
| Mayo-Portland Adaptability Inventory (MPAI-4) [35] | PRO | 339 | Experts | Cα =0.89 |  | r=0.88-0.99 |  |  | Disability rating scale: r=0.81 Goal Attainment Scaling : r=0.47 Independent Living Scale: r=0.26 Vocational Independence Scale: r=0.32 |  |
| Medical Outcomes Study 36-Item Short Form Health Survey (SF-36) [35] | PRO | NR | Experts | Cα = 0.79-0.92 |  |  |  |  | Beck depression inventory: r=0.77 |  |
| Pens taped to feet [2] | ClinRO | 12 |  |  |  |  | ICC=0.94-1 |  | Step length and width: r=0.93 |  |
| Rivermead mobility index (RMI) [2] | SRO | 20 |  |  |  |  |  |  | Barthel Index: r=0.91 Functional Assessment Category: r=0.89 Gait speed: r=0.82 Bohannon Balance Scale: r=0.82 6-Minute Walking Test: r=0.63 |  |
| Satisfaction With Life Scale (SWLS) [35] | PRO | NR | Initially 48 items were included; factor analysis showed that 10 items loaded highly (0.60) on a factor reflecting cognitive judgmental evaluative processes; 5 items were redundant, resulting in the current 5-item scale |  | ICC=0.89 |  |  |  |  |  |
| Sickness Impact profile (SIP) [16] | PRO | 25 | Focus groups | Cα = 0.70-0.91 | ICC=0,77 | k>0.7 |  |  | r>0.70 | ES=0.5-0.9 |
| Timed Up and Go test (TUG) [17] | PerfO | 24 |  |  | ICC=0.86 |  |  |  |  |  |
| Functional Arm Activity Behavioral Observation System (FAABOS) [13] | ObserO | 9 |  |  |  | ICC≥0.75 |  |  |  |  |
| Trunk Recovery Scale (TRS) [10] | ClinRO | 59 |  | Cα =0.9 |  | ICC=0.97 |  | SEM= 8.81 - 11.15 | Trunk Control Test: r=0.94 Functional independence Measure: r=0.85 |  |

_ClinRO: clinicians-reported outcome, Cα: Cronbacha alpha, ES: effect size, ICC: Interclass Correlation Coefficient K: Kappa, ObserO: observation-reported outcome, PerfO: performance-reported outcome, PRO: patients-reported outcome, r: Pearson correlation, SRO: self-reported outcome, SEM: standardized error of measurement, SOI: source of information, NR: not reported_

**References**

1. Scrivener, K., Sherrington, C., & Schurr, K. (2013). A systematic review of the responsiveness of lower limb physical performance measures in inpatient care after stroke. *BMC neurology, 13*(1), 4.
2. Tyson, S., & Connell, L. (2009). The psychometric properties and clinical utility of measures of walking and mobility in neurological conditions: a systematic review. *Clinical rehabilitation, 23*(11), 1018-1033.
3. Salbach, N. M., O'brien, K. K., Brooks, D., Irvin, E., Martino, R., Takhar, P., et al. (2017). Considerations for the selection of time-limited walk tests poststroke: a systematic review of test protocols and measurement properties. *Journal of Neurologic Physical Therapy, 41*(1), 3-17.
4. Gebruers, N., Vanroy, C., Truijen, S., Engelborghs, S., & De Deyn, P. P. (2010). Monitoring of physical activity after stroke: a systematic review of accelerometry-based measures. *Archives of physical medicine and rehabilitation, 91*(2), 288-297.
5. Salter, K., Jutai, J., Teasell, R., Foley, N., Bitensky, J., & Bayley, M. (2005). Issues for selection of outcome measures in stroke rehabilitation: ICF activity. *Disability and Rehabilitation, 27*(6), 315-340.
6. Barak, S., & Duncan, P. W. (2006). Issues in selecting outcome measures to assess functional recovery after stroke. *NeuroRx, 3*(4), 505-524.
7. Sivan, M., O'Connor, R. J., Makower, S., Levesley, M., & Bhakta, B. (2011). Systematic review of outcome measures used in the evaluation of robot-assisted upper limb exercise in stroke. *Journal of Rehabilitation Medicine, 43*(3), 181-189.
8. Gor-García-Fogeda, M. D., Molina-Rueda, F., Cuesta-Gómez, A., Carratalá-Tejada, M., Alguacil-Diego, I. M., & Miangolarra-Page, J. C. (2014). Scales to assess gross motor function in stroke patients: a systematic review. *Archives of physical medicine and rehabilitation, 95*(6), 1174-1183.
9. Salter, K., Jutai, J., Teasell, R., Foley, N., & Bitensky, J. (2005). Issues for selection of outcome measures in stroke rehabilitation: ICF Body Functions. *Disability and Rehabilitation, 27*(4), 191-207.
10. Sorrentino G., S. P., Solaro C., Rabini A., Cerri C., Ferriero G. (2018). Clinical measurement tools to assess trunk performance after stroke: a systematic review. *European journal of physical and rehabilitation medicine*.
11. Geroin, C., Mazzoleni, S., Smania, N., Gandolfi, M., Bonaiuti, D., Gasperini, G., et al. (2013). Systematic review of outcome measures of walking training using electromechanical and robotic devices in patients with stroke. *Journal of rehabilitation medicine, 45*(10), 987-996.
12. Teale, E. A., & Young, J. B. (2010). A review of stroke outcome measures valid and reliable for administration by postal survey. *Reviews in Clinical Gerontology, 20*(4), 338-353.
13. Lemmens, R. J., Timmermans, A. A., Janssen-Potten, Y. J., Smeets, R. J., & Seelen, H. A. (2012). Valid and reliable instruments for arm-hand assessment at ICF activity level in persons with hemiplegia: a systematic review. *BMC neurology, 12*(1), 21.
14. Velstra, I.-M., Ballert, C. S., & Cieza, A. (2011). A systematic literature review of outcome measures for upper extremity function using the international classification of functioning, disability, and health as reference. *PM&R, 3*(9), 846-860.
15. Connell, L. A., & Tyson, S. F. (2012). Clinical reality of measuring upper-limb ability in neurologic conditions: a systematic review. *Archives of physical medicine and rehabilitation, 93*(2), 221-228.
16. Ashford, S., Brown, S., & Turner-Stokes, L. (2015). Systematic review of patient-reported outcome measures for functional performance in the lower limb. *Journal of rehabilitation medicine, 47*(1), 9-17.
17. Stevens, P. M. (2010). Clinimetric properties of timed walking events among patient populations commonly encountered in orthotic and prosthetic rehabilitation. *JPO: Journal of Prosthetics and Orthotics, 22*(1), 62-74.
18. van Bloemendaal, M., van de Water, A. T., & van de Port, I. G. (2012). Walking tests for stroke survivors: a systematic review of their measurement properties. *Disability and Rehabilitation, 34*(26), 2207-2221.
19. Rowland, T. J., & Gustafsson, L. (2008). Assessments of upper limb ability following stroke: a review. *British Journal of Occupational Therapy, 71*(10), 427-437.
20. Ashford, S., Slade, M., Malaprade, F., & Turner-Stokes, L. (2008). Evaluation of functional outcome measures for the hemiparetic upper limb: a systematic review. *Journal of rehabilitation medicine, 40*(10), 787-795.
21. Tse, T., Douglas, J., Lentin, P., & Carey, L. (2013). Measuring participation after stroke: a review of frequently used tools. *Archives of physical medicine and rehabilitation, 94*(1), 177-192.
22. Van Peppen, R. P., Hendriks, H., Van Meeteren, N. L., Helders, P. J., & Kwakkel, G. (2007). The development of a clinical practice stroke guideline for physiotherapists in The Netherlands: a systematic review of available evidence. *Disability and Rehabilitation, 29*(10), 767-783.
23. Baker, K., Cano, S. J., & Playford, E. D. (2011). Outcome measurement in stroke: a scale selection strategy. *Stroke, 42*(6), 1787-1794.
24. Hong, I., & Bonilha, H. S. (2017). Psychometric properties of upper extremity outcome measures validated by Rasch analysis: a systematic review. *International Journal of Rehabilitation Research, 40*(1), 1-10.
25. Simpson, L. A., & Eng, J. J. (2013). Functional recovery following stroke: capturing changes in upper-extremity function. *Neurorehabilitation and neural repair, 27*(3), 240-250.
26. Fini, N. A., Holland, A. E., Keating, J., Simek, J., & Bernhardt, J. (2015). How is physical activity monitored in people following stroke? *Disability and Rehabilitation, 37*(19), 1717-1731.
27. Croarkin, E., Danoff, J., & Barnes, C. (2004). Evidence-based rating of upper-extremity motor function tests used for people following a stroke. *Physical therapy, 84*(1), 62-74.
28. Martins, J. C., Aguiar, L. T., Nadeau, S., Scianni, A. A., Teixeira-Salmela, L. F., & Faria, C. D. C. D. M. (2019). Measurement properties of self-report physical activity assessment tools for patients with stroke: a systematic review. *Brazilian journal of physical therapy, 23*(6), 476-490.
29. Pollock, C., Eng, J., & Garland, S. (2011). Clinical measurement of walking balance in people post stroke: a systematic review. *Clinical rehabilitation, 25*(8), 693-708.
30. Pearson, O. R., Busse, M., Van Deursen, R. W. M., & Wiles, C. M. (2004). Quantification of walking mobility in neurological disorders. *Qjm, 97*(8), 463-475.
31. Oczkowski, C., & O'Donnell, M. (2010). Reliability of proxy respondents for patients with stroke: a systematic review. *Journal of Stroke and Cerebrovascular Diseases, 19*(5), 410-416.
32. Salter, K., Jutai, J., Teasell, R., Foley, N., Bitensky, J., & Bayley, M. (2005). Issues for selection of outcome measures in stroke rehabilitation: ICF Participation. *Disability and Rehabilitation, 27*(9), 507-528.
33. Silva, P. F., Quintino, L. F., Franco, J., & Faria, C. D. (2014). Measurement properties and feasibility of clinical tests to assess sit-to-stand/stand-to-sit tasks in subjects with neurological disease: a systematic review. *Brazilian journal of physical therapy, 18*(2), 99-110.
34. Verheyden, G., Nieuwboer, A., Van de Winckel, A., & De Weerdt, W. (2007). Clinical tools to measure trunk performance after stroke: a systematic review of the literature. *Clinical rehabilitation, 21*(5), 387-394.
35. Wilde, E. A., Whiteneck, G. G., Bogner, J., Bushnik, T., Cifu, D. X., Dikmen, S., et al. (2010). Recommendations for the use of common outcome measures in traumatic brain injury research. *Archives of physical medicine and rehabilitation, 91*(11), 1650-1660. e1617.
