# Supplementary material for: Quality of mobility measures among individuals with acquired brain injury: an umbrella review
Source: Qual Life Res. 2022 Mar 11;31(9):2567–99. doi: 10.1007/s11136-022-03103-4 (PMC9356944; doi:10.1007/s11136-022-03103-4)
Supplement: Supplementary file 8 — Supplementary file8 (DOCX 19 kb) [file 11136_2022_3103_MOESM8_ESM.docx]

**Fig. 3 PRISMA Flowchart of the study selection process**

Potentially relevant systematic reviews identified through database searching
n= 1290

## Screening

## Included

## Eligibility

## Identification

Systematic reviews duplicated
(n = 175)

Systematic reviews screened
(n = 1115)

Systematic reviews excluded
(n = 1115)

Reasons:

- Not ABI: (n=326)
- Interventions: (n=306)
- Not mobility measures: (n=158)
- Not SR: (n=42)
- Not related to the aim of the study: (n=204)

Full-text systematic reviews assessed for eligibility
(n = 79)

Full-text systematic reviews excluded with reasons
(n = 47)

Reasons:
- Not mobility measures: (n=7)
- No psychometric properties provided: (n=13)
- Non-structured reviews: (n=10)
- ABI <50%, mixed with orthopedic conditions: (n=6)
- Protocol: (n=1)
- Not measurement study (n=10)

Systematic reviews included in the umbrella reviews
(n = 35)

Number of mobility measures (n=320)

Number of mobility measures

(n=147)

*some measures were used in multiple settings

Mobility measures duplicated

(n=173)

Traumatic brain injury (n=17)

- ClinRO (n=4)

- PerfO (n=5)

- ObserO (n=1)

- PRO (n=6)

- SRO (n=1)

Stroke at chronic setting (n=123)

- ClinRO (n=38)

- PerfO (n=31)

- ObserO (n=1)

- PRO (n=23)

- SRO (n=8)
- TechO (n=22)

Stroke at sub-acute setting (n=14)

- ClinRO (n=1)

- PerfO (n=7)

- PRO (n=2)

- SRO (n=2)

- TechO (n=2)

Stroke at acute setting (n=36)

- ClinRO (n=11)

- PerfO (n=15)

- PRO (n= 4)

- SRO (n=2)

- TechO (n=4)
