# Supplementary material for: Quality of mobility measures among individuals with acquired brain injury: an umbrella review
Source: Qual Life Res. 2022 Mar 11;31(9):2567–99. doi: 10.1007/s11136-022-03103-4 (PMC9356944; doi:10.1007/s11136-022-03103-4)
Supplement: Supplementary file 9 — Supplementary file9 (DOCX 43 kb) [file 11136_2022_3103_MOESM9_ESM.docx]

**Table 4. Interpretability and feasibility of mobility measures**

| Name of the measure | Type of population | Setting | Floor and Ceiling effect | Normative data | MID/MIC | Ease of administration | Length of the instruments | Ease of score calculation | Cost | Required equipment |
| --- | --- | --- | --- | --- | --- | --- | --- | --- | --- | --- |
| Clinician-Reported Outcomes (ClinROs) | | | | | | | | | | |
| Action research arm test (ARAT)[63,24,56] | Stroke | Chronic | Yes | NR | MDC=6 points | Yes  Paper and pencil | 5-15 min | Yes | Not Free | Various sized wood blocks, cricket ball, stone, jug and glasses, a small and larget tube, washer and bolt, ball bearing, a marble, a chair without arm rests, a table, a plank, a tin lid/not required training |
| Brunel Balance Assessment[51] | Stroke | Chronic | NR | NR | MCID= one level  (1/12) due to hierarchical scale | Yes | 10 min | Yes | Free | Plinth or suitable seating, ruler, step up block, stopwatch, tape to mark 5m walkway, 2 stools/required training (Reading articles/Manual) |
| Box and block test (BBT)[63,56] | Stroke | Chronic | NR | NR | MDC=6 block (weak hand);  8 blocks (sound hand)  MCID=6 min | Yes  Paper and pencil | 2-5 min | Yes | Not Free | Stopwatch, wooden box, partition, 150 wooden cubes/not required training |
| Chedoke McMaster stroke assessment scale (CMSA)[43,18,56] | Stroke | Chronic | Yes | NR | MDD= 8 points | Yes  Paper and pencil | 45 min-1 hour | Yes | Free | An adjustable table, chair with armrests, floor mat, pillows, a pitcher with water, a measuring cup, a ball 2.5 inch in diameter, a footstool, a 2m line marked on the floor, stopwatch/required training |
| Functional ambulation category (FAC)[53,25,60] | Stroke | Chronic | NR | NR | MCID =4.36 to 17.70  SDD= 1 point | Yes  Paper and pencil | 1 min | Yes | Free | No/not required training |
| Functional ambulation category (FAC)[49,26] | Stroke | Sub-acute | NR | NR | SDD=1 point | Yes  Paper and pencil | 1 min | Yes | Free | No/not required training |
| Functional gait assessment (FGA)[46,25,26] | Stroke | Chronic | NR | NR | MDC=4.2 m | Yes  Paper and pencil | 5-20 min | No | Free | Stopwatch, measuring device to mark off area, obstacles, set of steps/not required training |
| Fugl-Mayer Assessment (FMA)[16] | Stroke | Acute | Yes | NR | MCID was estimated to be  approximately 10% of total scale score | Yes  Paper and pancil | 30-45 min | Yes | Free | Tennis ball, a small spherical shaped container, a tool to administer reflex tests, enough space is needed for a patient to move around freely, if possible, space should be a quite, private room with few distractions/required training (Reading article/manual) |
| Functional Test for the Hemiplegic Upper Extremity (FTHUE)[48] | Stroke | Chronic | Yes | NR | MCD= 9 mm | Yes | NR | Yes | Free | NR |
| Functional independence measure (FIM)[43,24,56] | Stroke | Acute | Yes | NR | FIM change scores from admission to discharge associated with MCID were 22, 17, 3 points for the total FIM, motor FIM and cognitive FIM respectively MCID=11 points | Yes  Paper and pencil | 20-45 min | Yes | Free (need a license) | May vary based on level and impairment category measured/required training (Reading articles/Manual) |
| Grip strength[56] | Stroke | Chronic | NR | NR | MCID=2.9 kg | Yes | 5 min | Yes | Not Free | Yes/requires purchase of a handheld dynamometer/required training (reading articles/manual) |
| Motor evaluation scale for upper extremity in stroke patients (MESUPES)[48] | Stroke | Chronic | NR | NR | MDC for MESUPES total score (/58) at 95% CI = 7.43 points with the 3 outliers  MDC95%CI for MESUPES-arm test (/40) = 6.10  points  MDC95%CI for MESUPES-hand test (/18) = 2.61points | Yes  Paper and pencil | 5-15 min | Yes | Free | Mat, desk and chair, ruler, plastic bottle, dice/not required training |
| Motricity index (MI)[43,60] | Stroke | Chronic | NR | NR | MDD=12 points (arm) MDD= 13 points (leg) MCD (arm): 11 points, (Leg): 25 points) | Yes | 5-20 min | Yes | Not Free | 2.5 cm * 2.5 cm cube/not required training |
| Nine hole pig test (NHPT)[63,56] | Stroke | Chronic | NR | NR | MDC=32.8 sec. (more affected hand); 6.2 sec (less affected hand) MCID=32 sec | Yes  Paper and pencil | 2 - 10 min | Yes | Free | Wood or plastic with 9 holes, a container for the pegs, 9 pegs, stopwatch/not required training. |
| Trunk Control Test (TCT)[60] | Stroke | Chronic | NR | NR | SDD= 25 points | Yes  Paper and pencil | 5-10 min | NR | Free | Bed or treatment table.  Required training (Reading articles/Manual) |
| Mini Mental State Examination (MMSE)[43] | Stroke | Chronic | NR | NR | A score of 23 is the generally accepted cut off  point indicating presence of cognitive  impairment | Yes  Paper and pencil | 10 min | Yes | Free | Yes  Score sheet that demonstrate figure to copy, writing instrument/not required training |
| Performance-Reported Outcomes (PerfOs) | | | | | | | | | | |
| Arm mobility ability test (AMAT)[24] | Stroke | Sub-acute | NR | NR | AMAT detected the difference in change occurring as a result of the passage of 1 versus 2 weeks in sub- acute patients | Yes | 30-60 min | Yes | Not Free | Silverware and plate, play-doh, mug, comb, foam sandwich, towel, jar, shirts, light switch, door, dried beans, shoe and shoelaces, telephone/required training (reading articles/manual) |
| Barthel index (BI)[43,18,56,60] | Stroke | Chronic Acute | Yes | NR | MCID=16 points MDD= 4 points | Yes  Papaer and pencil | 5-15 min | Yes | Free | No |
| Berg balance scale (BBS)[43,45,46,18,26,60] | Stroke | Acute | Yes | NR | MDC90= 5.8 points  MDC95= 6.9 points  SDD= 6 points | Yes  Paper and pencil | 10-15 min | Yes | Free | Stopwatch, standard height chair (18-20 inch) with and without armsets, step or stool of average height (7.75-9 inch), ruler, slipper or shoe/not required training |
| Berg balance scale (BBS)[43,46,18,26,60] | Stroke | Chronic | Yes | NR | MDD= 6 points MIC= 3 points | Yes  Paper and pencil | 10-20 min | Yes | Free | Stopwatch, standard height chair (18-20 inch) with and without armstep or stool of average height (7.75-9 inch), ruler, slipper or shoe/not required training |
| Balance Evaluation System test (BESTest)[51] | Stroke | Chronic | NR | NR | MCID= 3 points | Yes | 20-30 min | Yes | Free | Stopwatch, measuring tape mounted on wall, a block, 10-degree incline ramp, stair step, shoe boxes, 2.5 kg free weight, firm chair with arms with 3 m in front marked with tape, measuring tape/required training (Reading articles/Manual) |
| Chedoke arm and hand inventory (CAHAI)[24,56] | Stroke | Chronic | Yes | NR | MCID=6.3 points | Yes  Paper and pencil | 25 min | Yes | Free | Jar of coffee, phone, ruler and pen, toothpaste and toothbrush, knife, fork, putty, glass of water, wet washcloth, eyeglasses, jacket and zipper, shirt with 5 buttons, towel, rubbermaid 38 liter container, plastic grocery bag with 4 pounds weight/required training (reading articles/manual) |
| Dynamic gait index (DGI)[26] | Stroke | Sub-acute | Yes | NR | MDC=4 m | Yes  Paper and pencil | 10-15 min | No | Free | Shoe box, two obstacles, stairs, 6 m pathway/not required training |
| Five meter walking test (5MWT)[43,52,53,25,26,60] | Stroke | Chronic | NR | NR | MDC90= 19.3  MDC95= 24.5  MDC=4.5 second (aid); 1.12 (no aid) MDC (with aid)=4.5; MDC (no aid)=1.12 s; MDC=0.3 m/s) | Yes  Paper and pencil | 6-10 min | Yes | Free | Stopwatch  Clear pathway 5-meter length/not required training |
| Five times sit to stand test (5x STST)[56] | Stroke | Chronic | NR | NR | MDC=5 | Yes  Paper and pencil | < 5 min | Yes | Free | Stopwatch and standard height chair with a backrest/not required training |
| Fugl-Mayer Assessment (FMA)[43,56] | Stroke | Chronic | Yes | NR | MCID=7 points  MCID= 10 points | Yes  Paper and pancil | 20-40 min | Yes | Free | Tennis ball, a small spherical shaped container, a tool to administer reflex tests, enough space is needed for a patient to move around freely, if possible, space should be a quite, private room with few distractions/required training (reading article/manual) |
| High level mobility assessment (HiMAT)[25] | TBI |  | NR | NR | MDC=1.36 | Yes  Paper and pencil | < 10 min | No | Free | Stopwatch, tape measure, house brick, 20m walkway, stairs/requiring training (reading articles/manual) |
| Rivermead mobility assessment (RMA)[43,18,56] | Stroke | Chronic | Yes | NR | MCID= 3 points  MCD= 2points | Yes  Paper and pencil | 20-45 min | Yes | Free | Block of 20 cm height, pencil, volleyball, tennis ball, piece of paper, fork and knife, plate and container, beanbag, cord, putty, watch with chronometer, non-slip mat/not require training |
| Six meter walking test (6MWT)[43,46,52,25] | Stroke | Chronic | Yes | NR | MCD=0.15 m/s at usual pace; 0.25 m/s at fast pace)  MIC:50 m MCID: 54m  MDC90%=28.6-42.1 m  MDC95%=50.2 m | Yes  Paper and pencil | 6-10 min | Yes | Free | Stopwatch  Clear pathway 6 meter length/not required training |
| Six meter walking test (6MWT)[26] | Stroke | Sub-acute | Yes | NR | MDC= 54.1 m | Yes  Paper and pencil | 6 min | Yes | Free | Stopwatch  Clear pathway 6 meter length/not required training |
| Six meter walking test (6MWT)[52,25] | Stroke | Acute | Yes | NR | MDC=54.1  MDC=61  MDC=39 | Yes  Paper and pencil | 6 min | Yes | Free | Stopwatch  Clear pathway 6 meter length/not required training |
| Six meter walking test (6MWT)[25] | TBI |  | Yes | NR | MDC =82 m; 0.25 m/s (fast pace)  MDC= 0.18 (comfortable) | Yes  Paper and pencil | < 10 min | Yes | Free | Stopwatch  Clear pathway 6 meter length/not required training |
| Time up and go test (TUG)[43,18,25] | Stroke | Chronic | Yes | NR | MDC=1.63 m/s | Yes  Paper and pencil | < 3 min | No | Free | Standardized armchair, stopwatch/not required training |
| Time up and go test (TUG)[43] | TBI |  | Yes | NR | MDD= 14 sec | Yes  Paper and pencil | < 10 min | No | Free | Bed or treatment table/required training (reading articles/manual) |
| Ten meter walking test (10MWT)[43,46,25,60] | Stroke | Chronic | Yes | NR | MDD=0.16 m/s SDD=0.16 m/s  MCIC=0.4 m/s (household ambulation)  MCIC= 0.4-0.8 m/s ( limited  community ambulation)  MCIC= 0.8 m/s (community ambulation)  MDC=0.19m/s | Yes  Paper and pencil | 5-10 min | Yes | Free | Stopwatch  Clear pathway 10 meter length/not required training |
| Ten meter walking test (10MWT)[25] | TBI |  | Yes | NR | MDC=0.19 m/s | Yes  Paper and pencil | 10 min | Yes | Free | Stopwatch  Clear pathway 10 meter length/not required training |
| Ten meter walking test (10MWT)[46,26] | Stroke | Sub-acute | Yes | NR | MCID=0.16 m/s | Yes  Paper and pencil | 5-10 min | Yes | Free | Stopwatch  Clear pathway 10 meter length/not required training |
| Two meter walking test (2MWT)[52] | Stroke | Chronic | NR | NR | MDC=11.2-11.6 m | Yes  Paper and pencil | < 10 min | Yes | Free | Stopwatch  Clear pathway 2 meter length/not required training |
| Wolf motor functional test (WMFT)[43,24,56] | Stroke | Chronic | Yes | Preliminary normative data have been collected from able-bodied participants (age range 40-80 years), which clinicians may find useful for interpreting client scores and goal setting | MDD= 0.04 sec  MCID=12 points | Yes  Computer | 30-40 min | Yes | Free | Standerdized table and chair, box, wrist weight, unopened can, pencip, paper clip, checkers, note cards, standerdized lock and key board, face towel, basket, dynamometer, stopwatch/not required training |
| Patient-Reported Outcomes (PROs) | | | | | | | | | | |
| ABILHAND[62,63,24] | Stroke | Chronic | Yes | NR | Yes | Yes  Paper and pencil | 10-30 min | Yes | Free | Paper questionnaire/required training (reading articles/manual) |
| Beck depression inventory (BDI)[43] | Stroke | Chronic | NR | NR | A score of 10 is generally accepted cut off  score for the indication of possible depression | Yes  Paper and pencil | 10 min | Yes | Not free | Testing form and writing instrument/not required training |
| Mayo-Portland Adaptability Inventory (MPAI-4)[64] | TBI |  | Yes | NR | MCID= 5 times positive changes | Yes | minutes | Yes | Free | No/not requiring training |
| Modified Rankin handicap scale[43] | Stroke | Chronic | NR | NR | Score of 2 reflects a good outcome | Yes  Paper and pencil | 5-15 min | Yes | Free | No/not required training |
| Nottingham leisure activity (NLA)[17] | Stroke | Chronic | Yes | Yes | Yes | Yes  Paper and pencil | 5-15 min | Yes | Free | No/not requiring training |
| Short form 36 health survey questionnaire (SF-36)[17] | Stroke | Chronic | Yes | Yes | Yes | Yes  Paper and pencil | 10-60 min | Yes | Free | No/requiring training (reading articles/manual |
| Sickness impact profile (SIP)[43,17] | Stroke | Chronic | Yes | Yes | Patients with a total score of 33 have poor health profiles | Yes  Paper and pencil | 20-30 min | Yes | Free | No^/n^ot requiring training. |
| Sickness impact profile (SIP)[21,17] | TBI |  | NR | NR | SDD=10.51 | Yes  Paper and pencil | NR | Yes | Free | No/not requiring training. |
| Stroke impact scale (SIS)[43,17,56,58] | Stroke | Sub-acute Chronic | A floor effect in hand function was reported in moderate stroke patients (40.2%) and a ceiling effect in the communication domain among mild-moderate stroke patients (35% vs. 67.5 % Barthel index ceiling effect).  Rasch analysis confirmed these two effects-a ceiling effect in the memory and emotion domains was also reported composite physical function domain displayed floor and ceiling effects of less than 3%. | Ceiling effects noted in communication and memory domains. Floor effect in hand function domain. Study population 96% male and limited to those with no co-morbidity and good potential from rehabilitation. | MDD= 10-15 points | Yes  Paper and pencil | 10-20 min | Yes | Free | No/requiring training (reading articles/manual) |
| Stroke specific quality of life scale (SSQOL)[43,17] | Stroke | Chronic | Yes | Yes | Yes | Yes  Paper and pencil | 10-15 min | Yes | Free | No/not requiring training |
| Self-Reported Outcomes (SROs) | | | | | | | | | | |
| Duruoz Hand Index (DHI)[48] | Stroke | Chronic | NR | NR | MDC= 1.4 points | Yes  Paper and pencil | 3-7 min | Yes | Free | Yes/paper survey and writing utensil/not required training |
| Disabilities of the Arm, Shoulder and Hand (DASH)[23] | Stroke | Chronic | Yes | NR | MDC= 10 points | Yes  Paper and pencil | 5-30 min | Yes | Free | No/not required training |
| Frenchay activities index (FAI)[43,56,60] | Stroke | Acute, Chronic | Yes | NR | SDD=1.3 points  Patients with a score of  15 are classified as “inactive.” | Yes  Paper and pencil | 5-10 min | Yes | Free | No/pencil and form/not required training |
| Motor activity log-14 items[62,63,24] | Stroke | Chronic | Yes | NR | LoA < ±1 point | Yes  Paper and pencil | 20 min | Yes | Free | Yes survey instruments/required training (reading articles/manual) |
| Rivermead mobility index (RMI)[46,25,21,49,63] | Stroke | Acute, Sub-acute, Chronic | Yes | NR | MIC= 3 points  MDC= 2 points | Yes  Paper and pencil | 4 -15 min | Yes | Free | No/not required training |
| Rivermead mobility index (RMI)[25] | TBI |  | Yes | NR | MDC= 2 point | Yes  Paper and pencil | < 10 min | Yes | Free | No/not required training |
| Technology-Reported Outcomes (TechOs) | | | | | | | | | | |
| Step activity monitor (SAM)[45,25] | Stroke | Chronic | NR | Extreme slow stride counts (mean/d steps/d: 2837-1503) compared with norms for older and/or sedentary adults (5000-7000) | NR | Yes | > 1 hour | No | Pricing provided by company upon request | Step watch, computer/required training (reading articles/manual) |
| Triaxial accelerometer/RT3[22,45] | Stroke | Chronic | NR | NR | MDD=23% | Yes | NR | No | Pricing provided by company upon request | Yes computer/required training (reading articles/manual) |

_CI: confidence interval, cm: centimetre, Kg: kilogram, LoA: limits of agreement, MID: minimal importance difference, MIC: minimal importance change, MCID: minimal clinical important difference, MDD: minimal detectable difference, MDC: minimal detectable change, MCIC: minimal clinical important change, min: minute, NR: not reported, SDD: smallest detectable difference, TBI: traumatic brain injury_

_Acute rehabilitation phase refers to a duration of 24 hours after stroke onset and for medically stable patients, lasts 5–7 days [83]; Sub-acute rehabilitation phase refers to a duration of 1 to 6 months where the functional recovery and long-term health status are more affected [84]; Chronic rehabilitation phase begins once the person is discharged home [83].We did not include the recovery phase for TBI because it was not defined clearly in the literature_.
